# Identification of chemosensory genes from the antennal transcriptome of *Semiothisa cinerearia*

**DOI:** 10.1371/journal.pone.0237134

**Published:** 2020-08-07

**Authors:** Panjing Liu, Xiaofang Zhang, Runjie Meng, Chang Liu, Min Li, Tao Zhang

**Affiliations:** 1 Institute of Plant Protection, Hebei Academy of Agriculture and Forestry Sciences/Integrated Pest Management Center of Hebei Province/Key Laboratory of IPM on Crops in Northern Region of North China, Ministry of Agriculture, Baoding, P. R. China; 2 Baoding Vocational and Technical College, Baoding, P. R. China; 3 College of Plant Protection, Nanjing Agricultural University, Nanjing, P. R. China; USDA Agricultural Research Service, UNITED STATES

## Abstract

Olfaction plays vital roles in the survival and reproduction of insects. The completion of olfactory recognition requires the participation of various complex protein families. However, little is known about the olfactory-related proteins in *Semiothisa cinerearia* Bremer et Grey, an important pest of Chinese scholar tree. In this study, we sequenced the antennal transcriptome of *S*. *cinerearia* and identified 125 olfactory-related genes, including 25 odorant-binding proteins (OBPs), 15 chemosensory proteins (CSPs), two sensory neuron membrane proteins (SNMPs), 52 odorant receptors (ORs), eight gustatory receptors (GRs) and 23 ionotropic receptors (IRs). BLASTX best hit results and phylogenetic analyses indicated that these genes were most identical to their respective orthologs from *Ectropis obliqua*. Further quantitative real-time PCR (qRT-PCR) analysis revealed that three ScinOBPs and three ScinORs were highly expressed in male antennae, while seven ScinOBPs and twelve ScinORs were female-specifically expressed. Our study will be useful for the elucidation of olfactory mechanisms in *S*. *cinerearia*.

## Introduction

Chemoreception, perceived through the olfactory system, plays a critical role in insect behavior (e.g., seeking hosts, selecting mates, locating oviposition sites, and avoiding adverse environments) [1–3]. The antennae, as the main olfactory organs of insects, comprise considerable numbers of olfactory sensilla, where chemical signals are recognized through olfactory receptor neurons (ORNs) [4]. When the external odorants enter the sensillum lymph, the binding proteins specifically bind to the odorants and transport them to the olfactory receptors on the neuron membrane, in which odorant stimulation is transformed into electric signals. Subsequently, these odorants are degraded by odorant degrading enzymes (ODEs) in the lymphatic cavity or in the sensor cells [5–7]. This complex process involves several important families, including odorant-binding proteins (OBPs), chemosensory proteins (CSPs), sensory neuron membrane proteins (SNMPs), olfactory receptors (ORs), gustatory receptors (GRs), ionotropic receptors (IRs) and ODEs [8].

OBPs and CSPs, abundant in the antennae and mouthparts, are involved in binding and transporting various chemical signals [9]. Insect OBPs are soluble proteins, that are typically defined based on expression in the antennae [10, 11]. Recent studies, however, have revealed that OBPs also existed in non-olfactory organs (e.g. legs, wings and mouthparts) [9]. Generally, the classic OBP has a conserved pattern of six cysteines that form three disulfide bridges, while Minus-C OBPs and Plus-C OBPs have two and more than three disulfide bridges, respectively [8, 12]. Insect OBPs can specifically recognize and transmit a variety of signal and environmental molecules. Based on ligand binding assays, OBPs can function as pheromone-binding proteins (PBPs) and general odorant-binding proteins (GOBPs) that recognize various odorants [12, 13]. In *Ectropis obliqua*, EoblOBP6, abundant in antennae and legs, has been shown to bind plant volatiles (e.g. benzaldehyde, nerolidol, α-farnesene) as well as the aversive bitter alkaloid berberine [14]. Recent studies showed that silencing the OBP gene will lead to a decrease in the sensitivity of ORs to specific odors [15, 16]. CSPs, which are also small, soluble proteins, have four highly-conserved cysteines that form two disulfide bridges. CSPs are expressed in chemosensory organs [17, 18], as well as non-olfactory tissues [19, 20]. SNMPs, a homologue of the CD36 protein family, are signaling components crucial for odorant sensitivity [21]. The SNMP family is small, and usually have 2–3 subgroups in Diptera, Lepidoptera and other insect orders [22]. SNMP1 orthologs are mainly expressed in pheromone-sensitive ORNs, and participate in the perception of sex pheromones [21]. *Bombyx mori* SNMP1, a functional orthologue of *Drosophila* SNMP1, functions in pheromone detection by heteromirization with the BmOR1 pheromone receptor and the BmOroco co-receptor [23]. SNMP2s are expressed in supporting cells but their functions are still unclear [21]. SNMP3, a novel SNMP gene subfamily, has been identified in non-olfactory organs of moths and participates in the immunity response [23].

ORs and GRs are seven transmembrane domain membrane proteins located on the dendrite membrane of neurons. Their N-terminus and C-terminus are oriented to the cytoplasmic side and the extracellular sides of the plasma membrane, respectively [24]. Enormous OR diversity has been discovered in various insects. A special OR, called OR co-receptor (Orco), is necessary and highly conserved in many insects [25]. Each OR can bind with a species-specific Orco to form an Orco-ORx complex, that functions as a ligand-gated ion channel that determines the sensitivity and specificity of the ORN where it is expressed [26]. ORs and GRs are mainly responsible for detecting odorants and tastants, respectively. Recent studies also suggested that several GRs recognize carbon dioxide and pheromones [27]. For example, GR21a and GR63a of *Drosophila* are required for carbon dioxide detection, and GR39a is involved in sensing a female pheromone [28]. In addition, GRs are reported to be expressed not only in gustatory organs but also in antennae [27, 29]. Ionotropic receptors (IRs), a new kind of olfactory receptor, are related to ionotropic glutamate receptors (iGluRs) that respond to both environmental and cellular signals and function similar to chemosensory and gustatory receptors [30].

*Semiothisa cinerearia* Bremer et Grey (Lepidoptera: Geometridae) is one of the most important pests of the Chinese scholar tree (*Sophora japonica* L.), which is widely cultivated in the urban greenbelt in China for its significant medicinal and ornamental value. [31]. In recent years, the leaves of Chinese scholar tree have been severely damaged by *S*. *cinerearia* larvae in northern China. Currently, the control of *S*. *cinerearia* heavily relies on pesticides. Excessive usage of pesticides, however, can result in various negative effects on the environment [32]. Pheromone-based pest management strategies have been proven to be efficient and environmentally friendly methods of pest control [33, 34]. To date, sex pheromones have been identified from more than 570 Lepidopteran species. Sex pheromone components of moths can be classified into three types: Type-I (75%), Type-II (15%), and miscellaneous type (10%) [35, 36]. Type-I pheromones are comprised of saturated or unsaturated alcohols, aldehydes and esters. Type-II pheromones commonly are polyenic hydrocarbons and their corresponding epoxide derivatives [35, 36]. The main components of the sex pheromone of *S*. *cinerearia* are *cis*-3,4-epoxy-(Z.Z)-6,9-heptadecadiene, (3Z, 6Z, 9Z)-3,6,9-heptadecatriene and (3Z, 6Z, 9Z)-3,6,9-octadecatriene [37], which are typical Type-II sex pheromones. Although the sex pheromone has been identified, the mechanisms of pheromone and host plant volatile recognition have not yet been clarified.

In the present study, using *S*. *cinerearia* antennal transcriptomes, we identified candidate olfactory-related proteins and used quantitative real-time PCR (qRT-PCR) to examine the expression profile of a subset of the transcripts. Our results provide useful information for further research on pheromone and host plant volatile recognition in insects.

## Materials and methods

### Insect rearing and total RNA extraction

Pupae of *S*. *cinerearia* were collected in July 2018 from soil under a Chinese scholar tree in a lot owned by a private seedling company located in Baoding, China (38.96°N, 115.46°E). The company had sought our assistance in controlling *S*. *cinerearia* because their scholar trees were under attack by the pest. Therefore, no specific permissions were required for the locations/activities; and our studies did not involve endangered or protected species. All pupae were separated by sex and maintained in a dark thermotank (26 ± 2°C and 70% RH) until emergence. The antennae of 3-day-old adults (100 of each sex) were cut off, frozen in liquid nitrogen immediately, and then ground with a mortar and pestle. Total RNA was extracted using TRIzol reagent (TransGen, China) following the manufacturer’s instructions. RNA quality was evaluated as previously described [38]. For transcriptome sequencing, two groups of 100 moths for each sex were used to collect antennae RNA. For qRT-PCR analysis, three biological repeats were conducted, and each RNA sample was extracted from antennae of 30 moths.

### Preparation for transcriptome sequencing

One microgram of high-quality RNA per sample was sent to Novogene (Beijing, China) for constructing cDNA libraries. The NEBNext^®^ Ultra^™^ RNA Library Prep Kit for Illumina^®^ (NEB, USA) was used to generate sequencing libraries following the manufacturer’s recommendations. Briefly, mRNA was purified from total RNA using poly-T oligo-attached magnetic beads. The double-stranded cDNA was synthesized using mRNA as a template and purified with the AMPure XP System (Beckman Coulter, Beverly, USA). Then, cDNA fragments 250~300 bp in length were preferentially selected after end repair and adaptor ligation. Three microliters of USER Enzyme (NEB, USA) was used with size-selected, adaptor-ligated cDNA at 37°C for 15 min followed by 5 min at 95°C before PCR. Then, PCR was performed with Phusion High-Fidelity DNA polymerase, universal PCR primers and Index (X) Primer. Finally, PCR products were purified (AMPure XP system), and library quality was assessed on the Agilent Bioanalyzer 2100 system.

### Clustering and sequencing

The clustering of the index-coded samples was performed on a cBot Cluster Generation System using a TruSeq PE Cluster Kit v3-cBot-HS (Illumina) according to the manufacturer’s instructions. After cluster generation, the library preparations were sequenced on an Illumina HiSeq platform, and 150bp paired-end reads were generated.

### Transcriptome assembly and gene functional annotation

Transcriptome assembly performed using high quality trimmed data and Trinity (r20140413p1) [39]. Corset was used to compare the number of reads and expression patterns of the above transcripts and to cluster the transcripts [40]. Finally, the longest transcript was selected as the unigene. BUSCO v3 was used to evaluate the completeness of the assembled unigenes using the metazoa_odb9 dataset [41].

All unigenes obtained from *S*. *cinerearia* were annotated against the NCBI nonredundant (Nr) protein database using BLASTn and BLASTx with a significant cut-off E-value of < 10^−5^. Then, the blast results were further imported into the Blast2GO pipeline for Gene Ontology (GO) annotation [42]. The functional annotation followed the latest databases (Nr, Nt, Pfam, KOG/COG, Swiss-Prot, KO and GO).

### Analysis of differential gene expression

The abundance of transcripts was calculated by the FPKM (fragments per kilobase per million mapped reads) method [43]. Differential expression analysis of two samples was performed using the DEGseq R package (1.12.0). The P value was adjusted using q value [44] which was defined as q value < 0.005 & |log2(foldchange)| > 1 and was set as the threshold for significantly differential expression.

### Identification and bioinformatic of putative olfactory genes

The putative OBPs, CSPs, SNMPs, ORs, GRs and IRs genes were retrieved from the *S*. *cinerearia* contigs as functional annotation based on our reference antennal transcriptome assembly. All candidate genes were manually checked using the BLASTx program with an E-value of < 10^−5^. All screened olfactory-related genes were given a four-letter code (uppercase first letter of the genus name + lowercase first three letters of the species name) followed by the abbreviation of the gene name and serial numbers [38]. The complete coding region was determined using ORF finder (http://www.ncbi.nlm.nih.gov/gorf/gorf.html).

The signal peptides of the putative OBPs and CSPs were predicted using the SignalP 5.0 server (http://www.cbs.dtu.dk/services/SignalP/). The transmembrane domains (TMDs) of ORs, GRs, IRs and SNMPs were predicted using TMHMM Server v.2.0 (http://www.cbs.dtu.dk/services/TMHMM/). Sequence alignments of OBPs, CSPs, and SNMPs were performed using ClustalX 1.83, and the results were formatted using GeneDoc software (http://nrbsc.org/gfx/genedoc). Secondary structures of full-length candidates were predicted using the online platform psipred (http://bioinf.cs.ucl.ac.uk/psipred/). The protein structure homology-model was constructed using SWISS-MODEL (https://swissmodel.expasy.org/).

### Phylogenetic analysis

A total of 115 OBPs, 153 CSPs, 18 SNMPs, 181 ORs, 154 GRs and 138 IRs sequences were used for phylogenetic tree reconstruction. All sequences are listed in the supplementary information. Evolutionary analyses were conducted in MEGA7 using the neighbor-joining method with a bootstrap test (1000 replicates) [45]. The evolutionary distances were computed using the Poisson correction method [46]. Finally, phylogenetic trees were viewed and edited using FigTree v. 1.4.3 (http://tree.bio.ed.ac.uk/software/figtree/).

### qRT-PCR analysis

First-strand cDNA was synthesized with 1 μg of total RNA by using All-in-One First-Strand cDNA Synthesis SuperMix (TransGen, China) following the manufacturer’s instructions. qRT-PCR was performed on an ABI 7500 Real-Time PCR System (Applied Biosystems, USA) with specific primers (S1 Table) designed by Premier 6.0. The amplification efficiency of the primers was 92–95% according to the pre-experiment. Then, the expression data analyses were performed using the 2^-ΔΔCT^ method [47]. One-way ANOVA was used to analyze gene expression in SPSS 22.0 software. Finally, graphs were made with Origin8 software. In addition, 17 genes were randomly selected to estimate the consistency between RNA-seq and qRT-PCR data.

## Results

### Transcriptome analysis and assembly

The antennal cDNA library was constructed from female and male *S*. *cinerearia* using the Illumina HiSeq^™^ platform, and the sequences were assembled by the TRINITY de novo program. A total of 25,769,348 (97.67%) and 22,137,323 (97.52%) clean reads were obtained from the transcriptomes of female and male antennae, respectively. After hierarchical clustering, 65,476 unigenes were generated with an N50 length of 1,702 bp. Among those unigenes, 64.01% (41,913) were longer than 500 bp, and 37.02% (24,239) were longer than 1,000 bp (S1 Fig). Furthermore, the evaluation of unigene completeness showed 869 unigenes are complete (88.9%), indicating the high quality of our assembly (S2 Table).

### Functional annotation of unigenes

To obtain comprehensive information on gene function, we annotated the genes using seven databases. A total of 30,805 unigenes (47.04%), 24,197 unigenes (36.95%) and 21,887 unigenes (33.42%) had significantly hits in the Nr, Pfam and Swiss-Prot databases, respectively (S3 Table). Furthermore, there were 6,077 unigenes with matches to all the Nr, Nt, Pfam, KOG, and GO databases (S2A Fig). Among the Nr-hit unigenes, 13,317 genes (43.2%) matched to *B*. *mori*, and 5,439 genes (17.7%) matched to *Danaus plexippus* (S2B Fig).

### Gene ontology and KEGG pathway analysis

To better understand the function of the unigenes, Gene Ontology analysis was carried out on 24,346 unigenes, which were divided into 3 distinct subsets: biological process, cellular component and molecular function (S3 Fig). In the category of biological process, cellular process (13,848) was the largest of 26 groups, followed by metabolic process (11,785). In the cellular component category, the unigenes were mainly enriched in “cell”, “cell part” and “organelle”. In the molecular function category, the annotations were mostly enriched in “binding” (13,546) and “catalytic activity” (9,902) (S3 Fig).

Furthermore, KEGG pathway classification was carried out to enrich the function of unigenes. A total of 12,722 unigenes were divided into five branches covering 36 subgroups: cellular processes, environmental information processing, genetic information processing, metabolism and organismal systems. Among them, “signal transduction” is the most significant pathway, which involved 1606 genes (S4 Fig).

### Candidate OBPs in antennae of *S*. *cinerearia*

A total of 25 transcripts encoding putative OBPs were identified in the antennae of *S*. *cinerearia*, including 20 OBPs, 3 PBPs and 2 GOBPs (Table 1). Of which, 15 OBPs had full-length complete open reading frames (ORFs). The BLASTx results indicated that 12 OBPs (ScinOBP3-4, ScinOBP6, ScinOBP8, ScinOBP10-14, and ScinOBP17-19) and two PBPs (ScinPBP1 and ScinPBP3) had highest amino acid identities (>50%) with those of *E*. *obliqua* (Table 1), a closely related species of *S*. *cinerearia*.

**Table 1:** Summary of OBPs in *Semiothisa cinerearia*.

Gene name	Accession number	Length	Full- length	BlsatX annotation (Reference/Name/Species)	Score	E-value	Identity (%)	Signal Peptide	FPKM	
									Female	Male
ScinOBP1	MT380331	135	Yes	ANG08529.1|odorant-binding protein 31 [*Plutella xylostella*]	167	6E-51	61.42	Yes	1292.56	1399.14
ScinOBP2	MT380332	231	Yes	ALD65883.1|odorant binding protein 9 [*Spodoptera litura*]	260	3E-83	63.56	No	299.12	260.29
ScinOBP3	MT380333	135	No	ALS03851.1|odorant-binding protein 3 [*Ectropis obliqua*]	173	6E-53	60.31	No	2.53	2.6
ScinOBP4	MT380334	185	Yes	ALS03852.1|odorant-binding protein 4 [*Ectropis obliqua*]	372	3E-130	96.74	Yes	16.59	7.78
ScinOBP5	MT380335	169	No	AGA16511.1|odorant-binding protein 7 [*Helicoverpa assulta*]	43.5	0.041	24.62	No	5.34	2.11
ScinOBP6	MT380336	154	Yes	ALS03854.1|odorant-binding protein 6 [*Ectropis obliqua*]	168	1E-50	53.95	Yes	6182.22	5892.8
ScinOBP7	MT380337	150	No	APG32531.1|odorant binding preotein [*Conogethes punctiferalis*]	214	1E-68	70.50	Yes	13.81	14.92
ScinOBP8	MT380338	138	Yes	ALS03856.1|odorant-binding protein 8 [*Ectropis obliqua*]	200	9E-64	68.61	Yes	778.18	675.58
ScinOBP9	MT380339	86	No	QCF41935.1|odorant binding protein [*Athetis dissimilis*]	126	2E-35	79.73	No	46.29	59.95
ScinOBP10	MT380340	191	No	ALS03858.1|odorant-binding protein 10 [*Ectropis obliqua*]	214	7E-68	57.06	Yes	1313.31	770.24
ScinOBP11	MT380341	142	Yes	ALS03859.1|odorant-binding protein 11 [*Ectropis obliqua*]	251	5E-84	82.86	Yes	1434.32	5803.32
ScinOBP12	MT380342	140	No	ALS03860.1|odorant-binding protein 12 [*Ectropis obliqua*]	139	9E-39	52.74	Yes	6360.84	5491.64
ScinOBP13	MT380343	95	No	ALS03860.1|odorant-binding protein 12 [*Ectropis obliqua*]	112	4E-29	67.50	No	244.04	337.73
ScinOBP14	MT380344	123	Yes	ALS03861.1|odorant-binding protein 13 [*Ectropis obliqua*]	189	1E-59	80.73	Yes	4.94	2.31
ScinOBP15	MT380345	100	No	QHI42038.1|odorant-binding protein 9 [*Glyphodes caesalis*]	112	3e-29	60.67	Yes	10.75	2.02
ScinOBP16	MT380346	128	No	NP_001153665.1|odorant binding protein [*Bombyx mori*]	79	4E-16	31.62	No	66.38	83.24
ScinOBP17	MT380347	116	Yes	ALS03865.1|odorant-binding protein 17 [*Ectropis obliqua*]	126	1E-34	73.08	No	1.59	1.4
ScinOBP18	MT380348	144	Yes	ALS03866.1|odorant-binding protein 18 [*Ectropis obliqua*]	184	2E-57	60.00	Yes	213.21	146.8
ScinOBP19	MT380349	243	Yes	ALS03867.1|odorant-binding protein 19 [*Ectropis obliqua*]	278	2.00E-92	64.00	Yes	428.32	270.08
ScinOBP20	MT380350	120	No	ALS03868.1|odorant-binding protein 20 [*Ectropis obliqua*]	99.8	2E-24	38.14	No	9.22	1.62
ScinPBP1	MT380351	172	Yes	ANA75014.2|putative pheromone binding protein 1 [*Ectropis obliqua*]	264	4E-88	73.96	Yes	4496.45	24530.74
ScinPBP2	MT380352	173	Yes	ACT34881.1|pheromone-binding protein 1 [*Bombyx mandarina*]	234	2E-76	65.41	Yes	1506.46	1021.32
ScinPBP3	MT380353	162	Yes	ALS03849.1|pheromone-binding protein 3 [*Ectropis obliqua*]	224	2E-72	70.83	Yes	238.79	185.66
ScinGOBP1	MT380355	140	Yes	P87508.1|General odorant-binding protein 1; [*Antheraea pernyi*]	251	2E-83	83.45	No	3109.41	3656.26
ScinGOBP2	MT380356	173	Yes	AFM36760.1|general odorant-binding protein 2 [*Agrotis ipsilon*]	264	4E-88	75.93	Yes	19641.32	7703.44

**Fig 1:**
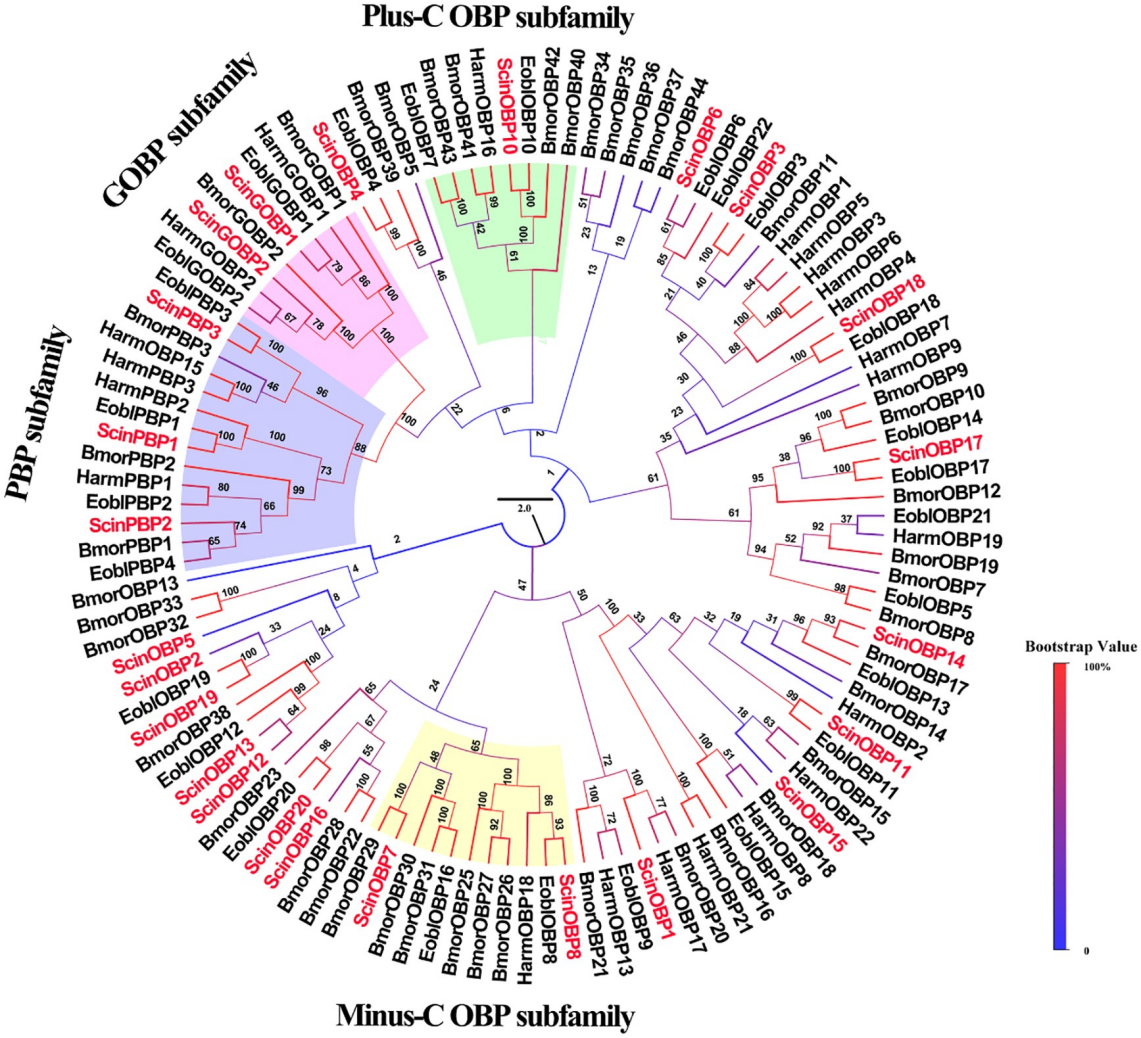
Phylogenetic tree of OBPs in *Semiothisa cinerearia*, *Ectropis obliqua*, *Bombyx mori* and *Helicoverpa armigera*. The sequences used in this analysis are listed in S4 Table.

**Fig 2:**
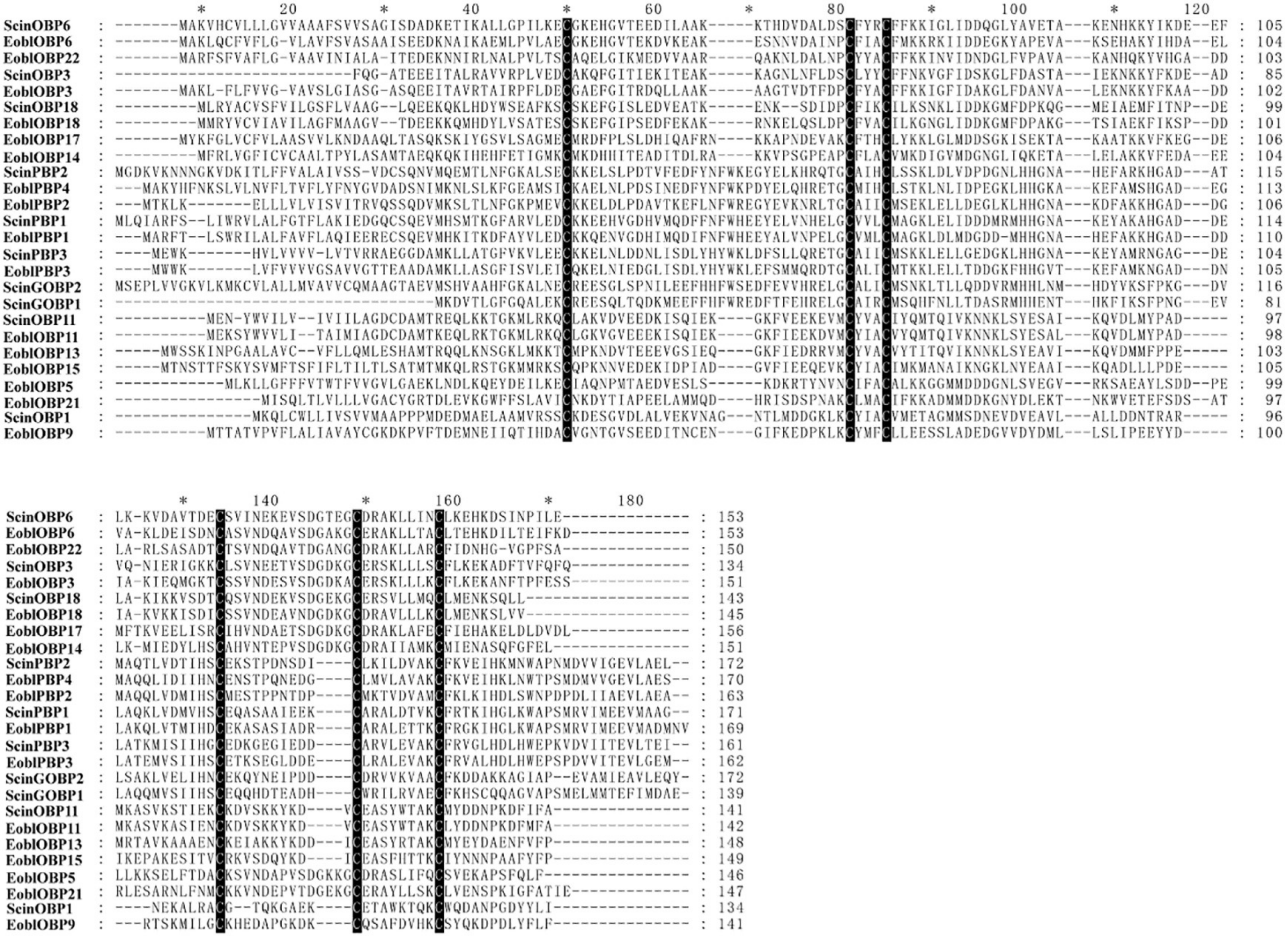
Amino acid sequence alignment of classic-OBPs in *Semiothisa cinerearia* and *Ectropis obliqua*.

According to the number of cysteine residues, OBPs can be divided into three subclasses: Classic OBPs (six cysteine residues at conserved positions), Plus-C OBPs (4–6 additional cysteines) and Minus-C OBPs (loss of cysteine residues, generally C2 and C5) [8, 12]. A total of 115 OBPs from moths were divided into several different branches in the reconstructed phylogenetic trees: the PBP subfamily, GOBP subfamily, Plus-C OBP subfamily and Minus-C OBP subfamily (Fig 1). The neighbor-joining tree showed that ScinOBP10, which was clustered with EoblOBP10 with a high bootstrap value, belonged to the Plus-C OBP subfamily. Additionally, ScinOBP8, which clustered with EoblOBP8, was in the Minus-C OBP subfamily (Fig 1). Notably, ScinPBP1 was clustered together with EbolPBP1, the geometrid PBP for binding Type-II sex pheromones [48]. The results of amino acid sequence alignment showed that 10 OBPs (ScinOBP1, ScinOBP3, ScinOBP6, ScinOBP11, ScinOBP18, ScinPBP1-3, and ScinGOBP1-2) exhibited a Cys spacing profile of “C1-X_25-30_-C2-X_3_-C3-X_37-42_-C4-X_8-14_-C5-X_8_-C6”, which is typical of Classic OBPs. These OBPs were highly conserved with the EoblOBPs of *E*. *obliqua*, a closely related geometrid moth (Fig 2).

**Fig 3:**
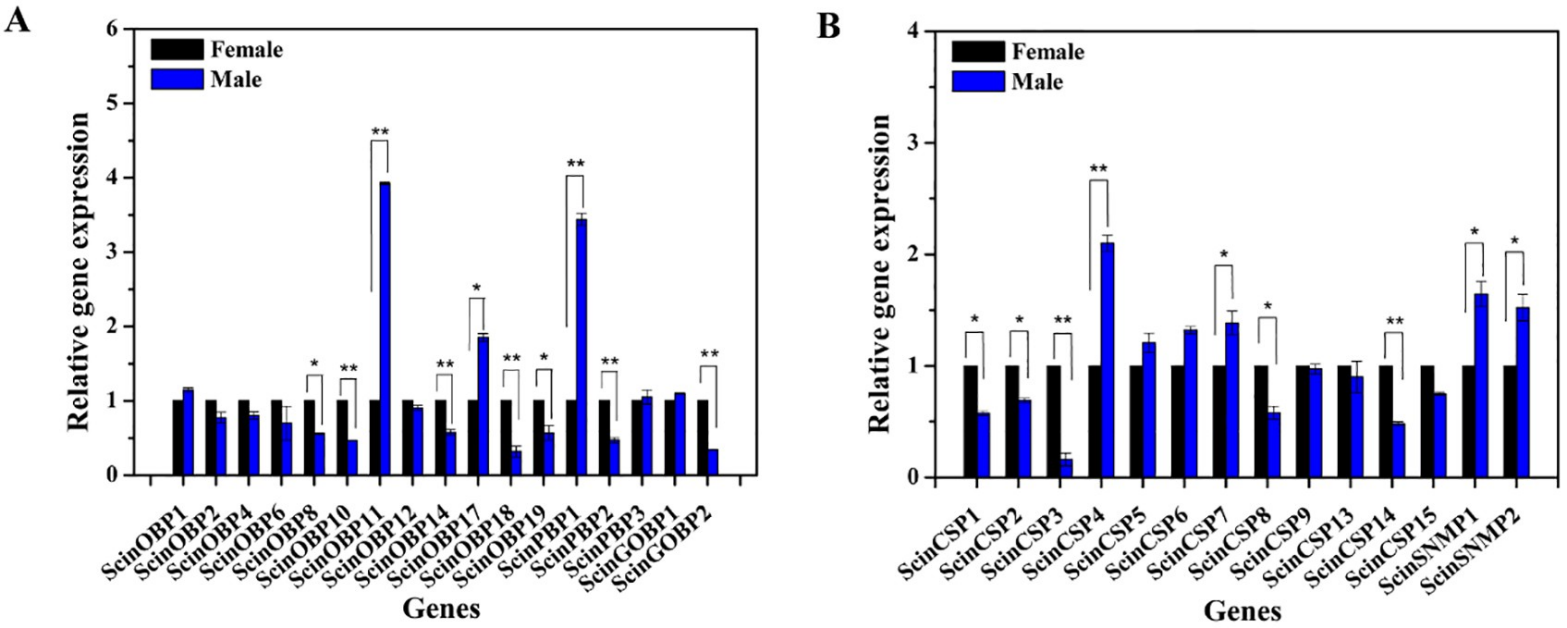
Expression of *Semiothisa cinerearia* OBPs (A), CSPs and SNMPs (B) by qRT-PCR. * indicates significant difference between female and male at p < 0.05, and ** indicates significant difference at p < 0.01.

Based on the FPKM values, ScinOBP6, ScinOBP12, ScinPBP1, ScinGOBP1 and ScinGOBP2 were highly expressed in the antennae of both female and male *S*. *cinerearia*. Further qRT-PCR tests using 17 OBPs with complete sequences showed that ScinOBP8, ScinOBP10, ScinOBP14, ScinOBP18, ScinOBP19, ScinPBP2 and ScinGOBP2 were significantly expressed in female antennae, whereas ScinOBP11, ScinOBP17 and ScinPBP1 were male antenna-biased (Fig 3A). Additionally, the correlation between RNA-seq and qRT-PCR data (R^2^ = 0.9207) confirmed the accuracy of the results (S5 Fig).

### Candidate CSPs and SNMPs in antennae of *S*. *cinerearia*

Fifteen transcripts were annotated as CSPs from the antennal transcriptome of *S*. *cinerearia*. All identified ScinCSPs had sequence lengths ranging from 71 to 303 aa and contained at least one signal peptide. There were 13 ScinCSPs with complete sequence, exceptions were ScinCSP5 and ScinCSP15. Based on BlastX, the majority of ScinCSPs were most similar to OBPs from *E*. *obliqua* with most exhibiting more than 60% identity (Table 2). In addition, ScinCSP2 was highly expressed and matched HarmCSP5 with an identity of 55.12%, which was reported to interact with 60 odorants (Table 2) [49].

**Table 2:** Summary of CSPs in *Semiothisa cinerearia*.

Gene name	Accession number	Length	Full- length	BlsatX annotation (Reference/Name/Species)	Score	E-value	Identity (%)	Signal Peptide	FPKM	
									Female	Male
ScinCSP1	MT380357	126	Yes	ALS03827.1|chemosensory protein 1 [*Ectropis obliqua*]	166	1.00E-50	63.71	Yes	522.22	300.05
ScinCSP2	MT380358	132	Yes	AEB54579.1|CSP5 [*Helicoverpa armigera*]	154	9.00E-46	55.12	Yes	1033.45	714.57
ScinCSP3	MT380359	124	Yes	AND82450.1|chemosensory protein 8 [*Athetis dissimilis*]	191	2.00E-60	72.27	Yes	64.41	10.41
ScinCSP4	MT380360	130	Yes	ALS03829.1|chemosensory protein 4 [*Ectropis obliqua*]	216	8.00E-70	79.84	Yes	901.55	1893.26
ScinCSP5	MT380361	71	No	ARO70316.1|Chemosensory protein 12 [*Dendrolimus punctatus*]	83.2	1.00E-18	68.97	No	22897.52	27669.39
ScinCSP6	MT380362	121	Yes	ALS03831.1|chemosensory protein 6 [*Ectropis obliqua*]	147	2.00E-43	61.34	Yes	461.01	609.08
ScinCSP7	MT380363	130	Yes	ANA75025.1|putative chemosensory protein 7 [*Ectropis obliqua*]	168	3.00E-51	67.72	Yes	8797.3	12187.53
ScinCSP8	MT380364	129	Yes	ALS03833.1|chemosensory protein 8 [*Ectropis obliqua*]	197	5.00E-63	70.16	Yes	48.23	27.98
ScinCSP9	MT380365	122	Yes	ALS03835.1|chemosensory protein 10 [*Ectropis obliqua*]	218	3.00E-71	83.47	Yes	14.66	14.26
ScinCSP10	MT380366	123	Yes	ALS03837.1|chemosensory protein 12 [*Ectropis obliqua*]	235	3.00E-78	92.56	Yes	0.76	2.94
ScinCSP11	MT380367	116	Yes	ALS03839.1|chemosensory protein 14 [*Ectropis obliqua*]	192	2.00E-61	83.48	Yes	0.57	1.76
ScinCSP12	MT380368	125	Yes	ALS03841.1|chemosensory protein 16 [*Ectropis obliqua*]	186	2.00E-58	67.48	Yes	2.68	2.05
ScinCSP13	MT380369	128	Yes	ALS03842.1|chemosensory protein 17 [*Ectropis obliqua*]	228	7.00E-74	82.68	Yes	739.04	665.8
ScinCSP14	MT380370	303	Yes	ALS03844.1|chemosensory protein 19 [*Ectropis obliqua*]	397	2.00E-136	73.68	Yes	81.59	39.19
ScinCSP15	MT380371	91	No	ALS03846.1|chemosensory protein 21 [*Ectropis obliqua*]	187	4.00E-58	55.21	Yes	46.28	34.66

**Fig 4:**
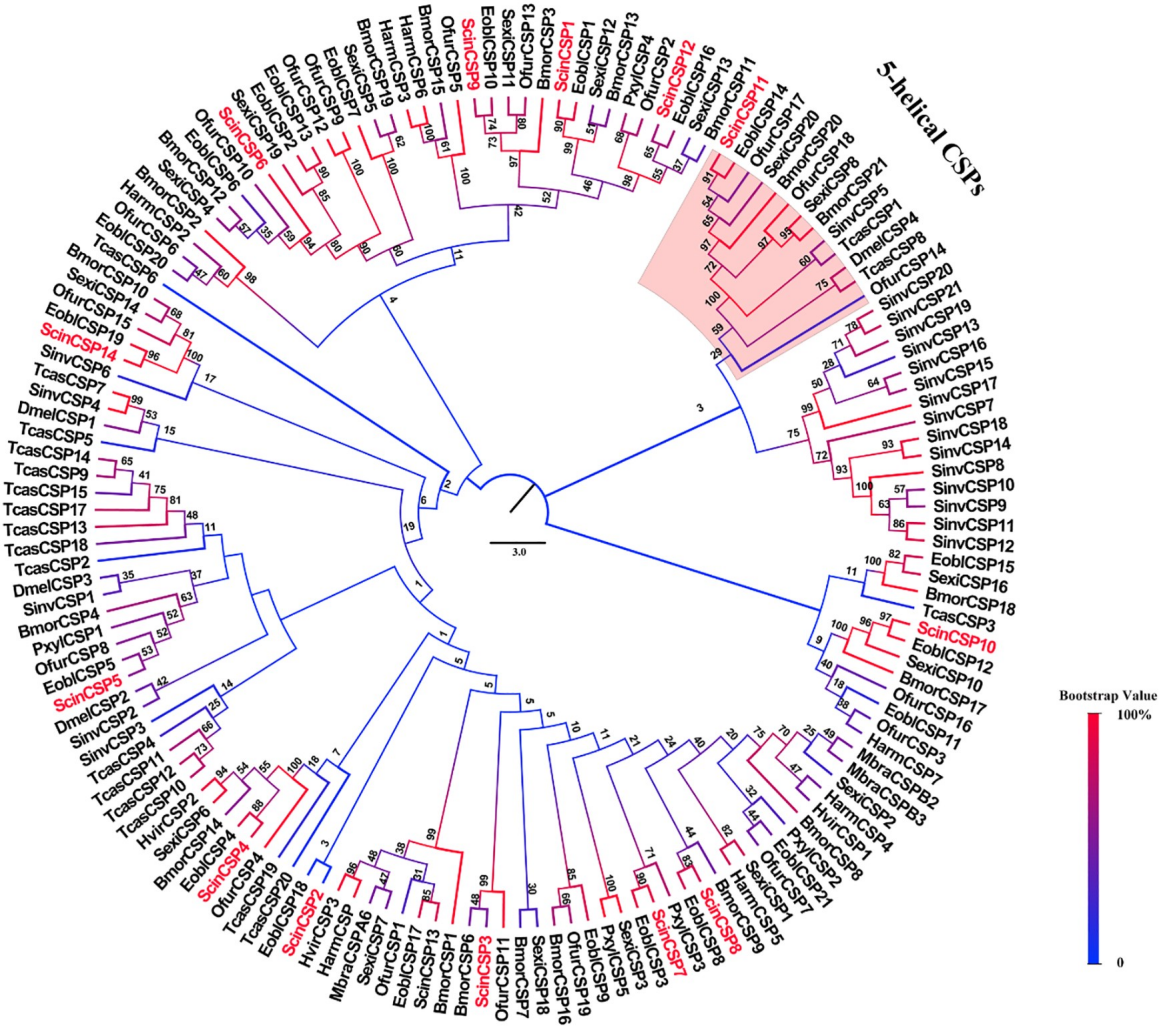
Phylogenetic tree of CSP proteins. Sequences used were from Hymenoptera (*Solenopsis invicta*), Coleoptera (*Tribolium castaneum*), Diptera (*Drosophila melanogaster*), and Lepidoptera (*Bombyx mori*, *Helicoverpa armigera*, *Heliothis virescens*, *Mamestra brassicae*, *Manduca sexta*, *Plutella xylostella*, *Ectropis obliqua*, *Spodoptera exigua*, *Ostrinia furnacalis*). The sequences listed in S5 Table.

The phylogenetic analysis of 153 sequences showed that ScinCSPs were distributed to different branches and had closest evolutionary relationships with corresponding proteins in *E*. *obliqua* (Fig 4). ScinCSP11 clustered 5-helix CSP subfamily. Secondary structure prediction result showed that ScinCSP11 lost the last helix (S6 Fig). All ScinCSPs had a relatively conserved structure, and ScinCSP1-14 conformed to a Cys spacing profile of “C1-X_6_-C2-X_18_-C3-X_2_-C4” (S7 Fig).

The FPKM analysis showed that ScinCSP5 displayed the highest expression levels among all CSPs, followed by ScinCSP7 (Table 2). Furthermore, the relative expression of 12 ScinCSPs (ScinCSP1-9 and ScinCSP13-15) in the qRT-PCR showed that ScinCSP3 and ScinCSP14 were significantly more expressed in the female antennae, whereas ScinCSP4 and ScinCSP7 had male antenna-biased expression (Fig 3B).

**Fig 5:**
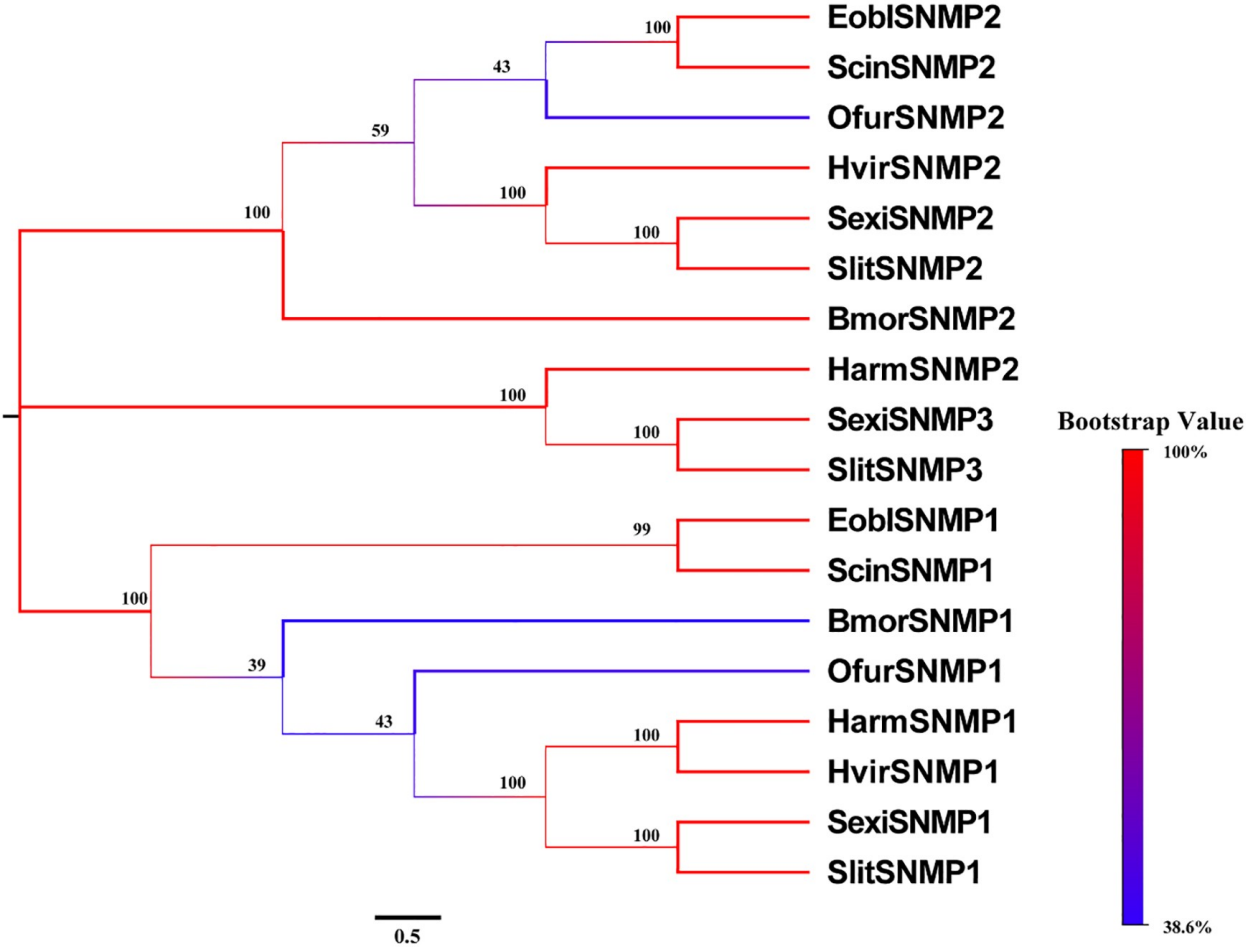
Phylogenetic tree of SNMP proteins in *Semiothisa cinerearia*, *Ectropis obliqua*, *Spodoptera exigua*, *Spodoptera litura*, *Bombyx mori*, *Ostrinia furnacalis*, *Helicoverpa armigera* and *Heliothis virescens*. The sequences used in this analysis are listed in S6 Table.

We also identified two full-length transcripts encoding putative SNMPs. ScinSNMP1 and ScinSNMP2 showed 73.86% and 80.39% identity with their orthologs in *E*. *obliqua* (Table 3). Sequence alignment results showed that SNMPs had five conserved cysteine residues and were highly conserved among insects (S8 Fig). The evolutionary tree showed that ScinSNMP1 and ScinSNMP2 were clustered with EbolSNMP1 and EbolSNMP2, respectively (Fig 5). The qRT-PCR results showed that both ScinSNMP1 and ScinSNMP2 were highly expressed in male antennae (Fig 3B).

**Table 3:** Summary of SNMPs and ORs in *Semiothisa cinerearia*.

Gene name	Accession number	Length	Full-length	BlsatX annotation (Reference/Name/Species)	Score	E-value	Identity (%)	Number of TMDs	FPKM	
									Female	Male
ScinSNMP1	MT380372	484	Yes	AKN78948.1|sensory neuron membrane protein 1 [*Ectropis obliqua*]	762	0.00E+00	73.86	2	7.98	13.13
ScinSNMP2	MT380373	516	Yes	ANA75033.1|putative sensory neuron membrane protein 2 [*Ectropis obliqua*]	907	0.00E+00	80.39	2	108.37	165.09
ScinOrco	MT380374	454	Yes	AKW50880.1|odorant receptor coreceptor [*Ectropis obliqua*]	863	0	89.18	7	241.08	126.08
ScinOR2	MT380375	449	Yes	AIG51888.1|odorant receptor [*Helicoverpa armigera*]	681	0	73.53	6	12.03	9.91
ScinOR3	MT380376	442	No	XP_026322996.1|odorant receptor 85c-like [*Hyposmocoma kahamanoa*]	536	0	55.23	4	4.84	1.81
ScinOR4	MT380377	430	Yes	XP_021200762.1|odorant receptor 13a-like [*Helicoverpa armigera*]	466	1.00E-159	53.05	6	8.79	3.95
ScinOR5	MT380378	428	Yes	AXF48810.1|odorant receptors OR74 [*Lobesia botrana*]	379	2.00E-125	45.74	6	0.51	3.15
ScinOR6	MT380379	421	Yes	AFC91713.2|odorant receptor 3 [*Cydia pomonella*]	339	6.00E-110	40.77	4	2.89	14.23
ScinOR7	MT380380	420	Yes	AII01072.1|odorant receptor [*Dendrolimus houi*]	569	0	63.72	6	4.53	2.26
ScinOR8	MT380381	417	Yes	AII01072.1|odorant receptor [*Dendrolimus houi*]	461	5.00E-158	53.00	6	3.06	5.88
ScinOR9	MT380382	410	No	AGK90001.1| olfactory receptor 7 [*Helicoverpa armigera*]	529	0.00E+00	63.61	6	4.78	5.11
ScinOR10	MT380383	408	Yes	AFL70814.1|odorant receptor 51 [*Manduca sexta*]	279	5.00E-87	34.88	6	3.39	2.05
ScinOR11	MT380384	408	Yes	CUQ99400.1|Olfactory receptor 17 [*Manduca sexta*]	461	2.00E-158	54.50	6	6.27	2.99
ScinOR12	MT380385	407	Yes	AII01104.1|odorant receptor [*Dendrolimus kikuchii*]	454	9.00E-156	56.40	7	2.74	0.35
ScinOR13	MT380386	405	Yes	XP_028028939.1|odorant receptor 4-like [*Bombyx mandarina*]	485	6.00E-168	57.53	6	2.9	2.3
ScinOR14	MT380387	405	Yes	ARO70503.1|Odorant Receptor 14–2 [*Dendrolimus punctatus*]	430	5.00E-146	54.70	6	5.85	4.38
ScinOR15	MT380388	405	Yes	XP_021196261.1|odorant receptor Or1-like [*Helicoverpa armigera*]	549	0	66.00	4	6.98	3.15
ScinOR16	MT380389	403	Yes	XP_021199795.1|odorant receptor 94b-like [*Helicoverpa armigera*]	486	2.00E-168	63.75	4	11.91	11.58
ScinOR17	MT380390	402	Yes	ARO70522.1|Odorant Receptor 59–2 [*Dendrolimus punctatus*]	373	8.00E-124	46.33	5	3.94	1.51
ScinOR18	MT380391	401	Yes	ALS03874.1|odorant receptor 3 [*Ectropis obliqua*]	695	0	85.43	6	6.07	3.75
ScinOR19	MT380392	397	Yes	XP_021194495.1|odorant receptor 2a-like isoform X1 [*Helicoverpa armigera*]	437	2.00E-149	54.80	7	1.19	1.74
ScinOR20	MT380393	396	Yes	CUQ99414.1|Olfactory receptor 34 [*Manduca sexta*]	436	6.00E-149	55.92	8	3.94	1.51
ScinOR21	MT380394	396	Yes	ALM26217.1|odorant receptor 28 [*Athetis dissimilis*]	353	7.00E-116	42.60	6	3.32	0.78
ScinOR22	MT380395	396	No	XP_030033340.1|odorant receptor Or2-like [*Manduca sexta*]	617	0	74.49	6	8.16	4.53
ScinOR23	MT380396	394	Yes	ARO70500.1|Odorant Receptor 1–2 [*Dendrolimus punctatus*]	297	4.00E-94	38.54	6	15.89	9.62
ScinOR24	MT380397	393	Yes	AII01084.1|odorant receptor [*Dendrolimus kikuchii*]	560	0	69.23	7	12.96	11.85
ScinOR25	MT380398	383	Yes	AII01058.1|odorant receptor [*Dendrolimus houi*]	473	8.00E-164	60.95	7	8.74	7.27
ScinOR26	MT380399	364	No	XP_023941745.1|odorant receptor 4-like [*Bicyclus anynana*]	419	9.00E-143	52.21	6	8.22	3.65
ScinOR27	MT380400	324	Yes	XP_022816228.1|odorant receptor 4-like [*Spodoptera litura*]	281	4.00E-90	43.93	4	1.92	1.08
ScinOR28	MT380401	322	No	XP_022815660.1|odorant receptor 4-like [*Spodoptera litura*]	405	2.00E-137	58.38	3	2.84	1.3
ScinOR29	MT380402	321	No	AFL70814.1|odorant receptor 51 [*Manduca sexta*]	231	1.00E-69	37.22	4	4.1	3.08
ScinOR30	MT380403	307	No	QEY02574.1|odorant receptor 5 [*Spodoptera littoralis*]	282	2.00E-89	42.30	1	1.73	8.89
ScinOR31	MT380404	296	No	CUQ99408.1|Olfactory receptor 26 [*Manduca sexta*]	296	2.00E-95	54.04	4	7.03	7.51
ScinOR32	MT380405	295	No	KOB67926.1|Odorant receptor [*Operophtera brumata*]	501	6.00E-114	57.04	5	2.64	1.12
ScinOR33	MT380406	292	No	AFL70814.1|odorant receptor 51 [*Manduca sexta*]	227	3.00E-68	40.00	4	6.23	4.63
ScinOR34	MT380407	283	No	XP_012545300.1|odorant receptor 94b [*Bombyx mori*]	350	2.00E-117	61.59	4	4.72	3.25
ScinOR35	MT380408	275	No	ALM26250.1|odorant receptor 85 [*Athetis dissimilis*]	248	9.00E-77	48.01	4	0.36	0.97
ScinOR36	MT380409	274	No	CUQ99415.1|Olfactory receptor 35 [*Manduca sexta*]	399	2.00E-136	67.90	4	7.76	6.12
ScinOR37	MT380410	267	No	ALS03872.1|odorant receptor 1 [*Ectropis obliqua*]	269	1.00E-84	53.23	3	0.53	3
ScinOR38	MT380411	249	No	AQQ73507.1|olfactory receptor 27 [*Heliconius melpomene rosina*]	367	9.00E-124	67.87	4	14.54	6.76
ScinOR39	MT380412	241	No	AVF19668.1|putative odorant receptor [*Peridroma saucia*]	280	4.00E-90	54.17	1	1.72	1.31
ScinOR40	MT380413	226	No	ARO70217.1|Odorant Receptor 5 [*Dendrolimus punctatus*]	392	1.00E-133	82.96	3	1.16	2.9
ScinOR41	MT380414	212	No	ALM26219.1|odorant receptor 30 [*Athetis dissimilis*]	246	4.00E-77	51.43	2	10.91	6.66
ScinOR42	MT380415	208	No	XP_022829617.1|odorant receptor 4-like [*Spodoptera litura*]	207	9.00E-62	47.09	3	1.73	2.77
ScinOR43	MT380416	204	No	AIG51872.1|odorant receptor [*Helicoverpa armigera*]	326	2.00E-109	74.75	3	1.67	1.55
ScinOR44	MT380417	161	No	KOB52347.1|Odorant receptor 50 [*Operophtera brumata*]	207	9.00E-63	58.86	2	2.78	3.73
ScinOR45	MT380418	146	No	AOG12912.1|odorant receptor [*Eogystia hippophaecolus*]	167	3.00E-47	56.43	2	3.08	1.02
ScinOR46	MT380419	142	No	XP_026734904.1|odorant receptor Or2-like [*Trichoplusia ni*]	186	4.00E-55	62.68	2	18.02	6.55
ScinOR47	MT380420	140	No	CUQ99414.1|Olfactory receptor 34 [*Manduca sexta*]	154	1.00E-42	59.84	0	2.55	1.62
ScinOR48	MT380421	139	No	ALM26250.1|odorant receptor 85 [*Athetis dissimilis*]	199	4.00E-60	64.49	2	6.48	6.02
ScinOR49	MT380422	138	No	ALM26215.1|odorant receptor 26 [*Athetis dissimilis*]	224	1.00E-70	78.10	2	0.4	0.86
ScinOR50	MT380423	135	No	CUQ99415.1|Olfactory receptor 35 [*Manduca sexta*]	156	2.00E-43	56.00	1	2.09	2.32
ScinOR51	MT380424	131	No	CUQ99408.1|Olfactory receptor 26 [*Manduca sexta*]	84	3.00E-16	40.82	2	5.55	1.87
ScinOR52	MT380425	126	No	XP_026737268.1|odorant receptor 85c-like [*Trichoplusia ni*]	136	8.00E-36	58.20	1	2.13	0.93

### Candidate chemoreceptors in *S*. *cinerearia*

**Fig 6:**
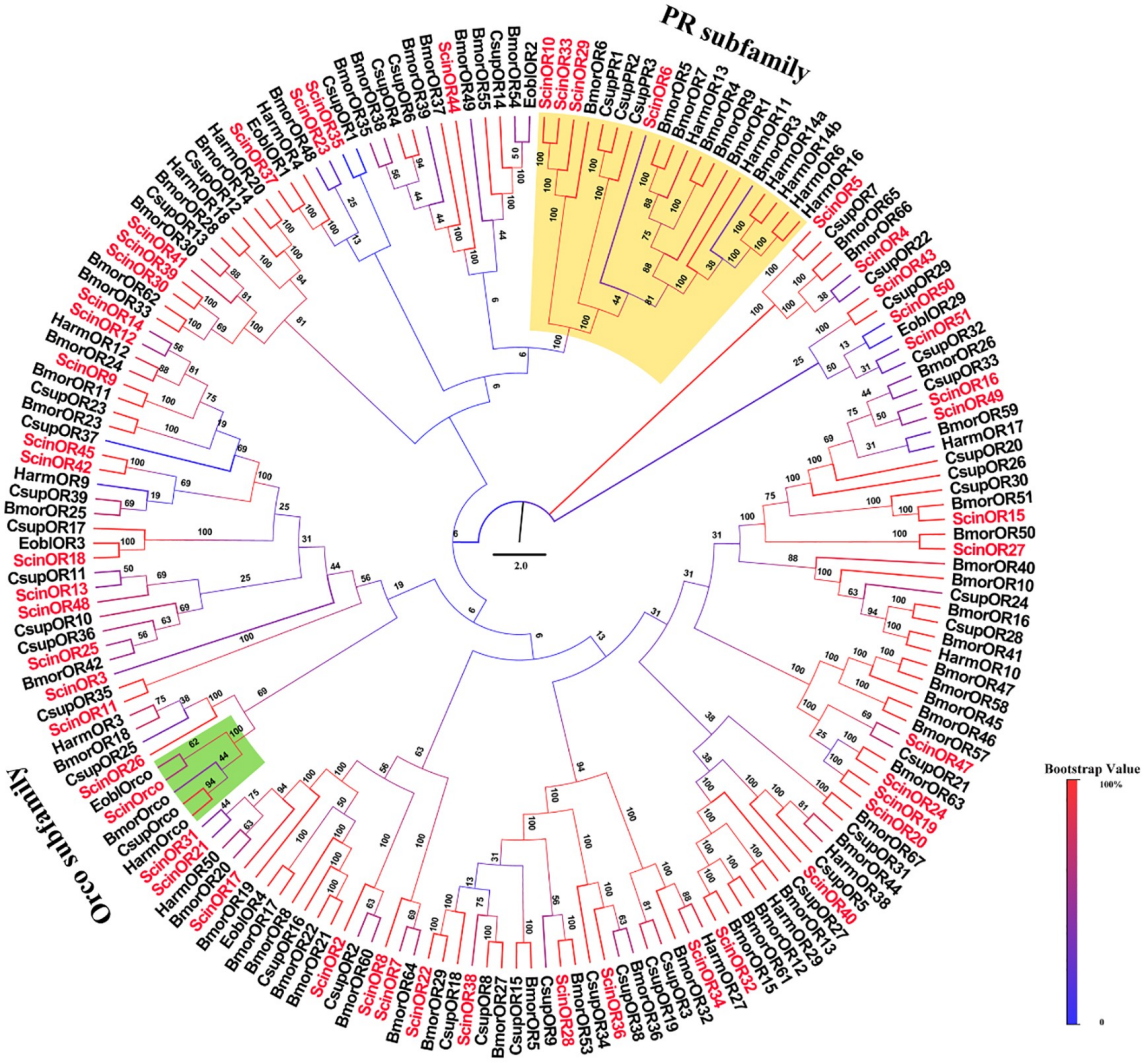
Phylogenetic tree of OR proteins in *Semiothisa cinerearia*, *Ectropis obliqua*, *Chilo suppressalis*, *Bombyx mori* and *Helicoverpa armigera*. The sequences used in this analysis are listed in S7 Table.

In this study, we identified 52 candidate ORs from the antennal transcriptome of *S*. *cinerearia*. Among them, 23 ORs were full-length ORs with 4–7 transmembrane domains. The incomplete ORs also contained 1–6 transmembrane domains (Table 3). ScinOrco had high identity (89.18%) with EoblOrco. The neighbor-joining tree reconstructed from 181 ORs from *S*. *cinerearia*, *E*. *obliqua*, *B*. *mori*, *H*. *armigera* and *Chilo*. *suppressalis* revealed that four ORs (ScinOR6, ScinOR10, ScinOR29 and ScinOR33) were clustered into the traditional pheromone receptor (PR) subfamily, that is thought to recognize Type-I pheromones. As expected, ScinOrco belonged to the Orco subfamily, with high homology to EoblOrco (Fig 6). The FPKM analysis revealed that ScinOrco had the highest FPKM value. qRT-PCR expression showed that 12 ORs (ScinOrco, ScinOR3-4, ScinOR7, ScinOR11-12, ScinOR15, ScinOR17, ScinOR20-21 and ScinOR 26–27) were highly expressed in female antennae. Only three ORs (ScinOR5, ScinOR6 and ScinOR8) were significantly more expressed in males (Fig 9A).

**Fig 7:**
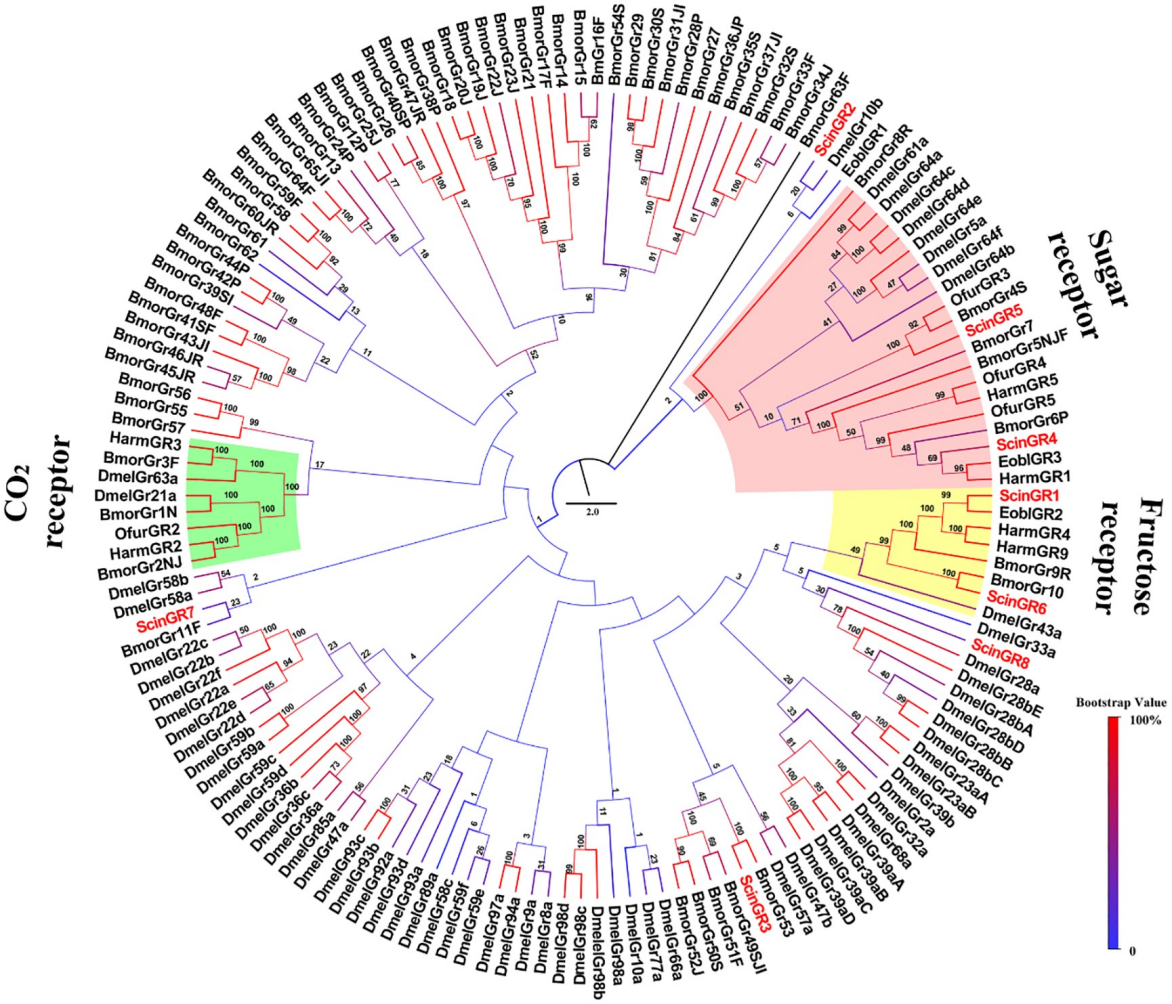
Phylogenetic tree of GR proteins in *Semiothisa cinerearia*, *Ectropis obliqua*, *Bombyx mori*, *Ostrinia furnacalis*, *Helicoverpa armigera* and *Drosophila melanogaster*. The sequences used in this analysis are listed in S8 Table.

Eight putative GRs were obtained by bioinformatic analysis. Although only one GR was intact (ScinGR1), all ScinGRs had 1–6 transmembrane domains (Table 4). The phylogenetic analysis showed that ScinGR1 and ScinGR6 were clustered with fructose receptors EoblGR2 and BmorGr10. ScinGR4 and ScinGR5 were members of the “sugar” receptor subfamily (Fig 7). qRT-PCR results showed that two GRs (ScinGR4 and ScinGR7) were highly expressed in female antennae, and three (ScinGR3, ScinGR6 and ScinGR8) exhibited high expression in males (Fig 9B).

**Table 4:** Summary of GRs in *Semiothisa cinerearia*.

Gene name	Accession number	Length	Full-length	BlsatX annotation (Reference/Name/Species)	Score	E-value	Identity (%)	Number of TMDs	FPKM	
									Female	Male
ScinGR1	MT380426	478	Yes	ALS03937.1|gustatory receptor 2 [*Ectropis obliqua*]	714	0	80.97	6	7.9	4.65
ScinGR2	MT380427	216	No	XP_026736704.1|probable glutamate receptor [*Trichoplusia ni*]	225	6.00E-65	59.04	1	1.56	1.63
ScinGR3	MT380428	215	No	KOB74473.1|Gustatory receptor 53 [*Operophtera brumata*]	256	6.00E-81	63.40	3	1.24	3.81
ScinGR4	MT380429	177	No	ALS03938.1|gustatory receptor 3 [*Ectropis obliqua*]	301	1.00E-98	91.77	3	1.81	0.64
ScinGR5	MT380430	164	No	XP_021182859.1|gustatory receptor for sugar taste 64a-like [*Helicoverpa armigera*]	231	9.00E-72	68.55	2	4.63	4.11
ScinGR6	MT380431	125	No	BAS18817.1|gustatory receptor 10 [*Bombyx mori*]	125	1.00E-31	51.61	1	2.18	4.67
ScinGR7	MT380432	119	No	ASW18693.1|gustatory receptor 4 [*Helicoverpa armigera*]	117	1.00E-28	49.58	3	6.58	0.55
ScinGR8	MT380433	108	No	KOB58122.1Gustatory receptor 67 [*Operophtera brumata*]	113	3.00E-28	59.55	1	1.04	2.37

**Fig 8:**
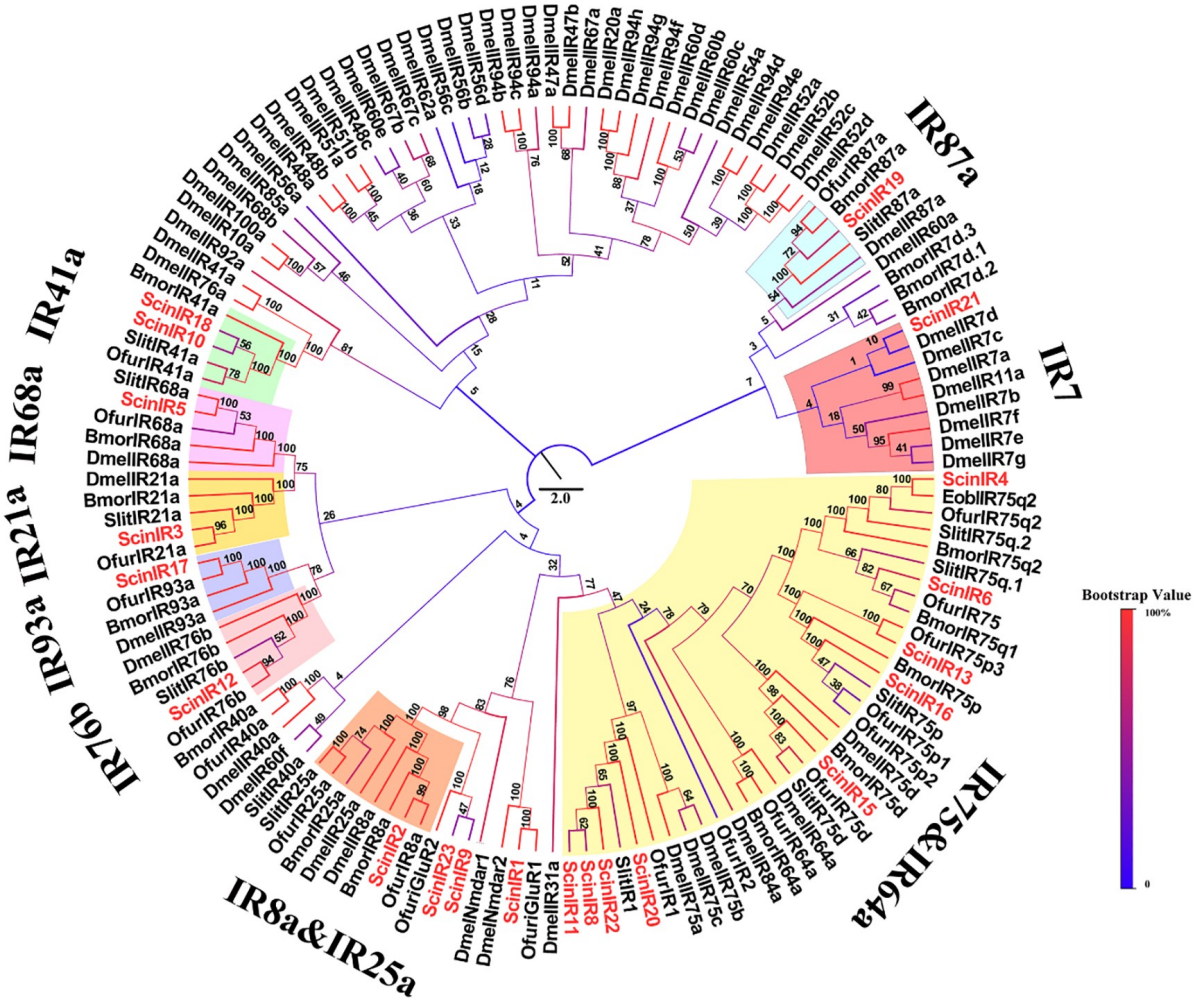
Phylogenetic tree of IR proteins in *Semiothisa cinerearia*, *Ectropis obliqua*, *Spodoptera litura*, *Ostrinia furnacalis*, *Bombyx mori* and *Drosophila melanogaster*. The sequences used in this analysis are listed in S9 Table.

**Fig 9:**
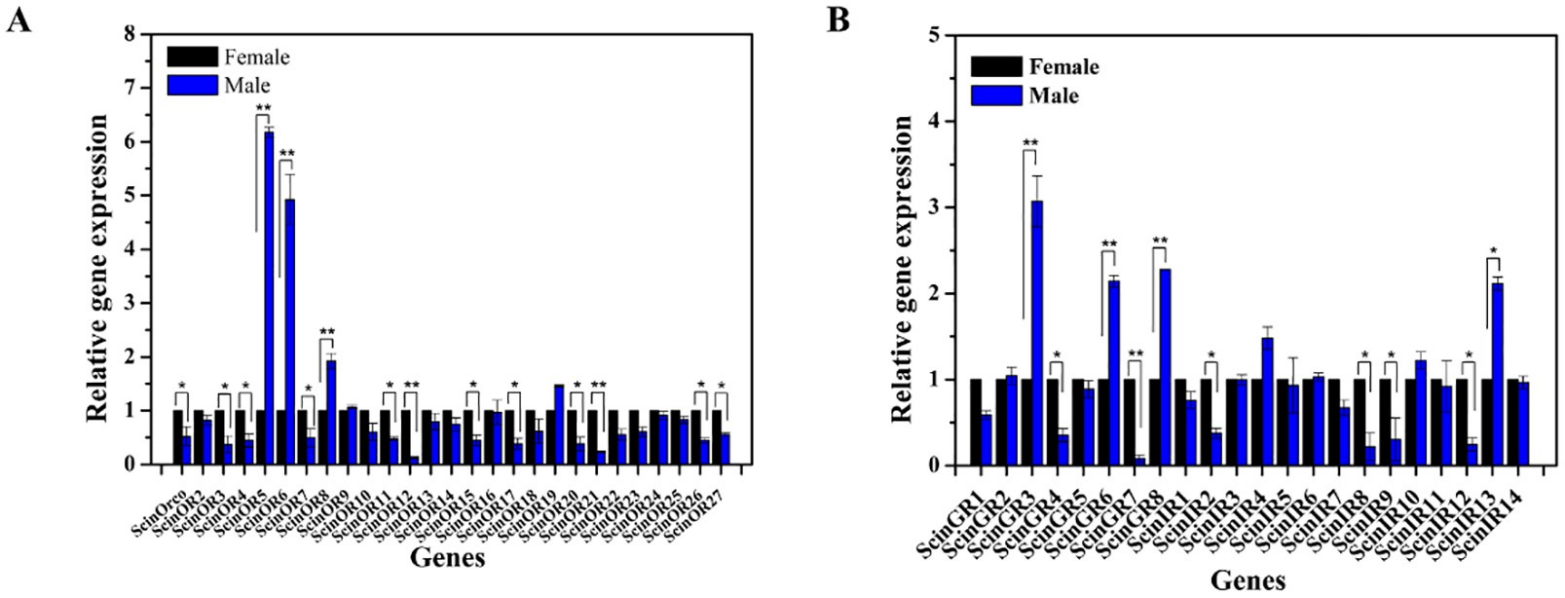
Expression of *Semiothisa cinerearia* ORs (A), GRs and IRs (B) by qRT-PCR. * indicates significant difference between female and male at p <0.05, and ** indicates significant difference at p < 0.01.

A total of 23 putative ScinIRs were also identified from transcriptomic analyses. Among them, seven candidate IRs (ScinIR2, ScinIR6, ScinIR8, ScinIR10, ScinIR11, ScinIR12 and ScinIR14) had full-length ORFs. The others were incomplete at the 5' or 3' end (Table 5). The evolutionary tree showed that five ScinIRs (ScinIR4, ScinIR6, ScinIR13, ScinIR15 and ScinIR16) were clustered with the IR75 subfamily and ScinIR17 was clustered with the IR93 subfamily. In addition, ScinIR10 was close to OfurIR41a, and ScinIR12 belonged to the IR76 subfamily (Fig 8). The qRT-PCR results revealed that four IRs (ScinIR2, ScinIR8, ScinIR9 and ScinIR12) were highly expressed in female antennae, and ScinIR13 had male-specific expression (Fig 9B).

**Table 5:** Summary of IRs in *Semiothisa cinerearia*.

Gene name	Accession number	Length	Full-length	BlsatX annotation (Reference/Name/Species)	Score	E-value	Identity (%)	Number of TMDs	FPKM	
									Female	Male
ScinIR1	MT380434	1015	No	AXF48844.1|ionotropic receptor IR13 [*Lobesia botrana*]	1791	0	90.99	5	12.89	9.81
ScinIR2	MT380435	881	Yes	QBX91201.1|ionotropic receptor 8a [*Helicoverpa armigera*]	1310	0	74.18	3	25.02	9.41
ScinIR3	MT380436	702	No	XP_030021838.1|ionotropic receptor 21a [*Manduca sexta*]	1144	0	78.57	4	21.59	21.53
ScinIR4	MT380437	689	No	AST36236.1| putative ionotropic receptor IR75q.2 [*Hedya nubiferana*]	845	0	69.80	3	2.18	3.23
ScinIR5	MT380438	676	No	AYD42277.1|ionotropic receptor 7 [*Carposina sasakii*]	1038	0	73.50	4	2.83	2.64
ScinIR6	MT380439	639	Yes	BAR64803.1|ionotropic receptor [*Ostrinia furnacalis*]	552	0	45.48	3	2.97	3.05
ScinIR7	MT380440	635	No	XP_030020446.1|ionotropic receptor 25a isoform X1 [*Manduca sexta*]	1241	0	92.76	1	12.22	8.21
ScinIR8	MT380441	629	Yes	QHB15311.1|ionotropic receptor 1.1 [*Peridroma saucia*]	747	0	58.60	0	5.64	1.25
ScinIR9	MT380442	627	No	AXF48869.1|ionotropic receptor IR38 [*Lobesia botrana*]	647	0	78.46	3	15.66	4.77
ScinIR10	MT380443	594	Yes	AOG12846.1|ionotropic receptor [*Eogystia hippophaecolus*]	747	0	59.00	3	7.23	8.84
ScinIR11	MT380444	550	Yes	XP_026739905.1|ionotropic receptor 75a-like [*Trichoplusia ni*]	653	0	58.12	2	1.1	1.01
ScinIR12	MT380445	545	Yes	ARO76467.1|ionotropic receptor 4 [*Conogethes punctiferalis*]	761	0	67.34	3	21.3	5.24
ScinIR13	MT380446	509	No	XP_028169063.1|ionotropic receptor 75a-like [*Ostrinia furnacalis*]	787	0	74.51	3	5.64	11.94
ScinIR14	MT380447	480	Yes	XP_021190111.1|ionotropic receptor 93a [*Helicoverpa armigera*]	771	0	75.57	1	5.8	5.6
ScinIR15	MT380448	464	No	ARO70544.1|antennal ionotropic receptor 75d-2 [*Dendrolimus punctatus*]	602	0	66.67	0	3.31	2.08
ScinIR16	MT380449	442	No	BAR64805.1|ionotropic receptor [*Ostrinia furnacalis*]	602	0	65.83	3	7.67	7.58
ScinIR17	MT380450	400	No	XP_021203225.1|ionotropic receptor 93a [*Bombyx mori*]	592	0	71.25	3	5.77	6.35
ScinIR18	MT380451	373	No	AOG12846.1|ionotropic receptor [*Eogystia hippophaecolus*]	494	4.00E-169	59.95	3	2.34	2
ScinIR19	MT380452	314	No	ARO76464.1|ionotropic receptor 1 [*Conogethes punctiferalis*]	554	0	83.71	4	5.02	2.8
ScinIR20	MT380453	187	No	AOE47994.1|putative ionotropic receptor IR1 [*Athetis lepigone*]	195	1.00E-59	63.38	1	11.16	12.2
ScinIR21	MT380454	156	No	AJD81613.1|ionotropic glutamate receptor 2 [*Helicoverpa assulta*]	203	2.00E-58	73.89	1	3.82	0.54
ScinIR22	MT380455	128	No	XP_026739905.1|ionotropic receptor 75a-like [Trichoplusia ni]	160	2.00E-43	62.18	1	15.53	5.93
ScinIR23	MT380456	127	No	AJD81618.1|ionotropic glutamate receptor 7 [*Helicoverpa assulta*]	186	4.00E-52	71.54	1	3.49	0.94

## Discussion

During the past decade, RNA-Seq-based transcriptome analysis has been widely used for screening the olfactory-related genes of insects [38, 50, 51]. In the present study, a total of 125 candidate olfactory-related genes were identified from the transcriptome of *S*. *cinerearia*, including 25 OBPs, 15 CSPs, 2 SNMPs, 52 ORs, 8 GRs and 23 IRs. Compared to that in the antennal transcriptome in Lepidoptera from *E*. *obliqua* (24 OBPs, 21 CSPs, 2 SNMPs, 4 ORs and 3 GRs) and *H*. *armigera* (26 OBPs, 21 CSPs, 2 SNMPs, 60 ORs, 19 IRs and 9 GRs), the number of olfactory-related genes in *S*. *cinerearia* was comparable [52, 53]. The number of olfactory transcripts in *S*. *cinerearia* was less than those in *Ectropis grisescens* (40 OBPs, 30 CSPs, 59 ORs, and 24 IRs) and *B*. *mori* (44 OBPs, 24 CSPs, 66 ORs, 17 IRs and 65 GRs) [53–55]. The difference among species might result from complex environmental changes or the diversity of gene functions. In addition, antennal cDNA libraries are notorious for under representation of transcripts, especially those with low expression [13, 56, 57].

Odorant binding is considered the first critical step in olfactory signal transduction pathways [58, 59]. Due to the various expression patterns of most OBPs, PBPs and GOBPs, their functions are more complex than previously thought [60–62]. For example, in *Drosophila melanogaster*, OBP49a is mainly expressed in the lip of taste organs and interacts with bitter chemicals [63]. In *E*. *obliqua*, EoblOBP6, which is highly abundant in the legs, can integrate with the benzaldehyde emitted from tea plants. EoblOBP6 also shows high binding abilities to nerolidol and α-farnese, herbivore-induced volatiles [14]. In *S*. *cinerearia*, ScinOBP6, abundantly expressed in antennae, was homologous to EoblOBP6 with >50% sequence identity (Fig 2; Table 1). Therefore, ScinOBP6 is predicted to bind similar volatiles, but this prediction requires further experimental verification. ScinGOBP2 might also bind volatiles from *S*. *japonica* leaves, similar to its homolog EoblGOBP2 (Fig 1), which has strong binding abilities with seven tea volatiles [64]. In addition, many insect PBPs are reported to bind pheromones [65]. ScinPBP1 and ScinPBP2 had high expression in antennae. In the phylogenetic tree, ScinPBP1 clustered together with EbolPBP1, the geometrid PBP for detecting Type-II sex pheromones [48, 66]. Combining the qRT-PCR results that ScinPBP1 was significantly expressed in male antennae, we speculate that ScinPBP1 likewise interacts with Type-II sex pheromones.

In insects, most CSPs are composed of 6 α-helices containing four highly-conserved cysteine residues over the protein structure [18]. However, some CSPs have only five α-helix domains [67]. The 5-helical CSP5 is likely involved in an essential ubiquitous function rather than chemosensation [68]. In our study, the modeled ScinCSP11 has completely lost helix 6, similar to the conserved 5-helical CSP5 (S6 Fig), indicating its potential multifunction. Insect SNMPs probably have similar functions in the binding and membrane translocation of fatty acids [69]. In the present study, two ScinSNMPs had typical features of the SNMP family, with two transmembrane domains and highly conserved sequences with other Lepidopteran insects (Fig 5; S8 Fig). Previous studies have shown that SNMP1 and SNMP2 are mostly abundant in the antennae, especially in males [21, 70]. The expression of the two ScinSNMPs was consistent with the above regularity (Fig 6), and the ScinSNMPs may have functions similar to those in other insects.

Olfactory receptors play crucial roles in odorant detection and recognition [71]. A total of 52 ORs, 8 GRs and 23 IRs were identified of *S*. *cinerearia*. For most insects, a coreceptor (Orco) is required for membrane targeting of canonical ORs on ORN membranes [71, 72]. ScinOrco showed the highest expression of all ScinORs, suggesting that it might have the potential to receive chemical signals [73]. In the present study, ScinOrco showed higher expression in female antennae (Fig 9A), which is opposite to the results of most species in Lepidoptera [74]. This difference may be attributable to sample size (i.e. three biological duplication) which potentially resulted more mistakes and consequently contributed to the significant differences. However, Orco showed higher expression in other moths, e.g. *Manduca sexta* and *E*. *grisescens* [55, 75], indicating the possibility that Orco is highly expressed in females. PRs, which function to detect sex pheromones, are reported to be male-specifically expressed in moths [76, 77]. In *S*. *cinerearia*, four ORs (ScinOR6, ScinOR10, ScinOR29 and ScinOR33) phylogenetically sorted with PR subfamily for Type-I sex pheromones components (Fig 6). In particular, ScinOR6 was highly expressed in male antennae (Figs 6 and 9A), suggesting that it might contribute to detecting sex pheromone. In Geometridae moths, including *E*. *grisescens* and *S*. *cinerearia*, Type-II pheromones dominate the chemical communication. The homology comparison with EgriORs related Type-II pheromone from *E*. *grisescens* revealed that ScinOR5 had high homology with EgriOR31 and EgriOR24, which function as Type-II pheromone receptors (unpublished data). Moreover, ScinOR5 was highly expressed in male antennae, further indicating that it may function as a PRs for Type-II sex pheromones [55]. The coexpression of PRs and PBPs could greatly enhance the sensitivity to pheromones [15]. Consequently, linking research on the function of ScinPBPs and ScinOR6 will be more interesting and meaningful.

GRs, mainly expressed in the gustatory organs, detect different sugars, bitter compounds, and contact pheromones [78]. Eight GRs were annotated from *S*. *cinerearia*, including two putative sugar receptors (ScinGR4 and ScinGR5). The expression of ScinGR4 and ScinGR5 was female-specific. Sugars and sugar alcohols are reported to affect host plant selection and egg-laying behavior of codling moth females [79]. However, most of the identified GRs were incomplete, and no CO_2_ receptor was found in *S*. *cinerearia*, which might be due to the low expression of GRs in antennae [24, 80]. Furthermore, we found that two coreceptors from IRs, ScinIR2 and ScinIR7, belong to the IR8a/IR25a subfamily, which are thought to act as coreceptors such as Orco [81, 82].

Transcriptome sequencing is ideal for obtaining target genes of interest [38]. GO analysis showed that the majority of ScinORs were mainly clustered in “signaling” and “molecular transducer activity”. Twenty ScinIRs (except ScinIR1, ScinIR8 and ScinIR20) matched with “molecular transducer activity”, “signaling” and “transporter activity”. Four ScinGRs (ScinGR1, ScinGR 2, ScinGR 4, and ScinGR 7) were enriched in “molecular transducer activity” (S3 Fig). This information confirmed the accuracy of gene identification from transcriptome data and increased the possibility of detecting odorant receptor function.

## Conclusion

In summary, our study provided the first report on antennal transcriptome analysis in *S*. *cinerearia*. A total of 65,476 unigenes were generated, and 30,805 unigenes were successfully annotated by the Nr database. A total of 125 unigenes were identified as olfactory-related genes, including 25 OBPs, 15 CSPs, two SNMPs, 52 ORs, 8 GRs and 23 IRs. Most olfactory-related genes showed female- or male-biased expression in the antennae. This will facilitate functional research on olfactory mechanisms in *S*. *cinerearia*.

## Supporting information

S1 Fig Distribution of length of *Semiothisa cinerearia*. (TIF)

S2 Fig Summary for the annotation of *Semiothisa cinerearia* antennal unigenes. (A) The number of unigenes matching in five databases. (B) The species distribution of the best Blastx hits in Nr database.
(TIF)

S3 Fig Gene Ontology (GO) functional classification of unigenes. The purple boxes indicate the process of accumulation of olfactory receptors.
(TIF)

S4 Fig KEGG pathway functional classification of unigenes. A: Cellular Processes. B: Environmental Information Processing. C: Genetic Information Processing. D: Metabolism. E: Organismal Systems.
(TIF)

S5 Fig The correlation analysis between the results of qRT-PCR and RNA-seq. The data was based on the average value of more than three repetitions, and students’ t-test was used to determine the statistical significance.
(TIF)

S6 Fig Secondary and 3D structures of ScinCSP11. (A) Sequence alignment of ScinCSP11 with CSP1 from *Bombyx mori* (template library identity: 2jnt.1A). α-helices are displayed as squiggles. The signal peptides are removed. (B) The predicted 3D structure of ScinCSP11.
(TIF)

S7 Fig Amino acid sequence alignment of CSPs in *Semiothisa cinerearia* and *Ectropis obliqua*. (TIF)

S8 Fig Amino acid sequence alignment of SNMPs in *Semiothisa cinerearia* and *Ectropis obliqua*. The red letters indicate conservative cysteine residues. The red box represents the transmembrane domains.
(TIF)

S1 Table Primer used in qRT-PCR. (DOCX)

S2 Table BUSCO assessment of *Semiothisa cinerearia*. (DOCX)

S3 Table Summary for the annotation of *Semiothisa cinerearia* unigenes. (DOCX)

S4 Table Amino acid sequences of OBPs used in phylogenetic analyses. (DOCX)

S5 Table Amino acid sequences of CSPs used in phylogenetic analyses. (DOCX)

S6 Table Amino acid sequences of SNMPs used in phylogenetic analyses. (DOCX)

S7 Table Amino acid sequences of ORs used in phylogenetic analyses. (DOCX)

S8 Table Amino acid sequences of GRs used in phylogenetic analyses. (DOCX)

S9 Table Amino acid sequences of IRs used in phylogenetic analyses. (DOCX)
