# Supplementary figures and images for: Identification of chemosensory genes from the antennal transcriptome of Semiothisa cinerearia
Source: PLoS One. 2020 Aug 7;15(8):e0237134. doi: 10.1371/journal.pone.0237134 (PMC7413487; doi:10.1371/journal.pone.0237134)

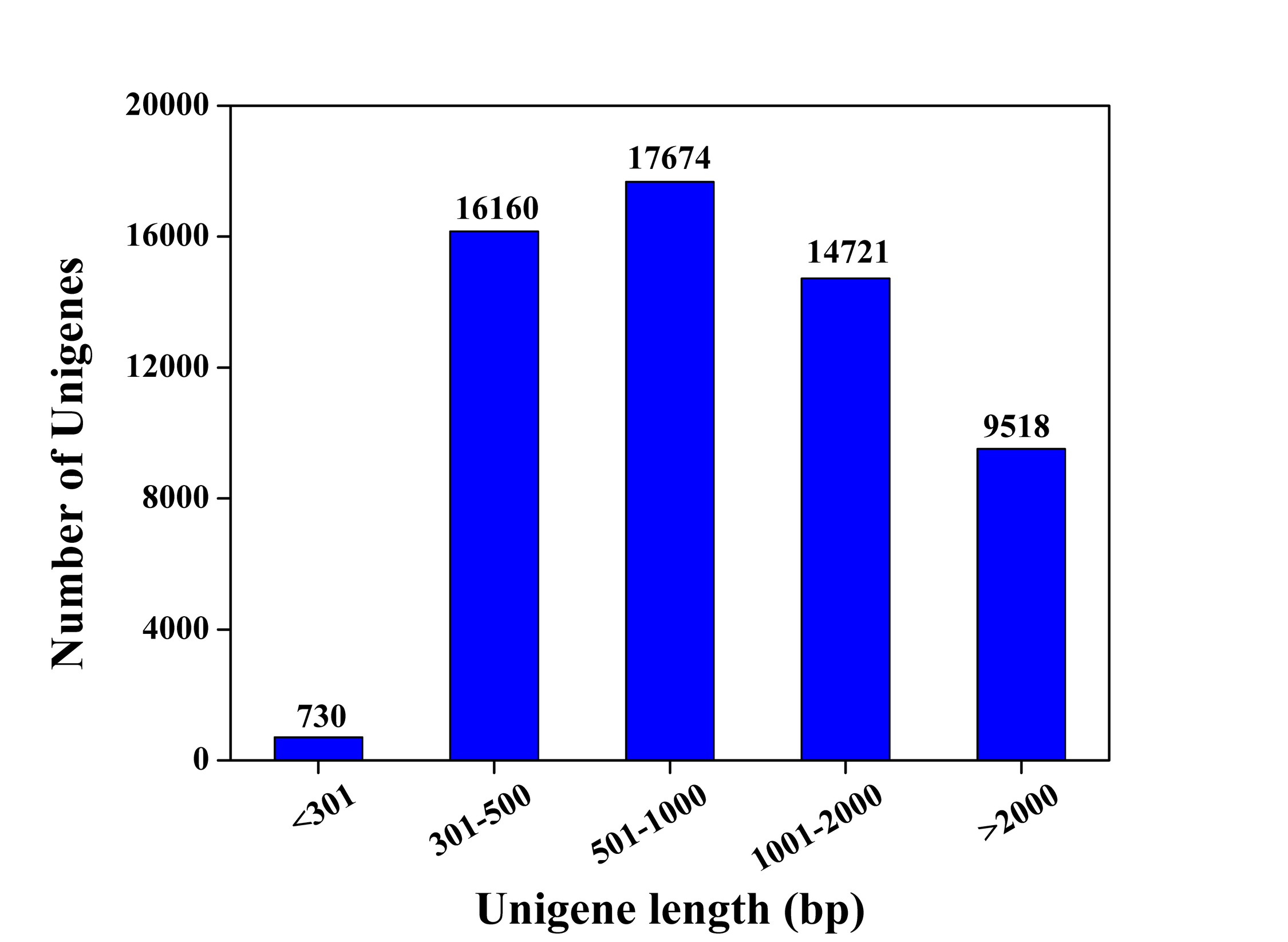

Supplement: S1 Fig — (TIF) [file pone.0237134.s001.tif]

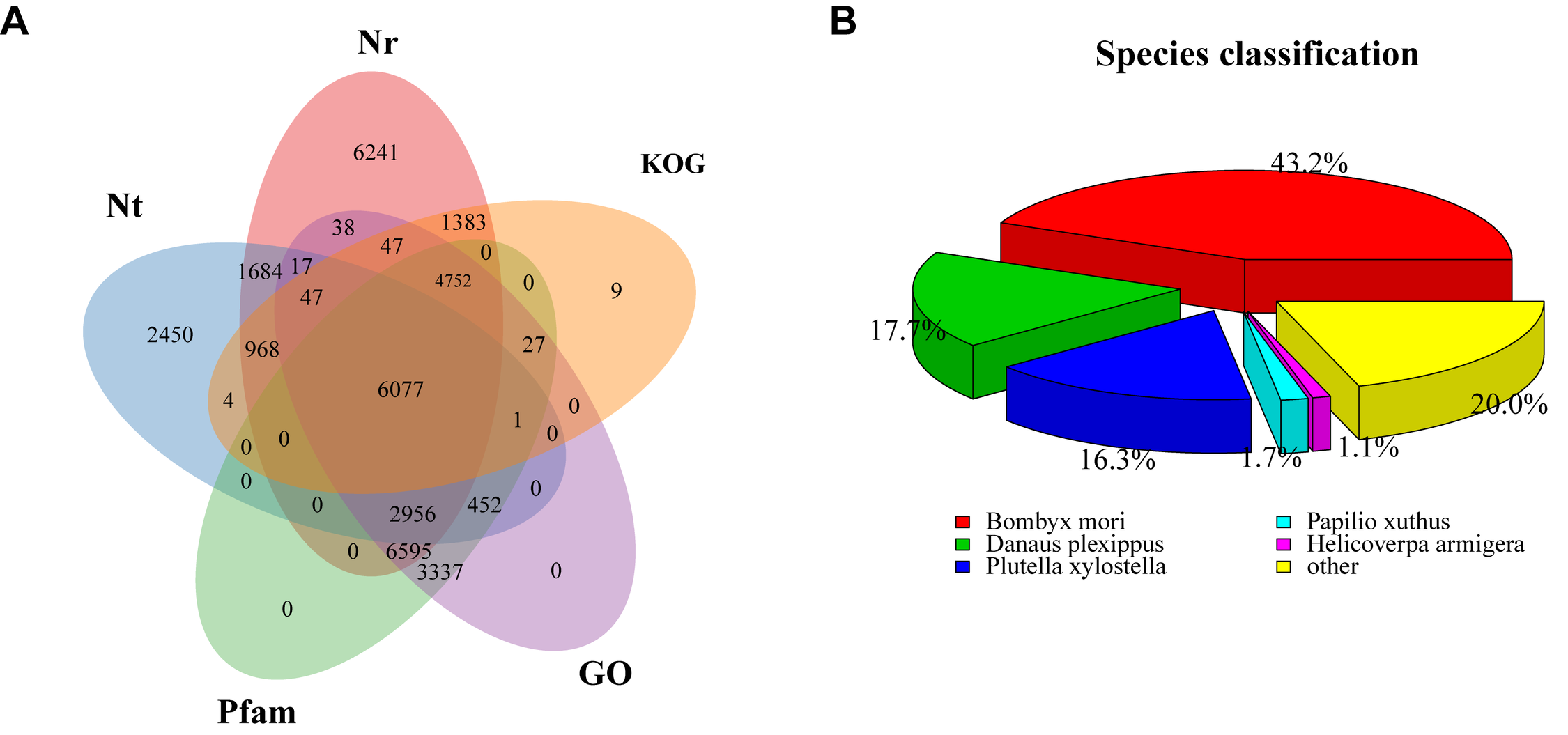

Supplement: S2 Fig — (A) The number of unigenes matching in five databases. (B) The species distribution of the best Blastx hits in Nr database. (TIF) [file pone.0237134.s002.tif]

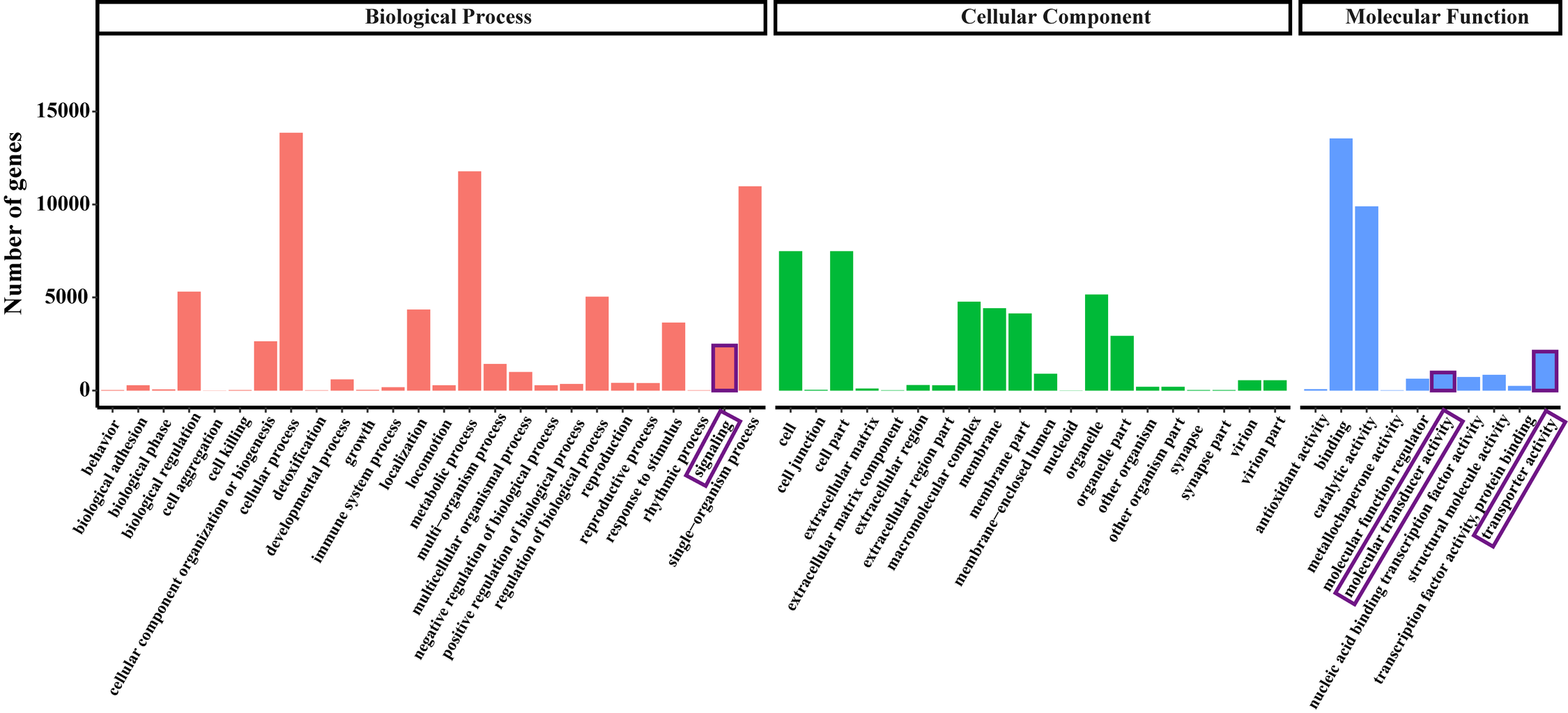

Supplement: S3 Fig — The purple boxes indicate the process of accumulation of olfactory receptors. (TIF) [file pone.0237134.s003.tif]

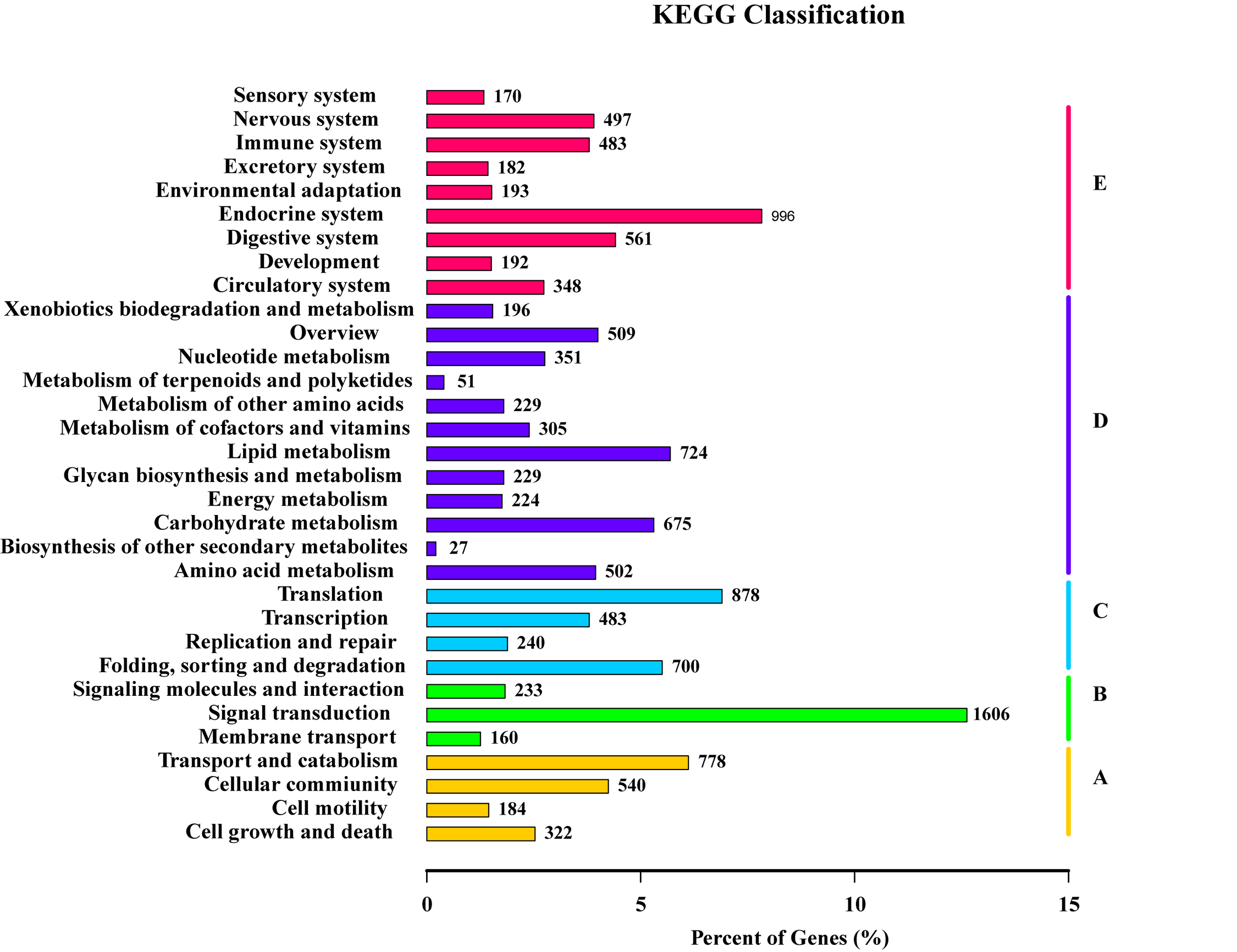

Supplement: S4 Fig — A: Cellular Processes. B: Environmental Information Processing. C: Genetic Information Processing. D: Metabolism. E: Organismal Systems. (TIF) [file pone.0237134.s004.tif]

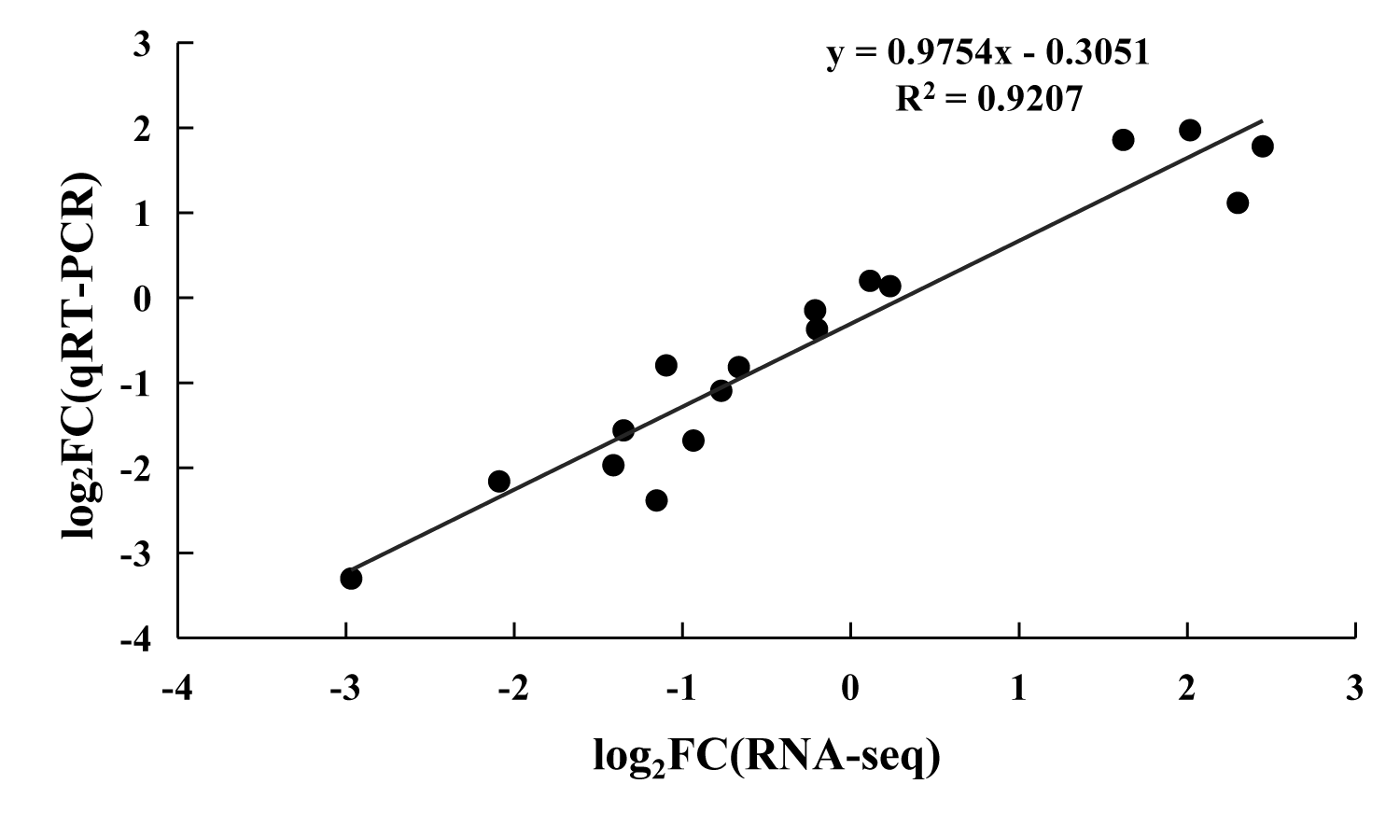

Supplement: S5 Fig — The data was based on the average value of more than three repetitions, and students’ t-test was used to determine the statistical significance. (TIF) [file pone.0237134.s005.tif]

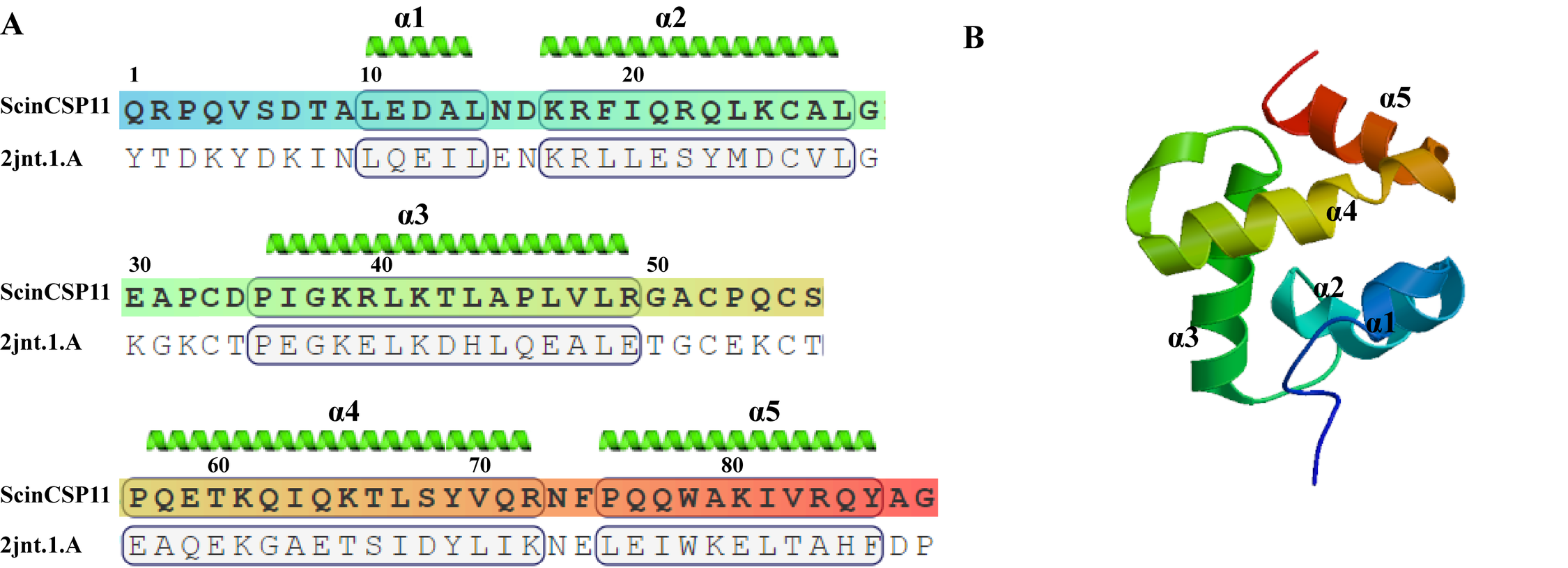

Supplement: S6 Fig — (A) Sequence alignment of ScinCSP11 with CSP1 from Bombyx mori (template library identity: 2jnt.1A). α-helices are displayed as squiggles. The signal peptides are removed. (B) The predicted 3D structure of ScinCSP11. (TIF) [file pone.0237134.s006.tif]

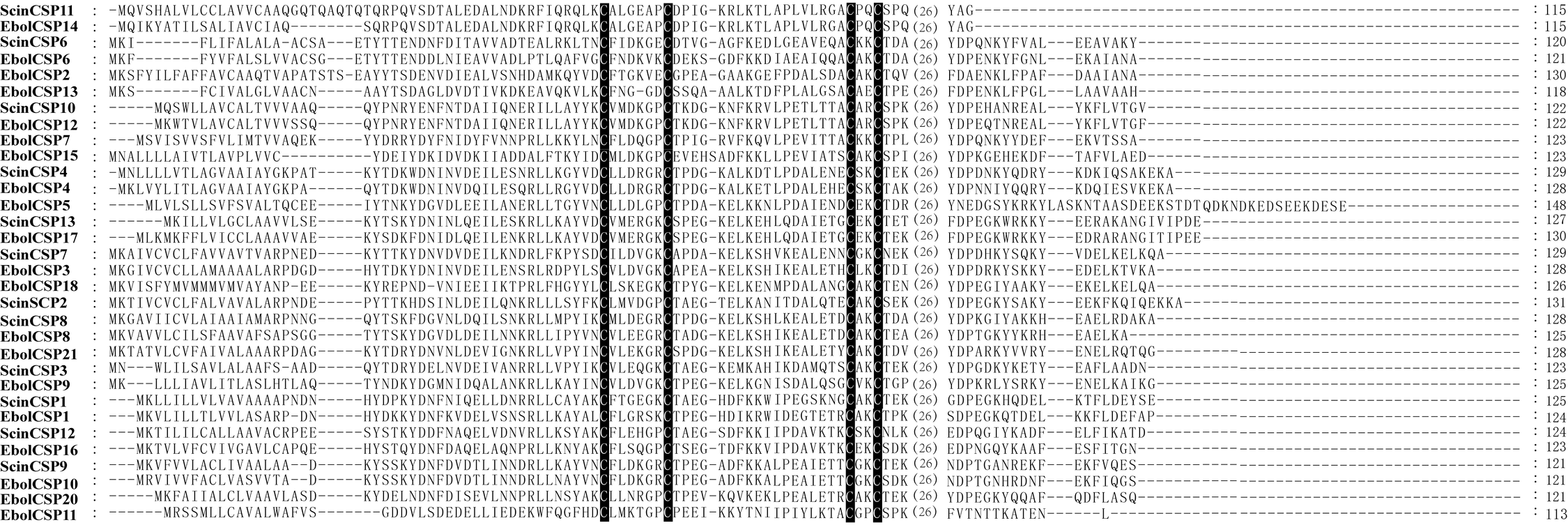

Supplement: S7 Fig — (TIF) [file pone.0237134.s007.tif]

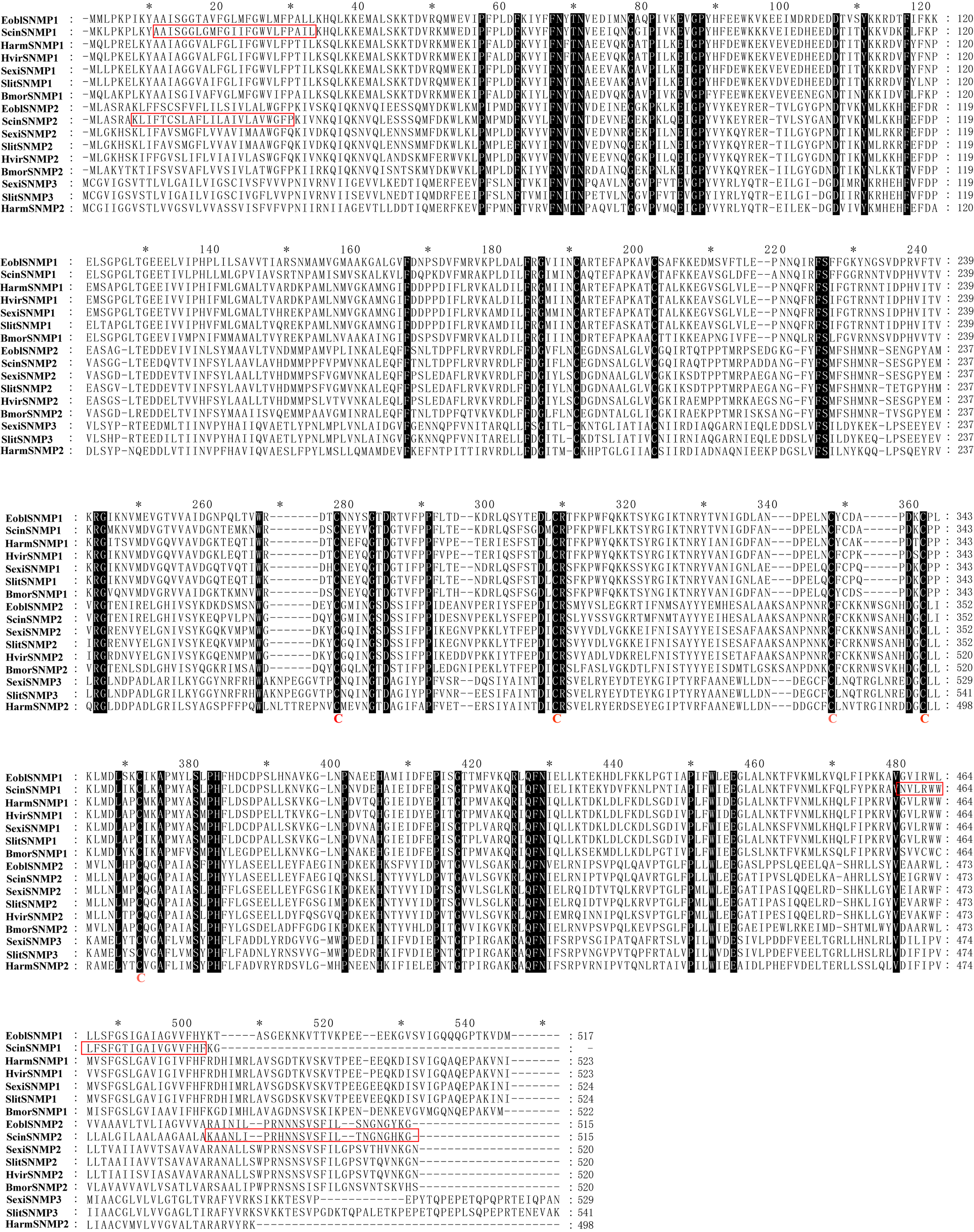

Supplement: S8 Fig — The red letters indicate conservative cysteine residues. The red box represents the transmembrane domains. (TIF) [file pone.0237134.s008.tif]
