# Supplementary material for: Identification of chemosensory genes from the antennal transcriptome of Semiothisa cinerearia
Source: PLoS One. 2020 Aug 7;15(8):e0237134. doi: 10.1371/journal.pone.0237134 (PMC7413487; doi:10.1371/journal.pone.0237134)
Supplement: S1 Table — (DOCX) [file pone.0237134.s009.docx]

**Table S1. Primer used in qRT-PCR.**

| **Primer name** | **Forward Primer sequence(5'-3')** | **Reverse Primer sequence(5'-3')** |
| --- | --- | --- |
| ScinGAPDH | GGTCTACCTCTTCCAATACG | GTCAGTGGTGGTGAACACA |
| ScinOBP1 | GAGTTGACCTGGCTTTGGTG | CAGCACTTCTGTGTCTTCCA |
| ScinOBP2 | GAGGTTATGATGGGATGGAT | GGTAGCCGTATTGATTCGG |
| ScinOBP4 | GTTCGCAAACTCAGTGGTC | CATCGTGTGCTCCTCTTTG |
| ScinOBP6 | CACGGTGTTACAGAGGAAG | CGGTTACAGCGTCAACTT |
| ScinOBP8 | TAACGCTGGCTCAGTTCAC | CACACCTGTAGGCAGTTTAGC |
| ScinOBP10 | GGCTGCTTGTTGACATTACTC | GGCATTCTTGTTTGCTGC |
| ScinOBP11 | GATTGCGATGCGATGACT | CGTAGATTTGACCGAAGCC |
| ScinOBP12 | GCACTACTTTGCGTAACTGTG | CTTTCATACTGCTGTCGTCTCC |
| ScinOBP14 | GACGCGACAGCAACTGAAA | ACAGCGGACCTCATTTCTG |
| ScinOBP17 | GCTTATAATTCTACTCCTCGAC | CGCTTCCTTCAATGCTTTC |
| ScinOBP18 | CTACGCTACGCTTGTGTGA | TCCTTTGTCGTCAATCAGC |
| ScinOBP19 | CCTTCTAACATTCCTCCCG | GCCATACCCATCGTAATCTG |
| ScinPBP1 | GCACAGTATGACGAAGGGT | GCGTACTCCTTAGCGTTC |
| ScinPBP2 | GGCAATTGTTAGCAGTGTAG | GCAAACTCATGAGCGTTC |
| ScinPBP3 | GCTGGCTACTGGCTTTGT | GCAGCCGTGGATTATTGA |
| ScinGOBP1 | CACAAGACAAGATGGAGGAG | ACGAGTGGATGATGGACAC |
| ScinGOBP2 | GTGTTGAAGATGAAGTGCGT | AAGTCCTCGCTCCAGAAGT |
| ScinSNMP1 | CCTGATTCTGGCGATAGT | GTGTCATTTGCTCCGTAG |
| ScinSNMP2 | GTTCGGTATCATCTTCGGA | AGTGTCATCTTCTTCGTGGTC |
| ScinCSP1 | CTGCTGCAGCTCCAAACG | CCACTGATGAACTTGGCG |
| ScinCSP2 | CATCGTGTGTGTTTGCTTG | GAGCGTCAGTTATGTTGGC |
| ScinCSP3 | CTGCTGCGGACCAATACAC | GCCTTCACTCGTTGTTTCTC |
| ScinCSP4 | CATCGCTTACGGGAAACC | GCTTGAGGACTTTGTCGGA |
| ScinCSP6 | GCTCACGAACTGCTTTATCG | CCTCAAGGGCTACGAAGTAT |
| ScinCSP7 | GTTTGTTTGTTTGCCGTGG | AGTTCCTTAGCGTCAGGTGC |
| ScinCSP8 | GCTGTCATCATCTGTGTCCT | CTCTGAACCTTTGCGTTGAG |
| ScinCSP9 | GGCTGCGGACAAATACAG | ATGTCGTCTCTATCGCCTCG |
| ScinCSP13 | CTGCTCGTTCTCGGTTGT | CTTTACCCTCAGGACTGCAC |
| ScinCSP14 | GTGAGGAACTATGTTGACTGCC | CTTTCGCTAAGAACTCCTCG |
| ScinOrco | CTTCTTCGCAAGGTCTACG | GTTCGACTGGTTCCATACAG |
| ScinOR2 | CCTGATGAAGGAGATACCAG | GACGAAGGACAGACTGAACA |
| ScinOR4 | CCTTCTACAACATCGCCATG | GCTCCATTTCCTCAATCTGG |
| ScinOR5 | CAGCCTATTTCTGGTCATGGC | CACATTCGGTCAAAGCGT |
| ScinOR6 | CAGTAAGATCACGTGTAG | CGAGCTCCTGTTAATGATTCTC |
| ScinOR7 | GGAGATGGCTTTCTTTGAGG | ATAACATTGACGCACGCC |
| ScinOR8 | GCTGCTAATTCTACTGTGTATCG | CAAAGTATGGAGACTCGTCC |
| ScinOR10 | GAATTTGAGGCATTGTCGG | CGCACAAACAGTTCAGCTTC |
| ScinOR11 | GAATCATGCCAAAGTACAGC | AGCGGTCATCCAACTTTC |
| ScinOR12 | GGTGTTAGAAACGGTGAAG | CAGAGCAATAACCACAAGAGG |
| ScinOR13 | CAGATTTGGAACTTGGCC | CTGAGTTTCTGTCAGGCC |
| ScinOR14 | GCGGGATTGTATGGTTCA | GTCTGCGTTTCATTCCTTTC |
| ScinOR15 | GCACTTTGAGACCGCATTTC | GCTGTGACGGACCTGAATA |
| ScinOR16 | CAAGCAGCTCGTGTTCTG | GTACAGCAGCACCAGTGAG |
| ScinOR17 | CTGCTTGAACCTGCTTTACTAC | CGAAGTCACATATGCCATC |
| ScinOR18 | CGTACCTGAAACAGCAAGTG | TCGTCCAAGAGCTTCGTG |
| ScinOR19 | CTGTGTCGTTGAATGTGGTG | CCATCAGAGGAACATCACC |
| ScinOR20 | GGAGGAACTGGTGAACGAA | GCACCGTCAGAAATACATACAG |
| ScinOR21 | CATTGTATTCTTCCTCGGCT | CCTCTTGGGTCTTCGTCAA |
| ScinOR23 | CGAGGTGTCTATCTGAAAGC | GCCAAACCAATCTCAAACG |
| ScinOR24 | GTAGACCTGTTCCTCATCTGG | GCAGTTGATTGCTCTCAGC |
| ScinOR25 | GCAGCATTCCTCGTTTCTAA | GCTGTAGAGGCGGCTAAGAT |
| ScinOR27 | GCGACCGATGTAATAGACG | GCATAGAAAGTAGCCACGAG |
| ScinIR1 | CCTGGACTCGCTATGTAAAG | GCTATTGTCGGCATTCCA |
| ScinIR2 | CGACTGAGAGAGATGTGGATAG | GGTAGTCCGTAAACACCAGG |
| ScinIR3 | GTGAACTTGCTCGTTATTGGTC | CCCATTGGACTCTTGGATT |
| ScinIR7 | GCAGGAGACCTTAGACAATG | CGTAGACTTCGCAACTGAG |
| ScinIR9 | GCTCAGCCTATTGAATGCG | GCGTTCCACTTGGCTATTC |
| ScinIR10 | CCAAACAGAACGGTTGATG | CAGACTGATTACCTGGAGC |
| ScinIR12 | GCAATGCGACTTTCTGTG | GTTGTCACCTTTCTCTACCC |
| ScinIR14 | GGTTTCCTCGCAGTATTCAG | CGTTGCTGGTCCTCAAAT |
| ScinGR1 | GTGTATTGGTTCCAGGCG | ACTTGAGAGGTGCCAGTC |
| ScinGR3 | CGATTGGTTCTTTATGCTCG | ACACACCAGATGATGACGG |
| ScinGR5 | CGTCAATCGGCTCTTGTT | CGAGTCAGGATACGAGTGT |
| ScinGR7 | TCGTTTGTCCGTGTGTCTC | TCGTCTCCATCTCCATAC |
| ScinGR8 | GTGTTGTTGGCGATAGTC | GATAGTTCAGCCCACGAA |

Note: GAPDH was used as an reference gene.
