# Supplementary material for: Identification of chemosensory genes from the antennal transcriptome of Semiothisa cinerearia
Source: PLoS One. 2020 Aug 7;15(8):e0237134. doi: 10.1371/journal.pone.0237134 (PMC7413487; doi:10.1371/journal.pone.0237134)
Supplement: S2 Table — (DOCX) [file pone.0237134.s010.docx]

**Table S2. BUSCO assessment of *Semiothisa cinerearia*.**

| BUSCO benchmark | Number of genes | Percentage |
| --- | --- | --- |
| Complete BUSCOs | 869 | 88.9% |
| Complete and single-copy BUSCOs | 579 | 59.2% |
| Complete and duplicated BUSCOs | 290 | 29.7% |
| Fragmented BUSCOs | 65 | 6.6% |
| Missing BUSCOs | 44 | 4.5% |
