# Supplementary material for: Identification of chemosensory genes from the antennal transcriptome of Semiothisa cinerearia
Source: PLoS One. 2020 Aug 7;15(8):e0237134. doi: 10.1371/journal.pone.0237134 (PMC7413487; doi:10.1371/journal.pone.0237134)
Supplement: S3 Table — (DOCX) [file pone.0237134.s011.docx]

**Table S3．Summary for the annotation of *Semiothisa cinerearia* unigenes.**

| Databases | Number of Unigenes | Percentage (%) |
| --- | --- | --- |
| Annotated in Nr | 30805 | 47.04 |
| Annotated in Nt | 14656 | 22.38 |
| Annotated in KO | 12722 | 19.43 |
| Annotated in SwissProt | 21887 | 33.42 |
| Annotated in PFAM | 24197 | 36.95 |
| Annotated in GO | 24346 | 37.18 |
| Annotated in KOG | 13315 | 20.33 |
| Annotated in all Databases | 4733 | 7.22 |
| Annotated in at least one Database | 37148 | 56.73 |
| Total Unigenes | 65476 | 100 |
