# Supplementary material for: Identification of chemosensory genes from the antennal transcriptome of Semiothisa cinerearia
Source: PLoS One. 2020 Aug 7;15(8):e0237134. doi: 10.1371/journal.pone.0237134 (PMC7413487; doi:10.1371/journal.pone.0237134)
Supplement: S4 Table — (DOCX) [file pone.0237134.s012.docx]

>ScinOBP1

MKQLCWLLIVSVVMAAPPPMDEDMAELAAMVRSSCKDESGVDLALVEKVNAGNTLMDDGKLKCYIACVMETAGMMSDNEVDVEAVLALLDDNTRARNEKALRACGTQKGAEKCETAWKTQKCWQDANPGDYYLI*

>ScinOBP2

MGSYDERDSNSGYGENTRGYDGMDGRMGSYDSRMGSGSHRYGNDREDSMRNHDSGYGDRDHGYNGRNDFQQSDYGSDMPNQYGYQSSTQSSRRYKREKRTENSGQRSQFNPNSKKSSFDDSYQSDEKSGKENSTSKDDDKKACALHCFLENLEMTAEDGMPDRYLVTHTLTKDVNNEDLKDFLQESIEECFQMLDNENTDEKCEFSKNLLMCLSEKGRANCDDWKDDLRF*

>ScinOBP3

FQGATEEEITALRAVVRPLVEDCAKQFGITIEKITEAKKAGNLNFLDSCLYYCFFNKVGFIDSKGLFDASTAIEKNKKYFKDEADVQNIERIGKKCLSVNEETVSDGDKGCERSKLLLSCFLKEKADFTVFQFQ*

>ScinOBP4

MVRKLSGLLCCLCVFGISLSDSAISADSEQRCKNPPTAPQKIERVITLCQDEIKLSILREALDVIKEEHTMPTQRKRNKREVPFTHDEKRIAGCLLQCVYRKVKAVDGYGFPTLEGLVGLYSDGVNERGYFMAVLEASRECLMRNHDKFSRTVPMDNGRNCDISFDIFECISDRIGEYCGNAGL*

>ScinOBP5

KRVFPRLTINPAWPSGLSHRHGLPEIQGLIKEQNHVITPRINAVSSECAQTTGMTAEQVEAAIRDKDINQLSACFFRCYINHFGVIRQEGSKDVVNVPAMLIFVKMYMPSISILELLSIASEIMVCAKQFENSSLQDEQPDCLKTKSLITCLAKKDTLINAFWIATQS*

>ScinOBP6

MAKVHCVLLLGVVAAAFSVVSAGISDADKETIKALLGPILKECGKEHGVTEEDILAAKKTHDVDALDSCFYRCFFKKIGLIDDQGLYAVETAKENHKKYIKDEEFLKKVDAVTDECSVINEKEVSDGTEGCDRAKLLINCLKEHKDSINPILE*

>ScinOBP7

NIFLIIVVIFQVATACKNCVVLGKAEKAMFRAHSEACAAQSGAAPRLVDALLAGELADDAALKRHVYCVLLKCKVVGKDGKLLKTAVLGKLAMRADGKNATKILEACSEQAGAGALSPEDAAWDLFRCGYDRKAVLFDYMPTDKLPADN*

>ScinOBP8

MNTYLVVCFAFAVAGINAGSVHLTGAQKDKAAQNVVECMKETGTKADVVAEVKKGHFTDDENIKKFTLCFFEKAGVVDHDGKLNVEAALAKLPTGVDKAEAKKLLEDCKNKKGKDAADTAYEIFKCYHAGTKTHILL*

>ScinOBP9

VGKTLQIFYAKMMLISVFEVLVLFAVSEAMTMKQLRNTGKMMRKSCQPKNNVEDEKIDPINEGVFIEEKEVKCYIACIMKMANTV*

>ScinOBP10

GGLGCLLTLLVGVQVISSQGPPGPPPHHGGGPPHKCAGPPQGVKDPQSCCQAKPMFSEEELAECGFEKFDEESRQGPPQPPHCSKQECLLKKRDLLNADSTINYDAVRAYMQSWVDANPEFKSAVETATSVCLGENGPPGPKQVCEANRLMFCVGAVVFTNCPNWQDTDDCKRLKEHMAECRGPNFPPPN*

>ScinOBP11

MENYWVILVIVIILAGDCDAMTREQLKKTGKMLRKQCLAKVDVEEDKISQIEKGKFVEEKEVMCYVACIYQMTQIVKNNKLSYESAIKQVDLMYPADMKASVKSTIEKCKDVSKKYKDVCEASYWTAKCMYDDNPKDFIFA*

>ScinOBP12

MSNFALLCVTVTLILVSLVTCSKMSTEMTTQTPKPTENSKMMDKSKTADNPEMSVPKTAESKVMGDDSSMKDDMDLMDAMTMCNESFRTEMSFLISLNESGSFPDETDKTPKCFLRCVLQTLEIATMDDGKIDPARAASF

>ScinOBP13

MIVKFLPRPSIPTVCPQCFLRCVLQTLEIATMDDGKIDPARAASFFAEERDGEDVVEIATVCAQRDEDNHCEMAYNFLKCLFSMEIERNNKKTQT

>ScinOBP14

MSPKQGVLLSLFVSVFLIFIQDSAAMTRQQLKNSGKLMKKTCMPKNDVTEEQVGDIEKGKFIEDRKVMCYVACIYSMTQVVKNNKLSYDAVIKQVDMMFPPEMRSAVKAAAENCKDIGNIIL*

>ScinOBP15

KYTMCVYLKTAIILMAISFALAMTRQQIKSSGKLLKKTCMPKNDVTEDQVGDLDKGKFLEVRNVMCYIACVYSLSQVVSIYNSIIYRKSIWEYYSISLN*

>ScinOBP16

QFNYLPIIILQKVVHVPPELAGKILTALQKCQPETGIDGNALLMIYDGKYRDDKQFKDFIYCSYKTTGYLKSDGYLNEEKAIKAFRNEPLIEEGIRRCGPLRGSNPKESLFMFFKCFIDTTPVQIGI*

>ScinOBP17

MIEIKRPVKCIKVFHHILKPLNHFFNAYNSTPRQAQLTEAQKAKIYGNLLTGGMECMKDFPLSLDHITAFRNKKVPDDEVVNCFSHCLYKKLGLMDNSGKLSEKKALKEAKKNLS*

>ScinOBP18

MLRYACVSFVILGSFLVAAGLQEEKQKLHDYWSEAFKSCSKEFGISLEDVEATKENKSDIDPCFIKCILKSNKLIDDKGMFDPKQGMEIAEMFITNPDELAKIKKVSDTCQSVNDEKVSDGEKGCERSVLLMQCLMENKSQLL*

>ScinOBP19

MLSSVNTVILLTFLPVLAKCTGEGNIKLLEEEVAAALKTCAESDNSSPKVQRQKRYADNAPRIDDGSQTEASQYSHERRNFTDMKDQIYVINSTDTDYDGYGSGNMGEKFVNSMPKAAAGRNYPEVDVNGINRTRRSEPLFSKPDTDQCLSQCVFANLQVVDSRGIPREAELWNKVQSSVSSQQSRAALKDQISACFQELQSEAEDNGCSYSNKLERCLMLRFTDRKQSGAKTNDNSGAKST*

>ScinOBP20

TSVKLPDDVHASVMKAFDKCSTNKEQIPDILRRMTAGEVIDDPDFRKLLHCVYLETGFIKEDGRLLVEKAAALFPDKEAMLKILQECDKQVVMSNPVDVVIQHFKCFQLTTPNRLALSP*

>ScinPBP1

MLQIARFSLIWRVLALFGTFLAKIEDGQCSQEVMHSMTKGFARVLEDCKKEEHVGDHVMQDFFNFWHEEYELVNHELGCVVLCMAGKLELIDDDMRMHHGNAKEYAKAHGADDELAQKLVDMVHSCEQASAAIEEKCARALDTVKCFRTKIHGLKWAPSMRVIMEEVMAAG*

>ScinPBP2

MGDKVKNNNGKVDKITLFFVALAIVSSVDCSQNVMQEMTLNFGKALSECKKELSLPDTVFEDFYNFWKEGYELKHRQTGCAIHCLSSKLDLVDPDGNLHHGNAHEFARKHGADATMAQTLVDTIHSCEKSTPDNSDICLKILDVAKCFKVEIHKMNWAPNMDVVIGEVLAEL*

>ScinPBP3

MEWKHVLVVVVLVTVRRAEGGDAMKLLATGFVKVLEECKKELNLDDNLISDLYHYWKLDFSLLQRETGCAIICMSKKLELLGEDGKLHHGNAKEYAMRNGAGDELATKMISIIHGCEDKGEGIEDDCARVLEVAKCFRVGLHDLHWEPKVDVIITEVLTEI*

>ScinPBP4

MHISDQIQTKFNQMVIREFLSYWDENIPLIDPDFGCVVMWILQKLELVEDNGILRQENAKAFMMAKGSDEITSETLIKLYALCLRSIDLRPEQGECQFGLMLAQC

>ScinGOBP1

MKDVTLGFGQALEKCREESQLTQDKMEEFFHFWREDFTFEHRELGCAIRCMSQHFNLLTDASRMHHENTHKFIKSFPNGEVLAQQMVSIIHSCEQQHDTEADHCWRILRVAECFKHSCQQAGVAPSMELMMTEFIMDAE*

>ScinGOBP2

MSEPLVVGKVLKMKCVLALLMVAVVCQMAAGTAEVMSHVAAHFGKALNECREESGLSPNILEEFHHFWSEDFEVVHRELGCALICMSNKLTLLQDDVRMHHLNMHDYVKSFPKGDVLSAKLVELIHNCEKQYNEIPDDCDRVVKVAACFKDDAKKAGIAPEVAMIEAVLEQY*

>EoblOBP3

MAKLFLFVVGVAVSLGIASGASQEEITAVRTAIRPFLDECGAEFGITRDQLLAAKAAGTVDTFDPCFYACFFKKIGFIDAKGLFDANVALEKNKKYFKAADDIAKIEQMGKTCSSVNDESVSDGDKACERSKLLLKCFLKEKANFTPFESS*

>EoblOBP4

MVRKFSGLLCCLCVFGISLSDSAISADTEQRCKNPPTAPQKIERVITLCQDEIKLSILREALDVIKEEHTMPTQKKRNKREVPFTHDEKRIAGCLLQCVYRKVKAVDGYGFPTLEGLVGLYSDGVNERGYFMAVLEASRECLMRSHDHFSRTVPMDNGRNCDVSFDIFECISDRIGEYCGNAGL*

>EoblOBP5

MLKLLGFFFVTWTFVVGVLGAEKLNDLKQEYDEILKECIAQNPMTAEDVESLSKDKRTYNVNCIFACALKKGGMMDDDGNLSVEGVRKSAEAYLSDDPELLKKSELFTDACKSVNDAPVSDGKKGCDRASLIFQCSVEKAPSFQLF*

>EoblOBP6

MAKLQCFVFLGVLAVFSVASAAISEEDKNAIKAEMLPVLAECGKEHGVTEKDVKEAKESNNVDAINPCFIACFMKKRKIIDDEGKYAPEVAKSEHAKYIHDAELVAKLDEISDNCASVNDQAVSDGAKGCERAKLLTACLTEHKDILTEIFKD*

>EoblOBP7

MKILFCFVLLTYTSGDLIGQPRNNKEATLKPISTCCDIPELGDPKPLAECSNPKLPGPCNDVQCVFEKSGFLIDKSTLNKETYKAHLRQWAEKHKDWSAAVERAIEDCVEKNLRQYLDIPCTAYDVFTCTSIAMLKKCPASSWKC*

>EoblOBP8

MQASILFGLVFVAAGINAGSVHLEGAQKDKAAETAMQCMKETGVKPEMVAEVKKGRLSEDEDLKKFTLCFFQKAGIISPDGKLNVDVALSKLPAGVDKTEAEKLLNDCKTKKGKTAADTAYEVFKCYQAGTKTHILL*

>EoblOBP9

MTTATVPVFLALIAVAYCGKDKPVFTDEMNEIIQTIHDACVGNTGVSEEDITNCENGIFKEDPKLKCYMFCLLEESSLADEDGVVDYDMLLSLIPEEYYDRTSKMILGCKHEDAPGKDKCQSAFDVHKCSYQKDPDLYFLF*

>EoblOBP10

MVGLGGLLLLVGLQIIASQEQGPPHGPPPQWANHKCAGPPPAIKNPQKCCEIQQMFTEEEMASCGINKFEEENRQGPPKPPDCNKQECLLKSKDCLNDDGSINHKAVAEHLNNWASEEWKPAVEAAVAVCLGENEVPGPPHICEANRLMFCIGGVIFSECPTWQDNDDCKQLKEHINECKAAKFPPPN*

>EoblOBP11

MEKSYWVVLITAIMIAGDCDAMTKEQLRKTGKMLRKQCLGKVGVEEEKISQIEKGKFIEEKDVMCYIACVYQMTQIVKNNKLSYESALKQVDLMYPADMKASVKASIENCKDVSKKYKDVCEASYWTAKCLYDDNPKDFMFA*

>EoblOBP12

MNYTLLCLSLTITSVSLLVTCSKLSTETTKVTATTESSNNMKPDDNNKELDDSKPASDTSRIGIDTKYTSNDTNALYDEVMDVLTTCNESFRIEISYLVSLNETGSFPNETDKTPKCFLRCVLQSLEVASMDDGKIDPKRAAEVFGDQRENIEETATLCAQRDEKCHCEMAYNFLKCLFSTKIENVEKSKT*

>EoblOBP13

MWSSKINPGAALAVCVFLLQMLESHAMTRQQLKNSGKLMKKTCMPKNDVTEEEVGSIEQGKFIEDRRVMCYVACVYTITQVIKNNKLSYEAVIKQVDMMFPPEMRTAVKAAAENCKEIAKKYKDDICEASYRTAKCMYEYDAENFVFP*

>EoblOBP14

MFRLVGFICVCAALTPYLASAMTAEQKQKIHEHFETIGMKCMKDHHITEADITDLRAKKVPSGPEAPCFLACVMKDIGVMDGNGLIQKETALELAKKVFEDAEELKMIEDYLHSCAHVNTEPVSDGDKGCDRAIIAMKCMIENASQFGFEL*

>EoblOBP15

MTNSTTFSKYSVMFTSFIFLTILTLSATMTMKQLRSTGKMMRKSCQPKNNVEDEKIDPIADGVFIEEQEVKCYIACIMKMANAIKNGKLNYEAAIKQADLLLPDEIKEPAKESITVCRKVSDQYKDICEASFHTTKCIYNNNPAAFYFP*

>EoblOBP16

MKTLIVLAVCFVAAQALSNEQKEKLKKHKTECLAETKPDEQLVNKLKTGDYKTENEPLKKYALCMLIKSELMTKDGKFKKDVALAKVPNAADKPAVEKIIDACLANKGNTPQQTAWNYVKCYHEKDPKHPIFL*

>EoblOBP17

MYKFGLVCFVLAASVVLKNDAAQLTASQKSKIYGSVLSAGMECMRDFPLSLDHIQAFRNKKAPNDEVAKCFTHCLYKKLGLMDDSGKISEKTAKAATKKVFKEGDEMFTKVEELISRCIHVNDAETSDGDKGCDRAKLAFECFIEHAKELDLDVDL*

>EoblOBP18

MMRYVCVIAVILAGFMAAGVTDEEKKQMHDYLVSATESCSKEFGIPSEDFEKAKRNKELQSLDPCFVACILKGNGLIDDKGMFDPAKGTSIAEKFIKSPDDIAKVKKISDICSSVNDEAVNDGDKGCDRAVLLLKCLMENKSLVV*

>EoblOBP19

MKDQISVINSTDYDYDGYGSGSMGEKFVNSMPKAADGRYYPYANGTNRTRRSEPLFSKPDNEQCLSQCVFANLQVVDSRGIPREAELWNKIQSSVTSQQSRAALKDQTSACFQELQSEAEDNGCSYSNKLERCLMLRFSDRKPSGTQTNNKQGTK*

>EoblOBP20

MFKILLFICAVSAVNCEIVRTAITLPPEIAFDIAKAIKEVCVPEDRVPDIIRMIREGETNNNTEFKKIIHCVIKEAKYMTADGKRINVEKAASIFPNKVLMFKILSQCDKNIVTNDPEEYCIKFYDCFQENTPYRLSF*

>EoblOBP21

MISQLTLVLLLVGACYGRTDLEVKGWFFSLAVICNKDYTIAPEELAMMQDHRISDSPNAKCLMACIFKKADMMDDKGNYDLEKTNKWVETEFSDSATRLESARNLFNMCKKVNDEPVTDGEKGCERAYLLSKCLVENSPKIGFATIE*

>EoblOBP22

MARFSFVAFLGVAAVINIALAITEDEKNNIRLNALPVLTSCAQELGIKMEDVVAARQAKNLDALNPCYYACFFKKINVIDNDGLFVPAVAKANHQKYVHGADDLARLSASADTCTSVNDQAVTDGANGCDRAKLLARCFIDNHGVGPFSA*

>EoblPBP1

MARFTLSWRILALFAVFLAQIEERECSQEVMHKITKDFAYVLEDCKKQENVGDHIMQDIFNFWHEEYALVNPELGCVMLCMAGKLDLMDGDDMHHGNAHEFAKKHGADDDLAKQLVTMIHDCEKASASIADRCARALETTKCFRGKIHGLKWAPSMRVIMEEVMADMNV*

>EoblPBP2

MTKLKELLLVLVISVITRVQSSQDVMKSLTLNFGKPMEVCKKELDLPDAVTKEFLNFWREGYEVKNRLTGCAIICMSEKLELLDEGLKLHHGNAKDFAKKHGADDGMAQQLVDMIHSCMESTPPNTDPCMKTVDVAMCFKLKIHDLSWNPDPDLIIAEVLAEA*

>EoblPBP3

MWWKLVFVVVVGSAVVGTTEAADAMKLLASGFISVLEICQKELNIEDGLISDLYHYWKLEFSMMQRDTGCALICMTKKLELLTDDGKFHHGVTKEFAMKNGADDNLATEMVSIIHSCETKSEGLDDECLRALEVAKCFRVALHDLHWEPSPDVVITEVLGEM*

>EoblPBP4

MAKYHFNKSLVLNVFLTVFLYFNYGVDADSNIMKNLSLKFGEAMSICKAELNLPDSINEDFYNFWKPDYELQHRETGCMIHCLSTKLNLIDPEGKLHHGKAKEFAMSHGADEGMAQQLIDIIHNCENSTPQNEDGCLMVLAVAKCFKVEIHKLNWTPSMDMVVGEVLAES*

>EoblGOBP1

MARLASSVLAVVAVAAAAVVADVQVMKDVTLGFGQALEVCREESQLSQDVMEEFFHFWREDFKFESRAVG

CALQCMSRHFNLLTDSSRMHHENTHRFIESFPNGSVLAKQMVSLIHGCEQQHEAEPDHCWRILRVAECFK

RRCQEAGIAPSMEIIMAEFIMETEAK

>EoblGOBP2

MKSVLVATVVLSVVGLAMGTAEVMSHVTAHFGKALSECREESGLTPEVLEEFQHFWREDFEVVHRELGCA

IICMSNKFSLLQEDSRIHHVNMHDYVKGFPNGQVLSAKMVELIHNCEQQYDDITDDCARVVKVAACFKRD

AKKEGIAPEVTMIEAVMEKY

>BmorGOBP1

MWKLVVVLTVNLLQGALTDVYVMKDVTLGFGQALEQCREESQLTEEKMEEFFHFWNDDFKFEHRELGCAIQCMSRHFNLLTDSSRMHHENTDKFIKSFPNGEILSQKMIDMIHTCEKKFDSEPDHCWRILRVAECFKDACNKSGLAPSMELILAEFIMESEADK

>BmorGOBP2

MFSFLILVFVASVADSVIGTAEVMSHVTAHFGKTLEECREESGLSVDILDEFKHFWSDDFDVVHRELGCAIICMSNKFSLMDDDVRMHHVNMDEYIKGFPNGQVLAEKMVKLIHNCEKQFDTETDDCTRVVKVAACFKKDSRKEGIAPEVAMIEAVIEKY

>BmorPBP1

MSIQGQIALALMVYMAVGSVDASQEVMKNLSLNFGKALDECKKEMTLTDAINEDFYNFWKEGYEIKNRETGCAIMCLSTKLNMLDPEGNLHHGNAMEFAKKHGADETMAQQLIDIVHGCEKSTPANDDKCIWTLGVATCFKAEIHKLNWAPSMDVAVGEILAEV

>BmorPBP2

MKLQVVLVVLTVEMVCGSRDVMTNLSIQFAKPLEACKKEMGLTETVLKDFYNFWIEDYEFTDRNTGCAILCMSKKLELMDGDYNLHHGKAHEFARKHGADETMAKQLVDLIHGCSQSVATMPDECERTLKVAKCFIAEIHKLKWAPDVELLMAEVLNEVSWKS

>BmorPBP3

MARYNIVVAVLVLGVVGARGSSEAMRHIATGFIRVLDECKQELGLTDHILTDMYHFWKLDYSMMTRETGCAIICMSKKLDLIDGDGKLHHGNAQAYALKHGAATEVAAKLVEVIHGCEKLHESIDDQCSRVLEVAKCFRTGVHELHWAPKLDVIVGEVMTEI

>BmorOBP5

MKQRLRVLLLRFCILQTVLSESGVDVVKNLSLSFARFFLECDEERHFQPEVRLKVMTFWYSESSTWDRDVGCAFLCIFKKMEIDNPQDPSYRTHLELLSFANSEDNKIANQMVEIFYACGENTETDPCLWALEQVKCYKNRINQLGLTPTF

>BmorOBP7

AVTEEELKIEFTKLVMKCTKDHPVDMSELMQLQQLIAPKKTESKCLLACAYKLNGVMTSQGLYNLEHAYKIAEMSKNGDEKRLENGKKVADICVKVNDVEVSDGE KGCERAALIFKCTLENAPKVFKFGSSEYNCQ

>BmorOBP8

MLRVVVICVCFLVIAPYGINASSLDDLKMVYKNVIKECVGDYPITAADLKLIKARQIPNDDIKCVFACAYKKTGMMTEEGMLSVEGIKDMSQKYLSDNPEQLRKSKEFAEACSSVNDQQVSDGTKGCERAALIFKCSTEKITNFGFEL

>BmorOBP9

MLRVVVICVCFLVVAPYGINAVSYEQKIKIRDQLDRAGFECFKDHKITEDDIKNLRANKPATGENVPCFIACVMKKTGVMNDQGVIRKGPVLELAKKVLADDKDIKKLQDYIHSCSHVNSETVHDKGKGCEFAMQAYTCMSANASKFGFNI

>BmorOBP10

MLRVVVICVCFLVIAPYGINAVSDEQKIKIREQIDKSGFECFKDHKITEDDIKNLRARKPATGENVPCFIACVMKKTGVMNDQGVIHTEPVLQLAKKVLTDDKDIKKLQDYIHSCS HVNSKTVHDKGQGCEFAIQTYTCMSANASKFGFDV

>BmorOBP11

MSANSFVVLAFCALAVGVNALTEEQKAEITKSSLPLIAECSKEFSVNQGDIDAAKKLGDPSGLNSCFVGCFMKKAGIINASGLFDVAATIEKSKKYLTSEEDLKAFEKLTETCAPENDKPVSDSDKGCERAKLLLDCFVANKGSFSVFSL

>BmorOBP12

MTSFMVFFVLSVLTLKYSDALTDEQKNKIQSKFIEIGAECIVEHPISIDDINSFKNKKFPSGVNAGCFVACIFNKIGLFDDKGNLSHNSALEKAKGIFNADEEVKNLEEFLNRCAKVNGEAVGDGVKGCERAKLAYNCLIENSLEFGFNIDF

>BmorOBP13

MLKIHVLLCFGMAILYFGSAKAVTPEESKAFEAFAKPVIEQCQKDFGMDKESFAQKNLDEIDECLIACVVEKFGITNDEKIDGDALKALVTKFVGNEEERNKINKIVEECTEDANKSGDGTCNTSTILFLCLLKNGKDLWGF

>BmorOBP14

MSRQQLKNSGKMLKKQCMGKNDVTEEEIGDIEKGKFIEQKNVMCYIACIYQMTQIIKNNKISYEASIKQIDLMYPPELKESAKASAGRCKDVSKKYKDICEASYWTAKCMYEDNPKDFIFA

>BmorOBP15

MFLKNIFIECVLLYFVMLNTSFVNTMTKQQIKNSGKILKKACISKNDVTEDQISDIDKGKFIEDKNVMCYIACVYSMSQVVKNNKFVHDAMVKQVDMMFPTEMRDAVKASIANCRGVAKNYKDICEASFWTAKCMYEFDPANFVFA

>BmorOBP16

MRISFLFLISVTIITFDSVFAMTRAQVKKTMTIMKNQCMPKNGVTEDQVGKIEEGIFLENHNVMCYIACVYKTIQVVKNDRLDKDLISKQIDVLYPQEIRESTKKAVGDCINLQEKYDDWCEGIFRSTKCLYEKDPANFIFP

>BmorOBP17

MTRQQLKNSGKIMKKTCMPKNDVTEEEIGQIEQGKFLEQRNVMCYIACIYTVTQVVKNNKLSYDAVIKQVDVMFPAEMRPAVKAAAENCKDISKTFKDICEASYWTAKCMYDFDPKNFVFP

>BmorOBP18

MILIVIAKFLILISLCETMTMKQIKNTGKMMRKSCQPKNNVDDEKINPINDGVFIEENEVKCYIACIMKMANTMKNGKLNFEAAMKQADLLLPDEMKEPTKEAIVACRKVADSYKDVCDASFHVTKCIYNHNPSVFFFP

>BmorOBP19

MTSAKTDVEIKAWFLGQAVECSKDHPVTTEELRMHKHELPDSKNAKCLMKCVFRKCNWLDSKGMYDINAAYASSTKDFSDDKTKQENANKLFDTCKSVNEENVGDGEEGCDRSLLLAKCLTKAAPQVSIYYS

>BmorOBP20

MAVHIFLILASYMALAAHGQLDDEIAELAAMVRENCADESSVDLNLVEKVNAGTDLATITDGKLKCYIKCTMETAGMMSDGVVDVEAVLSLLPDSLKTKNEASLKKCDTQKGSDDCDTAYLTQICWQAANKADYFLI

>BmorOBP21

MITASLHVIFALLAFVYGGKDKPVLSEEIKEIIQTVHDECVGKTGVSEEDITNCESGIFKEDVKLKCYMFCLLEEAGLVNDDGTVDYEMFTSLIPEEYFDRATKMIFSCKELDTPDKDKCERAFEVHKCSYEKDPDFYFLF

>BmorOBP22

MLKVFVVVVCTLGASQLCAALYTQKVAVSFPKDKTTIVVEAMKSCIAKTGANPNVIEVISSGKVSEDEKFKEFFYCACNDIGVVNPDGHIKVKECIELFPKETQPLVEPVI KNCDKEGVNKYDTLFKYLKCFQETSPVRVTLA

>BmorOBP23

MTSKVLLSCVVLAVLATTVLAEDSRKLVSFAPEVAKKLKVLIQECLNENGLGEDAIEVIRAGEYREDEPFQNLVYCAYKKFGALDENNRIISQVAAASFPKDIDVVTVIESCGKEDGNTPVEQVFKYFKCFQKNSPVRMQLY

>BmorOBP25

MKSVVLICLAFAVFNCGADNVHLNEDEREKANWYTAECGVETGVSTEVINAAKIGKYSKDKAFKKFVLCFFKKSAILNSDGTLNMVVALAKLPSGVNKSEAQSVLEQCKNKTGQDAADKAFAILQCFHKGTKTHILF

>BmorOBP26

MKSVVLICLAFAVFNCGADNVHLAETQKEKAKQYTSECVRESGVSTEAINAAKIGKYSKDKAFKNFVLCFFNKSAIFNSDGTLNMDVALAKLPPGVNKSEAQSVLKQCKNKTGQGAADKAFEIFRCYYKGTKTHILF

>BmorOBP27

MKSVVLICLAFAVFNCGADNVHLTETQKEKAKQYTSECVKESGVSTEVINAAKTGQYSEDKAFKKFVLCFFNKSAILNSDGTLNMDVALAKLPPGVNKSEAQSVLEQCKDKTGQDAADKAFEIFQCYYKGTKTHILF

>BmorOBP28

MLKVFIVTFFAFQLSAIARLQANGCVAVPFPKDKTIIIVEAMKSCIAKTGANPNFIDVIRSGKVSEDEKFKEFYYCTCNDTGFVNPDGHIKVKECIELFPKETQPLVEPVIKNCDKEEGVNKYDTLFKFLKCFQETSPVRVALA

>BmorOBP29

MTGPAAAAVLLALLAAAGQATTGCKNCVILGKEERAMFRSHSDACLAQSRVEPRLLESMMNGELIDDAALRKHVYCVLLSCKMIGKDGKLLKAAILGKLAARPAGRDVTKVLEACAEQPGASPEDVAWNIFRCGYNRKAVLFDYMPAGGASSGNTENHP

>BmorOBP30

MRSFVILLNYGLLCCGQFMAEDYYYDIVTRDPDDLMREKENEVRALRAFQADCAEDVQVKPDLVVNLKSGDWQTEDVSLKKWALCVLMKLGLMTAQGVFKMNEAMSKIPDMNDKIIAEKLIDDCLSLQATTPHDAAWNYIKCHHQKDPEGNFSSLNIF

>BmorOBP31

MKTFIVFVVCVVLAQALTDEQKENLKKHRADCLSETKADEQLVNKLKTGDFKTENEPLKKYALCMLIKSQLMTKDGKFKKDVALAKVPNAEDKLKVEKLIDACLANKGNSPHQTAWNYVKCYHEKDPKHALFL

>BmorOBP32

MYSHKYLNDFTNIPEILIILLSSVALMSYGYNTKLFSHSLGSEPSLSILYARDKKSDKVTNECLMEMYPKNLYKYPLRIDRNDIPCIIHCVLKKFGIISNDGFINIKNYYRRVQAIHRYDPRILISDVGETCAQNINGMNLDHDVCKKAKVFNDCTQLYAISYREPEDW

>BmorOBP33

MYAHDKLSDMIADQCLNEMYPRSKRLEIEESDEPCIIFCVLKKFGIMSPTGVINLEAYRKRVQLPEQLAQRNSINDFGSACLESAEATQHKQDVCKKAKVFNECTHLYKILLK

>BmorOBP34

MEKMILLNVFAVVLPCVLASRTRGSSGTLVDFTDPKVQGHLDALVRMAQSCVIKVRATPKDVRAYFTNSSPVSRSGQCFATCMLEQSDIINHGKVNRDLLVHLAGLVNGKNSRVVRKLNSVSRLCLDSISGMTDRCQLASTYNDCLNENMIEFAFPLDIAEEAVRKMPFHLIQPK

>BmorOBP35

GMSTHVLDFKRNMTECLKEVQNNDKRPIKRLSPKQESPIHGECLIACVLKKNGVIQNGKVNKDNLMALVSKFHAKETKLMKKLEKNLDRCINISVKNHDECSLASQLNDCTNDIMASSKQKILFNY

>BmorOBP36

MAVSEISRILTFLTIVSFIYIVYSFKPLTKDEHIERYNKMNEDIEPFRKNLTECARQVKASMADVEKFLKRIPQSNMEGKCFVACILKRNSLIKNNKLSQENLLEVNRAVYGDDSEVMSRLKTAILECSKIVEDIFEICEYASVFNDCMHMKMEHILDKITMERRMEALGQMSSNPDEWSEEEDEMLKLVKDEL

>BmorOBP37

MFYPFRFTLLFYGLFVIYLVRAEPEKENHFTLALKKTLFSTARSCMSHVNANETDLEYLRKDPPFPDKAACIIKCLLEKIGVVKNNKYSKMGFLTAVSPLVFTNKKKLDHYKSVSENCEKEINHDQTTECELGNEVVSCIFKYAPELHFKT

>BmorOBP38

MANLVLLLTFVLMTLSMARLKSTEAPKSKTALFNDQDNMGYEELDMEEIMSACNESFRIEYAYLESLNDSGSFPDETDKTPKCYIRCVLEKTEILSENGVLNPATAALVFAGERNGKPMSDLEEMAVACADRHEKCKCEKAYNFVKCLMYMEIDKYEKKN

>BmorOBP39

MVRKISALLCCFCVLGISMCDSAISTDNEQRCKNPPTAPQKIERVITLCQDEIKLSILREALDVIKEEHTMPAERKRNKREVPFTHDEKRIAGCLLQCVYRKVKAVDGFGFPTLEGLVGLYSDGVNERGYFMAVLEASRECLMKNHDKFSRTTPMDNGRNCDVSFDIFECISDRIGEYCGTSGL

>BmorOBP40

MSEFIQPSWRTQCNFRLNWDNRNRLSIDISHGAATTQTPVPTTKPKALRDFMVVPQSCDKTTCVFKKLNIVSDKGVVDVKSFIKLLDKFTNSYPVWNSAKARVITTCLRKSLIAYDGGCELNNILACTFDVLSENCPLNGNNQTC

>BmorOBP41

MLTILFLLPIVVGVLSGNIPEQPRVYCGELPNTIYSCLGNPKIIQPEVSEKCNKPISECDKTRCIFKESGWAKNNVIDKKKVSDYFEQFAKDNPDWSAAVQNFKTTCLSDSLKPQGVDTNCPAYDIIHCALISFIKFASPSQWSTSEQCVYPRQYAGACPVCPERCFAPSVPNGSCNACLALLRTP

>BmorOBP42

MMGYACVFVILAVLQAISAEDPPGLPPFLKDAPEKCKSPPRVKNPNECCISEPFFKEADFIECGIEKPGSERGPPDCSKQNCLLKKYNLLKNDETPDIEAIKSLLDKYIEKNPSFKSSVEKAKECLREDLPGPPQICLANRMTLCIGTVLLMECPDEKWNTTDDCKAFKDHMTECQKYFPK

>BmorOBP43

MKVCVLFAIFTVAQAAKATLKPISACCNIPELGNPEPLAECSNPKLPGPCKDIQCVFEKSGFLTENKTLIKEAYKTHLRQWAKEHEGWSVAVEKAISDCVDKDLRQYLEFPCSAYDVFTCTGIAMLKKCPNEHWTC

>BmorOBP44

MSRLVLFFTILVVLQEFIINLYFNFITEIDSCCVKKYPKLFDSEFITECYNTQRKANDKCERDMCVARKLNLLTEEDSINKDALLRFVEEGFKTEIDLVNAIKKKCFEEDISNIGKPEMCEVAKYKICITSRMAEDCPKWDSKGICSSAQQKVENFMKMLS

>HarmPBP1

MEFHRSTMMSVRLALVVAVCLFIRVDASQDVIKNLSMNFAKPLEDCKKEMDLPDSVTTDFYNFWKEGYEFTNRQTGCAILCLSSKLELLDQELKLHHGKAQEFAKKHGADDAMAKQLVDLIHGCAQSTPDVADDPCMKTLNVAKCFKAKIHELNWAPSMELVVGEVLAEV*

>HarmPBP2

MAASRWLFARAFCLVLMMGSAMSSKELLTKMTGGFTKVVDACKTELSVGDHIMQDMYNFWREEYQLVNRDLGCMIMCMTAKLDLIGDDQKMHHGKAEEFAKSHGADDALAKQLVGLIHGCETQHQAIEDHCSRALEIAKCFRTKIHELKWAPSMEVIMEEIMTAA*

>HarmPBP3

MGSRHVFFALVVLAVSVRKAEPSKDAMQYITSGFVKVLEECKHELNLNEQILADLFHFWKLEYSLLGRDTGCAIICMSKKLDLLDANGRMHHGNAAEFAKKHGAGDEVASKIVTIIHECEKKHEQDGDECLRVLEVAKCFRTGIHELNWQPKVEVIVSEVLTEI*

>HarmGOBP1

MPGVLRALLVLAAAAPLLADINVMKDVTLGFGQALDKCREESQLTEEKMEEFFHFWRDDFKFEHRELGCAIQCMSRHFNLLTDSSRMHHDNTEKFIQSFPNGEVLARQMVELIHSCEKQFDHEDDHCWRILHVAECFKGSCVQRGIAPSMELMMTEFIMEAEAR*

>HarmGOBP2

MTSKSCLLLVAMATLTASVMGTAEVMSHVTAHFGKALEECREESGLSAEVLEEFQHFWREDFEVVHRELGCAIICMSNKFSLLQDDSRMHHVNMHDYVKSFPNGHVLSEKLVELIHNCEKKYDTMTDDCDRVVKVAACFKVDAKAAGIAPEVAMIEAVMEKY*

>HarmOBP1

MSKFTFFVLCVVAVSLSKVYASDEDKAKLHEALKPLVEECMKDHEVSLDDLKAAKEAKSADGVKPCFLACVYKKAEVLNDKGEFDADHALEKLKEFVSDEDVLAKVAEVGNTCKAVNDKAVSDGDAGCERAALLTACFLEHKAEILV*

>HarmOBP2

MMDRKRLCLLIIAMFLAQGSDAMSRQQLKNSGKMLKKNCMNKNQVTEDQIGSIDKGKFVEDKKVMCYIACIFEMTNVVKNNKLNYDASIKQIDLMYPPDLKESAKAAVEKCKDVQKKYKDICEASYWTAKCMYDFKPEDFIFA*

>HarmOBP3

MSKFTCFVLCVLAVSLGEVRSNALEKAAIRAAVYPLIVDCAKEHAVTLEQLKAAKASHSAEGINPCFQSCVYKKTGIFNDNGEYDVANAKTKLQKFVTDEDEYARIAEVGKTCASVNDKSVSDGAAGCERAALLTACFLEHRAQIII*

>HarmOBP4

MSKLTCVVFAAVAVVFSNVNADDETRASFRQVLGPLVMECRNEFGITEDDLKKAQQERSPDALKPCFIACVFKKFGIITSAGKYDSDASISRIKDVVKNDDLLAKLKSVGEKCNSVNDASVSDGDAGCERAALLAKCFIENKSELSI*

>HarmOBP5

MSKFTCLVLCVVAASLSQAYASEEEKAAFREAIKPIVEECSKEHGVSHDELKSAKDNQNADNIKPCFLGCVYKKAEVFNSKGEYDVDKALEKLKKFVSNDEAYAKFAEVGKKCASVNDKAVSDGDAGCERGALLTACFLEHKAEVPL*

>HarmOBP6

MSKFTCLLLCVVAVSLSKVHATEEEKEAIRAAVRPIMQECGKEHGVTLDDLKAAKAAHSADGIKPCFQSCVYKKAGIFNDNGEYDIANAKTKLQKFVTNDEEYARIAEVGKMCASVNDKPVTDGAAGCDRAALLTACFLEHRAQIII*

>HarmOBP7

MFRFGVLSFVVLLFCMESSYALSSEEELSIKEALHPFVVECAEEYGMTEEMFEEAKKKGSAEDIDPCFMSCFLKKTGFFDDSGKFDAEKSISFAKEHITSESAIKFLEAGAGECVKINDEDVSDGENGCDRAKLLFDCLTELKKKMSE*

>HarmOBP8

MLLIEIVKFLTLVAMCEAMTMKQIRNTGKMMRKSCQPKNNVADEQIDPIAEGVFNEDKEVKCYMACIMKMANTIKNGKLNYEAAIKQADLLLPDDIKEPAKEAITACRKVADAYKDICDASFHITKCIYTQNPGIFYFP*

>HarmOBP9

MCKFSVLFLYSAVMAVNIWSASCISEEDKAAIITAIAPLAQNCGSECGLDNDDFEKYKEDGSDMDPCFKACLMTQMGVLDKEGKYDGKGLHKAMEEADYPGDKDDAQKFLDELDRCFDAKGDNSGSDEEAKMKRADVLFRCMQDMKEK*

>HarmOBP13

MFTGTLPLVVFLATFAYGGKEKPVFSDEIKEIIQTVHDECVAKTGVAEEDITNCENGIFKEDPKLKCYMFCLMEEASLVDDDDAVDYDMLVSLIPEEYVDRTTKMIFSCKHLDTPDKDKCQRAFEVHKCSYEKDPDLYFLF*

>HarmOBP18

MKSFVVFCVLVAGAFAANVSLPPKQNEKANQIATECMKESGLKPEVLAEAKKGHISDDEHLKKFTFCFFKKAGIVSEDGKLNTEVALAKLPPGVDKAEAEKLLETCKGKTGKDVTDTVFEIFKCYHHGTKTHILLGF*

>HarmOBP15

MGSRHVFFALVVLAVSVKKEKPSKHPMPYITSRFVKVLEECQHELKLNEHILEHLFHFWKLEYSLLGKDPGCAIICMSTKLDLLDLYGRMHRGNAAEFAKKHAAGDEVPSKIVTIIHFCQKKHEQDGDECLQVLEVATCCRTGLHDLNWQHQVEVIVPDVLTEI*

>HarmOBP16

MFKLCVVLAFIVATCHGGTLERTSSTCGQIPRELTACLDLQPAVSPEIQEKCRRANECERLTCVFREYNLLDGAEVNKERTAAFLDNFVKQYPSWEVAIDVAKTSCLRSSGLKPQGVFLDCPAYDIIQCVFANLVKNALPSQWSSMSQCNHAREFAAACPICPDACFAPLVPIGTCNACSAARRSS*

>HarmOBP17

MRAWSVTLVALLGALGAARAVAMDEDMAELARMVRENCAAETGADVALVERVNAGADLMPDDKLKCYIKCTMETAGMMADGEVDIEAVLALLPPELAEHNAPSLRACGTVRGADHCDTAFRTQQCWQNANKADYFLI*

>HarmOBP18

MTRQQLKNSGKLMKKSCMPKNDVTEEEVGDIEKGKFIESRNVMCYVACIYTMTQVVKNNKLSYEAVIKQVDMMFPAEMRDAVKAAATSCKDITKKSKDLCESAYWTAKCMYDYDAENFVFP*

>HarmOBP19

ARTEHEIKEWLFREGVACNKDFPITPDEMMMLKDNKLPDSTNAKCLIACIFKKTGMIDSKGMFDPDKSIAMTEKDFADNPEKLATSKKLMEACRGVNEQAVADGEKG

>HarmOBP20

KVFYLLTVLSACYGAVDITKYFKTCNRNAIDVNDCMADAVQKGIAVMINGIDELGIPPIDPYLQKDFRLEYKNNQIAAKLNMKNIQVEGLRAAKVHDARLRADDDKFHLEVDLTSPKVTVHAEYHGEGKFNSLRILAFGEVNTTMTDLVYTWKLDGVPEKNGTETYIRIKEFYMRPDVGSIVTNFKNDNPESRELTDLGTRFANENWRTLYREFLPYAQANWNKIGTKVANKLFLKVPYDQLFPTSS*

>HarmOBP21

FQMSRAQVKKTMSLVKNQCMPKNSVTEDQVGKIEEGVFLEDRNVMCYVACIYKNLQVVKNDKLDMSLITKQIDALYPPELKEPVKKAVSLCIHSQDNYNDLCEKVFHASKCLYEKDPASFIFP*

>HarmOBP22

MTREQIKNSGKLIKKTCMAKNDLSEDQVKDVDKGKFIEEKPFMCYIACVYKMGQTIKGNTVNHDMMIKQVEMMFPNEMKAPMKAAIEHCRPVVKKYKDVCEVSYWTAKCIYEFDPPNFMFP*
