# Supplementary material for: Identification of chemosensory genes from the antennal transcriptome of Semiothisa cinerearia
Source: PLoS One. 2020 Aug 7;15(8):e0237134. doi: 10.1371/journal.pone.0237134 (PMC7413487; doi:10.1371/journal.pone.0237134)
Supplement: S5 Table — (DOCX) [file pone.0237134.s013.docx]

>ScinCSP1

MKLLILLVLVAVAAAAPNDNHYDPKYDNFNIQELLDNRRLLCAYAKCFTGEGKCTAEGHDFKKWIPEGSKNGCAKCTEKQKKLVAKFISGIQVHCAEEWEKLNKQGDPEGKHQDELKTFLDEYSE*

>ScinCSP2

MKTIVCVCLFALVAVALARPNDEPYTTKHDSINLDEILQNKRLLLSYFKCLMVDGPCTAEGTELKANITDALQTECAKCSEKQKSGVEKVIGHLYHHEKEMWEELKAKYDPEGKYSAKYEEKFKQIQEKKA*

>ScinCSP3

MNWLILSAVLALAAFSAADQYTDRYDELNVDEIVANRRLLVPYIKCVLEQGKCTAEGKEMKAHIKDAMQTSCAKCTEKQRVKARKVVQHIKENEKDYWEQIKAKYDPGDKYKETYEAFLAADN*

>ScinCSP4

MNLLLLVTLAGVAAIAYGKPATKYTDKWDNINVDEILESNRLLKGYVDCLLDRGRCTPDGKALKDTLPDALENECSKCTEKQKTGSDKVLKHLINKRPDLWKELAVKYDPDNKYQDRYKDKIQSAKEKA*

>ScinCSP5

TKRQREGADQVMRYIIDNRPEDWLKLEQKYNEDGSYKRKYLARKRTEESNSTENLNQNEAKKESAEDDKS*

>ScinCSP6

MKIFLIFALALAACSAETYTTENDNFDITAVVADTEALRKLTNCFIDKGECDTVGAGFKEDLGEAVEQACKKCTDAQKHIFKHFIAGLKEKLPAENQAFRNKYDPQNKYFVALEEAVAKY*

>ScinCSP7

MKAIVCVCLFAVVAVTVARPNEDKYTTKYDNVDVDEILKNDRLFKPYSDCILDVGKCAPDAKELKSHVKEALENNCGKCNEKQKEAVRKVIKYLINEKEEVWKKLVEKYDPDHKYSQKYVDELKELKQA*

>ScinCSP8

MKGAVIICVLAIAAIAMARPNNGQYTSKFDGVNLDQILSNKRLLMPYIKCMLDEGRCTPDGKELKSHLKEALETDCAKCTDAQRKGSEKVIRHLINHETEAWDQLKAKYDPKGIYAKKHEAELRDAKA*

>ScinCSP9

MKVFVVLACLIVAALAADKYSSKYDNFDVDTLINNDRLLKAYVNCFLDKGRCTPEGADFKKALPEAIETTCGKCTEKQKLNIRRVIKAIQQKHAKQWDELVKKNDPTGANREKFEKFVQES*

>ScinCSP10

MQSWLLAVCALTVVVAAQQYPNRYENFNTDAIIQNERILLAYYKCVMDKGPCTKDGKNFKRVLPETLTTACARCSPKQKQVVRKMLLGIRSKSEPRFFELLDKYDPEHANREALYKFLVTGV*

>ScinCSP11

MQVSHALVLCCLAVVCAAQGQTQAQTQTQRPQVSDTALEDALNDKRFIQRQLKCALGEAPCDPIGKRLKTLAPLVLRGACPQCSPQETKQIQKTLSYVQRNFPQQWAKIVRQYAG*

>ScinCSP12

MKTILILCALLAAVACRPEESYSTKYDDFNAQELVDNVRLLKSYAKCFLEHGPCTAEGSDFKKIIPDAVKTKCSKCNLKQRQLVRVVVKAMQQKVPELWQELVKKEDPQGIYKADFELFIKATD*

>ScinCSP13

MKILLVLGCLAAVVLSEKYTSKYDNINLQEILESKRLLKAYVDCVMERGKCSPEGKELKEHLQDAIETGCEKCTETQEKGAYTVIEHLIKSELDIWREITAKFDPEGKWRKKYEERAKANGIVIPDE*

>ScinCSP14

MRAILTLCVVFVCAVVCQNANDMASMPKYDSRYDYLDVDAIFTNKRLVRNYVDCLINSNRCTPEGKALKRLLPEALRTKCIRCTERQKRTAVKIIKRLKYEYPDEWAKLSQRWDPTGDFTRYFEEFLAKENFNAIPGSGVAIPLSTVTDASPPQLPAAVPGPAPSPEPAPGSSPVPTAAPVAPVTIPSTSPRPVILNRFGGDGELMVGSPSSATGTTRPTAQTTMRISTMRPPVQTRPTAPSPPSAGASSNAIPTRFPLRPTELPPPYSTAITLIDQIGYKIIRTTELVTDLLRNTVRAVVG*

>ScinCSP15

MKTSTVLFVVVSLAFVTAKPNGGQYTSKYDNINLDDVLGNQRLLVPYIKCVLEQGKCSPEGRELRCKYQLKMSYRLNRVKIKSKYLRKCA*

>EoblCSP1

MKVLILLTLVVLASARPDNHYDKKYDNFKVDELVSNSRLLKAYALCFLGRSKCTPEGHDIKRWIDEGTETRCAKCTPKQKVLVAKFIKALMEHCPEEWELLQKRSDPEGKQTDELKKFLDEFAP

>EoblCSP2

MKSFYILFAFFAVCAAQTVAPATSTSEAYYTSDENVDIEALVSNHDAMKQYVDCFTGKVECGPEAGAAKGEFPDALSDACAKCTQVQKHTSKVFFAEFKKSFPADYEAMKKAFDAENKLFPAFDAAIANA*

>EoblCSP3

MKGIVCVCLLAMAAAALARPDGDHYTDKYDNINVDEILENSRLRDPYLSCVLDVGKCAPEAKELKSHIKEALETHCLKCTDIQKEQTKKVIKYLINNREEDWKKLCDKYDPDRKYSKKYEDELKTVKA

>EoblCSP4

MKLVYLITLAGVAAIAYGKPAQYTDKWDNINVDQILESQRLLRGYVDCLLDRGRCTPDGKALKETLPDALEHECSKCTAKQKSGSDKVVRFLVNKQPALWKELGVKYDPNNIYQQRYKDQIESVKEKA

>EoblCSP5

MLVLSLLSVFSVALTQCEEIYTNKYDGVDLEEILANERLLTGYVNCLLDLGPCTPDAKELKKNLPDAIENDCEKCTDRQRDGADEVMHHIINHRPQDWQKLELKYNEDGSYKRKYLASKNTAASDEEKSTDTQDKNDKEDSEEKDESE

>EoblCSP6

MKFFYVFALSLVVACSGETYTTENDDLNIEAVVADLPTLQAFVGCFNDKVKCDEKSGDFKKDIAEAIQQACAKCTDAQKHIFKVFLTGLKEKLPADYAAFKAKYDPENKYFGNLEKAIANA

>EoblCSP7

MSVISVVSFVLIMTVVAQEKYYDRRYDYFNIDYFVNNPRLLKKYLNCFLDQGPCTPIGRVFKQVLPEVITTACKKCTPLQKRFTYKAFNAFKTMLPDLHQDLREKYDPQNKYYDEFEKVTSSA

>EoblCSP8

MKVAVVLCILSFAAVAFSAPSGGTYTSKYDGVDLDEILNNKRLLIPYVNCVLEEGRCTADGKELKSHIKEALETDCAKCTEAQQKGAEKVFRHLINHEPEIWDKLKTKYDPTGKYYKRHEAELKA

>EoblCSP9

MKLLLIAVLITLASLHTLAQTYNDKYDGMNIDQALANKRLLKAYINCVLDVGKCTPEGKELKGNISDALQSGCVKCTGPQRAGIRKVIAHLINHEERYWNQLVDKYDPKRLYSRKYENELKAIKG

>EoblCSP10

MRVIVVFACLVASVVTADKYSSKYDNFDVDTLINNDRLLNAYVNCFLDKGRCTPEGADFKKALPEAIETTCGKCSDKQKGNIRRVIKAIQQKHAKQWDDLVKKNDPTGNHRDNFEKFIQGS

>EoblCSP11

MRSSMLLCAVALWAFVSGDDVLSDEDELLIEDEKWFQGFHDCLMKTGPCPEEIKKYTNIIPIYLKTACGPCSPKEKEIFQKHKQLVAAIYPKMFADLKAKFVTNTTKATENL

>EoblCSP12

MKWTVLAVCALTVVVSSQQYPNRYENFNTDAIIQNERILLAYYKCVMDKGPCTKDGKNFKRVLPETLTTACARCSPKQKQVVRKMLLGIRAKSEPRFFELLDKYDPEQTNREALYKFLVTGF

>EoblCSP13

MKSFCIVALGLVAACNAAYTSDAGLDVDTIVKDKEAVQKVLKCFNGGDCSSQAAALKTDFPLALGSACAECTPEQKHNTKLFIGAVEKFFPEEYKSLKTNFDPENKLFPGLLAAVAAH

>EoblCSP14

MQIKYATILSALIAVCIAQSQRPQVSDTALEDALNDKRFIQRQLKCALGEAPCDPIGKRLKTLAPLVLRGACPQCSPQETKQIQKTLSYVQRNFPQQWAKIVRQYAG

>EoblCSP15

MNALLLLAIVTLAVPLVVCYDEIYDKIDVDKIIADDALFTKYIDCMLDKGPCEVEHSADFKKLLPEVIATSCAKCSPIQKKHVRKTVKTLGEKKPAELLEFKKMYDPKGEHEKDFTAFVLAED

>EoblCSP16

MKTVLVFCVIVGAVLCAPQEHYSTQYDNFDAQELAQNPRLLKNYAKCFLSQGPCTSEGTDFKKVIPDAVKTKCEKCSDKQKELIRIVVKALQEKLPELWQELVKKEDPNGQYKAAFESFITGN

>EoblCSP17

MLKMKFFLVICCLAAAVVAEKYSDKFDNIDLQEILENKRLLKAYVDCVMERGKCSPEGKELKEHLQDAIETGCEKCTEKQEKGSYTVLEHLIKNELEIWRELTAKFDPEGKWRKKYEDRARANGITIPEE

>EoblCSP18

MKVISFYMVMMMVMVAYANPEEKYREPNDVNIEEIIKTPRLFHGYYLCLSKEGKCTPYGKELKENMPDALANGCAKCTENHMTAIRKVIKFMIENKPEEWKKLKSIYDPEGIYAAKYEKELKELQA

>EoblCSP19

MRAVITLGVCLCVCVVMCQDLNDMSQMPKYDSRYDYLDVDDIFTNKRLVRIYVDCLTSSVRCTPEGKALKRLLPEALRTKCVRCTERQKRTAVKIIKRLKYEYPDEWAKVSTRWDPTGDFTRYFEEFLAKEQFNSIPGSGVVLPLSTVTQQPPPPPAPVPAPAPAPAPSTNPPPTAAVLPLSTSPRPIILNRFGGDGELMIGSPSSAAGTTRPATPPGVKISTMRPLMQTRPTPAASSGSASNAIPTRFPLRPTTDLPLPYSTAITLIDQIGYKIIRTTELVTDLLRNTVRAVVG

>EoblCSP20

MKFAIIALCLVAAVLASDKYDELNDNFDISEVLNNPRLLNSYAKCLLNRGPCTPEVKQVKEKLPEALETRCAKCTEKQKQMGKQLAQEVKKHHPKLWSDLVALYDPEGKYQQAFQDFLASQ

>EoblCSP21

MKTATVLCVFAIVALAAARPDAGKYTDRYDNVNLDEVIGNKRLLVPYINCVLEKGRCSPDGKELKSHIKEALETYCAKCTDVQRSGTRRVIGHLINHEPGYWAQLTNKYDPARKYVVRYENELRQTQG

>SexiCSP20

MQIKYALVLCCVAAVSVAQSQRPPVSDTALDDALQDKRFIQRQLKCALGEGPCDPIGKRLKTLAPLVLRGACPQCTPQETKQIQRTLSYVQRNYPQQWAKIVRQYAG

>SexiCSP19

MKYILVALVATIAVVKAQETYGTQYDNVNGEAIVSDDQQFQSFVDCFMGAATCNEPAAAFKKVLPEAIVQACAKCNPAQKHLVRVFLEAYSKKMPQEYEKFKDLFDPERKYFPKFEASVAGF

>SexiCSP18

MKGITMICALGVLACAVASPADHYTDRFDNINIDDILNNPRLLNAYINCVLDKGKCTSEGKELKSHISDALENHCEKCTEKQRQGTRTVLAYLINNKPATWNQLTAKSIPMEICRSV

>SexiCSP16

MNALLIAVFALAAPLVLGYDEKYDKLDVDKILGDDALFTAYIDCMLDKGPCSVEHSADFRQLLPEVISTACAKCSAIQRQNVRKTVKALSEKKPDDFAQFRTKFDPKGEYEKAFSAFVIGTD

>SexiCSP14

MRAVLFLCALVHVVVGQDVNDMVNMPKYDQRYDYLDVDAIFANKRLVRNYVDCLINAVRCTPEGKALKRILPEALRTKCVRCTERQKRTAVKVIKRLKNEYPDEWSKLASRWDPTGDFTRYFEEFLAKEHYNTIPGSGSALPTSAPIAPPRVSPLPPSTTPTPGPTESTPPRPLVLNRFGDDGELMMGSPSSAGVTPRPMTQATTRPSTTTKTPSTRPIPPRPTMMTWAGAASNTQSTRFPLRPVSEISPPYSTAITLIDQIGYKIIKTTELVTDTLRNTVRAVVGR

>SexiCSP13

MKSILVLCLLVTAVSCRPETYDTRYDNFDVEALVGNVRLLTAYGHCFLGTGPCTPEGSDFKKTIPDALRTGCGKCSPKQRHLIRVVVQGFQNKTPALWQQLVKKEDPNGEYKEIFTRFLNAKD

>SexiCSP12

MKLVIILALVAIALARPDDGFYDKKYDNFNADELIENDRLLKSYAHCFLEDGKCTPEGNDFKKWIPEATTTSCGKCTDKQKVLVAKTIKAIKEKLPAEYEALVKKHDPEHKHHDDLNKFLEKYAP

>SexiCSP11

MRVLVVLSCLVVVAFAADKYNPKYDNFDVDTLISNDRLLKAYINCFLEKGRCTPEGSDFKKALPEAIETTCAKCTDKQKGNIRKVIKAIQQKHPKEWEDLVKKNDPSGKHRGNFDKFIQGSS

>SexiCSP10

MRSWLLCLCVLTVVVSCYSQANRYENFNPDAIVQNDRILLAYYKCVMDKGPCTRDGKNFKRVLPETLATACGRCNPKQKTIVRKLLLGIRSKSEPRFLELLDKYNPDRSNRDALYAFLVTGA

>SexiCSP8

MQIVVVLVVACVGLVAGLHVQAGPQMTDAQLEQTLADKSTMQRHIKCALGEGPCDPVGRRLRTLAPLVLRGACPQCSMQETRQIRRTLAFVQRNYPWEWAKIVRQYG

>SexiCSP7

MKFVLVLCLMAAAVLADDEKYTSKYDNIDLDEILTNKRLLTAYVNCIMERGKCSPEGKELKEHLVDAIETGCTKCTENQEKGAYKVIEHLIKNELDIWRELTGKYDPSGKWRKTYEDRAKANGIIIPE

>SexiCSP6

MKLIVVVALCLVAVAWAKPASTYTDKWDNINVDEILESQRLLKAYVDCLLDRGRCTPDGKALKETLPDALEHECSKCTEKQKKSSDKVIRHLVNKRPDLWQELSGKYDPENIYQERYKNQLDAVKRQ

>SexiCSP4

MKCIYVLSVLLAFAAVQAEDKYSTENDDLDIEAVVADLDTLKGFVGCFMDAMTCHAVAADFKKDIPDAVATSCAKCTNAQKHIFHKFLLGLKEKLPSDYEAFKKKFDPQGQYFEALEAAVASS

>SexiCSP5

MIGLNKYNVPVSIILIFLFVSTVLSQEKFYDRRYDYYEIDTLIQNPRLLKKYLDCFLGKGPCTPIGRVFRQILPEAVQTACKKCTPSQRRLARKTFNAFKGYFPETHEELRKKLDPKNKYYEAFEKAISSA

>SexiCSP3

MKVVFLVFVLTAVVYSHPHDSHYTDKYDNIDLDEILNNKKILTSYINCCLDLGKCTPDGKELKSHIREALENKCGKCTEAQKNGTRKVMTHLINFEPDYWNQLCAKYDPEGKYKAMYEKEYKTLVH

>SexiCSP2

MKSMIVLCVLSVAALVVARPDDSHYTDRYDNVDLDEILSNRRLLVPYIKCILDQGKCAPDAKELKEHIREALENECGKCTETQKKGTRRVIEYLINNEEEYWNELTAKYDPERKYTTKYEKELKKIKA

>SexiCSP1

MKSFIVLCLFGLAAVAMARPDGSTYTDRYDNINLDEILGNRRLLTPYIKCILEEGKCTPDGKELKSHIREALEQNCAKCTDAQRNGTRRVLGHLINNEEESWNRLKAKYDPQSKYTVKYELELRKLKQ

>PxylCSP1

MKSAAFIALFLIGKAVCEDKPTYTTKYDNIDLDEILSSERLLTGYVNCLLDQGPCTPDGKELKHTLPDAI

DNDCRKCTQKQKEGSDRVMGYIIEYRPNDWAKLEKKYLSDGSYKKKYLEKKNASENNGDSKSTEAKNKDD

EEKKSKGDGEEK

>PxylCSP2

MQKLTLACLLVAVAAAAARPNDSHYTDRYDNVNLDELISNRRLLVPYVKCVLDQGKCSPDGKELKEHIQE

ALENNCGKCTDKQREGTRKMIGHLINHEQEFWDQLIAKYDPERKYVSKYEKELKEVKASCCWLV

>PxylCSP3

MNSLVLVCLALVAVAAARPQATYTSKYDGVNVDEILANDRLMMPYIKCALDHGRCSPEAKELKSHIKEAL

ENNCAKCTDKQKPAVRKVIAHLINHKPAEWRQLSDKYDPAGKYTAQYEDQLRAVKA

>PxylCSP4

MQTVTLLCLLAAVAAAAAAPADTYDAKYDSFNAHELVQNQRLLKSYGKCFLSKGPCTAEGSDFKRVIPEA

LKTTCGKCTRKQRELVRVVVKGFQEQLPQVWTEIVSKEDPKGEYKDSFAKFLEGSD

>PxylCSP5

MKVVFLVFVLTAVVYSHPHDSHYTDKYDNIDLDEILNNKKILTSYINCCLDLGKCTPDGKELKSHIREAL

ENKCGKCTEAQKNGTRKVMTHLINFEPDYWNQLCAKYDPEGKYKAMYEKEYKTLVH

>OfurCSP19

MKTLLFAITLAALACCARAQVYTDRYDTVNLDDVLANKRLTVAYIKCMLDKGGCTSEGRELKSHIAEALQ

NGCAKCTKAQREGMRRVIKHLIQHEKGYWQELVEKYDPKRVYTQKYENELNSL

>OfurCSP18

MQKLIILALVCTMGWSVVVAAPQMTDAQLDQTLTDRATMQRHLRCALQEGPCDPVGKRLRILAPLVLRGT

CRQCTPQETRQIRYTLAFVQRNYPWEWAKLIRQYG

>OfurCSP17

MQTTLVLLLVVAACAYAAEAPRPQVTDTALEDALNDKRFIQRQLKCALGEAPCDPIGKRLKTLAPLVLRG

ACPQCSPQETKQIQRTLSYVQRNYPQQWAKIVRQYAG

>OfurCSP16

MSHRKVLVLSHLMVFLCVQCFAKLHNYDNFDMETLLLNTTRSRALFECVRDETKCANKEDKEMKDDIFEM

VTTSCANCTAKEKQKFGDAMKALHRSMGESQIITMFINKMTNMFQGGLSDTEKTT

>OfurCSP15

MRAVLLLCACAAAVCGQNLDSNRMARMPKYDERYDYLDVDALFNSKRLVRNYVDCLINAQRCTPEGKQLK

RILPEALRTKCIRCTERQKKTAVKVIKRLKYEFPEEWAKLSSRWDPTGDFTRYFEEFLANESFNTISGSA

DGNDAAGPSSIPPLPPVPPRLPAAPPSTPPPLPVEPVSTSPKPVILNRFGDDGELMMGSPSSAALTPRPS

TARPPLS

>OfurCSP14

MWIQLAILATFVSIVITEMGPPGIERTFSDGVTSRGYRVVYGDEDLTVINEVVGNMEKNDILKAKASLNE

AIQPLPAGDVKCLMSADRYCSVEMRKVKGVLIQALKNDCEKCSNTEKDTAGRVAASMMTYDPVGWKLFLT

RYDGLSKIQRILG

>OfurCSP13

MRAVVFLSCLVVVLAADKYNSKYDNFDVETLISNDRLLKAYINCFLEKGRCTPEGADFRKALPEAVETTC

AKCTEKQKNNIRKVIRAIQQKHPKQWEELVKKTDPSGKHRAGFDKFIQSN

>OfurCSP12

MKFLVVLSAVLAVALARPDSYKTDHDGLDIEGIVNNPEALAKVTACFLEKAPCTPIAAEFKSVLPDATET

ACSKCTAAQKHMLKLYLLKVRETAPDDLKALKTKYDPDSKHIDALIAAIKDA

>OfurCSP11

MRHIIILLAVVALVTQSFADEETEKKEEKKEEKKEEQTDEKKYTDRFDDINFEEIIANRRLLVPYLKCVL

DKGRCTPEGKELKAHVKDAMQTACEKCTDKQKTGARKVVNHIRDNEKEYWEELINKYDPKGEFKSIYEPF

LAAKE

>OfurCSP10

MKTIMLVAFLVGLAMADEKYTSENDNFDVEALVNNTEELQKFSGCFLDKNDCDAVSGDFKKDIPEAFQQA

CAKCTDAQKHLFKRFLNGLKEKLPQDFEAFKKKYDPEDKFFAALDKAINA

>OfurCSP9

MKFLVLSAVLALALADSYKSDYDSLDIAPIVNDPEALSKLTACFLDKGPCTPIAADFKTYLPDATETACS

KCNTAQKQKLKLYLQKVKETSPDDLAALKAKYDPDSKHVDALIAALKE

>OfurCSP8

MKLVAFIPTFTYLLLGANAEESPTYTTKYDGVNLDEILENDRLLTSYVNCLLETGPCTPDGKELKNNLPD

AIQNDCKKCSERQREGADQVMEYIIDHRPDDWEKLEKKYNSDGSYKKKYLERKEARNQSNSAEKSQENDS

KSKE

>OfurCSP7

MKTFAICLLALVAVVSAYPQAKYTDRYDSINLDEIVGNRRLLVPYIKCILDQGKCSPEGKELKSHIKEAL

ENYCAKCTETQRDGTRKVIGHLINNESEYWNQLTAKYDPQRKYVVKYEKELRTVS

>OfurCSP6

MKLVIISLCLAAAVVAQEKYDSIDDNFDISEVLNNERLLNSYTKCLLDKGPCTPEVKKVKDKLPEALATR

CAKCTDKQKQIGKQLAKEVKAKRPDLWKELVAHYDPEGKYQEAFQDYLKP

>OfurCSP5

MHPQHFCMIVMVTTAAADFYSAKYDDFDIQPLLENDRILQGYTKCFLDQGPCTPDAKDFKKVIPEALETS

CGKCTPKQKILIKKVIRAVMERHPDSWKELEDKFDKDKKFRDSFNKFLEEKD

>OfurCSP4

MKTFVLLALSLVVAVAYARPGAQYTDKWDHINVDEILESQRLLRGYVDCLLDKGRCTPDGKALKETLPDA

LEHDCSKCTEKQKASSDKVIRHLINKQPDYWKELSAKYDPNNIYQDKYKDKIEEVKSKN

>OfurCSP3

MKTFILICLSALVMVSSADKLDDLLNTDMEKLLADDAVRKQVVGCMTDELPCGDYQAYKDMLPDLIATNC

GKCTPEQKKRYEEINKFVLEKYPNEYNAVVSKYRPKTE

>OfurCSP2

MKTIVALCALVAVALARPEDTYSTAFDSFNAQELVDNIRLLKNYGKCFLDQGPCTPEGSDFKKKIPEALK

TDCGKCTPKQRELIKTVVHGFQSKLPDMWAELVKKHDPEGQYTESFDAFLNSK

>OfurCSP1

MISTKYLIVLCCVAAAVARPSDKYTDKYDNLNIQEILENKRLLKAYVDCVMGQGKCSPDGKELKEHLQEA

IETGCAKCTEAQEKGAYTAIEYLIKNELDIWKQLAAKFDPEGKWRKTYEDRARANGIVIPE

>HarmCSP

MKVLLVLCLFAAAALADDKYTDKYDNINLDEILENKRLLLAYVNCVMERGKCSPEGKELKEHLQDAIETG

CSKCTEAQEKGAYKVIEHLIKNELDIWRELAAKYDPKGDWRKKYEDRARANGIQIPE

>HarmCSP2

MKVVLLTLCFALGVLAQDQYESANDNFDISEVIGNDRLLHAYANCLLNKGPCTPEVKQVKEKLPEALETR

CAKCTDKQKQMGKALAQEVKKNHPDIWKQLVAMYDPQGKYQQAWKDFLQE

>HarmCSP3

MNADWFLIFTLITVVSSDFYNSKYNCFNVQPLLENDRILLSYTKCFLDQGPCTPDAKDFNKVIPEALETT

CGKCSPKQKLVIKTVIKAVISRHPDAWDQLTEKYDKDKKYKDSFDKFLA

>HarmCSP4

MNSLIVFCVLSLAALTIARPDGATYTDKYDNVDLDEILGNRRLMVPYIKCMLDQGKCAPDAKELKEHIKE

ALENECGKCTEAQKKGTRRVIGHLINHEADFWNELTAKYDPERKYTTKYEKELKEVKA

>HarmCSP5

MRTFVVVCLLGLVAVTLARPESKYTSKYDNINLDEILANQRLLVPYLKCILEEGKCTPEGKELKSHIREA

LEEDCAKCTENQRKGTRKVLAHLINHEEGYWNRLKAKYDPESKYTAKHEQELRELKH

>HarmCSP6

MKADCFLFVTLIAVVAADFYNSKYDSFDVQPLLENDRILLSYTKCFLDQGPCTPDAKDFKKVIPEALETT

CGKCSPKQKQLIKTVIKAVISRHPDAWDQLTEKYDKDQKYKESFDKFLAEQD

>HarmCSP7

MKVFVVLSVLIAFTAAASLTPAELDLAEAFDYEALFSNDEQRKLVFDCILGKGECGDYQKMAEISRKVLE

SKCADCNPKQKAKYETVLKTIQTKYEPFYNELLKNVAAKKE

>BmorCSP14 BGIBMGA004068 Bmb037503 DQ855516

MKILIIVVMACVAVTWARPESTYTDKWDNINVDEILESNRLLKGYVDCLLGKGRCTPDGKALKETLPDALEHECVKCTGKQKSGADKVIRHLVNKRPDLWKELAVKYDPDNIYQARYKDKIDAVKGSA

>BmorCSP1 BGIBMGA004045 Bmb008613 DQ855510

MKVLIVLSCVLVAVLADDKYTDKYDKINLQEILENKRLLESYMDCVLGKGKCTPEGKELKDHLQEALETGCEKCTEAQEKGAETSIDYLIKNELEIWKELTAHFDPDGKWRKKYEDRAKAKGIVIPE

>BmorCSP8 BGIBMGA004066 Bmb018111 DQ855509

MNSLIAFCLFAVLAVALARPDDKYTDRYDNVNLDEVLSNSRLLQPYIKCILDKDRCAPDAKELKEHIREALETECAKCTEAQKKGTRRVIGHLINNESKSWNELTAKYDPENKFTAKYEKELREIKA

>BmorCSP9 BGIBMGA004065 Bmb018112 DQ855511

MKTVIVCLLALTAVALARPEQYTDKYDTVDLDQLISNRRLLIPYVHCILEKGQCTAEGKELKSHIKEALETNCAKCTKAQKGGTEKMIGHLINHEAEFWEELKAKYDPTNEFTKKYETELKRVTA

>BmorCSP16_N BGIBMGA001840 Bmb037911

AHITDALQTGCTKCTGAQRKGIRRVIKHLIDSEPGYWDRLVDMYDPKRVYTGKYEKELRTIKA

>BmorCSP6 BGIBMGA004046 Bmb018109 DQ855507

MKCLTIAALLFVAGLSIAEKYTDKYDNIDVDEILENRKLLVPYIKCVLDEGRCTPDGKELKAHIKDGMQTACAKCTDKQKVSARKIVKHIKQHEADYWEQMKAKYDPKDEFKEIYEGFLAGQN

>BmorCSP4 BGIBMGA004047 Bmb005346 DQ855518

MFMLFIISFIIVPVLKCCGTETSTYTTQYDEVDIKEIMGNERLLVAYIGCLLDKNPCTPEGKELKRNIPDALQSDCSKCSDKQRENADAWIEFMIDNRPEDWTKLEERYNPDGSYRTKYLEGKHNATSNVDESK

>BmorCSP7 BGIBMGA004067 Bmb018110 DQ855508

MKSVILICFLGVATVVIARPKTPFDNINIEEIFENRRLLLGYINCILERGNCTRAGKDLKSSLKNVLEENCDKCSEDQRKSIIKVINYLVSSEPESWNQLKSKYDPEGKYLIKYEAKMESN

>BmorCSP2 Bmb030868 DQ855519

MKLLLVFLGLFLAVLAQDKYEPIDDSFDASEVLSNERLLKSYTKCLLNQGPCTAELKKIKDKIPEALETHCAKCTDKQKQMAKQLAQGIKKTHPELWDEFITFYDPQGKYQTSFKDFLES

>BmorCSP3 BGIBMGA004042 Bmb040763 DQ855512

MKSLIVLSCLLAACLAADLSKYENFDVEPIVTSDRLLKAYINCFLDKGRCTPEASDFKKALPDTIATNCGKCTEKQKANVRKVIKVIQQKHSTEWEKLVKKHDPSGKHRADFDKFLLGS

>BmorCSP15 BGIBMGA004044 Bmb037504 DQ855517

MKLTSFLLVGMAMVSAEFYSSRYDDFDVKPLVENDRILQSYTNCFLDKGPCTPDAKEFKKVIPEALETTCGKCSPKQKQLIKTVIKAVIERHPEAWEELVNKYDKDRKFRPSFDKFINEDD

>BmorCSP11 BGIBMGA004040 Bmb028718 DQ855514

MKTILILCALVSVVVCRPEEYYSSQYDNFDVEQLVGNLRLLKNYAKCFLDQGPCTAEGTEFKKRIPEALRTKCAKCNPKQRHLIRTVVKAFQTKLPDLWEELAIKEDPKGQYKHEFTAFINAMD

>BmorCSP13 BGIBMGA004035 Bmb030714

MKFVLALIALAVVVAARPNDDLFYDKKYDNFNVDEIIDNPRLLKAYTFCFNDKG

KCTAEGNDFKKWIPESLQTSCGKCSEKQKYLVAKFVHAIKDKMPDEFDILRKLHDPKGEYTENLDKFLETYGH

>BmorCSP17 BGIBMGA004043 Bmb030869_INC DQ855520

MKSSLFCVLVLTVVVSSSRQQSYPRNDNININAILQNDRILLGYFKCVMDRGPCTKDGKTFKRALPEALPTACARCSNKQKAAFRTLLLAIRARSEPSFLELLDKYDPSRSNRELLYTFLATGL

>BmorCSP10 Bmb022146 DQ855515

MRAVIFLYTCVFVVVGQDINAMMSMPKYDERYDYLDVDDIFRNKRLVRNYVDCLINAQRCTPEGKALKRILPEALRTKCIRCTERQKRTSVKVIRRLKNEYPEEWAKLASRWDPTGDFTRYFEDYLAKEHFNTIPGSGL

>BmorCSP18 BGIBMGA004070 Bmb040762

MNNLLIAILALTLPFSIWCYDEKYDKIDVDKILSDDKLFTDYINCMLDKGPCEVEYSSEFKELLPEVIATSCAKCTPIQKTGLRKTVKALSVKRPDDFSQFRAKYDPKGEYEKQFAAFVVATD

>BmorCSP22_N (JK did not find from genbank)

MNNLLIAILALTLPFSIWCYDEKYDKIDVDKILSDDKLFTDYINCMLDKGPCEVEYSSEFK

>BmorCSP5_N BGIBMGA007272 Bmb007542

MKFLVTAVLLSLAIAIQAGSYSDRYDNINVAEILGNKRLLTAYIKCVLEEGKCTAEGKELK

>BmorCSP12 BGIBMGA004041 Bmb028717 DQ855513

MKGFYVLCFALFAAVYCKETYSSENDDLDIEALVGNIDSLKAFIGCFLETSPCDAVSGDFKKDIPEAVAEACGKCTPAQKHLFKRFLEVVKDKLPQEYEAFKTKYDPQGKHFDALLSAVANS

>BmorCSP19 DQ855521

MIENFYSKCTISKSVLFLCLIFLPYALNQKYYDSRYDYYDIDHLVQNPRLLKKYLDCFLGKGPCTPIGRLFKQVMPEVITTACAKCTPTQKRFARKTFNAFRRYFPETLMELRRKFDPESKYYDAFEKVITNA

>BmorCSP20 BGIBMGA001469 DQ855522

MIEWKRFKILHFLSYLGLLVLVVVCAAQQNRPQVTDTALDEALNDKRFIQRQLKCALGEAPCDPIGKRLKTLAPLVLRGACPQCSPQETKQIQKTLSYVQRNFPQHWAKLVRQYAG

>BmorCSP21

MPPNLKNIVFIGVCTCLVLTVLAAPQMSDAQLEKTLADKGTMQRHLRCALGEGPCDMVGRRLRTLAPFVLRGACPQCSVQESRHIRRTLAYIQRNYPWEWARIVRQYG

>SinvCSP4

MKGYFLVVLVSLVVLAVADEKYTRKYDDVNVDKILQNNRVLTNYIRCLMDEGPCTAEGRELRKTVPDALSSGCDKCNDKQKAMTEKVIDHLKTKRSRDWDRLVAKYDPNGEYKKRYEKS

>SinvCSP2

MKVLALFLLVVAIALAEEKYSTKYDNIDLDTILKSDRLLKNYVNCLLDKGNCTPDGKELRETLPDALMTECKKCSEKQKEGTEKVIRYLVNKKPETWEQLKKKYDPNGQYTAKYLDEAHKQGINVZ

>SinvCSP3

MKLTVFCLLAVISVVYVYAEEKYTSKYDNIDIDQILQNDRLLKRYVDCFLEKPNVRCPAEALEAKAHIQEALDDECAKCSDHQKEMSKKVIRHLITNKRDMWNELKAKYDPDGKYAKKYEDEAKKEGVEI

>SinvCSP1

MRHLVVTLITVYILSFSCVFAQEGTYTTKFDNVDVDAIISNDRLLNGYVGCLLDRNPCTPDAAELKKNLPDALEHDCAGCSETQKNAADKISHHLIDNKPDDWKLLEDKYDPTGTYRRRYLESRSKEGGSVD

>SinvCSP6

MSLTFVLLFSLAFSGLVSGIEYFSDNIDVDAIINSDRLLNQYVNCILDKGPCTADGRSLKHFLPDAIATTCEKCSEKQKQTARKIIKYLKEHKPNIWAEFLERYDPDEEHVAFYKEFLAQGGA

>SinvCSP5

MLQILLVLLCALLAVAMATESSTDNVEGQQTGRSRVSDEQLNIALSDKRYLNRQLKCALGEAPCDPVGRRLKSLVPLVLRGSCPQCSPEEIRQIKKVLSHIQRSFPKEWNRIVQQYGAS

>SinvCSP7

MDRLNFYLLAILAVLATIVAQETYSDMFDHINPDEILPNDELRNQYYNCFMDRGPCVTDDQKYFRQNIAEAFVTKCQKCTETQMKNYGKIVEWYTENRPDEWQAMVEKLLEEAKKLNITPA

>SinvCSP8

MARLSCIVTVIGIALMCVAAQDLYSDKFDHIDVASIVTNDKLRNEYYSCIMDTSPCKTADAKFLKEIFAEALNNDCKKCTEKQKEHMKTIQDWYTTNKPDEWQAAVAKAEDLKKNAR

>SinvCSP10

MARLSCIVTIIGIALMCVVAQEDLYSDKFDGIDVKSIITNNRLRNEYYDCFMGISPCVTADAKFFKDIFFDALGNKCKRCTEKQKEYMKIIQDWYTTNNPDKWQAAVAKSEDLKKKNARKZ

>SinvCSP9

MARLSCIITIIGIALMCVATQEDLYSDKFDGVDVPGIITNDRLRREYYNCFMGTSSCVTADAKFFKEIFFDALGSKCKRCTEKQKENMNFIVDWYTTNKPDEWQTLVAKSIEDLKKKNARKZ

>SinvCSP11

MARLSCIVTIIGITLMCVIAQEDLYTDKFDNVDVPGIIANDKLRNEYYGCFMGSSPCITADAKFLKEVFSDALNNNCKRCTEKQKEHMDYIVDWYTKNKPDEWQAIVVKSIEDLKKKNA

>SinvCSP12

MARLSCIVTIIGIALMCVAAQEDLYSDKFDGVDVASIIVNDKLRNEYYGCFMETSPCITADAKFFKGVFADALNNKCKRCTEKQKEHMDYIVDWYTKNKPDEWQALVVKSIEDLKKKNARK

>SinvCSP18

MARLSFIVTIIAVALACVLAEEELYSNRYDDIDIDRILENKKLRLQYYNCFMDTEPCRTADAKFFHEVISEAMQTQCRRCTEKQKVLLNRMADWYTQNAPEQWEAFIRKTLEDTLQKKG

>SinvCSP14

MARLSNIVLIIAVNVLICVLAKEELYSEQYDHLDVRGVLANNIQRKSYYNCFMGITPCTSEXXXFPDLFSEAYQTKCRKCTKKQIEHLNVISDWYTTHQPLKWLQLIQKMIN

>SinvCSP13

MARLNRIALLVVATSVLMCILAEELELYPSELDDIDVAKILENDAERKGELNCYLKREPCAEEFNKYTEIFREAVRTNCKRCTEKQKEHLETITNWYKKNQPDNWELILENVNL

>SinvCSP16

MARLNCIALFIVATSVLMCILAEELQPYPSEYDIYVPKILANDVVRQKAVDCYLKKGPCTEQEKLATDLFRDALKTNCKKCGEKQKEHVKILTEWFVKNQPDTWKLIIENVDS

>SinvCSP15

MARLNCIALLIVATSVLMCVLAEDLHSELDDLDIPKILANDAERQGVIDCILENASCTELETKAAAAIKDALKTNCQACGDKRKENMKIITDWFNQNQPDTWTLVVAKVNS

>SinvCSP19

MARLNYIALIVVAMSALMCVFAGDLGLYPSELDDLDVVALLADAAWRQQSDDCFLNKGPCSEEQKYLNDLFREAVRTDCERCTDKQRQIMNTITEWYEQNEADVWKIILEDARAZ

>SinvCSP20

MTRLNSIALIIVAMNVLMCVLGEELELYPPELDELDVPQLLADDAWRGNIEDCYFKRAPCTEEQKYLEDKFRYALNTNCKRCTETRKKCMKTVTEWYEKNQPDTWKLVLENVDSZ

>SinvCSP21

MARLSSIALIIVAMNVLMCVFGEELELYPREIDDIDVLKILSDDAWRRRAEDCYFKRVPCAKEKQYLSDIFKDMLKTKCEKCTEKQKKLVKTATEWYEQNEPDTWKLILEDAHSZ

>SinvCSP17

MAQLNRIALIFIAMSVLTCVLAEELWFYSGEFDDMDVLSILEAQAEQEVDCYMKRGPCTLEQQRIADSIREAIRTNCRRCTPKQKQQIQLITDWYKSRMPQNWELIVANVDL

>HvirCSP2 gi|21898574|gb|AAM77040.1| chemosensory protein 2 [Heliothis virescens]

MKFIVAVALLCLVAESWAASTYTDKWDNINVDEILESQRLLKAYVDCLLDRGRCTPDGKALKETLPDALENECSKCTEKQKAGSDKVIRYLVNKRQDLWKELSAKYDPNNIYQDRYKDKIEAVKGQ

>HvirCSP3 gi|21898558|gb|AAM77042.1| chemosensory protein 3 [Heliothis virescens]

TDKYVNINLDEILENKRLLLAYVNCVMERGKCSPEGKELKEHLQDAIETGCSKCTEAQEKGAYKVIEHLIKNELDIWRELTAKYDPKGDWRKYEDRARANGIQIPE

>HvirCSP1 gi|21898556|gb|AAM77041.1| chemosensory protein 1 [Heliothis virescens]

MALARPDGAAYTDKYDNVDLDEILSNRRLLVPYVKCILDQGKCAPDAKELKEHIIEALENECGKCTEAQKKGTRRVIGHLINNEADYWNELTAKFDPEKKYVQKYEKELKEVKA

>DmelCSP1 dmelPhk3 DmelPhk3 CG9358 FBgn0035089 NP_611990

MKASLALVFCVCVGLAAAAPEKTYTNKYDSVNVDEVLGNNRVLGNYLKCLMDKGPCTAEGRELKRLLPDALHSDCSKCTEVQRKNSQKVINYLRANKAGEWKLLLNKYDPQGIYRAKHEGH

>DmelCSP2 dmelPebIII DmelPhk2 CG11390 FBgn0011695 NP_524966

MKMILALVVLGLVLVAAEDKYTTKYDNIDVDEILKSDRLFGNYFKCLVDNGKCTPEGRELKKSLPDALKTECSKCSEKQRQNTDKVIRYIIENKPEEWKQLQAKYDPDEIYIKRYRATAEASGIKV

>DmelCSP3 dmela10 DmelOSD CG6642 FBgn0011293 AAF49381

MGQPGFRRAIGHVSLVVALMCTTCFQVEGLPHPPATSPSPMMERMVEQAYDDKFDNVDLDEILNQERLLINYIKCLEGTGPCTPDAKMLKEILPDAIQTDCTKCTEKQRYGAEKVTRHLIDNRPTDWER

LEKIYDPEGTYRIKYQEMKSKANEEP

>DmelCSP4 dmelCG30172 FBgn0050172 NP_726402

MLLLNKNRVISLVVNFIFLIILISSSVQADERNINKLLNNQVVVSRQIMCILGKSECDQLGLQLKAALPEVITRKCRNCSPQQAQKAQKLTTFLQTRYPDVWAMLLRKYDSA

>MbraCSPA6 gi|7960304|gb|AAF71289.1|AF255918_1 chemosensory protein [Mamestra brassicae]

MKFVLLLCVMVAAVVAEDKYTDKYDNINLDEILANKRLLVAYVNCVMERGKCSPEGKELKEHLQDAIENGCKKCTENQEKGAYRVIEHLIKNEIEIWRELTAKYDPTGNWRKKYEDRAKAAGIVIPEE

>MbraCSPB2 gi|6631019|gb|AAF19653.1|AF211183_1 chemosensory protein [Mamestra brassicae]

EEAHYTDRYDSVDLDEILGNRRLLVPYVKCILDQGKCAPDGKELKEHIKEALENECGKCTDAQKKGTRRVIAHLINHEEDFWNELTAKFDPERKFTAKYEKELKDIKE

>MbraCSPB3 gi|26007526|gb|AAF71290.2|AF255919_1 chemosensory protein [Mamestra brassicae]

MKSCIVLCVLSVAVMALARPEEAHYTDRYDSVDLDEILGNRRLMVPYIKCILDQGKCAPDAKELKEHIREALENECGKCTETQKNGTRRVIGHLINHEDAYWKELTAKYDPQSKFTAKYEKELKEIKH

>TcasCSP7 GLEAN_14534 DQ855493

MKLISAVILCAFLVAVSAAENKYTNKYDNVDVDKILNNDRVLTNYIKCLMDEGPCTSEGRELKKTLPDALSSGCTKCNQKQKETAEKVIRHLTQKRARDWERLSKKYDPQGQYKKRYEEHVATSRAA

>TcasCSP10 GLEAN_08682 DQ855496

MKTFVLVAFAAVLGLALARPQEKYTTKYDNIDLEEILKSDRLLKNYFNCLMERGTCSPDGEELKKALPDALHSGCSKCTEKQKEGSRKIIHYLIDNKRDWWNELEAKYDKDGVYRQKYKDVIEKEGIKL

>TcasCSP12 GLEAN_08680 DQ855498

MKTLVLVLFVAVLSVVFAADKYTTKYDNIDLNQILKSDRLLKNYVNCLLDRGKCSPDGQELKNNLADALQTSCSKCSQRQKDGSRTIIRYLIKNKRDWWNELEAKYDPTGIYKNKYADELKAEGIVL

>TcasCSP11 GLEAN_08681 DQ855497

MKTLVPLLFFVIAIASSLAENSKYTTKYDNVDLDEIIKSDRLLKNYVNCLLEKGKCTPDGAELKRHLPDALHTECSKCSETQKNGSKKIMRHLIDHKRDWWNELEEKYDKEGEYRKKYEAEIKGKKD

>TcasCSP19 GLEAN_15950 DQ855505

MKFFIAFLMLLGAVWCEQYTTKYDNINVDEILASERLLKNYFNCIMDRGACTPDADELKRVLPDALKSDCAKCSEKQKEMTKKVIHFLSHNKQQMWKELTAKYDPDGIYFEKYKDKFDS

>TcasCSP20 GLEAN_15902 DQ855506

MRFFVIFFVACVSVALARPEDQYTIKYDNVNLKEILQSDRLTENYVNCLLEKKPCTPDGEELKRVLPDALKTSCAKCTDKQKQGAKTVIQHLYKNKQDWWKQLEAKYDPEHTYVKAHEDELKAL

>TcasCSP9 GLEAN_08677 DQ855502

MTAIVFLLALACLKTYVSSQEYLVPQNIDVDEILKNDRLTRNYLDCVLGKGKCTPEGEELKKDIPEALQNGCAKCNEKHKEGVRKVIHHLIENKPNWWQELESKFDPQGEYKKKYDELLKKEGLAN

>TcasCSP14 GLEAN_08678 DQ855500

MFATSALFAFICIQGLVSAEEYLVPQNIDLDEILKNDRLTRNYIDCILGKGKCTPEGEELKRDIPEALQNECAKCNEKHKEGVRKVLHHLIKNKPNWWQELEAKFDPKGEYKQKYNKLLEKEGLQA

>TcasCSP17 GLEAN_08676 DQ855503

MFKVLFVVFACVQAYVYAEEYTVPQNIDIDEILKNDRLTKNYLDCILEKGKCTPEGEELKKDIPDALQNECAKCNEKHKEGVRKVIRHLIKNKPSWWQELQEKYDPKGEYKSRYNHFLEEEGLN

>TcasCSP13 GLEAN_08679 DQ855499

MFLAIVLVVCACTNVLSEEYTNQYNDELDAALKSERLMKSYFECLLGTGKCTPSGEELKKDIPDALKNECAKCNDKHKEGIRKVIHYLVKQKPEWWEQLQKKFDPQGIYKKRYQNYLDKEGLKA

>TcasCSP18 GLEAN_08674 DQ855504

MLFTVFLVLTCAHVVFLEEYVIPDNIDIDDILSNERLLKNYVNCLLDKGRCTPEGKKLKSTIPEALSTDCAKCNEKVKANVRKVLHHLIDNKPDMWKQLEAKYDPSGEYRSKYKDELEKNGIHV

>TcasCSP4 GLEAN_14531 DQ855490

MYSYLIPLYLFLFVHYGWSEDTTHKYTTKYDNIDLENVVKNERLLKSYVDCLLEKGRCSPDGLELKKNMPDAIETDCSKCSEKQKEGSDFIMRYLIDNKPDYWKALEAKYDPDGTYKKRYFESQKDEVSKVEA

>TcasCSP5 GLEAN_14532 DQ855491

MKTFVILFFGVFFIIFSDFVNGKTLHRSTRDDKYTTRYDNVDVDRILHSKRLLLNYINCLLEKGPCSPEGRELKKILPDALVTNCSKCSEVQKKQAGKILTFVLLNYRNEWNQLVAKYDPDGIYRKQYEIDDDYDYSELDSAKK

>TcasCSP2 GLEAN_03085 DQ855489

MKIIILAVLIATAVAATYDVYPTKYDNVDIDAILHNKRLFDNYLQCLLKKGKCNEEAAILRDVIPDALITGCRKCNDHQKVSVEKVIRFLIKERNSDWQQLISVYDPKGEYQTQYAHYLEKI

>TcasCSP15 DQ855501

MIFKIHFLVFGALLTYVSSVEYLILREIDTILKNDQMTRNYLDCVLDKGKCTKEAEKLKKGITETMKNGCVKCEQKQKEDVHKVFQHLMIHRPNWWHELETKFNPHHEIKLQHLHQSKFNPHEEVKLQHLHQFPHHDFLEREGFIR

>TcasCSP8 GLEAN_14535 DQ855494

MPLVKSLVVVVLLIGVVYQVQGQLGLAGNNYIEKQLLCALDKAPCDALGNQIKGALPEIIGKNCERCDSRQVANARRIARYVQTKHPDVWNALVKKYSV

>TcasCSP1 GLEAN_04430 DQ855488

MLILQIAHLCAQFCLLAAIFTCVKPQLTRISDEAIESTLNDRRYLLRQLKCATGEAPCDPVGRRLKSLAPLVLRGSCPQCTPQEMKQIQKVLAFVQKNYPKEWNKILHQYAG

>TcasCSP3 GLEAN_10151

MKLFVINFILMSLVYMSFGASVPYETVDIDKLLADDKMVTEYMACLRGEGPCNPAEKDLEEHIPLVLGNYCADCNDKQKNFVIKLATFVIKNRFDEWRQVQKRFDPDLSHADDFNKFILGS

>TcasCSP6 GLEAN_14533 DQ855492

MIPLIAIAGILAVSAAPAEFYESRYDHLDVESILNNRRMVNYYAACLLSKGPCPPQGVDLKRVLPEALQTNCAKCTEKQRTAAYRSIKRLKKEYPKIWEQLRAVWDPDDVFIRKFETSFESGKPSGVISTNTSPPSPILSNRFGENEEADAASNVISSTPLPPTTSTTTRTTLTTKFTTKPSTKPTNKPVVVTKPPQAPPFATVGANLQATVSFGTNLVGGIVRSLGTLGSRVVESGTKLANMVISAAIRP
