# Supplementary material for: Identification of chemosensory genes from the antennal transcriptome of Semiothisa cinerearia
Source: PLoS One. 2020 Aug 7;15(8):e0237134. doi: 10.1371/journal.pone.0237134 (PMC7413487; doi:10.1371/journal.pone.0237134)
Supplement: S6 Table — (DOCX) [file pone.0237134.s014.docx]

>ScinSNMP1

MKLPKPLKYAAISGGLGMFGIIFGWVLFPAILKHQLKKEMALSKKTDVRKMWEDIPFPLDFKVYFFNYTNVEEIQNGGIPIVKEVGPYHFEEWKKKVEIEDHEEDDTITYKKVDKFLFKPELSGPGLTGEETIVLPHLLMLGPVIAVSRTNPAMISMVSKALKVLFDQPKDVFMRAKPLDILFRGIMINCAQTEFAPKAVCTAFKKEAVSGLDFEANNQIRFSFFGGRNNTVDPHVVTVKRGMKNVMDVGTVVAVDGNTEMKNWRDSCNEYVGTDGTVFPPFLTEKDRLQSFSGDMCRPFKPWFLKKTSYRGIKTNRYTVNIGDFANDPELNCFCDAPDKCPPKGLMDLIKCLKAPMYASLPHFLDCDPSLLKNVKGLNPNVDEHAIEIDFEPISGTPMVAKQRIQFNIELIKTEKYDVFKNLPNTIAPIFWIEEGLALNKTFVNMLKFQLFYPKRAVNVLRWWLFSFGTIGAIVGVVFHFKG*

>ScinSNMP2

MLASRAKLIFTCSLAFLILAIVLAVWGFPKIVNKQIQKNVQLESSSQMFDKWLKMPMPMDFKVYLFNVTNTDEVNEGEKPKLQEIGPYVYKEYRERTVLSYGANDTVKYMLKKHFEFDPVASGGLTEEDQVTVINFSYLAAVLAVHDMMPPFVPMVNKALEQFFTNLTDPFLRVRVRDLFFDGIFLNCEGDNSALGLVCGQIRAQTPPTMRPADDANGFYFSMFSHMNRSESGPYEMVRGTENIRELGHIVSYKEQPVLPNWGDQYCGMINGSDSSIFPPIDESNVPEKLYSFEPDICRSLYVSSVGKRTMFNMTAYYYEIHESALAAKSANPNNRCFCKKNWSANHDGCLIMGLLNLAPCQGAPAIASLPHYYLASEELLEYFAEGIQPNKSLHNTYVYLDPVTGAVLSGVKRLQFNIELRNIPTVPQLQAVRTGLFPLLWLEEGATIPVSLQDELKAAHRLLSYVEIGRWVLLALGILAALAAGAALAKAANLIPRHNNSVSFILTNGNGHKG*

>EoblSNMP1

MMLPKPIKYAAISGGTAVFGLMFGWLMFPALLKHQLKKEMALSKKTDVRQMWEVIPFPLDFKIYFFNYTNVEDIMNGAQPIVKEVGPYHFEEWKVKEEIMDRDEDDTVSYKKRDTFIFKKELSGPGLTGEEELVIPHPLILSAVVTIARSNMAMVGMAAKGALGVFDNPSDVFMRVKPLDALFRGVIINCARTEFAPKAVCSAFKKEDMSVFTLEPNNQIRFSFFGKYNGSVDPRVFTVKRGIKNVMEVGTVVAIDGNPQLTVWRDTCNNYSGTDRTVFPPFLTDKDRLQSYTEDLCRTFKPWFQKKTSYKGIKTNRYTVNIGDLANDPELNCYCDAPDKCPLKGLMDLSKCIKAPMYLSLPHFHDCDPSLHNAVKGLNPNAEEHAMIIDFEPISGTTMFVKQRLQFNIELLKTEKHDLFKKLPGTIAPIFWLEEGLALNKTFVKMLKVQLFIPKKAVGVIRWLLLSFGSIGAIAGVVFHYKTASGEKNKVTTVKPEEEEKGVSVIGQQQGPTKVDM>EoblSNMP2MLASRAKLFFSCSFVFLILSIVLALWGFPKIVSKQIQKNVQIEESSQMYDKWLKMPIPMDFKVYIFNVTNVDEINEGGKPKLKEIGPYVYKEYRERTVLGYGPNDTVKYMLKKHFEFDREASAGLTEDDEVIVINLSYMAAVLTVNDMMPAMVPLINKALEQFFSNLTDPFLRVRVRDLFFDGVFLNCEGDNSALGLVCGQIRTQTPPTMRPSEDGKGFYFSMFSHMNRSENGPYAMVRGTENIRELGHIVSYKDKDSMSNWGDEYCGMINGSDSSIFPPIDEANVPERIYSFEPDICRSMYVSLEGKRTIFNMSAYYYEMHESALAAKSANPNNRCFCKKNWSGNHDGCLIMGVLNLHPCQGAPAIASFPHYYLASEELLEYFAEGINPDKEKHKSFVYIDPVTGVVLSGAKRLQFNVELRNIPSVPQLQAVPTGLFPLLWLEEGASLPPSLQEELQASHRLLSYVEAARWLVVAAAVLTVLIAGVVVARAINILPRNNNSVSFILSNGNGYKG

>HarmSNMP1

MQLPRELKYAAIAGGVALFGLIFGWVLFPTILKSQLKKEMALSKKTDVRKMWEKIPFALDFKVYIFNFTNAEEVQKGATPILKEIGPYHFDEWKEKVEVEDHEEDDTITYKKRDVFYFNPEMSAPGLTGEEIVVIPHIFMLGMALTVARDKPAMLNMVGKAMNGIFDDPPDIFLRVKALDILFRGMIINCARTEFAPKATCTALKKEGVSGLVLEPNNQFRFSIFGTRNNTIDPHVITVKRGITSVMDVGQVVAVDGKTEQTIWRDTCNEFQGTDGTVFPPFVPETERIESFSTDLCRTFKPWYQKKTSYRGIKTNRYIANIGDFANDPELNCYCAKPDTCPPKGLMDLAPCMKAPMYASMPHFLDSDPALLSKVKGLNPDVTQHGIEIDYEPITGTPMVAKQRIQFNIQLLKTDKLDLFKDLSGDIVPLFWIDEGLALNKTFVNMLKHQLFIPKRVVGVLRWWMVSFGSLGAVIGIVFHFRDHIMRLAVSGDTKVSKVTPEEEEQKDISVIGQAQEPAKVNI

>HarmSNMP2

MCGIIGGVSTLVVGSVLVVASSVISFVFVPNIIRNIIAGEVTLLDDTIQMERFKEVPFPMNFTVRVFNMTNPAQVLTGGVPVMQEIGPYVYRLYQTREILEKDGDVIVYKMHEHFEFDADLSYPNQEDDLVTIINVPFHAVIQVAESLFPYLMSLLQMAMDEVFKEFNTPITTIRVRDLLFDGITMCKHPTGLGIIACSIIRDIADNAQNIEEKPDGSLVFSILNYKQQLPSQEYRVQRGLDDPADLGRILSYAGSPFFPQWLNLTTREPNVCMEVNGTDAGIFAPFVETERSIYAINTDICRSVELRYERDSEYEGIPTVRFAANEWLLDNDDGCFCLNVTRGINRDDGCLLRGAMELYTCVGAFLIMSYPHFLFADVRYRDSVLGMHPNEENHKIFIELEPNTGTPIRGAKRAQFNIFSRPVRNIPVTQNLRTAIVPILWIEEAIDLPHEFVDELTERLLSSLQLVDIFIPVLIAACVMVLVVGVALTARARVYRK

>BmorSNMP1

MQLAKPLKYAAISGIVAFVGLMFGWVIFPAILKSQLKKEMALSKKTDVRKMWEKIPFALDFKIYLFNYTNAEDVQKGAVPIVKEVGPFYFEEWKEKVEVEENEGNDTINYKKIDVFLFKPELSGPGLTGEEVIVMPNIFMMAMALTVYREKPAMLNVAAKAINGIFDSPSDVFMRVKALDILFRGIIINCDRTEFAPKAACTTIKKEAPNGIVFEPNNQLRFSLFGVRNNSVDPHVVTVKRGVQNVMDVGRVVAIDGKTKMNVWRDSCNEYQGTDGTVFPPFLTHKDRLQSFSGDLCRSFKPWFQKKTSYNGIKTNRYVANIGDFANDPELQCYCDSPDKCPPKGLMDLYKCIKAPMFVSMPHYLEGDPELLKNVKGLNPNAKEHGIEIDFEPISGTPMVAKQRIQFNIQLLKSEKMDLLKDLPGTIVPLFWIEEGLSLNKTFVKMLKSQLFIPKRVVSVVCWCMISFGSLGVIAAVIFHFKGDIMHLAVAGDNSVSKIKPENDENKEVGVMGQNQEPAKVM

>BmorSNMP2

MLAKYTKTIFSVSVAFLVVSIVLATWGFPKIIRKQIQKNVQISNTSKMYDKWVKLPMPLDFKIYVFNVTNRDAINQGEKPNLKEIGPYVYKQYREKIILGYGDNDTIKYNLKKTFVFDPVASGDLREDDELTVINFSYMAAIISVQEMMPAAVGMINRALEQFFTNLTDPFQTVKVKDLFFDGLFLNCEGDNTALGLICGKIRAEKPPTMRISKSANGFYFSMFSHMNRTVSGPYEMVRGTENLSDLGHVISYQGKRIMSAWDDQYCGQLNGTDSTIFPPLEDGNIPEKLYTFEPDICRSLFASLVGKDTLFNISTYYYEISDMTLGSKSANPDNKCFCKRNWSVKHDGCLLMGVLNLAPCQGAPAIASLPHFYLGSDELADFFGDGIKPDKEKHNTYVHLDPITGVVIKGVKRLQFNIELRNVPSVPQLKEVPSGLFPLLWIEEGAEIPEWLRKEIMDSHTMLWYVDAARWLVLAVAVVAVLVSATLVARSAALIPWPRNSNSISFILGNSVNTSKVHS

>SexiSNMP1

MLLPKELKYAAIAGGVALFGLIFGWVLFPTILKSQLKKEMALSKKTDVRQMWEKIPFPLDFKVYIFNYTNAEEVAKGAVPILKEIGPYHFDEWKEKVEVEDHEEDDTITYKKRDVFYFNPEMSGPGLTGEETVVIPHVFMLGMALTVHREKPAMLNMVGKAMNGIFDDPPDIFLRVKAMDILFRGMMINCARTEFAPKATCTALKKEGVSGLVLEPNNQFRFSIFGTRNNSIDPHVITVKRGIKNVMDVGQVTAVDGQTVQTIWKDHCNEYQGTDGTIFPPFLTENDRLQSFSTDLCRSFKPWFQKKSSYKGIKTNRYVANIGNLAEDPELQCFCPQPDKCPPKGLMDLAPCIKAPMYASMPHFLDCDPALLSKVKGLNPDVNAHGIEIDFEPISGTPLVARQRLQFNIQLLKTDKLDLCKDLSGDIVPLFWIEEGLALNKSFVNMLKHQLFIPKRVVGVLRWWMVSFGSLGALIGVVFHFRDHIMRLAVSGDSKVSKVTPEEGEEQKDISVIGPAQEPAKINI

>SexiSNMP2

MLGKHSKLIFAVSMGFLVVAVIMAAWGFQKIVDKQIQSNVQLENNSMMFDKWLKLPMPLDFKVYVFNVTNVEDVNRGEKPILNEIGPYVYKQYRERTILGYGPNDTIKYMLRKRFEFDPVASGDLTEDDEVTVINFSYLAALLTVHDMMPSFVGMVNKALEQFFPSLEDAFLRVKVRDLFFDGIYLSCDGDNAALGLVCGKIKSDTPPTMRPAEGANGFYFSMFSHMNRSESGPYEMVRGRENVYELGNIVSYKGQKVMPMWGDKYCGQINGSDSSIFPPIKEGNVPKKLYTFEPDICRSVYVDLVGKKEIFNISAYYYEISESAFAAKSANPNNRCFCKKNWSANHDGCLLMGLLNLMPCQGAPAIASLPHFFLGSEELLEYFGSGIKPDKEKHNTYVYIDPTSGVVLSGLKRLQFNIELRQIDTVTQLKRVPTGLFPMLWLEEGATIPASIQQELRDSHKLLGYVEIARWFLLTVAIIAVVTSAVAVARANALLSWPRNSNSVSFILGPSVTHVNKGN

>SexiSNMP3

MCGVIGSVTTLVLGAILVIGSCIVSFVVVPNIVRNVIIGEVVLKEDTIQMERFEEVPFSLNFTVKIFNVTNPQAVLNGGVPFVTEVGPYVYRLYQTREILGIDGDIMRYKRHEHFVFDPVLSYPRTEEDMLTIINVPYHAIIQVAETLYPNLMPLVNLAIDGVFGENNQPFVNITARQLLFSGITLCKNTGLIATIACNIIRDIAQGARNIEQLEDDSLVFSILDYKEKLPSEEYEVLRGLNDPADLARILKYGGYNRFRHWAKNPEGGVTPCNQINGTDAGIYPPFVSRDQSIYAINTDICRSVELRYEYDTEYKGIPTYRFAANEWLLDNDEGCFCLNQTRGLNREDGCLLKGAMELYTCVGAFLVMSYPHFLFADDLYRDGVVGMWPDEDIHKIFVDIEPNTGTPIRGAKRAQFNIFSRPVSGIPATQAFRTSLVPILWVDESIVLPDDFVEELTGRLLHNLRLVDILIPVMIAACGLVLVLGTGLTVRAFYVRKSIKKTESVPEPYTQPEPETQPQPRTEIQPAN

>SlitSNMP1

MLLPKELKYAAIAGGVAIFGLIFGWVLFPTILKSQLKKEMALSKKTDVRQMWEKIPFPLDFKVYIFNYTNAEEVAKGAVPILKEIGPYHFDEWKEKVDVEDHEEDDTITYKRRDVFYLNPELTAPGLTGEEIVVIPHVFMLGMALTVQREKPAMLNMVGKAMNGIFDDPPDIFLRVKAMDILFRGMIINCARTEFASKATCTALKKEAVSGLVLEPNNQFRFSIFGTRNNTIDPHVITVKRGIKNVMDVGQVVAVDGQTEQTIWKDTCNEYQGTDGTVFPPFLTENDRLQSFSTDLCRSFKPWYQKKSSYRGIKTNRYVANIGNLAEDPELQCFCPQPDKCPPKGLMDLAPCIKAPMYASMPHFLDCDPALLSKVKGLNPDVNAHGIEIDFEPISGTPLVARQRIQFNIQLLKTDKLDLCKDLSGDIVPLFWIEEGLALNKTFVNMLKHQLFIPKRVVGVLRWWMVSFGSLGAVIGIVFHFRDHIMRLAVSGDSKVSKVTPEEVEEQKDISVIGPAQEPAKINI

>SlitSNMP2

MLGKHSKLIFAVSMGFLVVAVIMAAWGFQKIVDKQIQKNVQLENNSMMFDKWLKLPMPLEFKVYIFNVTNVEDVNQGEKPILNEIGPYVYKQYRERTILGYGPNDTIKYMLRKRFEFDPEASGVLTEDDEVTVINFSYLAAVLTVHDMMPSFVGMVNKALEQFFPSLEDAFLRVKVRDLFFDGIYLNCDGDNAALGLVCGKIKSDTPPTMRPAEGANGFYFSMFSHMNRTETGPYHMIRGRENVYELGNIVSYKEQKVMPMWGDKYCGQINGSDSSIFPPIKEGNVPKKLYTFEPDICRSVYVDLVGKKEIFNISAYYYEISESAFAAKSANPNNKCFCRKNWSANHDGCLLMGLLNLMPCQGAPAIASLPHFFLGSEELLEYFGSGIMPDKEKHNTYVYIDPTSGVVLSGLKRLQFNIELRQIDTVPQLKRVPTGLFPMLWLEEGATIPASIQQELRDSHKLIGYVEVARWFLLTAAIIAVVTSAVAVARANALLSWPRNSNSVSFILGPSVTQVNKGN

>SlitSNMP3

MCGVIGSVSTLVIGAILVIGSCIVGFLVVPNIVRNVIISEVVLNEDTIQMDRFEEIPFSLNFTVMIFNITNPETVLNGGVPFVTEVGPYIYRLYQTREILGIDGDIIRYKRHEHFVFDPVLSHPRTEEDILTIINVPYHAIIQVAETLYPNLMPLVNLAINGVFGKNNQPFVNITARELLFDGITLCKDTSLIATIVCNIIRNIAQGARNIEQLEDDSLVFSILDYKEQLPSEEYEVLRGLNDPADLGRILKYGGYNRFRHWAKNPEGGVTPCNQINGTDAGIYPPFVNREESIFAINTDICRSVELRYEYDTEYKGIPTYRYAANEWLLDNDEGCFCLNQTRGLNREDGCLLKGAMELYSCVGAFLVMSYPHFLFADNLYRNSVVGMWPDEDRHKIFVDIEPNTGTPIRGAKRAQFNIFSRPVNGVPVTQPFRTALVPILWVDESIVLPDEFVEELTGRLLHSLRLVDIFIPVIIAACGLVLVVGAGLTIRAFYVRKSVKKTESVPGDKTQPALETKPEPETQPEPLSQPEPRTENEVAK>HvirSNMP1MQLPKELKYAAIAGGVALFGLIFGWVLFPTILKSQLKKEMALSKKTDVRKMWEKIPFALDFKVYIFNFTNAEEVQKGATPILKEIGPYHFDEWKEKVEVEDHEEDDTITYKKRDVFYFNPEMSGPGLTGEEIVVIPHIFMLGMALTVARDKPAMLNMVGKAMNGIFDDPPDIFLRVKALDILFRGMIINCARTEFAPKATCTALKKEAVSGLVLEPNNQFRFSIFGTRNNTIDPHVITVKRGIKNVMDVGQVVAVDGKLEQTIWRDTCNEYQGTDGTVFPPFVPETERIQSFSTDLCRTFKPWYQKKTSYRGIKTNRYVANIGDFANDPELNCFCPKPDSCPPKGLMDLAPCMKAPMYASMPHFLDSDPELLTKVKGLNPDVTQHGIEIDYEPITGTPMVAKQRIQFNIQLLKTDKLDLFKDLSGDIVPLFWIDEGLALNKTFVNMLKHQLFIPKRVVGVLRWWVVSFGSLGAVIGIVFHFRDHIMRLAVSGDTKVSKVTPEEPEQKDISVIGQAQEPAKVNI

>HvirSNMP2

MLGKHSKIFFGVSLIFLVIAIVLASWGFQKIVNKQIQKNVQLANDSKMFERWVKLPMPLDFKVYVFNVTNVEEVNQGGKPILQEIGPYVYKQYREKTILGYGDNDTIKYMLKKHFEFDPEASGSLTEDDELTVVHFSYLAALLTVHDMMPSLVTVVNKALEQLFPSLEDAFLRVKVRDLFFDGIYLSCDGDNSALGLVCGKIRAEMPPTMRKAEGSNGFYFSMFSHMNRSESGPYEMIRGRDNVYELGNIVSYKGQENMPMWGDKYCGQINGSDSSIFPPIKEDDVPKKIYTFEPDICRSVYADLVDKRELFNISTYYYEISETAFAAKSANPNNRCFCKKNWSANHDGCLLMGLLNLTPCQGAPAIASLPHFYLGSEELLDYFQSGVQPDKEKHNTYVYIDPVTGVVLSGVKRLQFNIEMRQINNIPQLKSVPTGLFPMLWLEEGATIPESIQQELRDSHKLLGYVEVAKWFLLTIAIISVIASAVAVARANALLSWPRNSNSVSFILGPSVTQVNKGN

>OfurSNMP1

MQLQKPLKIGLGMMGAGLFGIIFGWVLFPVILKSQLKKEMALSK

KTDVRAMWEKIPFALDFKVYMFNYTNVEEVMKGAAPIVKEIGPFHFDEWKEKVDIEDH

DEDDTITYKKRDYFYFRPDKSGPGLTGEEVVVMPHLLMLSMATIVNNEKPAMLNMLGK

AFNGIFDEPKDIFIRVKVLDLLFRGIIINCARTEFAPKAVCTALKKEGATGMTFEPNN

QFRFSLFGMRNGTIDPHVVTVRRGIKNVMDVGKVIAIDGKTEQDVWRDKCNEFEGTDG

TVFPPFLTEKDNLESFSDDLCRSFKPWYQKKTSYRGIKTNRYVANIGDFANDPELQCY

CDSPDKCPPKGLMDLMKCMKAPMYASLPHYLDSDPQLLKDVKGLSPDANEHGIEIDFE

PISGTPMVAKQRVQFNIILLKTDKMDLIKDLPGTMTPLFWIEEGLALNKTFVKMLKNQ

LFIPKRIVSVVKWLLAGVGFVGLVGSLVYQFKGKMINFALSPSSAQVTKVNPEINQQN

QPKDISIIGESQNPPKVDM

>OfurSNMP2

MLGKHTKLFFGVSLVALIVSVILAAWGFPKIVSKQIQKNIQIDN

SSVMFEKWRKIPMPLTFNVYVFNVTNVEDVNNGAKPRLQQIGPYAYKEYRERTVLGYG

DNDTVSYTLKKTFIFDQEASGPLSEDDEVTVIHFSYMAAILTVNDMMPSITGVVNGAL

EQFFTNLTDPFLRVKVKDLFFDGVYVNCAGNHSALGLVCGKLKADAPQTMRPAGDGNG

FYFSMFSHMNRTESGPYEMVRGRENIKELGHIISYKGKSFMKNWGNDMYCGQLNGSDA

SIFPPIDENNVPEKLYTFEPEVCRSLYASLVGKSSMFNMSAYYYEISSDALASKSANP

GNKCYCKKNWSANHDGCLIMGILNLMPCQDAPAIASLPHFYLASEELLEYFDGGISPD

KEKHNTYIYLEPVTGVVLKGLRRLQFNIELRNIPMVPQLAKVPTGLFPLLWIEEGAEL

PDSIIQELHQSHTLLGYVEAVRWALLAIAIVATAISAIAVARSGLIPVWPRNANSVSF

ILSPHPNSDVNKVH
