# Supplementary material for: Identification of chemosensory genes from the antennal transcriptome of Semiothisa cinerearia
Source: PLoS One. 2020 Aug 7;15(8):e0237134. doi: 10.1371/journal.pone.0237134 (PMC7413487; doi:10.1371/journal.pone.0237134)
Supplement: S7 Table — (DOCX) [file pone.0237134.s015.docx]

>ScinOrco

MQMAGHFLFNYYDENAGMSVLLRKVYAGVHAVLITVHFVLMGINMAQYSDEVNELTANTITVLFFAHTIIKLVFFAFNSKSFYRTLAVWNQSNSHPLFTESDARYHQLALSKMRKLLYLILFTTAAAVVSWVTLTFFGESVRMITNKETNETITEPAPRLPLKAWYPFDAMSGSMYIFAFVFQIYWLLFSMSIANLMDVMFCSWLIFACEQLQHLKAIMKPLMELSASLDTYRPNTAELFRVASTDKSEKMPDTTDADIRGIYSTQQDFGMTLRGAGGRLQNFAANPTNPNGLTQKQEMLARSAIKYWVERHKHVVRLVASIGDTYGTALLFHMLVSTITLTLLAYQATKVNGINVYAFSTIGYLSYTLGQVFHFCIFGNRLIEESSSVMEAAYSCQWYDGSEEAKTFVQIVCQQCQKAMSISGAKFFTVSLDLFASVLGAVVTYFMVLVQLK*

>ScinOR2

MPNTVNSRPRRYFGFHYVLLRCTGLGWWHHPDEGDTRNFPGWYLYYSIATQIIWVAGFVGLETIDPFVGEKELDSFMFSLSFVITHNLTIIKLYLFFFRNKEIQDIVRTIEIDLHDYYQNEDTNRKTIKTCKTFTFAFIFFGWLTIGNSNTYGAIQDIHWKAFLRSANDSSQVPPRSLAQPIYIPWDYQKDQSYIPTFILELVGLLWTGHIVMGIDTFIGSVILHMSSQFMILQEAIETAYDRTLSALREGTLHEADENLPSDSADISLMNSEENNERIVRAFYTEKQIESALQTTLVNCIRQHQVLVGCVEKFSKTYSYGFMTQLLSSMAAICVVMVQVSQDASSFKSLRLITSLAFFAAMITQLAIQCFTGNELTFQAGRVSSAVMKSKWERMPPRLRSLLLLLMMRAQRPIRLSAAGFANMDNDCFLAIMKAAYSYYAVLSQKQV*

>ScinOR3

PILKALFSKKLRTTSLNEELICSRSDYESSFDIPKKILRWITIELTHDISPCASICWLYFYWFEFTNVFVAMLMELFSMIETARGGSFDDVVKIFRMLPCFGMAVLSLVKSYSMVKHRSVYENLVLEVGDMWHDSALSEGEWNIINNARKQLLFVIKGYYWCNNVLLVSFLSPPLIESIKRLLGYENQMLLHFFYWLPFDPLQPQYVDILLLMQTWHALIVIWANICSDLLFCTFLSHLTTQFDVLSLRIQSFIYVPIDKQLIEEYPLSEFSKKYLMNEHSEIDWETKMLKDLSEIIIRHKTLIRLSGDVEAMFTFGLLMNFLNSSIIICFCGFCCVVVEKWNETAYKSFLLTALSQTFLICWYGQKLLDSSEGISHAIYNSGWYRAPRRVKSALIIMLHRSQKRIQITTYGFSVVSLESYTKIIKTAWSYFTLILNTYKQ*

>ScinOR4

MGNAQFIHDILSKRNYDFGRTDVTLKNFHPQIYMTLATEGVFFVKRDSYLRFCIPFYNIAMTIIGVTFEGLNVYMGMNTGDYSVVWESVCYVSIISSTITVYAGLLLNREKIWSQIEEMERDFEEICNLGPRYRDPFMEGQLLVWKLFCGWSIFVITICSSFFIYPCILLVYQSLKPSEEYKNRPLVFPLWLPNDDPYRTPNYEIFFVVEVFFVIAFTLCFGGYVYTLFHFLLHYYTKMDMIILDFQVIFDGLDESVVKLPKTDPRRISVQMTLNKRLKRIVTWHLSIFRAIHTAASVFRPTLIYQVMCSAVILCLIFNQIVMKLDRGEFDVVFTLLGAGAVLQLWIPCYLGTILRNKGFAVGDACWNCGWHETSLGLLIRIDILVVLMRAQMPIFIKYTGQPVVQLETFSAIMSTSYSYFNMLRQSQK*

>ScinOR5

MIFTRYKKRFLVYLNKENFDHMEGLIDPFEFHSLFLVMAKLFSVMDYKEIPKWMYISPICSAASAVSAGTIFLISSVEYVRTFKVHALTECGTYLMIMCYKFLILCSIVNGKKHYHNFLQALRDDFHYICTRGFKYRDRFFANQRVTFRACVFVAIFIGSMGGGMAILACIAVLVALHRREEGAVRPLPFPFWLFGVNLHASPAYEMLFLSSMLFTFFHTYLYIFMMMSQVLWIREITCKADIIRWNLQDLLVDVSPARNNEEENYYTALIKHRMRDIIMMHHSMIKLTEDYAAVFKKCLLYEQMLASPIICMTAYCFAEKFDAGEIQLILLVLCTAVIVVVFVPSYLCTHLLEKCQSISDACWDIPFWNAGPVVRVYLVLMMQRSLRPLPIVAAGFDDISLQTYSNIMTRSYSCFNMLRQADFNFS*

>ScinOR6

MGPSVRSRVGPLNTPYLKLMSITLSILAFWPDEVSGKKRIINRSSKKMLVFYFFVTVLCVLYIKNNVKIQPFLEMGPNYINTLLLVVCLSRLIIPCSASYKELTNTFFNDIHLFHFKDQNEFAMKTYKEVEKYASFFCKILHFEVTSGVFFYNLTPLYKSYRNGMFKDIKPANATYEQAVYYEMPFDYINQFPGFLAVYLANCFISFDVAFIAGIFDLYVYVIIFNLWGHLKILKNNLITFPKPKTFVGTSYNTMPWYSEEESKEVRKLLIKSIEHYKSIKNFMNDMSGLFGPALCCYFSYFQICGCILLVESTQMTAEAIGTHGILTFTTFQQLIQISVIFELIGTQSDTIQDAIYEVPWECMDVKNKKIVLFYLLIAQKPLSFKPLGMVSVGVQTMASILKTTISYFMLLRTTTSSHE*

>ScinOR7

MGIVKNIWKKITHTNALDQVSGALEMAFFEDVYRVTYLAGLSTTDRTVLYLLYSSIVKLMVALIVTGELWYTFTLASSLDEIAACVNVIVIQLITLFKFKNMITHKEFYQTLARSMETSYFDMTTKKRRELVLYWAKTHERYVKLLLGLGNCTLAAWHLYPLVDELDYNLMLSIRLPFHFDTPLLYPFTYLIVGIAFTYTAHSVMVTDLVMQAHMVPLICQLSVLADCFENIIRDCCVASGAVFPDGHRGDLTLNNDFKREYLRRLGNLVEQHKLILNHSMHLKAILSGPLLGQLAASGTLMCFIGFQATTTILENVTKCLMSLFYLGYNMFGMYIICRWCEEITNQSHSIGESVYCSGWETGVSLLPGVRTTILLVIARANNPVVFTAGGMYDLSLKSFTTLVKTSYSALTVLLRFRH*

>ScinOR8

MGIITGLWMQLTYSEYLESCGELERLFFESLYRVLLFNGLISTDKRWAYIVYSYFIKLLLILLCIGGAIYVLTEKHSIDEDIAAANEGLIWYLTIFRYLNMEKNQALFKKLSSSMDESPYFDISTPKRRQLMEMYAKENEKYLKLLMVVGHITMIEWIIFPLVDGVDYNLVVGVRLPFDHRSDDLYPLGYFLVALAFAQMAYFVMVMDIRLQAYLIHLLCQFAVLVDCFENVVEDCRHGFEDVPLNYLMYNKVFAERYLKRLGNLVEQHKHILSNATLLRNTLSRPFLGQLASSGFLICFIGFQMITTITEDHNYLQGIVSFCFLGYNLFELYIGCRWCEEITIMSQRVGDSVYFSGWESGLSMLPGVGANVRIVIARAAKPLIFTAGGMYELSLFSFSNLLKTSYSALTVLLRVR*

>ScinOR9

MAETKLLFDQSIQKLNILFKFSGMNLKHKIKTPLDNIKHRWLFTFNFIWVFSAVIACVYYILVGFVQGKDFIEVTSVAPCLTFSMLAIFKSIFYLINEDEVFNLIHGLRELEAKGSERAHSIEKENIVKEDRDFLNTVINVLYFLNCSMIVVFDMTPLVLIAVKYVRTKEFEMLLPYLDVWTFLPYKMKYWPIAYLHQVWSECIVLLEMGAADYLFFTCCTYIKIQFKLLKYDFTKLIAVPTDDEDSRDDIRDADIKAKFTELVKWHQDLIECSNRLETIYSMSNLLNFMSSSVVICLTGFNVTIGGDIVVVITFVTFLFMALMQVFFLCFFADMLMAESTKVSDAVYNCRWYTAQRHLVKDLILVQTRAQKPCKLTAFGFADVNLRAFTRVLSTSWSYFALLQSVYGSV

>ScinOR10

MKNEHFLKHPKDQRFYKTIALVMGAIGLNTQYWQWFKMPKNVLFRIQRDTLFITVPMSVISQTTYLVLNHSKHEFEALSVMYAVLPVTTIVWIKLYSAQTRSYKLVMENFMTKIHLYNYLTTHENSKYVKKILVEAELFVRWSTFYLVFFCYASCTGFEIVPIVHNFQKFESFKNITERLEIIVHFWLPFDYEYNFNNWVIVQTILFYCGYMCVTIISMFEMINLTCIYHIIGHIKIIKHQIKTSFTNDMTNEEVTKNIIKISRYYNFITESLKDVENAFGFNVASIYLHNLIAGGLLIYHLSMSGTETGTILTYGLMIPLYMGVLIIVSLALEYIRIVASDIPDLIYDLHWESMSVTNQRMLLLVLCQVQPVLEFNAAFNIKTGVQPAIDILKTTFSFYVMIKSTV*

>ScinOR11

MEPTSFDNLKEDYLTTIDFVTSKLSKTFIYPFVGRSKNVIRCYKTICICIAITIFQLTTSLIVSKFEDWFEIINIAPNLGVCVLILVKYTKMHTKNHVYDNFLNHAKVQLWNVVDVKSNKHRKILESNTHLTKHLLFFQYYYMILLIAIVVTFPRIIMFVDTKILGNDIKYLYPFDGWYPFDKVRWYNFIYVWESWMTAIVVSLFVFCDMLHLLLSRHVCMELNILGSTMENLISSQDRKSITKQILAKETHNNIKEKLKTIIKSHQTLVGMVADFEEVLGSGMLLIYIFGSVFICLTILTALMVDDLYMSLRYFCFFISLLVEVFVQCIIGQILIDNSKSLEQAIYFSDWSYADKETRQMLHIFLIRAQRPLKLSAKGYLIMNLNTFGGVCSLSYQFFNLLRTVYT*

>ScinOR12

MQQISHLDKTLNGLEILNRYAGIYIKSGNNTNVDRFKYRCLYLSHSFAANYQLFCSIIWCINGVRNGEDFIAITYTIPCLTISVLAQFKGIFLIKNEKRADELIAKLKNLDEKEKKRAQSEEKDEIVKSEWKFLNYVLKTLSIFYTLLVAGFSISPLVVIALNYFQTKEVELILPFFAIYGYDPFRWMIWPFVYLHQCWSELAVVLNISAADYFFFTCCIYIRIQFRLLKYDLENIIHVEKNSNKLFVENEMFRNHFNELVKWHQDLIESVDILEIIYSKSTLFNFFSSSLIICLTGFNVTIVHDATNMMTFTSFLFVSLVQVFCLCFFADLLMTSSMNIGEGIYNSNLYMAEPKVAKKLLLILTRSQKPCKITASGYADVNLKSFMRVLSTAWSYFALLQTMYGN*

>ScinOR13

MEVLKDYPKEFAKLFRPSFNYLFKINMKFLADDKGFVRKYWRYSYIIPCLMINYVTQIWNLANMMSKGKIFEIAYLLPPCLVSTQAILKTIVLVPKTHLLTNIISELGTLWRARGLTETQNNAKDLLLKRMNFCNGVSYWVGIIGTTQYLSTPLVETLVRQLVLKQDCELLLPLDCSYPFNVTGWPVYIAVYGFQVYSMTLCVSVYVGSELIMITLCALLGVEFTLLCEDLLHVTTNNKGKREKLGNGKERMTLNDVVKRHQKLCVLSKKLEEAFNQMIFVDLMFVGLTTCFFRYAAQYSRGPTYMANNYVAVISSLVYVLYLCYYGELLMGASVRIGDMAYHSSWYRGDKRYQKTILIIILRSQTPCCLTSMKYAPVTLNMFTKVMSSTWSYFSLMNTVYGDN*

>ScinOR14

MEKNTLLIDQTIKKIEILFLYFGINIKSGEQTASTLKIKLVYWINFFLLLYNFLGGIVWFIFGIQEGKDFLELTEIAPCILLVTCAYSKGILFNANEKHNHKLIHILRNLEKKGMKRRQSLKKDSISNKEKKALEATIDKLNILFAAVIISFSLSPLVGMAMKYYKSNVIQPSLPILIIYPFDEFDLRIWPIVYIYQIWSEIVTTVYIGTADFFFFTCCTYINIQFRLLREDFEELFPSTNEIVVENKEFQKRLRELIKWHQELIEAANILEIIYSKSTLLNFMSSSMIICLAGFNLAVLVSDLTFIVKFLCFLCMGLVQIFFLCFFADILTTSSMGVSDAVYNSLWYNAEAKIGKDLLLILARAQRPCKITACGFADVNLKSFMRVLSTSYSYFALLQTMYAN*

>ScinOR15

MKINLCSTFSTLRPHFDALARVGYFKIVLRDRTGTQRALQYALRVFMWSLVLSYNLQHLIKVIQVRHSTDQLVDTLFILLTTLNTLGKQVAFNVRCGHVDNIIRITNGPLFSATKLYHVDLIKSEALLMSRLLKLYHGAVFFCGILWSVYPVVNRALDQPDNFTGFIPFDTTPTVAFALAHAYMSVAITLQAYGNVTMDCTIVGFYSQAKTQIQMLRHNLEQLVDGVKINGRLIVEDNKQTVYRDNGDETTLLQRRFVRCVEHYKQIIGYVKEVESTFGEAMVVQFLVMAWVICMTVYKMVGLSITSAEFVPMTVYLSCMLAQLFIYCYFGTQLKYESELVTQSIYSSDWLVLSPQFRRQLLVMMQVSSRPLLPRIAYVVPMSLETYIAVLKSSYTLFTFLDHN*

>ScinOR16

MHDDASAGASVRAHVRLLRACGFLRARDAGARGAVYRALALAVTATYLLQECACAWAARTDMDELARVMFLLLCHVTSLAKQLVFCADADRIDALLASLEGPMFAGGSERARALLAARARVSSRFVRCYSGTAVVTCTLWAVFPVLQALRGEAVRFPFWVGFDYSSWPRFSLVLLYSYYVTTLVGIANTTMDAFIATVLSQCKTQLTILRMNLEDLPERALVAARRERDGARAACASERDGACGRYHRWLDTLFLECLAHYDQICQTAHTLQDIFGGAILIQFGIGGWILCMAAYKIVSLRVLSGEFASMTLFICCILTELFLYCYYGNEMSVESARVVQAAYCMRWARTPARFRRALLVLMERARRPLRPAAGRVVPLSLDTFLKIIKSSYTFYAVLRQTK*

>ScinOR17

MEPTNKYQYKPSDTTKLFQKLSIILYLCGFVDFWIEDVKLPAKFVRLYNSFRIFIELLMFSFMVLEVGAFFTQNNLTEKQSADRLVYGISHPILYIYCLNLLYYKKEIREVLYRLAASLKDVYNDEEVEKHMLRKLKFYLMAYVTSCGSCMVFYGASAWIQYIRLGSPFTTVITAWPDTLDESHQAHIFRVLFYLIWWIYFTRVSASYILILSMFISLSFQYTNLQSYFSGLKRIFDSPLEQRDKEAQFLSAFETGVRLHLDTIWCKQECQRIYNIIFSAQLLQTVAELVSVMSMMINSERTVVNLGAVMSTTCGALISTGFLMWNAGDVTVEASYLPAAIYCSGWQNVRHASLQSRHLVIMAITQAQRPVVFTALGFVPISYETYLKIAKISYSVFSVVY*

>ScinOR18

MANSIEQAKSEIDKSLNLCTFCMRRIGLSFASPKGAAAYLKQQVMFALSVLGICYHVFSEIVYIGLTLSNSPRVEDVVPLFHTFGYGALSIAKVFVLWYKKDIFTKLLDELAEIWPLEPLNEDDQDIKNNSLNALRIAHRWYFTVNVLGVWFYNITPIAIYFYELWKTGEATIGFVWVSWYPFDKHQPIAHVAVYIFEIFAGQTCVWIMVCTDLLFSGMASHISLLLRLLQHRLETIGAEDRPEDDHYQEIVDNIKLHQRLIKYCNDLENAFSLSNFVNVVLSSVNICCVVFVIVLLEPFISVSNKLFLGSALIQIGMLCWYADDIFYANANVALSAYKGNWYVTNPKCRRALLFLIKRAQKPIAFTAMNFTDITLVTYSSILYRSYSYFALLYTMYSEH*

>ScinOR19

MGFLLKQTIGSLTVSLNVVKLFGLLVPGQLTKKQRTMFMWYQISWYIYMNGIYLIVQAGDLFLIWGDVPLMVSTSLLLFTNIALGMKIINVMLRRSAVQNIIDDGEKELANEDREEGVAIIQSCNRETSQFSNLYIFLAATTVAAWATSAESGDLPLRAWYPYDTSKSPAYELTYFYQISALSTSAAVNINLDIVATSLIAVCRCRLKLVNLSLMNLCKGPFVSTNGSHQTVEEIILERVKACVRHHQAVLSSARQIQRCFSIPVLAQFTVSMVIICVTAYQLVVELHDTPINVVRLASMSGYLLCMTLQVFLYCYQGNQLMIESTEVASAAYSCPWYLCSLRFRRALLIIMIRSKRASQLTAGGFTTLSLSCFTAIVKASYTFFTVLQRVEDRQK*

>ScinOR20

MGFLLKNTIWPLTLCLNLIKLFGFLVPGELTERQRTLWYFYQSFWFIYLAGIYIIVQAGDLILIWGDVSLMVSTSFLLFTNIALGMKIINVMIRGRAVQDIIDDGEEELVNEDRDEGLEIVRSYSRCNREVSQFLYLYVFLTVLTVLGWAASAEKSTLPLRAWYPFDTSKSPAFEITYFHQVSALYISASLNVSLDTLATCLIAVCRCRLKLLNLALLNLCIGDNGDLQTHDEAVLPRLKSSLRRHQAVLKSAGEIQRCFSEPILAQFTVSMVIICVTAYQLAVELHNTPKNVVRIVGMTAYLFCMILQVFLYCYQGNQLMVESTEVTSAAYSCPWYLCSLRSRRALLIIMIRSKRASQLTAGGFTTLSLTCFTAIVKASYTFFTMLQRVEDRQK*

>ScinOR21

MTYPNKTAAYFYKLSYIVFFLGSPNFWIEDLNLPDTFYKTYRRASRYLNVLFTYFIVFELGSFITQKNLTKTQEADLMVYAIAHPIMFSYTWILSKYDKDVQTLFRLILRLKSIYNDKDVEEKWLKKSKRDSRAIAFTCIMAVTMHTYGPLMTYIKSEGTFTTLITAWPLVDDPSAAAHAARVFNNIMFWVFVSRVIASYMLVISLVTSISHQYMNLQSYFHSIEAVFEDENTDQCIKEVEYEKSFKLGIELHTRTIECVDQFRHICGIIYSAQIVFVITTLVFLMSQMMEAERNIVSAATTAITVSTVMFGAGFFLWNAGDITEEAAALPTAMYCSGWQHCRGHSARRVRKLVTIAMAHAQKPVYIKGLGMMIFSYESYVSLVKFSYSVFSVVY*

>ScinOR22

QFLRSLEDPEWPLMAPNYWILKRVGFLLPQTQVGKILYIIVHEIVTLFVITQYMELYVIRSNLDLVLTNLKISMLSVVCIFKVNSFIFWQKRWIEVIEYVNEADKFERQNQDKPKEKIINAYTKYCRRITYFYWALVFITFLTTTGQPLMRYLTSPTFRENLRNGIEDFPHIFSSWMPFDKNHAPGCWITVVWHVLLCAYGAAVMASYDTSVVVIMVFFGGKLDLLRERCKQMLGARGIEISDDEAGVVIRELHKIHIMLIKHSRLFNSLLSPVMFFYMVMCSLMLCASAYQLTSATNAAQKLLMAEYLIFGIAQLFVYCWHSNDVLIKNENITLGPYESQWWSASLKQRKDIVTLTGQLQIRNVFSAGPFANLTLPTFITILKGAYSYYTILRK*

>ScinOR23

MKLVSPEALLKHTQTPGERHELDDIFAPVLFLQNIVGTHVLDPNWTWQKNGIKIAAMFSLIIYVVIGTIQFIKETDNFVLCAEAIFTFIITFQFPFLVFPIIFLRSTFCDLYTLVKSSLFQIIEEISPERGVYLKAMMRHITRLVISSIVTCSLSYFLPVIWLYLNGQRVNLSPSTSILMPMTSPTFEIGLALHLIFFIMLATSTLTVNMWFVVLVAFLCEACDCAVKILNQKKDKNDASYADSLNETLKRFYSVHIILMRYLNILNVMYRWPMLTSMVVMYAATCVYLLVLTEDVNWKLAIIDIIVFVEIAAFNILGELIKIKAENMRRAIIYFDWTSLSLKQRKNYYIIICYMNKDFGVRTAIGVQLSLVTLTSICKGSYQAYAVINNMNN*

>ScinOR24

MGVFVKNVNRSVSLSLTALKLVGFWPPQGLGRNEKIVYSFYAVLSFMFLLGTYLIIQVVDLFLIWGDLPLMTGTAFLLFTNLAQAAKILNIVLRRKVVSSIVDEANEALMTVSSDEARDIVKSCDRETFTQQVLYFGLTVITTIGWATSAESNQLPLRAWYPYDTSKTPAYQLTYVHQVIALFIAAYLNVAKDTLVSTLVAQCTCRLRLLGLALTTLGNDLTVPDELMFTPAQEEMITSRLRGCVVQHQAALEAVLQLQTCFSIPTFAQFTVSLIIICVTAFQLVSQTGNVVRLLSMGTYLLNMCFQVFIYCYQGNQLSEESSELGVSAYQWPWYACGARARRSLLVVMVRTRRAARLTAGGFTALSLASFMAIIKASYSLFTLLQQVEEKK*

>ScinOR25

MDMPHYDDIFKQLKLNFWLIGIPWGNVGLTIRFFICLLSLSLIVIEEAAFLVSKVNTVNMLELTQLAPCTGVGVLSVVKVIAISMKRRKIFELSENLSRLYSAMKFESETGPVIRSQMVSLKKLVRYFFILNAMLISVYNFSTPVMILYNYLRNGEASFILPYQVLLPFEINSWTIWGLVYTHSIISGFICVLYFSTIDVLYSVLTTHICINFTHISHQLKHMNKSNPALRDIVKNHQYILKLSEDLEDIFTAPNLFNVLVGSVMICALGFNLAMGDLKQIPGCILFLASVLLQILMMSVFGENIITESRKVGDAAYTCEWYEMEEKIKKDILTVMIRSKRPAKLTAYKFSTISYGSFCTILSTSWSYFTILRTVYTPSETS*

>ScinOR26

INLTYLFLVGLWPGKDWTKKQASIYRIYQKTLLCFSVTFLVITGVKTYEIRHDIIYFLANIDKSIVAYNYFFKVIIFLKRRGDFKRLVDDILSSGDTVRGVNRDRMIDHLVLVTGFNSVLMLVFSMLGLYNGDMTIEAWMPFDAKKNKMNMALSSQILAVIFCIPFSYRAIGMQGIVCSILIYLCDQLETLQNNIKDFEFCKENERESRERFKKIVQKHIRLMRYATSMESIFSEYFLIQNLAVTMELCLNAMMVTMVGTSQKIVLAGFLGLLGLALLNAYIYCKLGNELIDQSEGVSFAAYSAAWTHWPVDLQRDVLLIIRASQRPLQMSAGGIAAISMHTYGTALYNGYSIFAVLNDVVEN*

>ScinOR27

MIVIPNITLLMKIPTLQLQDSKLDKVYELLKGTAFAATDVIDAKILKSNSMQMNRIARVLNYAISFMSVFWVGSFYFKRYFDRTEVVHSYVPFRTDTWANYTISVLLECGPILWIGFGHISLDILVATFYAQAQTQLKMIKHQLTQLYNEEGENKLSTFNFQYKDFTDGTVFQRLVQVVNRFERLVWYKNEIDSIFNYSLSFQFLAATSGVCLVIYRMTVVSVTSFPFIFLALLFCVLLTQVFLYCYFGNLVEFESRSINDSLYLSDWASLSPSFRRLLLVAMTRWSQPIRPQVCGIIPLSLTTFVQILKSSYTLYTVLEAAK*

>ScinOR28

ARQKLLSLAMKLEDPNYPLLGPTVKGLYLFGLWQTGSKLRTVTYNMAHFWTIIFTITEFMDIYTVRHDLNKVMNNLSKTVVTVICNVKAISYINRQKEWRQLVTEISQEEIMQLNKEDTIIPKQMNAYKKYTRIVTYLYWMLVFNTNLLLIISPALKYASSSSYREEIRRGKEPLPQIVCSWFPFDNTKMPGYVWGVCFHVVIGSFGCGVLAVYDMNAVAIMSYLKGQMRILNGKCRCIFHGVVSQEVVLERIKECHRHHAVLLKHFKIFNALLSPTMHLYVLVCSCSIFSSVIQFNLLFLFCWHGNEVAIEVKLFCDFLP*

>ScinOR29

CKVLSAQTNSYKIAHSDFMDKIHLHCLYTQDFPRQKHVFIKNLLLKIEKNTRTIFYSLASIAFICCTAWVFVPMMHNIQNNHSFRNITTRLELGLYLWQPFDYEYNFYGYLVSQILITYCCFIGTFILVLFDTINIVYIFHITGHIHILKYDLEIGQNELLTDQEWERKLKDITVKYYFITRSFKNLEKAYGLNIASIYGQNLVQGGLLLYMFITAGDDVGVILTYGLMIPIFMGTLIILSAVLENVRIQADDIPNMVYNLPWESMNIRNRKMVQCIIFQVQPKLEFKAAFNIKAGVQPAVSILKTTFSYYVMIKSRKEM*

>ScinOR30

MSWNQKKIRGILNLIDEDYKIVKNLPEEEQAIMHKYIKRGREVSVEWLTVTGGGSLIFPGKNVILLLYYWIIGDPKLIPIFDFEYPAFIENNVDYLVIYLTVYALIIYFGSYATCMYTAFTPLGPIFMMHCCSWLEIMEIRIGKLFDGDLKETEEKLKDIIKHLQHVYSLVEQILTSFRVIYECTLRGAILLIPITFYEIIMAANKGEVPLEFISLIVGGVLVTSSPCYYSELLMSKGESLRQTMYSCGWESVPDTKIRNMIRLVLVRALRPCALRSLFKPICLETLAEVFQQSYAIFNVINSMWN*

>ScinOR31

LVDISRLKEVYNDLDVEKKMMKKSKLYSSAFCGMCVFSMLMYTIDPLVQCARKGTTFNTVITVWPLVEDSSTSANVGRFVWNLLWWSMMSRVLGVYAFVFSLTSCMSHQFIDLQSYFLSLDSIFQSEKLSQILKERKYEEAFIIGIKLHYNTLQCVESTKNICGWLFSGQIIFNITLLVLLLSQLLKSEGNLILVTAVGGAAFWIITGTGLFMCSAGDVTVEASALSTAMYSSGWHRCRGASSLRVRRLVVTSMVYAQRPVVIKGLGILSLSYQSYVSLVKSTYSVFSIVFQKIK*

>ScinOR32

MSSSRQSDIFYKHFIFWKVFGLWSGRKPNKNYKYYSFIYILITLVIYNVLLTLNLAFTPREIEYLIPEVIFYFTEIAVTSKVFMIIIMRDKILEAFSLMDGEELGSDDENSRKIVHNANANYRTYWKSFAAISHAAYLSQVLVPVVLHFIFHTKLDLPICKYNFLNDEIRNRYFLFWFIYQSWGMYGHMIYNVTVDSFIAGLLVNAIIQLKILNAKLSRLKLSHQEKKLSSDIQEKIQMLRLRKCLVRYDVILKYCEIIQNIMDKTMFVVFGMSSLIICVVLCGLLLVIIFQSR*

>ScinOR33

LIRVILLKQIIFIFQILITVEKLTRWTNYYYIFFCYSCYIGWEFVPIVHNFQRYDNFVNITTRLEIVVHFWLPFDYEYNFNNWIIAQIASFYCGFIAVTIIAMFDTINLSFIFHIIGHIKIFKHQVKSMFMENMTQEEIKCKIIEMAQYYNFILETLKNVEAAFGFNVASIYLHNLFGGGLFIYQISKSTEFGTMVTYGLMIPLYMGVLLAISFSLEYVRIVADDVPDLIYSLNWENMSVSNQTMILLVLFQSQTILEFKAAFNMKTGMQPALNILKTTFSYYVMIKSRDE*

>ScinOR34

KKFKRAASILIKIYCFTGVVFILSPFIEYAFALAGGKIPEYPHILPSWNPFDKIHILCYIAVIVCETVAAVYCVCVHIAFDATVIGVMIFICGLFSSLVYQSTTIGGRGKICNLSKRRDARAHSRIIKCHKIHIELVRCITGLDRLLKNILGFYFLLATLTLCTVAVRLKTERMGAMQLVSLLQYMCATLTQLYIYCSYGDAVSNESSISATQGPFGAAVWCLSPKIRREICILGIGMMKQRRLHAGPFNTVDLPSFIQIVRAAYSYYAVLDGNKTNDNINT*

>ScinOR35

LPAEYTEVLLPCLDLLKNCNILFFDDRSPLRRVLPYFYILPAVLAYHISLAIYLIEGFTRSMDLDELVFVVPIYVVCVQSVFKASIVFSNKKNIRSIILRLGKLWRTQELSEVQISKKNVLLKRLKFCYAAFYWMNILGSWQYILTPLLETCFRRLILRQDSQDFLFPFGCYFPFNPRSNWIVYLAAYVFEAYSMLKIVYYYLGSEFLMIILCSHLSTEFILLQQDIQAYKEMPGNRSISHFNEQERSAIDFEPYVNEKTELKALIRRHQILIG*

>ScinOR36

QQDGIINAYINRSRKVTYFFFALAFFSNFSIFSEPYQKNQISENGTSIYLYLFDGYTPFAREPPGYYFSMGVQTVVGHMMSACVVSWDMLVVSIMIFFAGQLRMSRLLCKRVIDLNNAEMSHQNIANCHQFHISLIKNQAEFNNLISPVMFTYLIVICTSLGVCIIQIAEIENDFPAFVAGCLYIGACLVQLLLFYWHANEVTVESEMVRYSTFESNWVDANKHIQKEIALLTQATRKKLTFRAGPFNEMSLTTFIKILKASYSFFTLLKSTN*

>ScinOR37

PQIWIGSLYSCYYVAFWILQLRETAFIMSTLHVMHCLRDINRQLKALLRELTLGNELPLLSLFNSSFCKNVKRNPFKSSFTRAISDAGILTSGVAQPRSQCARVRRLTLAYARTCDVVQDMDAANGVIVLMMLGSFLLHLVCTPYYFIHYMSRDPELAKTPVKHALQVSWQLLWCLYHGGRLLMVVEPCHLTRMEMETTREVVSHLMRCPASVYDPLAIELDLFFKHLLLNQASYSAMQATVISRSLVATILGSITTYLVVILQLQ*

>ScinOR38

VYLSSPKLRDLIRNGTTPCPEIINAWLPFDRTHGYGYWISVLLHSCCCVYGGGVVAGYDSTAIVLMSFLNGQLEILSKNCARLFGVGETVTYEEAVKRIGACHEHHVNLVKFSSTLNSLLSPVMFMYVIICSLMICASAVQLTMEGTETMQRIWIAEYLTALIAQLFLYCWHSNEVLCMSNNVEKGVYTSAWWSQDVRIRRSVILLGGQLRKTIVFTAGPFTNMSVSTFVAVMKGAYSYYTLLSKKEN*

>ScinOR39

YYWAIGEAKLILMIDLVYPNYIEENLDNIYVYLAMYAWILFYDLYATIMYIAFVPLGPVFLIHACGQLELVETKIQQGLFLGSLEDTGRKLKEVAQQLQYVYCFVDQILDVFQVIYEFTLRGTTILLPVTVYEIIEALNKGDLPVEFISFIAGGLIISSSPCYYSDLLMENGEKSRIAMYSCGWESVPDRRIRSTISIIMLRAIQPVALRTLFRTVCLETLADVLQQSYALFNLMNSMWK*

>ScinOR40

QFQVYYKPATCIIDGVMDTILAAFIASAIGQIKILAFNLRNFDIMAERKRSRAIRARENNGSLAKDFYIKEVLKDCIKHHNCIIRYVSMIESAFSVASALQFMLSVMVLCLVGIQFLSIENPTSHPMQIVWMAIYLTCMLIEVFILCWFGDELIWKSMDLRLAAFQGPWLKIDRRTCMLVIIFLERCKRPLRVTAGKIFTLSLDTYTVLINWSYKAFAVMRNMKK*

>ScinOR41

FCGIFGLCLFFDSYAFLVYLGFEPLIPIFTLHVCGQLELLSLKISKIISESETEQEIKEKFRTINVKLQELYRFIDEVQNDFKMLMEFNMKTSTFIVPCNAFQIVQEFRVNGNISIEFISLLIASLIHFLTPCYFSDILMESSERFRQAVYCCGWEKCRHKSIRKTVLLMLIRAGKPFSISTIFYFISLDTFSTMCRQAYAIFNVMNAAWT*

>ScinOR42

FWSAWLAIFFVYGPDCFYYLACAFVYGQFYSLQFDMSKIGPSCFVDQTRFNIYMGKLIHRHRELIRCVKMINVIFGKSSLYNVVSSSFLICLAGFNVTAIDNLILMTPFAVFVVVTSVQIFLLCYYGDLLMRASMDISNAVISSEWYKLNTSSMKCCLFIVMRAQQPCKITAFDFRDINLSTFAKILSSAWSYFTLIKTMYSKQNSK*

>ScinOR43

AYPGLQSIIILLVGQIIRQLRILTFVLLHLEQIGLDIAGDERELWQRCCTAIFAQCVAHYVKIKRFSNKLNVICRPFYLSLILIAIVLVCMCSVKIAISSKLSGDTMKYYIHEFCYTLVVLMFCLLGQQVDNECKQLQEAVTEKWYIYDQKHKKNVLIFGMALSQRMPIYIFGTISLSLPTFTWFLKTGMSFFTMMISVLEQQ*

>ScinOR44

FDKHTQNKINAEVRKGLVDIMQRHARIVTLLKNIDDFFNVPNAIAFVSLAIALTAELLGGLENTYLQVPFTFFQVAMDCYIGQSVVDASVAIEEAVYACKWENFNKENMKIVLVMLQNSQKTLKVTAGSMAVMNFTCLMSIVRSVYSTYTTLHSTMRQTL*

>ScinOR45

ELIRCVQMINVIFGKSSLYNVISSSFLICLAGFNVTAIDNLILMTPFAVFVVVTSVQIFLLCYYGDLLMRASMDISNAVFSSGWYKLNTSSMKCCLFIVMRAQQPCKITAFDFRDINLSTFAKILSSAWSYFTLIKTMYSKQNSK*

>ScinOR46

MGEEIVIPEFKPFRDTYKLITFALCVGMIYPNPNTDKCRVISVLFILITVAPLAITIFIDMYFSWLDRDIVNIIRHSTVVGPFLGGFFKMILMFHKRVRAKQILDEIDEDYESYNHLTREYQLIAARAVHNNLIYSERAWVI

>ScinOR47

LLLLTTSYILVCSSRYPYDTSQSPAYQLTFLFQILAVSFVASLNVSTDQLVVISTAVCRCRFQLLNMSLRTLCQGIKVTDELITLEEEKLVTRRLRSCVLQHQAVLESAAQLQDCFTTSILGQFTISIVIICVTAYQLAA

>ScinOR48

FAQDLDDIFNKMIFVNLFSATISICFFGFSAKVSHDAVSLINNFVAVVASMLPVFNLCYYGELLKEASEGIAIAAYDSFWYNGSKGYAKAIWFILKRSQKPCCLTSLKYNPISLSTFTTVMSTTWSYFSLASSLYDEN*

>ScinOR49

RTAHTLQDIFGGAILIQFGIGGWILCMAAYKIVSLRVLSGEFASMTLFICCILTELFLYCYYGNEMSVESARVVQAAYCMRWARTPARFRRALLVLMERARRPLRPAAGRVVPLSLDTFLKIIKSSYTFYAVLRQTK*

>ScinOR50

KSNGRIYTDRMVKLAKLLKFLEDLKHPCLGPHIRCLKFLGILHPDTKSIRIRLKLAFIYFTILFFISQYIKCLIHFNVESLKLILQYAPFHMGVVKTCFFQKDHKNWERLINFISTVELREISKKDKQQDGIINA

>ScinOR51

ERLNTGFLNKTVEFFYKLCIICFYSGLPNFWMEALTFPKRIDGHCRTYFRVVTILVYVFILSELASKLTQKNLTTKQESDQMVFMMSHPLLWSYTVSFSRHADKVRIFCKLKKPVTNYRFILLVNNNIYT*

>ScinOR52

NACCLFLLTNSIFRHDAKAGNYADASKNFAMLIICFNSNIKYYTMLYFQESIAGLIRTIDTDYELAKQFPEEEKNIVLEYSKKANKVCKFWIFVALFTSSIFPVKAFYLMTYYYFKGDMQLVPMFD

>BmorOR1

MLLSFKDDSRSPDIQKPQNFQYMKILRFNLKIICAWPEKQLNEIRSLGHSIHRVILPIQSVVCLACGILYIHFHFNEIPFFILASTFITVMMNLVTCSRTALVMLFERYLVLTGRFITVMHLFNFQKNSDYAYKLCTFVNRMSHFYTLYVLFSMFMGLGLFNLLPLYNNYVSGAFSDPYGPNVTFFHSVYFAFPFDYSHNFRGYIIMALFNSYVSVTCSIGLVMFDLLMCLMVMHVWGHLKILSHNLINFPRPKASHVITTPNGPTNVETYTEEESKEVFARLRECIKHYGTVDDFANDMSETFGVILLVYYGFHQVSLCMLLLECSDLSTKAMLRYGPLTLIMIQQLIQISIIFELLGSVADRIPDAVYQLPWECMDVKNRRVVYGFLRRTQNPVRFKAMGMLDVGVQTMASILKTSISYFVMLRTVAT

>BmorOrco

MMTKVKTQGLVTDLMPCIRLLQAAGHFLFNYHADTSGMNMLLRKIYSSAHAVLIVVHYICMGINMAQYKDEVNELTANTITVLFFAHSIIKLAFFAFNSKSFYRTLAVWNQSNSHPLFTESDARYHQISLSKMRRLLYFICGMTVFSVISWVTLTFFGESVRMIASKETNETLTEPAPRLPLKAWYPFKTMSGGGYVFAFIYQIYFLLFSMALANLLDVIFCSWLIFACEQLQHLKAIMKPLMELSAALDTYRPNTAELFRVSSTDKTEKVPDAVDMDIRGIYSTQQDFGMTLRGAGGKLQNFNAENNPNGLTAKQEMLARSAIKYWVERHKHVVRLVASIGDTYGTALLFHMLVSTITLTLLAYQATKINGINVYAFSTIGYLVYTLGQVFHFCIFGNRLIEESSSVMEAAYSCQWYDGSEEAKTFVQIVCQQCQKAMTISGAKFFNVSLDLFASVLGAVVTYFMVLIQLK

>BmorOR3

MIFVDDAVIGIKDPREYRHLRVLRTSLRLLGAWPGHYLGEETGSKYECAPMFLLMFIKIACLYLTIVYLRNNADVLGFFELGHVYLTIFMTFVTLSRGFSLTWNPNYHKVVKKFITEMHLLYFKDNSEYAMKTHRRVHKISHFYTVFLKVQMIAGLTLFNVIPMYNNYRQGNYASDRPANITYDLSIYYETFDILNTPNGYIFICVFNWFASYICCSFFCSFDLILSLMISTVSGHFRILIHNLLTFPLPEAITASKKFVDKHRCNGNRSEFVLEEAKLYSPAEMWQVTDRLRQCIDYHRKLVEFTGDISEAFGPMLFVYYLFHQVSGCLLLLECSQLNTAALVRYGVLTVVLYQQLIQLSVIVESVGTVTGRLKDAVYEVPWEYMDTSNRKTVAIFLMNVQEPLHVNALGLAKVGVQSMAAILKTSFSYFTFLRTVSE

>BmorOR4

MFKIIKNIIVENDALKQVEKPQEFQYMKWVQYHLKYIDGWPNMDMNKKNVSKIRFHKRHLLVVEQTITFLSQMFYIVKNYGKLSFFEIGHSYITALMTIVIFSRSVVTALGRYRKIARYFVSSLHLYHYKDISEYALQTHLLVHRLSHYYTVYLISLVVTGMLLFNITPLYNNISSGVFNSPRPENMTFQHAVYLGLPFDYTTDIKGYFVVFILNWHLSHIAASYFCTFDLFLSLLILHLWGHLRIILNNLKTFPKPYTNNSMYTEEENQVVLLKLQECIRYHNFIISFTVMMSNVYDVVIIVYYLFHQVTGCLLLLQCSTLDWESLSRYGPLTLIIFQQLIQVSMIFEILGFLSDKLPNAVYSIPWEAMNVTNRKLVQVLLQKSQKPIQFKAMNMMSVGVQTMASIIKTSISYFIMLRTIARD

>BmorOR5

MLLYYPNTQVKEKVNNVEEFTYIKFLKSFCKIMDFWPEREEKNSKTRIFRLRYILVLQFCFTLVAGVLYLTNSVGKQTFYDLGHTIITVLMNVVSLSRLILRCFKKYDVVGQQFINKIHLYHYRNDSEYAMKIHTVVHKISHNMTYIFSFCIIFGTVTFNLTPIFNNIGSDAYKNPRPDNVTLQQCVYYALPFDYTGNFKWYLLVAIFNVQKTFFCTSLFILFELSLSLMIICLWGHLRIFIHNLNHIPAPRNSFEYTKEERQEVDDTLKKCIQHHTLIIGFVRIMSETYGLAVLIYYAFQQVVGCLLLLQCSQMELKTVTRFGFLTLVLNQQLIQISVIFELLGYMSDKLQDAVYCVPWEYMDTSHRKMVYMMFRQSQIPLQLKAMNMLSIGVKTMVSILKTSVTYYLILKTVTTD

>BmorOR6

MKEEYYLQHPRTQLFYKVLAHVSTIESTIDLTWWGYTFPKYVGWFYHLQCNVVRLFGKCVVVSQILFIILNYQTIDKSVFIIAITITPLGALVGIKAESAKAECYVNLMKNFMDKVHIHSIYRKNENNEFVKKKVIQIERVSRFTAYFLVILIAINCLSWMLKPTLHNIKHFEEIMNKSMEFQYYIYFWTPLDYKYNLRDYIIIHTLCIYLGATAVTVIVTFDIFNFIAVFHVVAHIQILKNNVKSNWSDDFNESEKKGYLVSILEYHAYIIRIFGEVQSAFGLNVASNYLQNLIEDGLFLYQIMNGEKENVLMYGLMIILYLGGLIFLSIVLEEIRRQNYDLCEYVYALPWEGMSLENQKIFVVFLQRTQPDLEFETVCGMKAGVKPAFSIVKSMFSYYVMINSRF

>BmorOR7

MLLYHPNTQVEEKVNNVEEFTYMKFLKSFCKIMDFWPEREEKNSKTRIFRLRYILVLQFCFTLVAGVLYLKNNFGKKTFYDLGHTIITVVMNVVSVSRLILRCFKKYDVVGQQFINKIHLYHFRNDSEYSMKTYKAVHKISNNMTYIFSFSIFVCVVTFNLNPVFNNIGSGAYKNPRPDNVTLQQCVYYALPFDYTGDFKWYMLVAIFNVQKTFFCTSLFILFDLLLSMMIIHLWGHIRIFIHNLNHIPAPRNSLEYTREERQEVDNTLKKCIQHHTLIIGFVRIMSETYGLAVLIYYAFQQVVGCLLLLQCSRLDLKTITRFGFLTTMVNQQLIQISVIFELLGYMNDKLQEAVYCVPWEYMDTSHRKMVYMMFRQSQIPLQLKAMNMLSIGVKTMASILKTSVTYYLMLKTITANEA

>BmorOR8

MSLSTRCLLKDFCKYVYYAGAGNFWYEDIYKETVPYKMYVVISFFTYTVMIFLENLAALFGKLPEVEKNSAVMFAAIHNIVLTKMFLLLYHKRSISKLNCEMAAVGENLEEASIMRRQFRKMRLGTALYFISVYLSLVAYGVESARRTIVEGAPFYTVVTYLPDYDNTTVLASFLRIFFYITWLYMMLPMMSADCMPIAHLITMTYKFVTLCRHFDQIREKFQINVKIMAKTEATEILKLGFIEGIKMHQKLMYLADEIHRVFGIIMALQVCESSAVAVLLLLRLALSPHLDLTNAFMTYTFVCSLFLLLALNLWNAGELTYQASLLSNAMFYSGWYFCDFEKDWCRDIRRLVLIGCAQAQKPLILKAFGVLDLSYETFVSVARMTYSVFAVFYKRGD

>BmorOR9

MVARRPLQFHQGRNVDNVEDFKYVKWLRNHLKTVDAWPVHSKSKRKIQKRYVLPIFSAACFISQTVYLKNGIGTLSFVVLVHSYICFLINGSCLCRGILIATERYKRLATCYLKTVHLFHHKNRSEHAMKIHVIVHRLSHYYTIYLISLVFVGMVLFNFMPIYNNINSGAFKSPRPENVTFQHAMYLALPFDYTTNIKGYFVVFILNWYISLVTTSHFCTFDLFISLMIIHLWGHIKILMCSLEDIEGFVPGSSFKFTIEQNRKIYLILQECIRHHQFTIDFTNEMSSTFGLVILFYYFFYQVSGCLLLLACSQMDIESLSRFGPMTFILFQQLIQLSIVFELISSLSENLPNAVYNVPWEFMDKNNRKMIQVLLLQSQKLIQFKATSMMNVGVQAMATILKTSVSYFIMLRTMYQEH

>BmorOR10

MRTNAKSFLFVPSKVLTLCGVWPVEKTSIFSLIYRSIMLSSQFCFLVFNGIYIGLMWGDLKAVSDALYMFFTQTTCCSKAIGFYFNFMKIKRIVASMDDVLFTAMSIEDQATIFSHSRTVNKLYKGVLGFTGFTLVQWTVLSLIGSGRTLPFNEMWVPTDISKSPNYEITFVVELWMMVISAALFMSVDTITVATMMFSCAQLDIIMKKTQQIQEIPLSPDLSSRNRSELHEKNNGILIDCIKQHQAIVRFSELCEGTFQVHSFFHLGGIVFMICVIGFRMAGESPVSAQFWAALSYLVIILGQLYLYCWCANELTTKSEQLRDKLYLTPWYDQDVKFKRNLCIAMECMAKALTFRAGSYIPLSRAMFVSILRSSYSYFAFLNQANEQ

>BmorOR11

MDEHSHFETSLNKIKVLFKYSGMNLENTVTNTYEFLNHRWVYILNHAWTLAAVTFICIGISNGQNFIEMTCIAPCVAMTVLAVSKSFFHYINENAVKSLLENLIELERTDFERTKSVQRTEIVATEKQLLNMVINVLYVLNCSMILVFDMTPLIIIAIKYWTTNKFVRLLPYLDIFVFVPYKFEYWVMAYILQIWAECIVLLFIGAADCLFFTCCTYIRIHFRLLQYDFERLTSSRRESDGLRDDEDFRETYTNLVKRHQGLIESSSILEMIYSKSTLSNFVLSSLVICLSAFNVTVVNDVTIVMTYLIFLAMSLMQVYFLCFFDMLMSASEEVGNAVYNCSWYTEKASTGKDLLFTITRAQKPCELTAAHFAYVNLKAFMRVSFTSASITTLPTI

>BmorOR12

MTRITDVFSLNFIFWKFLGLWGKSAPSKYNMAYTVFYLFASLFVYDIFLTLNLIHTPRKLETLVRETMFYFNHLVAVTKILMMFIMRKKILVIFDLLDCEEFKPNDENSQEIMKRKTDFYYIYWRIVAVTSNLSCFMLVIGPLIKMLIWKIELGLPVCKFYFMSDELRNKYFVIWYIYQSFGIYNQMVNNLNLDTFNCGMLWMAVGQLQILKTKFVNLKLNDFENGLDLKSRDDMQIERLRKYLTHYEIILKYCAIVQDILNITIFVQLGMSSIVICVGLCGFVAMPSNTETAIFMFSYLTTMTMQIFVPSWMGTQISFECGELMSAAYSCEWIPRSKLFKRSLILFVERAKTPVRITGLKIFTLSLDTFTSIMKTTYSFFTLIRQLQVDEVN

>BmorOR13

MAPKQIDCFEINWKFWKFLGIWSENKPHRYYKYYSKIFITFFVILYDVLYTINFYFVPRQLDLIIGEMLFYLTELSVLSKVFTFIIMRHKLKIIFEILESDAFQTDTEEELKILHRAKVFIKRYWKIVALVSITANLTHISSPLLKNLIFKVELVLPVCSYSFLSESFLKTFEYPLYFYQIVGIHFHMLYNLNIDTYFLGLMILIIAQLDILNVKFRNLKSGKDHTQLNESIMGLNKNLDHYNEIERFCSLVQNIFSFTLFVQFSMASCIICVCLFSFTLSVPVEYYIFLATYMFIMIIQIMVPCWFGSRIMDKSILLSSAIYNCDWTSNSKDFKINMRLFVERANKPLSITGGKMFSLSLATFTSIMNSAYSFFTLLRYIQTRE

>BmorOR14

MSNYIFKPFHETYRIITFTMIAAMIYPNPATEKRRLIYIGLMLLSVIPLAFMIVTEMYEFFMASDLNNTIRHSTVIGPFIGGFVKVALMYYKRRQANELVSEINRDHLAYNGLKGEDREIAASSIRNCQIYCELGWTLIVMSCGLSFPVIAILLKIHSFTFKLDSTKHMIHDINNPFTDDPEDRFESPFFEIMFVYTFFSSFIYIINYVGYDGFFGLCINHACLKMKLYCRALEDAMRSDSRRHEKIVAVIEEQRRTYEYIALIQDTFNIWLGLIYVATMIQMCTCMYHIVQSFNIDVRYIIFVISIIHIYLPCRYAANLKCMAAETPTLIYCCGWESVSDLRIKRMMPFMVARSQVIVEITAFNMFAFDMELFVWIMKTSYSMFTLMRS

>BmorOR15

MMTLVYQTDIFKPNVFFWKMFGIWADRKSSKTYKYYSFVFLFITLIMYNSLLAINLLYTPLKIELLIREVIFCFTEITVTTKVLMILFKRNKILDAFDLLNKNEFRGNSEESSAIIQKNNSAYKTYWKLYAILSNFAYSSQVLGPLIVKLIWKTKLELPICNYYFLNEELRHDFFSGWYIYQSFGMYGHMMYNVNIDTFISGLLMMAVTQLKIIQTKLLSLKLNPRERKMDRGLMNITEVLKLNEILKHYELVLKYCSTVQSILDVAMFVQFGVASAIICVAMCGLIMVRSSTETLLFMVTYLFAMTLQIFVPAWMGTQLHFQSQELVFAAYNSEWIPRCQSFKRSIIIFVERAKIPITITGLKMFPLSLATFTSIMKTAYSFFTLIRNMQTLQEE

>BmorOR16

MSFNSEDLYLNRAKFVMKYLGVWVPPENENFARKFYKIFMMSLQHLFLFFQIIYIVEVWGDLEAVSQASYLLFTQACLCFKITVFQINMNKLKELLKQMNGYVFQPKNINQQNIIKVQATRIKRLLFAFMISSQLTCGMWALKPLFDDVGSRKFPFDMWMPVSPERSPHYHLGYSFQLVTICMSAYMYFGVDSVAFSSVIFGCAQIGVIKDKIMSIKPLGIYRNHKTYTKISRYNRKTLIECVKHHQAVISFTELVEDTYNSYLLFQLVGSVGIICMSALRILVVDWRSVQFFSILCYLSVMISQLFVCCWCGHELSATSEELHTILYNCAWYDQDVKFKRDLNFMMARARRPILLRAGYYISLSRQSFVSILRMSYSYFAVLDQTNK

>BmorOR17

MREDKMEINNSQKFYTKMIFRYLYSVGLGDWWYQHEDRSDSHRKLYCLWAVISNAYIFLNICNELLANFRKDLTDVEKNDAIQFSFAHPLIFAKIASFFFNRKKIREVFGRLLEENRSVYSCGELEKESMKQIKRYSLAFIGVSYMTLVMSTIDGLRAHFKEGIPIRTEVTYYPSPSNSGVIVNILRFLVEFHWWYIVSVMVAIDSLAVASFVFVTFKFKLLQRYFKDMGLTVRRDQSNMTDEALADKFRRDFIVGVKLHENALWCAENVQKAFGWVYSVQVFETVALLVMCLVKLVTTNHNMIFLLANFAFMLCVIILNGSYMMPAGDVTYEASEVPTSIFLCGWELVRQTDLRFLVVVAIQRSQVPVIMKAFGIMTLSYSNFIAVSLFKFYVQFQINLF

>BmorOR18

MGDRMVTRGHFFDFNIKYLFYVGLWPSNEAKRIEKIAYKIYEYQLHVLSLIFLVTTGIGTYKNHKDIIALLTNLDKTLVAYNFVFKVIVFVYKREELRKLIEQIVQSGDQITEDRKALMAKLVIVLTGISTVIITAFSCLALFEGEMTIDAWMPFDPMKSKMNLFAASQILAATFVVPCGYRAFAMLGIVCSLILYLRDQLVDLQNKIRDLRFATGNVEKLRDDFKLIVKKHVRLLGYSKVIEMIFKEYFFIQNMAVTAELCLNAMMVSVVGLEQKTLAASFLAFLSVALLNAYIYCYLGNELIVQSEGIAMAAYESSWILWPVDMQKDLLIVITAAQKPMKLSAGGMAVLSVQTYSQTLYNGYSIFAVLNDIVN

>BmorOR19

MHEFVINVQNETTKLYDQLNIILYILGLQGIWVDEIKLSRRFHVFFKVVTFILHIMCGMFAGLQFFAIFTQNSLNSQQKSDVIVIGISNPMAYIFCINFIRNRNEIKDLFYHLAVVLKIYYNDVEIEKSMVNKIKSYLSTYVFASITILVSNGIIAFFQTINSDEPFLGIITAWPDKTDTSKTASYARIGFYLFWCIHFFRISTVFAVIVCILISIKYQYKFLCSYFESLNKIFDDETSSHEVKEAEFENAFCNGIKIHTQIIWCVRRCQIMCRTVFSANIMLDTFVLVILMLAMVNSENDFYGLCSQMSSVLVTVVLMAFFMWTAGDINVQASQLPDAIYGSGWYNCRGKSSARIRSLVTISMNKAQQPILMWALGFVELSHKNFVAIIKSAYSVFSVFY

>BmorOR20

MIQASKYPNSKTKELFRKIAHIAYICGLPNFWIEELNLPKSFIRVYDKIVRIFNVATYFFLGIEIAAHFTQHHLTNKQKFDLLLYSISHPILNGYGVIVSRQVGNVKKVLLDLIVNLKVKYNDPVIEEAMIKISMTYSVSFITNCVLSMLTYTFDALLMVYKKGVTFNVIITAWPDVEDTTTEASIGRIGFHIFWWLFVTRPFAVYVLVINLTTCLSHQYMNLQSYFFHLEDIFKENLSQNEKEAKYEAEYKIGVMLHANTLRCTRRCHMVWNGVMSGQIIFNISLIVIIMAQMMNSDRTLVNTFGTVLTASAILISTGFFMWNAGDVTVQASRLATAMYCSGWQNCRGKSSVSIRNMVMNTIAVAQRPLVLRGLGVIDLSYQSYLSIVKASYTVFSVIY

>BmorOR21

MNKNHYILKTYCDKIFLVGSGNFWYQKTESRNDKTLLYKIYSCVLFFTYGFMTVLEIMAAMMGDFPEDEKRDSVTFATSHTVVMIKFISIIKNKELLKTLNRKMMMICEAHEEQTLMDEMYRTVKINVVAYCVAVYGSATFYVFEGLRKFYNGSHFVTIVTYYPSNDDDTLAATIVRIATTLVLLMMLLTMIISVDTYTMAYLIMYKYKFITLRHYFKRLRENVDELVAAGKARLAAEKLAQGLVEGIKMHNELLSLSKDIDKAFGTVMALQLCQSSGSAVSLLLQIALSDQLTFTMGMKIFFFLAAMYLLLALFLCNAGEITYQVCTSIV

>BmorOR22

MNKNHYILKTYCDKIFLVGSGNFWHQKTESRNDKTLLYKIYSCVLFFTYGFMTVLEIMAATMGDFPEDEKRDSVTFATSHTVVMIKFISIIKNKELLKTLNRKMMMICEAHEEQTLMDEMYRTVKINVVAYCVAVYGSATFYVFEGLRKFYNGSHFVTIVTYYPSNDDDTMLASIVRIATTLVLLMMLLSMIISVDTYTMAYLIMYKYKFITLRHYFKRLRENVDELVAAGKARLAAEKLAQGLVEGIKMHNELLSLSKDIHKAFGTVMALQLCQSSGSAVSLLLQIALSDQLTFTMGMKIFFFLAAMYLLLALFLCNAGEITYQASLLSDEIFYCGWHKCNSPVLSTQRNIRDIVLIAILRAQSPLVMKAFKMVELTYATFILVVRSTYSVFALFYAQNK

>BmorOR23

MRAKTEFEKTIKLTKTALFLSGINIFLGEWNHWTRTFVDSIAYYLNIVGLYFVLIGEMYWLIDGTITGKSFVELSLIVPCLTISVLATAKVHYLYHNKESLLDVVDKLREIYPDEIEETANDNDQCLNDKKETVYDNDVTEVGIVNEANELLKFVNFLLSTVSFVVTMTFCTMPLFGMAGEFMETGKFVVLYPFAVKYPFDVYNTSFWVIVYVNQFWATIIVCTNIFGVDTLFYALCSYIGMNFRLLSYKFEHLEIKRNDRIINEIIVLIKRHQELIELVNKTQSLYSLSTLFNIVTSSLLICLSGFNITILSRSWSYFALLKTIYS

>BmorOR24

MPEELFLDRSIKKIESYFRWMGINIRSGDNNNKKDVFKIRCIYFINFVLLNTDVLGAIFWFRSGLEQGKTFTEVTYNAPCLTFSFLANFKMLSLIFYEKTVHELIAALQKLEIKHFLRQNCAEELKMLKDEKNFLHAVFKGSKIVNYASILTFGCSPLVLIASNYYKTGRMDYLLPLIVLYPFDVDNITVWPIIYVRQIWSVITAVIGVCATDYLFYTFCVYISTQFRLLGHSIERVVPNNGLSVRTRLNGNLRMKFVENLKWHQELIRAASLLEQIYTKSTLYNFVTSSVIICLTGFNVAVVEDFAVILSFLFFLFMSLLQIILLCFFGDKLMKSSTNISDAVYNSKWYLTEKNVGKVLLMVQIRSQRACRLTAYGFAEVNLRAFMKILSTAWSYFALLQSLYSSHE

>BmorOR25

MFEKALRSANFYMRVIGIPTDIRDGNRTLMERLRNRWFYCINFLWLNTDVAGEITWFVKGLLSGSSTLIENTYLIPCLTLCILGNVKTFFTIKYANHIIDLVAILKDLEIKNNAARKNETEIVKERLKFLTTSNKFLLFVIGTGIIAFGIGPLMLTASIYFSSGDMKLKLPFLIWYPFDSSDIRYWPFVYVHQVWSACIACCAVYGPDCFYFTSCTFIHIHFIHLQNDITNVIVESSRARRNGLYRGCHQAFLELTNRHKDLIRCVNLLEIIYSKSTLVNVVSSSLLICVTGFNVMAIDFLPLIAPFTSFLALGLVQTYLLCYYGDTIMCSSTEVSDAVYNSTWYGTNISQMRDYLFVMKRAQKPCKLTAYGFSDVNLRTFSRILSTAWSYFALLITIYRGNGQQ

>BmorOR26

SLSGSSVFTHLFLLRCCGFCRLSRSSTARRGLSVAHEVYRALTLTLTVVYLLQECVYAYQERTDMDKLSRVMFLLLCHITSVAKQLVFYLDADRIDYLIATLDDPSYNEMSHQRLLVDASRWASRFVWAYSGCAVVTCTLWIVFPIIYHVQGQTVEFPFWIQIDYTKSSMFVVVLLYSYYVTTLVGIANTTMDAFMATILGQCKTQFTILRIKFETLPTRAKQALRCDSEQNYDEVLMRLFHDCLKHYQKIVSAILIQFGIGAWILCMAAYKIVNLSVLSIEFASMILFISCILTELFLYCYYGNEVSTESERLVTSIYSMEWVGARLGFQRGLLVLLERARRPVRPAAGLVIPLSLQTFLKIIKSSYTFYAVLRQTK

>BmorOR27

MPSSFFLPNLENPDYPSLGPTLKGLKYWGMWQSGGIKRILYNSIHAFATFFVITQYVELWIIRNNVELALRNLSVTMLSTVCVVKAGTFVCWQKYWSGIIGFVSNLEKEQLSKNDAATQAAIVKYIKYSRRVTYFYWSLVTATVFTVILAPLVGFLSSPERELIANGTLPYPEIMSSWVPFDRSRGFGYWVTALVHTLICFYGGGVVANYDSNAVVLMSFFAGQMKLLSINCSRLFDDGNEVISNNEAMKRIKECHYHHVFSTIFNSLMSPVLFLYVIICSLMLCASAVQLTTDGTSNMQRIWISEYLMALIAQLFLYCWHSNQVLYMALEDRLGGLFEACLESGRFPSKWKTGRLVLLRKDGRPADSPAGYRPIVLLDEAGKMLERIVAARIVRHLTETAPDLSAE

>BmorOR28

MHTLALVFALLYPSNCNIIKRAIGITLIIALSGGQLFWCMTYTFNVCVLILNYSGFDGSFCIASIRLCMKLKLVVYKVQKAFAESKSVSELKHQLNDAIKDNLDALKFHEQIQNVFFIALVGRRAYGPPDGEWLPSPMDFSNTRGRTKPLSTVYEPWLFLIFLLTFLII

>BmorOR29

MFDFLQNLEDSERPLLGPNFWLINKTGLLLPKTNFGKLAYILVHEIVTFFVVTQYVELYVIRSDLDLVLTNLKISMLSIVCIVKVNTFVFWQTSWREVLEYVNEADKFERNQTDETRGKMIETYTKYCRRLTYFYWSLVFTTFLTTTNTPLMRYWSSPIFRENLRNGTEDFPHIFSSWMPFDKNHSPGSYCTIVWHVLLCAYGAAIMAAYDTCIVVIMVFFGEKLNLLRERCKKMLANDLYNHAFVIGQLHDIHVQLIKQSRLFNSLLSPVMFLYILMCSLMLCASAYQLTSATSTAQKLLMAEYLIFGIAQLFVFCWHGNDVLFKNANVSLGPYESNWWSSSPRVRADVLLLCGQLRVRHVFTAGPFADLTLSTFIKILKGAYSYYTLLRK

>BmorOR30

MSVSNLKFEVLFKPTTMSLHMNRSHPSIKRNKIWLLQFISLMTLTAFCATGLITSLLFHDLKFGKYMEASKNGTIAMLSFTTTFKYSLLLYLQKSLNRLIAKIDMDYEIAKGLTPQEKVTVLNYAKKGVIVSKFWLFTAFAITFCFPLKAFIIMGYRFIIKDEFRLEPMFDMTYPEPIESYKTSFPVYFILFVVCFLFGCYASSLYVAFDPLVPIFVLHACGQLDLLSVRITKLFSDTKNPRIIAKELKVIIIKLQELYSFVNFIKVNFSILYEYNMKITTISMPLSAFQVVESLRRGEFNIEFTYFFFGCILHFFMPCYYSNLLMERSENFRFAIYSCGWENHNDKNIRQMLLFMLTRAAEPLGIATVFTNISLDTFAEVNTFDTVLLA

>BmorOR32

MTTRHAEPCAEAPRLSPAAGGMVGLAPVPQPSSNEMLVQERGRPPGLEGEYVANRPFRKSLRGSQEEPRANGKSENVRFLINSHILHCGLRFNETNCHTHYIAKVAIFCFIVTYMLQVMELYWSKGDQEKLFECFSILSFCGMGVMKLVILRVYHQRWRFLLNQVSILENRHLDPGPLSYDSDNDNDDNEIVTFITKYTDKFKRTSSILIKMYASTLVIYVLSPFVEYIFRQFRGDLNIAYPHILPAWTPLDEFSVTGYLIMVSFETVACIYCVFVHVAFDLTCVGLMIFACGQFYLLRYRSERIGGKGRICRLLKSTEVRAHYRIVFCHGIHVLLVQLIEELDRLIKHILGVYFFLATLTLCSVAVRLKTEDMSITQLVNLLQYMCGTLTQLFLYCKYGDSVYNEADEPYGLPDACLESGRFPKQWKTGRLVLLRKERRPADSPAGYRPIVLLDEAGKLLERVVAARIVQHLTGVGPDLSAEQFGFREGRSTIDAVMRVRALSDEAVGRGGVALAVSLDIANAFNTLSWSVIAGALQYHGVPAYLRRLIGSYLEDRSVVCTGHGGTVLRFPVQRGVPQGSVL

>BmorOR33

MELNFDKIFKIAIISQKFSGTYPYTKRDKKWATHFILMHGELTIICMLFIYNIIEFDLKAADYSQMCRNMCLSFVYLVITLLYINMLYYQSKLKMLIETMKAEYELAKTMSEEEQNVILEYAKKGRWLCRAWAILTTCGMAQFFLKSIVCTIYSAIQGNFRIVQYYEVICPEVIERHRNNPVIFITLYFCTFFYSLYTSALYTSVLPLGPIFLLHGCAKLEIVRLNIKNLFDNDDYVVQERLKKTVLQMQDIYCYSHEINECFQILYEFLLKATSLVLPITIFAVIQALGRGQFIPEFFAFIFGAFMVGTTPCYYSNMLMEK

>BmorOR34

MELNFDKIFRIAIISQKFSGTYPYTKRDKKWATHFILMHGELTIICMLFIYNIIEFDLKAANYSQMCRNMCLSFLYMVITLLYINMLYYQSKLKMLIETMKAEYEIAKTMSEEEQNVILEYAKKGRWLCRAWAILTTCGMAQFFLKSIVCTIYSAIQGNFRIVQYYEVICPEVIERHRNNPVIFITMYFCTFFYSLYTSALYTSVLPLGPIFLLHGCAKLEIVRLNIKNLFDNDDYVVQERLKKTVLQMQDIYW

>BmorOR35

MVVLSLGISSEIGTLKFFYTFIYIKKVQRIVREYLECDHMVVPGSRFADNVLKTMRNVKKRAILYWVVVIGNGVVYVTKPLFMSGRHHMEDRYIVYGLEPMFESPNYEVAYFLMMFGLCFICYPPANVTVFLIVVVGYTEAQMIALGEEMLRIWEDAVAHYNNKYHTVGALTNSSEKNKIINQYVKFRLTEIIKMHTTNIQLLRQVEFVFRSAIAMGYVFLVLGLIAELLGGLENTYLQIPFALIQVLVDCYTGQKVMDASSLFEQAVYDCKWENFDKSNMKTVLLILQNSQKSMRLSVGGITVLGFSCMMSVMKSIYSAYATLRTTMS

>BmorOR36

MVFNSKKNIISLFSLLEDSRHPSVGPHLRLLSLTGIWYPNSKTNITLLKRACFYVIVLFFVSQYLKCIIKFKIDSLQLILEYAPFHMGIVKTCFFQKDYNVWQDLVSFISKTERDQIAKKDPKSIKTIQSYISRNRKITYSFWALAFIANIGVFSKPYQNNQSDVNGTVTYNHLFDGYTPFSEEPPGYYFSMGIETILGHVVSFYVLGWDTLVVSIMIFFAGQMQMSRLQCSRMINGSPERTHKNIIKCHKFHTDLIKYQKQFNSLISPVMFVYLFVSSINLSVCIVQIAEIEDDFATVLSSFIFLLACLIQLLLFYWHSNEVTVQSELVSYSTFESNWTSTQNKLQKEVALLGLTTSKTLVFTAGSFNHMTLATFISIIRASYSFYALLNSTKY

>BmorOR37

MELGCSRHLKLPCSLHPIGISKHGNTLSELLIYFPAIPKITYAILAVLLTVYYYIYLCSITWFVFVRCPQTGDLAAASIVFSLGVSSEIGAIKLFIMYVYRAKLRDITGEYLQCEADMAPGRLRARVGRSLRTVRRRAFVYWLVLVVNAFAYDLMPAFLPGRHLSEDVFVIYGFEPMFESPNFEIASTLMGVSVVFICYTAGSISAFLIVIVGYSEATMLALSDEISCVWDDACASECQQPNDFIRARLGKIVAIHTKQIRLIREVEVVFRGALAGGFACVAFGLIAALLGGLENTFLQLPFCVIQISVDCFVGQRLRDANVAFETAVYNCKWEYFDKSNMKTVLLILQNSQKTMGLTAGGVAALDFTSLMTIFKSVY

>BmorOR38

MVVFSLGISSEIGSTKFFNTIIYIKELRKLFKDYLLYDATCPAQGRLRLHLLTTLRYVKRRAIIYWLVIIGNGFIFAIKPLLVEGRHLAQDDLVLIGLEPMRQSPNYEIAYAIMTMGVCFICYPPAHVTMFLIIIVGYTEAQMLALSEELKHLWNDAIEHYEKHSRTEREADAAMKSKILNSFVNFRLVQIIKSHSTNVNLIGRVENVFRGSLAVGYVFLIVGLIAELLGGLENTYLQVPFALIQVAIDCFIGQRVNDANIDFEKAVYDCKWENFDKRNMKIVLLLLQNAQKTVSLSAGGIAKLNFSCFMSVIKSIYSAYTTLRTTMK

>BmorOR39

MLWSVFSYFTRADDVLAGIVIFSLGVSSEIGLVKLCFMYANIDKIQKITEGYLKSDAASARNSRFSKNILHTMQSVKKRGVIFWLVIISNGVVYLVKPIVTPGRHFMEDQFIILGLEPKYETPNYEIGFFMMAVGVCVTCYLPANITAYLITVAGYSEAQFLALGHELANLWPDAQLHCRAMNLSQSVNEQANEYVKMRLRELVKIHSTNVNLLRDIEGAFRGAIAVEFLLLIVGLIAELLGGLENTYMQVPFALIQVSVDCLTGQRVMDANLALERAVYDCRWEEFDASNRRVVLLLLQNAQKVATLSAGGIATLNFSCLMAVIKSIYSAYTTLRTTMK

>BmorOR40

MTGAGAGTFRTGAGPGRGDGVARRGESGETTTLGRDAFAALGCFGAADGSTARARFFPRVTVLNPSEVPGSGLAADSNSISDSESEPELDAAQDAIDAGAGVGGDIGESRARTVFGIQGHDASDSALRMHNNVAIYAKTTMSGNSQLTFATAATIFLKNASGPNGVAIGTDYAICVVSLSLFFCYRFTELVEDTYNSYLLFQLVGSVGIICMSALRILVVDWRSVQFFSILCYLSVMISQLFVCCWCGHELSATSEELHTILYNCAWYDKDVKFKRDLIFMMARARRPILLRAGYYIGLSRQSFVSVSIPRIRFNAILVI

>BmorOR41

MMGNSTDLFLDRTKSILNFFAMWRSFEKPIPLKVYMAFIMTTQYLFLIFEIIYIVNVWGDMAEVSEASILLFTQASVCYKITSFISKTNNFVILLGLIESEIFSAQTELHEKILILKARKIKRLCMFFLVNAVTTCSLWAVIPLLDISSKMLPFKIWMPASTGESPHYELGYLYQMITIYISAFLFIGVDSVPLSMIMFGCAQLEIIMDKIGKVKSRPLDQQPMQRQAVLNSNYELLVECVRRYQSVVRFIELTEKTYHANIFFQLSGSVLIICNIGFRIAIVDSNSLQFYSMLTYLVTMLSQLFQYCWCGHELTIRGEELRETLYQSPWHEQDIRFRKVLIITMERMKRPIIFKAGHYIPLSRPTFVAILRCSYSYFAVLNRVRNE

>BmorOR42

MDIPKFEELLKQIKMNFWLMGIPFDNPKIQIRYYVLLLPLSLMLIEEIAFFGSRMSSENFLELTQLAPCICIGVLSVLKILALTAKRQKIYELTQNLECLHKIILNDTRKTELVRKNLVLIKFITKYFFVLNAVLIFVYNFSSPVIIAYNYIVSNEVQFVLPYAVLLPFKTDSWIPWLIVYVYSIFCGFTCVLYYATVDVLYCVMTSLVCNNFSLISFKLQKVNRNTAHLLKEVVKEQQYVLKLAEDLENIFTAPNLFNVLIGSVEICALGFNLMIGDLTQIPGCILFLSSVLLQILIMSVFGENLISESSRIAEAAFLCKWYEMDQKSKKTILTIMIRSHKPKKLTAYKFSVISYGSFSKIISTSWSYFTILRTMYTPPGTKFQDDL

>BmorOR44

MYTYFKVLVFWLNKDKVISLQKILHCKEFKPKEPEHKEIIRKSIRKARFVMTSYATMCVGAVSVGIILPLTENFDILPTNVEYPFFDVYKNPTYAYLYLHHIYYKPATCIIDGVMDTILAAFVASAIGQIEILAFNLRNFDVLAERRRKRAISGNKYIGKYTNLYFTKRILKECILLHNSIIRYVSVIESAFSLASALQFMLSVMVLCLIGIQFLSIENPTSHPMQMVWMAIYLTCMLIEVFILCWFGNELIWKSNDLRQAAFDGPWRNLNRKTCMFIIIFMERCKRPMRLSAGKIFTLSLDTYTVLINWAYKAFAVMRNMKK

>BmorOR45

MKVLDNVNHAVKVTMNCCRLYGLFVSDDLTKRQLIIMRAFSLMLYLFFVGFFITTQSALIITMWGDLNLMTNVGLVLGTHLTLSAKVFTLHYKEKEITNVIYKNEVRLRAETREQGKYIISEYPCNTTKSPAHEIILAHQGIAVILTATLEIAIVLLMTSIVAVCRCRLKLVGLSFETICDDLPSNIMNKLTADEQVIVAKRVRENVIEHQAVLECINDIQDCFSSAMLVHIAISTMIICATAYQLAVEKSLDLTQRMTMASFLGGMSTEIFLFCYQGGHLSIDSMEVATAVYSCPWYTFPTSLKRSLLVIMIRAQQPALLTAGGFAPLLLDTFVSIMKASYSFFTVLQNASE

>BmorOR46

MAFFIRNKMLGLTITLNTLSWAGLIMRDQYTKTQRIIVRVYGWLVFLYLFVAATYVQIADLIDIWGDLDLMAETSLLLFMELAVISKILTLIFKYDKIMEIINGTEDILCSENRLEGQKIIASIDKETTRFFQYYTSSVIFTTFFWFLGEHSSTFFIRAKYPFNELKSPGYEFALIHQCMMMVFTGYFEFNINIFFASVVAGCRCRLKLVALSLRNICINIPVNKKNLITPEEEKLITERLHCAISQHKYALDAAEDVKHCLSKVLLVQLTVSIVIICTTAYQMAVNKSTDTIQKLSMAGYLLGASFEVFLFCFQGQSLSNASEDIADAVYECPWYTLTQPLKRTLLIIMMRAQSPAILTAGGFVTLDITEYMAVLTGHGGFGDFLHRTGAEPMAECHHCGCDLDTVQHTLLVCPAWKGWRRDLVVKIGNDLSSVLWHRCSAATSRGRRCLTSASAPSRRRRRGA

>BmorOR47

MKLVFDNFISALKVTLNWSRYIGIFIPDELTGRRQKLLVQAYSVFMYYLFIGFFITTQIISFILVWGDLNLMTDVGLVLGTNLALSAKIAVFFFKREDLANILKKNDDTLRSETRAEGKKIISETSCESARVGTTTLPISAVVTILETLELISQASLLITVDIIMLSMIAVCRCRVKLVGLSLQTICDDLPCNVKNKLTSDEEVIVAKRIREYVIEHQAILDCISELQNHFSPALLVQLLTSVVIICVTAYQLAVEKSSDLLRKFTMASFLFAMSTEMFTFGYQGGHLSHDSMEVATAAYSCPWYTFPTSLKRSLLVIMIRAQQPALLTAGGFTTLSLETFVTIMKASYSFFTVLQEATD

>BmorOR48

MEGTSEGDEPFIYRLNDNGTGPNLVVKILYCPCWYTAHLMPFKLHYCFTAEIVRLMIVPLVDSQDVLPPVITQIVISIFITRCYSFIVEVNREHLLMQETLLKPARRDPKIANYNMVRNDRLSARGGGTVIYYRRALHCVPLDPPALANIEASVCR

>BmorOR49

MLTCFATIFSAVNQTGYIVLFINLLAHELGHFYVITDVLNGIFEKNDADRDPVFIDRKLKFCAKHYQYLLKFHNEIKNLYKIIFGAHFLMMTIVLVTTLQTMNSWDIRNTVLTAVTGIMPLFIYCFGGELLITAGMDMSTAIYQCGWEKMGVKQAKVVSVILCLSQRPLCLTAANVFVMNRETFGGIAQVVYKIYAVFN

>BmorOR50

PIFAAKTLKDKQHLIQNKKEVTRFARLLLTYVTVGGFIWPMSFCFRRIKDPNTVVPFYVPFTPDNWTKLINEVTDIFNPCLTFQFFTSSVAICMVIYKLSDTYIVSLEFVFLLNFIFVLLTQMFIYCYYGNVVSYESKYINTSLYLSDWSSASPGVRKMFLIVMPRWTRPLVVRIARVVPLSLDSFVSVRKYKCSIEHFIYSHVKQTRARDIFSGASNKP

>BmorOR51

MDCTIVAFYSQAKTQIKMLRYDLEQLGKIDNIETKFTENIFERSSHIWKALKDEKIKIHSKLVFCVEHYRQIVWFVKEVESIFGEAMTVQFFVMAWVICMTVYKIVGLSIYSAEFVSMGVYLGCMLAQLFIYCYYGTQLKVESESVNTSLYCSNWLSSLPKVRRQMLIMMQYCSKPLTPRTAYVIPMSLETYISVLKSSYSLFTLLNQKH

>BmorOR53

MALKKMLALTKGLEDPTHPLLGPTLKALSVFGLWQTGSQKSTVIYNTFHFLTFLFVITEYIDLYTVRKELSKMLNNLSVTVLSTICMIKTLSYVCRQSHLKVLVREISELELELMKTTDKNIVKRLRQYTVYTRAVTYVYWFLVVGINVVLLTSPLLKYASSEIYRSEIKNGTEPPPLILCSWFPFDSARMPGYFWATMVHIIMSIQGCGVVATYDMNAVAVMSYLKGQTSILKDKCKAIFDETASSRDVLNRIRDCHRHHNILLRHYYMFNSLLSPIMFVYMLICSFTICCSIIQLDSSETTISQRIWIIQYSIGQISQLFLYCWHSNEFAAKVKKKHFPLFPINLF

>BmorOR54

MGLNTIKEFFVNVKRRFQDVSIDSLLWIVNIVPSLAGFSIRSDRVSAPFWIVHWSLLVYVYAVGNAVYQWKFANEAIDYITSFINVSLLILIGNNSWWFLANRRLLKSVLHKIEVNDELSRRSEQSRLKHKKLLKIIKRIVLVFYMSNYVNASFIYLPNRVDVLNNYAMTPCVGMEPLTVSPNRELCLTILCMQEFSIMTVVLNFQALLLCFIAHTAVMFQILADEIMALNNYENLEEHQAYVKEMLPIFVKRHSLTLSAVDNYKSLYSVPLGVNFGSNALTILLILYLPVLEWFKFIPIFVFCFMLFFLYCFLCQKLVNASEAFETAIYCCGWENFALREMKMIYVMLHQAQKPVELLAADIVPVNMNTFATTLQAMYKFVTVVKF

>BmorOR55

MCFLKIKQQIIDIQKHFKDYSLNGSLWIVNLLPRLMGFNLRADKVGVFFWTIYILLLVYVFGIGIFVYLWKHVDTMSGLMKSYLNLSLILVIVNNSCWFLSKRSLLNKVLKKIHLIEDLSCESEHALAKYRRVFKIVTHLLLASYVLFYFTEIYFMFLFRNYDLLEDYSLAPCVGLEPLSSSPNSEICLIIVLIHEFISTTVMMSFAALFLVLIAHTAVMFLVLAEDMTKLTDLINLADHRKMIRESLRSLIHRHSLLLQIVYELRLLYSVPLGINFISNAMSILVLLCLPIHEWPSFLHIIGYCFFAFFLYCFLGQNVINASEKFIDAIYCCGWEHFGVAEKKLVHVMLRQAQKPVEIIALGMISVNMNTYVEALQLIYKFVTVLKI

>BmorOR56

MKLLEKLEDPDRPLLGPNVKALKFWGLLLPESRSKKYFYLFMHFAVTVFTATEYIDVWFVKSDLALLLNNLKITMLATVSVLKVTTFLLWQNAWRDLIGYVSRADLEQRATSDSRKLALINGFTGYCRKITYYYWFLMYTTVAIVTVQPIFKFFSSAAYRLDVQSGNGTYLQVVSSWIPWDKNTLPGYLLASIYQTYAAIYGGGWITSFDTNAIVIMVFFRAELELLRIDCAALFDDEKSFGDMAFMRRLKECHRRHTELVKHSRLFDSCLSPIMLLYMFVCSVMLCVTAYQITIETNPMERFLMTEYLVFGVAQLFMYCWHSNDVLYASQDLSRGPYESAWWSRDVKYRKNLYILVAQFNKVIVFSAGPFTKLTVATFIRILKGAYSYYTLLSQSQMNKT

>BmorOR57

MPSLIKNRIFGLTLTLNTLSWAGLILRDDYTKTQRIIMKVYGGLVFLYLFVFTAYVQIADLVVIWGNIDFMTETSLILFMQLAVSAKVLTLMLKSKKIMEVTNEADAILISEKKVEGQRIIASIDKNTTLFLKYYGFFVAFTIICWFMGENTSTFFIRSKYPFNELKSPGREFAFVHQCIVVIFTGSFDFNVDIIIISLVAVCRCRLKLVALSLRNLCLDIPMNKRNLITSDEEKVITERLRNIISQHKRALDAAEAIKHYLSGALLVQLMVSIVVICTTAYQLAVKKSTTMQSLTMAGYLFGTSLEVFLFCYQGEFLRESSEEIADAAYECPWYTLTRPLKKTLLIIMTRAQRPATLTAGGFVTLDITEYMAVSLISNT

>BmorOR58

MKLVFDNFIFALKVTLNWCRYFGIFIPDELTGRRQKLLVQAYSVFMFMLFIGFFIITQIILFILVWGDLSLMTDVGLVLGTNLALSAKIAVFFFKREELASILKKNDDTLRFETREEGKKIISEYPCDTKRSPAYEIIMIHQTIAVAVIASLAITADLLMLSMIAVCRCRVKLVGLYLQTICDDLPCNVKNKLTSDEEVIVAKRIREYVIEHQAVLDCISELQNHFSPALLVQLLTSVVIICVTAYQLAVEKSSDMLRKFTMASFLFGMSTEMFMFGYQGGHLSHDSMEVATAAYSCPWYTFPTSLKRSLLVIMIRAQQPALLTAGGFTTLSLETFVTVS

>BmorOR59

MDTNPSAAGDSVAPHLRRLRQVGFCQLDPTSQSRRPILALMHRVYHRLVLAATVLYIFEQLTYAYQARNDMERLSRVLFLMLCHLTCIAKQFVFHSDADKINQLVVGLDDALCNQPVETHRLLLLETSRRAARLLMLYSGCAVSTCILWAVFPLLDQLRGRTVEFAFWIPIDYRHNAFQFAVVLAYAFYSTSLVAVANTTMDAFIATVLYQCTTQLRILRMNFESLPERAYALSRKTRQDYHTVTHELLVDCLLHYKKITETCNLLEQIFGKAILVQFGVGGWILCMAAYQIVDMEILSIEFASTALFMGCILTELFLYCYYGNEVTVQSGLVSESVYAMSWLSLCPRERRALVVVLERARRPLRPAAGRVVPLTLNTYLKILKSSYSFYAVLRQTK

>BmorOR60

MVRPCRYFAIHFILLRFLGLGWWHHPHENETRNYPGLYLYYSILTQLVWVVGLVGLETIDPFVGEKDMDRFMFSLSFVITHDLTLIKLYIFYFRNVEIQDIVRTIEIDLYRYYQNDDKIRATIRISRIFTAAFLFFGWVTIGNANIYGIVQDLRWKDIVKNLNETTSKPLRTLPQPIFIPWPYQEDKHYILTFILETMGLLWTGHIVMTIDTFIASVILHMSTQFAILREAIVTAYDRTMIALSEGALQSGVLCENSNGNEENNQIFLESFYSKEHIESVLESTLLSCIRQHQLLIGCVEKFSKTYSYGFMTQLLSSMAGICVVMVQVSQGASSFKSVRLVTSLAFFFAMVIQLAIQCFTGNELTIQAERIADAVMESKWEKMPVRLRRLLLVTMMRAQRPLHLTAAGFAYIDNTCFLSILKAAYSYYAVLSQKQG

>BmorOR61

MARITDVFRLNFIFWKFLGIWGKSAPSKYNMAYTALYLSASLFVYDIFLTLNLIHTPRKLETLLRETMFYFNHLVAMTKILKMFIRRKKILVIFDLLDCEEFKPSDEDSQEIMKRKNEFYYIYWRIVAVTSNLSCFMQVVGPLIKMLIWKSELGLPVCKYYFMSDEFRNKYFVIWYIYQSFGIYNQMVNNLNLDTFNCGMLWMAVGQLQILKTKFVNFKLNDIENSLDLKTRDDMQTERLRKYLTHYEIILKYCATVQDILNITIFVQLGMSSIVICVGLCGFVAMPSNTETAIFMSSYLITMTMQIFVPSWMGTQISFECGELMSAAYCCEWIPRSKLFKRSLILFVERAKTPVRITGLKIFTLSLDTFTSIMKTTYSFFTLIRQLQVDEVN

>BmorOR62

QNVILEYAKKGRWLCRAWAILTTCGMAQFFLKSIIVCTIYSAIQGNFRIVQYYEVIYPEVIERHRNNPVIFITMYFCTFFYSLYTSALYTSVLPLGPIFLLHGCAKLEIVRLNIKNLFDNDDYVVQERLKKTVLQMQEIYCYSNEINECFQVIYEFLLKSSSLVLPITIFAVIQVSSLHICFIFLIPNTQWNVCSQNKHFLGSLEMSCRNCHSK

>BmorOR63

MKLWIRNANFTISLSLTLLRCLGFWSPDGLAGNKRLLYNCYSFVFFMFLLGIYILIQVVDMIKIWGDLPLMTGTAFLLFTNFAHATKVINIVIRKNRIQRVIQQANAVLMGVQSEEARRIVKSCDFETSIQLCLYFLLTFVTTVGWATSAEKHQLPLRAWYPYDTSKSPAYELTYIHQVAALLIAAYINVAKDSLVSSLIAQCRCRLRLVGLALASLGQDLKIDYQSQLSPAQENILNLRLKTCVLEHQTVLAAVTELQACFSKPTFAQFTVSLIIICVTAFQLVSQTGNLVRLLSMGTYLMNMIFQVFIYCYQGNKLSVESSEIAGSVYFSPWYLGSVKLRRALLIVMVRSRRVAKLTAGGFTTLSLASFMAIIKASYSLFTLLQQVKQKK

>BmorOR64

MGVSNGRGTVKPFLYPLVDELDYNLIVGVHLPFEYKTPSRYPLAYITVVIAFIYVSYFVMVTDLIMQAHLLHLLCQFNVLADCFENMLNDCVKGFEGPLVSLHEYIHPLIDEFEYNLMVGLRLPFSFDTPLRYLFTYVIVLIAFNYTAHYVMVTDLIMQSYLIPLICQYAVLADCFENILIDCSNDYGDHARRNDIVYSRSMELRAILSRPMLGQLASSGLLICFVGYQATTSISVNIVKCLMSLFYLGYNMFTLFVVCRWCEEITNKSLNIGNAVYCSGWESGMTVVPTVRSTILLVILRANKPIVFTAGGMYNLSLTSYTSLVKGSYSALTFLLRIQHE

>BmorOR65

MRLGFEVSISEYLYRNIFYIYTLFHILLHFYYILHMIKLDLEAIFDDIDESVALLPHRDTRRIEVQKILNGRMKRVVTWHISVFKAVEAVSSIYGPPLAYQVMFTSIAICLIAIQITQKLENGILDIRFTMLGVAACLQMWIPCYLGTLLRNKAFGVGEACWNSGWHQTPLGRMIRQDIIIVLLRAQQPVTIKFPGLQSIQLETFSSVIFNLYGYYYFLLLRWVDELTAHLVLSGYWSP

>BmorOR66

MRFGLKVYIYTLFHILLHFYYILHMIKFDLEAIFDDIDESVALLPHRDTRRIEVQKILNGRMKRIVTWHISVFKAVEAVSSIYGPPLAYQVMFTSIAICLIAIQITQKLENGILDIRFTMLGVAACLQMWIPCYLGTLLRNKAFGVGEACWNSGWHQTPLGRMIRQDIIIVLLRAQQPVTIKFPGLQSIQLETFSSVIFNLYGYYYYYCLDG

>BmorOR67

MRFGLKGGAAVVTILETLELISQGGFIETIQVTFGGQLSSMLFISACIICSTAVQILAIESPLDNLTTVGWILVYLSLCILILFVDCYFGNTITVKCAYLPTAVFSIPWLDQPKNIQVSTLLFMAKTQQPVQLIAAKLVPVSLTTFTQVSYCPPLDLKCLQGGVIAHLAKD

>BmorOR68

MFTIDFHDERITSFNKNQTRKIIICVITGGRTSCESARVGTTTLPISASEDVCMTLYSCGWETRFDLNTRKCIILMLCRALRPVSIRTIFRSVSLTTLTGVFQQAYALFNLLNAVWN

>HarmOrco

MMTKVKAQGLVSDLMPNIKLMQMAGHFLFNYHSENAGMSNLLRKIYASTHAILIFIHYACMGINMAKYSDEVNELTANTITVLFFAHTIIKLAFFALNSKSFYRTLAVWNQSNSHPLFTESDARYHQIALTKMRRLLYFICGMTVLSVISWVTLTFFGESVRMVTNKETNETLTEVVPRLPLKAWYPFNAMSGTMYIVAFAFQVYWLLFSMAIANLMDVMFCSWLIFACEQLQHLKAIMKPLMELSASLDTYRPNTAELFRASSTEKSEKIPDTVDMDIRGIYSTQQDFGMTLRGAGGRLQNFGQQNPNPNGLTPKQEMLARSAIKYWVERHKHVVRLVASIGDTYGTALLFHMLVSTITLTLLAYQATKINGINVYAFSTIGYLSYTLGQVFHFCIFGNRLIEESSSVMEAAYSCQWYDGSEEAKTFVQIVCQQCQKAMSISGAKFFTVSLDLFASVLGAVVTYFMVLIQLK

>HarmOR9

MLDQFDRCLKSVNLYLKFLGLYLESKDTDKTFVERTRSHRLYFAHLFSLNLEVVAQVLWVLEAVITGKSFVEITRLIPCLILCLISNFKTLSLLYYGRHNNEFIVTMRSLLLNQMQVEEKEHRFRKNLIDKHVLILTSISKKISYVIVLDLLMFALAHAFIIIPHYFKTDEVKLEMPFIAYYPFNEFDLRVYPWVYFHQVYSAVIAMIMVYGPDCFFFTCCTFIHIQFSLLNNDMERIVTEETPRYDKTKFKKLAVRHIELMRCVNLLEKIFSKSILFNALTSSVIICVTGFNVLVVDNIVMMASFTAFLIFGLMQIFLYCYYGDTIMRSSMEVSTSIYNSLWYNIPAADRKGFLIVIIRAQKPCALTADGFFKMNLSAFASILSKSWSYFALLKTMYHPE

>HarmOR12

MEDEPLLIDKTVKNIEFLFRCTGINIKSGTKTRKDMIKSRTVYIINFLWLNIDLAGAVMWFFTGIANSKSFTELTYVAPCITLSFLGNLKSLFLILREKHVDKLIQVLRDLEINEKARPKSEETDAIIKYEHNFVTTVISVLNVLYFVLLVAFALSPVSLVALKYFTTNELELLLPFLIVYPFDPYDIRYWPWVYLRQIWSEVVVIIDICTADYLFYTFCTYIRMQFRLLKHYIERVIPEDDGGGRLTNIEQVRAEFVLLIKWHQDLISSANMLETVYTRSTLFNFVSSSVLICLTGFNVMAISDVAFVATFLSFLFMSLLQIFFLCFFGDLLMTSSTEISEAVYNCRWYLADTSLGKDLLLVQTRAQTPCKLTASDFSEVNLKAFMKILSTAWSYFALLQTLYGAPT

>HarmOR3

MIDDGFFSFNLKYLFFVGLWPEKTLTRNQKILYKMYEHFISFLTTTFIVLAGIGTYQHKDDLVVVFCNIDKCLVVYNFFFKTIIFFIKRNKLRDLIDEIEMSGDEVTEERKKLMANYVMFITGVTAAVIGAFSLLALFEGTMSIEAWLPFDPMGSLMNQILSLEILAFCVFPGLCRAFAMQGLVCSMIMYLCDQLIHLQKELRDLTYVKETEMVMRTKFKNAIRKHIRLMGYSGRMENIFKEYFLVQNLAVTVELCLNAVMVTVMRVLHIALHITSLAYLTLALQKTHLFLYLAAELIIQSQGIALAAYESTWTSWPVDLQKDLLIVILAAQRPLKLSAGGMALLCIQTFSQALYNGYSIFAVLNDAVN

>HarmOR10

MAVKNTSLFLGRPKKILSAHGVWPHPNNFVILRKLYMLFVMWTQYSFLLFEIIYIADVWGDIDAVSEASYLLFTQASLCYKSTAFMVNKQSLLELLEIMDCEIFEPKSAEHEKILAAQARKIKRLCLFFLTSATTTCTLWAMIPLFDAASKRSFPFRIWMPVTPLKSPDYELGYLYQMVSIYISAFLFISVDSVAVSMIMFGCAQLEIIMDKIQKIKYVFESADSEEGRRNIIKTNNEFLVECVKQHQTVERFIQLCEDTYHANIFFQLTGTVAIICNIGLRISIVEPNSVQFFSMLNYMVTMLSQLFLYCWCGHELTIRSENLREWLYQCPWYEQDTEFKRALFIAMERMKKPIIFKAGHYISLSRPTFVAILRCSYSYFAVLNRVNTE

>HarmOR18

MEMKVDVLPEKKYKGFNETFKLCAFSLAFAFLYPNRTTALRRCITITLIVTFCGGQLFWFITYTFKCLYTLDIYNFARNMTLAVVLVLFFIKTYYVIYATSKFAPLLDKISDDLLEANNLEEEFQVLYDDHIKIAKVGEISWLLIPTIMSALFPIYAGALMTIESIQTDDYERRMVHDMELLFVEDIRSETPFFQCMFAYNCVQCVVLVPNYCGFDGSFCIATTHLRLKLKLMTLKVNKAFKYSKSRQELRMRLYDSIKDHQDALDFYVQLQNVYGPWLFAVFLLTSFMISFNLYQIYLLQRIDPKYTSFGVVGVLHIYLPCRYASDLTRVSEEIPDDLYLAQWEAWADPSITKLLMFMITRAQQEMIVTGMGLVVFNMEMFKSILQTSYSFFTLITA

>HarmOR20

MDEELEFKPFHETYRLITFSLCIAMIYPNPRTEKWRLFSIPILIATVAPVAIMIFLDMYKCWKNGDIVNIIRHSTVVGPFLGGFFKMILMYHKRVQAKQILDEFDRDHLMFNTVAETYKDIARASIRNCQIYSERLWACLVTTCVMTFPVMAIVLNIYNFMFKSEPTKYMIHDLEKPFSKEPEERFESPYFELLFVYMFYAAILYVVNFTGYDGFFGLCVNHARLKMELYCKALEEAMMADREEVYGRVIAVIREQCRMFRYVDLIQDTFNIWLGIIFIATMIQICTCLYHITEGYGFDIRYMIFVYGAVVHIYLPCRYAAKLKAMSMETSNRFYCSGWERVDDERVRKMIVFMIARAQVPNEITAFNMMAFDMELFLSILQTSYSMFTLLRS

>HarmOR14a

MGGIRDFIFNLEAKEGITKPTDYPYMILCRHLLTVITCWPKEPKEGLDTRAKLKARIWVTFQKIFHLNGCFITTIGMAMYIALHKNSMSFFELGHLYISLLMTVVIFSRVTTLCWNPEYQAVATDFLTKIHLFYYKDDSDFSMQTHKQVHKISHLFTLLLTGQMVAGMSLFNLTPMHNNFSTGKYKKGGLKNSTFEHSLYFSYPFNASSDVRGYILSNIFHWIISYLCSTWFCTLDLFLSIVVFHVWGHFKILIHDLNHFPRSLNTISFRLDQSNITLTTEMYSSRELVQVSERLNKSVEYHRRIVSFTDKMSEVFGPMLFVYYGFHQTSGCLLLLECSQMTVEALVRYLPLTIILFQQLIQLSIIFELVGSVSDKLKDAVYGLPWEDMDTKNRKTVAFFLMNVQEPVHVKALGLADVGVTSMTAILKTSMSYFTFLRSM

>HarmOR14b

MAGLRDFFFNYEANEAITTPKNYPYLIIMRISLSLIKCWPKKTTENLAAGAKMKAKVWGMVQNVLHLAFCVLTIVGTATYVMIHKKNMTFFELGHLYITLMLSCVVFSRLATLTFNEEYQVVANEFLNKIHLFYYKDNSEFSMQTHKQIHRVSHLFTLYVTGQMLGGLSLFNLTPMYNNYSAGKYSKGGLKNSTFDHSLYYSYPFDVSTGVRGYIFSNILHWFFSYIVSTWICTLDLFLSVIVFHIWGHFKILLHDIDNFPKPSKMVSFKLKNTNVTISNENYSTEELEQLADKLKKCIDYHREIISFTNKISEVFGPMLLAYYGFHQASGCLLLLECSQMTPEALARYLPLTLILFQQLIQLSIVFELVGTVSSKLNDAVYGLPWEDMDVKNRKTVAFFLLNVQEPVHVKALGPADVGVTSMTKILKTSMSFFTFLRSM

>HarmOR6

MSFRKFLFENEAVDGIKSPSDYLYIKILRFTLDVIRSWPRKELGEPESASFTVFMKYFYLVLTIATVVGSILYVVVHVSELSFLEAGLMYLIILMSFLDALTVMSLTFSAKYRVLAKDFLTKIHLFYYKDRSKAAMEIHKKVHLISHLFSLWLLFQMLSGLSLFNLTPMYSNLAAGKYRRGGLGNTTFEHSLYYLYPFNTSTDVFGYIVACILHWIISYLCSTWFCMFNLFISLMVFNLWGHFKILIITLEEFPRPKSIGTSESAYKYSQEELVEVAERLKDCINYHREIKNFTNRMSDVFGPMLFVYYSFHQASGCLLLLECSQMTAQALMRYLPLTIILTQQLIQLSVVFELVGSESEKLKDAVYSVPWECMDTKNRKMVRFFLMNVQEPIHVKAMGLANVGVTTMAAILKTSMSYFTFLRSM

>HarmOR11

MHLAGNAVTGITGPMDYKYMKVLRFVLRIISGWPGKALGEKTLRIEGMGHAYYNTILSLVYLALGIAYLKKNFHRFDFLELGQLYIVLLMNMLSTSRAFTLCLSQKYREVAKIFIQKIHLFYFKEKSDFAMKIHITVHKISFISAVYLSVLLFIAACMFNLIPMYNNYSAGRFASFDNLENTTYEQAISCLYPWNFETNFNGYLAATLSGWYGTILCGSSVSMFDLFLCLMIFNLWGHFKILIYNLEHFPRPASEVVDAEGEERSGRTVGSEMYSQSELEEVAVLLRDCIQYHMLIYNFTNNMSDAFGMALFIYYSFHQITGCLLLLECSQMTAAALTRYLPLTIIMFGELVLLSIIFETIGTMSEKLKDAVYKVPWEYMDTKNRRTVLIFLIKVQEPIHVKAGGLVDVGVTTMASILKTSFSYFAFLRTF

>HarmOR13

MKILSDGSDLEGVEKVEDIFYINLARKSMWILDSWPRTPNESVTYRYFVLALNVATLVGGAVYLRNNTGVLSSFELGHTYITVFMNCITCSRCIMILSREYNEVMLSFVNKIHLFHHRHKSEYAYKTHIFIHKISHFYTVYLLGLALNGLLLFNMIPFYNCYSRGMFRDVIPANATYDHSVFYSVPFDYTTKFKGYIAMTSFNCFISYTCTSYFCVVDLTVSLVIFHLWGHMRPLTYHLANFKKPASVLESNENTDAIKDHSYTQEELKEVFGKLREYIRHHNLILKFSSEMSNAFGPALLAYMVFHQVSGCILLLECSQLDMKTLVRYGPLTVVILQQLIQISVIFELLGSSNDKLIDAVYLVPWEYMDTKNRKLVFVMLRQSQRSIDLKMMSMLTVGVQTMTAILKTSFSYFVMLKTVAEEE

>HarmOR17

MFLRSECARSVAPHVRVLRCVGFLRGAALSSRGRAERLALRSYHALALAATSLYVLQQAVYAYQERGDMDKPSQVMFLMLCHVTCVVKQIAFHVDADRIDRLIASLDEPLLNQCAGERGALLRGTARGAARLLRTYAGCAVATCVLWIVFPVINRIQGISFEFPFWTGFSYDHNAVFTLVLLQSFYCTNLVAIGNTSMDAFMATILDQCKTQLRILRINFESLPERARALHVESGENYDTILDKLFVDCLVHYNKITEMCTELHDVFAVPLLVQFGVGGWIPCMAAYKIVSLDVLSIEFASITLFITCILIELFIFCYYGNEVTVESERVSQSLYSMEWRRARLTFRRSLVLVMERAKRPLRPAAGRVIPLSLDTFVKILKSSYSFYAVLRQTK

>HarmOR38

MIILSENIKQKLAFLSPYLPYGVIESWEDLNPRLYHAVHIYWLKFYGMWFNNYSPNNIKFWLHMVYTLTVLWLACFFPGIGEVVYLLKQRENIGDIADGLYLFLSEMYTYVKIAVFWMNRDKVISLLEYLHCKEFKPKEPEHRDIITKSIKSARFVMTYYSTMCVGAVSVGIIMPLTENFDILPTNVEYPFFNVYRSPAYEAVYIHHIYYKPATCIIDGVMDTILAAFVASAIGQIEILAFNLRNFNLVAERQRRRDLAQNKYIEEYPAQHYVRSVLKECIRHHNCIIRYVSMIESAFSLASALQFMLSVMVLCLIGIQFLSIENPSAHPMQIAWMGIYLTCMLIEVFILCWFGDELIWKSMDLAKAAFEGPWMNSDRQTNMFIIILLERCKRPLRLSAGKIFTLSLDTYTVLINWSYKAFAVMRNMKK

>HarmOR29

MGYQQIDCFDIHLKILRILGVWPHDNPSIYYIYFSRIFVFTFTVLYVVIYTMNFYFLPQQLEVFADELIFYFTNVGALSKALAFIFLRDKVKKMLFMLESEIFQSDDPEEIKLIKEGKEKSNFYWKITAGLSVSANTVNVCLPLLVHIIFSVELEFPVCRYSFIPEKYEAMFAYPAYFYQSIGITTHMLYNVNIDTFLLGVMFLAMTQLDILDRKLRKVTDVCINPDAARGSVDKFIDDQNAVLEIIKCIKHYDAICEYCKLIQDAFSEILFVLFSSGSCKICMCLFRFTMPATTGYFVFLYTYVTVMTLQVMVPCWFGSRLMDKSSQITIAAYDCDWTPRCRRFKSNLRLLVERANRPITIIGGKMFLLSLGTFTAIMNSSYSFFTLMRHMQSR

>HarmOR50

METTTYTRSKTTEFFYKMNFAIYIFGLPNFWIEDLKLSKRFVKIYDKISLFNDLLVYLLLVMEFGAFFTQHNLTDKQKFNLMVFAISHPLLCSFCVMVSKLKKKVRLVMYSQAVALKRDYNDPEVEKQMIARSLTYVLAFMSSCTITMIMFAIEAIWDVIRHGATFTTLITAYPDVQDRSILADVVRVLAFVTWWIFLTKMVAVYMLVIPLTISLRYQFKNLQSYFLSLAELFERSDLSQKEKEEKYEAGLKLGIKLHSETLSCAEDTQDVCRGVFSGQIIFNILLLIVLMAQMVTSERTFVNMFGTVATSCTVITSTGFFMWNAGDVTVEASYLPTAIYFSGWQHCQRDSSMRVRRLVVTCMSHAQQPVIFKGLGYIELSYQSFITIVKSSYSVFSVLY

>HarmOR27

MLSKIKNIIWCLGRQKIRNGEIDSVVTLLDRLILYNSGLASYTTTYKVHWTAHVLLTCFIIACVLQIIALFMGKDDPDRLFECFSVLSFCAMGMLKLLSLRKNHRKWRKLLTQITILENTQLSNRSISYVEYQSDSEDSDNFSEHISIYTKKFRGTSIVLTRIYSFTAFLFILSPFAERIICEIRGVECVGYPHVFPGWTPLDDFSIFGYLITVLCEVFSAVYCVCVHMAFDLTVIGIMIFVCGQFSLLRDYSSRIGGKGGQCNLSMRRDERARFRIIRCHDINLLLVNSITELDMLLKNIIGVYFFVATLTLCSVAVRLKSEDMGVMQLVSLIQYMCGTLTQLFLFCRYGDAVLHESTMGMGEGPFAAASWCLSPRVRRDLSMLSAGMMSQRHLRAGPFSFIDLPSFIQVVRAAYSYYAVLGKKE

>HarmOR4

GAHAFILRISSFFGLAPLRFESRSNGFTVSISGAMCVYSYILVTVLVICTIFGLVAEINVGVELSVRMSSRMSQVVSTCDVLVVVATAGAGVYGAPRRMRNMLKFMENIASVDTSIGGQYSLVTERKLCGIILAILIFFSILIADDFTFYALQAKKLDREWDVVTNYLGFYLLWFVVLILELQFAFTALSVRARFSAVNDALALTARQVSIPAEKPKSSSPLNIYAIRVAPVDSQRSANVSLLVDTMTGREHVVIIKRTASGEPRLVVSPCDAVRRLAALHGTLCDVVNSIDDSYGLPLVVILISTLLHLIVTPYFLIMEIIVSTNRIHFLVLQFLWCVTHMLRMIVVVEPGHYTIAEGKRTEGLVCRLMTSAPSTGVLPSRLEIFSRQLMLQSVSYAPMGMCTLHRPLIASVIGAVTTYLVILIQFQRYDN

>HarmOR16

MGLRQFLFENEAVEGINSASDYLYIKVLRFMLLIVNSWPRKEIGEPESPKFSAFVKYFYLVVTVSGSAGFILYLVKHNSELTFLETGHMYIVLLMSLIDVSRVATLTMSTTYREVARDFLTKIHLFYYKDRSKQAMETHRAVHKIAHLFTLWLVSQMLSGLSLFNIIPMYSNYAAGRFSGEVSKNSTFEHSMYYPYPFNTSTDIRGYSIACIIHWIISYLCSTWFCMFDLFLSLMVFHLWGHFKILNYTLNDFPRPSSEVEAAKYSDEELVEVAARLKDCILYHREIILFTDRMSNVFGPMLFLYYMFHQASGCLLLLECSQMTAQALIRYVPLTIILTQQLIQLSVIFELVGSESDKLKHAVYGVPWECMDVKNRRSVVIFLANTQEPVHVKAMGVANVGVTSMAAILKTSMSYFTFLRSL

>EoblOrco

MMAKSKSVGLVSDMMPNIRLMQWAGHFLFNYYDENSGMNMLLRKVYACVHAFLISLHFIFMCINMTQYSGEVNEFTANTITVLFFAHTLIKLVVFAFNSKNFYRTLAVWNQSNSHPLFTESDARYHQQALTKMRKLLYMICAVTGGAVISWVTITFFGESVRMITNKETNETLTEPAPRLPLKAWYPFDAMSGSMYIFAFVFQTYWLIFSLGIANLLDLMFCSWLIFACEQLKHLKAIMKPLMELSASLDTYRPNTAELFRISSSLNSEKMPDTTDADIRGIYATQQDFGMTLRGAGGRLQNFVANPNNPNGLSQKQEMLARSSIKYWVERHKHVVRLVASIGDTYGTALLFHMLVSTITLTLLAYQATKINGMNVYAFSTIGYLSYTLGQVFHFCIFGNKLIEESSSIMEAAYSCQWYDGSEEAKTFVQIVCQQCQKAMSISGAKFFTVSLDLFASVLGAVVTYFMVLVQLK

>EoblOR29

MENQAPPERTVPVQYARSRAFRQFKNPPQPHMCIQDSIKDTAEKLFINVLGWQKIANPKQYSDPIPLYGGMQVPQGCGPNSTRPAMLVFAVMVNPDILKANGKNAASATDREALVSLLCDFVEAMNPGLVLTRNPLILKDRDLAGELKDVWLAVQAKREREKGMSQDVMYKVYDIDGIGGDEQEDDKTYARNKHNEDSPSKGDKKPVKSSKQILLNAGQKSEFDSGMNNCQLNQNPNRDKTGNTNADTTYCTPVYGQIVSSRENHNQINEIQQKFASNETKPSGSFQKNRRFSPVKSEERVVQWDEFSKRDMAAVKNEEMKKQRCFKTEKTKQQMNGKNHYDFFPYFDNKSVESRETRDVPTEDTSKIIIDPMQKLVLHSTDNNICDNKASLSSIGS

>EoblOR4

GVPIRTEVTYYPSKSDSGLPVNILRFFIQFHWWFAVSIMVGIDSLCISSLIIVAYKFKVLQVHFISLRDKVLEDTKTKTRSEVEKVFREDFILGIKMHDDALWCAKNVQSALGNLYSVQIFEGVVLLIMCL

>EoblOR3

MSSTTLEQAKNEIDMTLNLSIFCMRRIGLSFDAPKSTTAYLKQQLMLVLSVCGICYHVFSEIVYIGLTLSNSPRVEDVVPLFHTFGYGALSIAKVSVLWYKKHVFIKLLNELAEIWPLDPLNQEDKTIKDNSLGALRMAHRWYFTVNVLGVWFYNLTPIAIYLYELWKGEDPTIGFVWVSWYPFDKHQPIAHVAVYIFEVFAGQTCVWIMVCTDLLFSGMASHIGLLLRLLQHRLECVGTIDQTDEEHYQEIVDNIKLHQRLIKYCNDLENAFTLSNFVNVVLSSVNICCVVFVIVLLEPFMAVSNKLFLGSALIQIGMLCWYADDIYYANLDVSISAYKGNWYVTNPRGRRALLFLIKRAQKPIAFTAMNFTDITLVTYSSILYRSYSYFALLYTMYSES

>EoblOR2

MSLICSQLKVSFKKIKNIFTESSFESLIAIVNFVPNLAGFSIRRKKIAAPFWILHLSLLLYVYGVGCLVYQIKVADGAEDFIKNFVNVSLLILIFNISWWWLNKRPLLLETLQLIENSDVEARTTEENLREKHNKMLHRIKIIVLIFYMTNCTNAACIYLPNRINVSNEYSMALCVGLEPITVTPNGQICSALLCIQELTIMIAVLNYQTLMLFIISHTATMYHMLAEELMSFNVYTNLAENQALVKERLPIFIKRHSMILSTCDNLKSLYSLSLGVDFGSNAICICLFFYLSLQEMVKYMPILIYCVLAFFLYCFLCQKLMTASEVFERAVYSCGWENFALNEKKMIYVMLRQAQKPVIILAADIVPVNMYTFATTLQTLFKFVTVVKL

>EoblOR1

MDDQLECYVSKSLAAMLQVSRLAGLAPLEFEKKGDKWRIRVSRSWSYYGYVIITAIIITVFAALLMDLQLDPRESVRMPSSTRRVVWITDVGCMLLLSATSVYSAPGLMRAMIATVNHIEKINFDLNIRQYPMAFNKKQIWFFFVWTAVTIILTTVDHVVLLLMEDTKKTPTQSTISDMYMIYYCCYWILQLRQLNFVITTLQVMYCLRRINDCLKNLLRELADRDEHSFISLFNTKLSKATKKKQLKFKVDISVTTDFKKFQPEGTHKTAYYQDAIRRLGLAFGNTCDVVRHVDNAHGLVVLMMLGSFLLHLVCTPYYLITGMFEFSSEENWTAHLVYNAVQFVWCFYHTVNLLMVIEPCHLTQIEIETTCELVSHLMRCTDSVHDPLAMELEIFFRHLFLNQASYSAMHVTSISRSLVATILGSVTTYLVIIIQL

>CsupOR1

MRFRIFQRKLSLEENKSTLQSEPITNYLHFLEIPLKIVGCWDWYMKPETECQIILNNIYYGMVLFFLINVPATLCVHLSTEWKDVMTTLDEIADCLPYFVSIVIVIYFGVYRQEMYDLIQSMGEQFKYRSANGLTNMTMLNSYITAKKFALFYTICTLFSVSMYVVPELISWWTNKPLQSFMYMDITKSPFFEITFLTLYLSQAFVGLAMGQFGVFFAANSILLCGQLDLLCCSLRNTRYTALLQSGVHYRSLRLSHSDIKSDELHNYIYNVAEMEESSYHYDDKMEAITRPRKTKFDIYSSEYDEATERAVRDCARMCRVVNSYRERFERFVSPLLAMRVVQVTMYLCMLLYSATLKFDMVTVEYLGAVALDIFVYCFYGNQIIIQADRVTTAAYQSAWATMGVRGRRLLLNVLLANRRAVAVRAGYFLPLNLHTFLVIIKTSFSYYTLLVNVNEK

>CsupOR2

MASSTRPRHYFYLHFLLFRVLGLGWWHQPDERDTRNFFGWYLYYSIVTQFVWVVGFVGLETIDPFIGEKDIDRFMFSLSFVITHDLTLIKLYIFYFKNDQIQDIVRTLEIDLYNFYQNNAKNRATIKISRLMTASFVFFGWITIGNTNVYGTIYDIRWKAEVAKLNNTDLAPPRTLPQPIYIPWNYQTEEAYISTFVLETVGLLWTGHIVMGIDTFIGSLILHMSNQFSILREALITAYDRTMIRLYEGVRQDFIAITNSDIDKKEQYTQDNIEEVVKSRYSKEEIEVALTETFKNCFRQHQALIGCVENFSTTYAYGFMTQLLSSMAAICVVMVQVSQDASSLKSTNLVTSLAFFVAMIIQLALQCFTGNELTLQASRIADAVMHSKWEKMSPKLRRLLMITMMRAQRPLRLTAAGFAYINTDCFISIMKAAYSYYAVLSQKQST

>CsupOR3

MCFNIASVRHTTDNSTQISFEKKSLGALFTIVNLKVKDIRKMARSQRLSIIASIRHILSTAGIKFTDTMHVHWMAKVAMICLIFTYVLQASALIQIRHNWEFFFECSGDLFYRGMSLVKFYIFRRNYETWCSLIEQADKIEEDELSNERDRENGNFLFSEHIQAYSVRYEKIKKIITTIFRTCTVMYVSSAFIEYGIKKQTVDGSVDLPHILEIWSPLDVSIVGYIITVSFELISAVYDTSTQIAFDLTSIGAMIFISGQFSLIRHYSEAIGCKEQIYPSKEQDDLAHKQIIICHQIHIQIKHLTEMLKGLLTNILWLYFIMSTVMLCSIVVRLNLETSLVQLMTMFLYMCGITTQLFLFCYFCDDIQNKSAIGMGEGPYGAAYWSLSPRIRKELMILARGMSIPCQLYAGPFIPVTLPSFVQILRTAYSYYAILGNRG

>CsupOR4

MKTVLTPSQFAIFVQFVMLERVRKFGLRYCDLPTMLSNVSCMLRVLTLNVDSRHQKGIPIIFYVLTATAAASYFYIYLVSMVWFVFWRCSDTGDTIPAMIVFSLGIASEIGPCKLIFMFLYRDVIMKIVDGYLSCDATVIKGKRFSENLLKTLRVVKKRALIYWLVIIGNGITYVMKPIILPGRHIMEDQFILLGLEPMFETPNYEIAFTLNTMGVYFTCYLPANITAFFIVIIGYTEGTMLALSKEIRHLWDDAQQFYQETFNNAEVAINGGTIDPTFKKRVINQFIKERLQKIVVIHTTILNLIHQVEYVFRITIAVEFVLLSAGLIVELLGGLENTYIQMPFTLIQVAMDCFIGQRLMDASVVFEEAVYDCKWENFDTKNMKTVLLILQNSQKTMALSAGGVTTLSFSCLMTVIRSIYSAYTALRSTMT

>CsupOR5

MKILKRNIKEKLAILKPILPYGVLESWDDLDPKLYHAVHIYWLKFYGLWYYDFAPGSFMFWIRFLYTMLVMWLVCFLPGIGEIVYLLKRRDDIGDIAEGLYLFLSEMYTYFKMSVFWLNKDKVLNLLQYLTCEHFKPIEAEHREIIKKSIGTARFLMTFYSTICVGAVSVGIIMPLTENFDILPTNVEYPFFDVYQSPAYGILYFHHAYYKPATCIIDGVMDTILAAFIVSALGQIEILTFNLRNFDVIAKRRHKRALDGNKPEASWSNERHIRAVLKDCIIHHKSIIRYVSMIESAFSLASALQFMLSVMVLCLVGLQFLSIENPASHAMQIIWMAIYLTCMLIEVFIICWFGDELIWKSRGLVQAAFDGPWLKIEQKDKIFIVIFLERCKRPLRVTAGKIFTLSLDTYTILINWAYKAFAVFSKVKK

>CsupOR6

MWKTIRKFGLEYCDLPTMIWNVSSMLRVLNLNIDPGNTKPIPTTYYIITAIVTASYFYVYLVSMVWFVFWRCRETGDLTAGMIVLSLGITSEIGTTKLMFMMIFRNKLREIVELYLECDSHVNPDSRFLHNMMKTLRHVKKRAMIFWLVIIGNGVVYIVKPAIQPGKHLMEDVFTLYGLEPSTEWPNYEITFVLMALGVVQTVYLPANITAFIIIIIGYSEAQMLALSEEVLNIWNDGLHHLNDHVIIDACADSNDQLTSLEEIISANRNNYDRINEFIKIRLREIIKVHMTNINLVQQMEQVLRGAIAVEFGLLIIGLIVELLGGLENTYMEVPFALMQVAMDCLTGQRLMDASIIFEKSVYACKWENFNVENMRTVLLMSLISQKTMKLSAGGVTMLSFSSLMMVIRSIYSAYTALRPTMS

>CsupOR7

MFWNKIKAVYNKEDFDYSKRFVDPKIYHRIFFAVQRAYQVIDEPFATWTYITKTITALCGVGVLTDACLSLYHAIDIFDMSLITESGTYVLMLLYKMMTLITTKVNLSDYIHLIHAMKEDFNYIETKKEKYRKVFFETQLGTWKACFVVISFMFSMGTSLVLFASGTLVVYHLTHTPGDGSHRTLVFPFWAPGVDYTTTPAFEIAFTFANIGVMACCYNYAFVIQTNIVWIRQIAAKADLIGMCINDLLEGIYSTDDEEQRQHFASLINFRMKEIVSQHIIMYRLLECYAAVYRKCLMFEQFIASPLVCMLAYCSAEKIDNGEVHVVMMVLCLSAILVLFIPCYLCTYLRTKLSGIHDACWNIRFWDAGPNIRPYLVLIMQRCLRPLPLQMPGFQEVSIKTFSSKMTSAYSLFNMLRQADLDF

>CsupOR8

MERMIKNLCDILISVSYREVTTMLRPFFKRLENNNHPLLGPTLWGLARWGMWQPRLGINTKIYCILHIVATLFVISQYVELWIIRYDFNLALRNLSVTMLSTVCVVKAGTFVIWHDQWQEIIEYVSKCENRQLSKRDKITSEIINNYTVYSRRVTYFYWALVAATVFTVTLAPLAAFWSSKEYRARIRAEQIPYPEIMSSWLPIDRTRGIGYWLSIVEHTLICFYGGGIVATYDSNAVALMSFLAGQLKLLNTNCSRLFEENYESRNNTVAKIREYHHDHLRLIKYSKILNGVLSPVMFLYVIICSLMICASAIQIATNGTTSMQRIWIAEYLMALIVQLFLYCWHSNEVLIMSHKVDDGVYASSWWSQSQSVRRSVLLLGGQLRRPIVFTAGPFTKLNLPTFLAILKGSYSFYTLLINKED

>CsupOR9

KRRRQWLSALIQFQLNQQKSTMLLDRLISFAKRWEDPESPLLGPNLKALHLFGLWKTDIRVRSTLLIIVVVFVITQIIDLYLSREDINKALCNFSLTTLSVICIAKSYSLIVHPVLWKKLVENISQEEATQIKKQNPETLSVIGNYTRYSRFISYTYWIMVAMTNFALIVAPLIRYLTVSKYREDIANGIERYPHIINSWFPFDDNAMPGYVYASAIQIIMSIVGSGSLAAYDTTAFAIMIFMKGQLIILKNNCKELFRWETKENNIEFFAKIKECHRHHDFLKRQFNQFNDLMSPTMCLYVLLCSITLCCSVVQLISKEATASQRLWIVQYSSGQILQLFLFCWHANELFLESQNIDGGVYASDWWKADVRMRKQILLLAGKVNYPMLLRAGPLSVLSLSTFFNIIKRSYSFFTLISRMQE

>CsupOR10

HPSAKSPIQRKSFDIKGVLLERVLVSSISMDLPTYDEIFKGIKNIFWLTGIPLDAPHKKLRFYVACLSLIITLYGEIAFFTSKISSENILELMDLAPCFCIGALSFFKGIFLAWKLNKIIVLKNSLEILYDTIFKSDSKRKLLHREIMKVHKLVKYYFGVNTALITVYNFSAPIFMTYHYLSQGKVKFMVPYAVIYPFAIDNWPAWIVAYTEQVFSGFVCILFITMSDALFCVLTSQICNNFYVISDEIKRLKNGNHIGLGEIVKQHQYILKLSEDLEDIFRLTNLFSYLVGSLEICALGFSITIGDWSHFLGYILFLVSVLLQILMMSVFGENIIRESGRVGEAAFLCEWHEINEKAKRTILIIMIRSHKHQKLTAYKFSVISYGSFTKIISTSWSYFTILKTVYKPSEVNNI

>CsupOR11

MNVISINPAEYRNTLILSLNYLKICGITLDRNDSFWEKYCHLCVVSIIMMLHFVSASLYIVQELVQNILQEANFISLWLITVQVFLRGIIILTNKTSIRGIIEQIGCNWRSSDLNEEQIRIKKDFLNRLLYTQKVIKIIGWCAGSLFLLPPLLETVFRSFVLHQDSAFVLPFPCYYQFTVTGWFTYFIAYFIQIYCSSKLIFMYLGADLLLIVLCAHLSNEFELLQVDLGATIKPTKNENEILEDEITAFGREERSIGDFVRRHQKVILLVQLLNISFNKMIFINLLFAAIAIVFFALGGRASRDPTNVANNYMAILVILINMFVLCYSSEMLCTSSSGIADYAYNNIWYEADMRYRTDIYFIIMRSQKACSLSSLNYLPISLSTFGKVLSTTWSYLSLANTFFEN

>CsupOR12

MKILTTQNENSEKEIRIKPFHETYKKITYGLTLGFMFPNPRTAKIRIVTIVIMLVLFQPIVVTVLIDMYSCWQKSDMFNIIRHSTILGPFLGAFYKMFLMHYKRAEVKRIIDEINDDYLTYNNYNHELKQIALESIKSSVFFVEQLWTYTVTACIMAFPVMGIVLTFISHLTQSEPKKYMVHDLKIPFRPPEDRFETPFFEIMFVYMFLAAIICVLNYVSYDGLFGLACYHACLKMRMFSKKLEYVFQCKDGDAYSRLVQVIEEQKATYEYNALVQNSFDIWLGTIVISTMIQLGSLLFHISAGYGFDFRYMLFSCTSVVHIFLPCTYASKLRNTSVETSTLMYCSGWERSRERRIVRVMPFLLARAQIASRITAFYLFDVDMQLFVTMMRTSYTIFTLLRT

>CsupOR13

MKKNMKEFEYDFEKAFRITTKALHLNRAHPFIERNLFWCFQFLLILTLSVMTFVFTFNSLLFYDIPAGEIAEASKNGTMAIVSLTITFKYTFLLYNQNYIKRYIAIINKDYELSKGFVAEERAIVIDYSRKGAKVSLYWLVATTATSILFPVKALVQMVYYHWEDEFRFVPMFDMRYPTTIEIMKNVPAMFCLLFLLCLMFDVYATTMYVGFDPLVPIFLTHICGQLDILSQRIMDIFSDESNLNSQEVNYKLKCINVTLQDQYNMIKEIKSKFTFLYEFTMKTTTILLPLSMFQIVEDLQRRKLNLEFISFFFATILHFYMPCYYSDMLMDRSQKLRDAVYACGWEKRHNARARKTILLMLTRTTVPLALSTVFYPICLDTFAEMCRQSYAIFNIMNAAEV

>CsupOR14

LHSCVLFRVTKMDILTSIGGVFRRIVTRFREDSFDSLLGIVDTVPSLAGFSLRKDKIFVPFFIFHLSLLTYIYGVGSFEYQAKTAKSAGDFIKSFVNVALLVLIANNSHFFMMKRSLLRSTLTEMQNSDKLARCNPASRLKHKKLCNRIKYIILIFYFVNLTNASCVYLPSRSNVDVNIYGVTPCYGMGSLTSPKREICKAMLFGQEVTVMIVVLNFQALLIFVIGHTSLLYQILSDEIMALNDYDKSMFFNNPVVKDILPVLIRRHAMILSIINKCKVLYSVPIGVNFGSNAVCMSLFFYLPLREWIDFFPILMYCFIVFFLYCFLCQRLTNAAQLFETSVYACGWENFETNEKKAVYFMLRQAQKPVELLAADIIPVNISTFATTLQAMFKFVTVVKV

>CsupOR15

MFYLLKKLEDKNRPLIGPNVKALKFWGILLPKNLYTRYLCILMYLLVVIFVGTEYVDIWFVKADLNLLLNNMKITMLATMSVVKVSTFYRWQQHWLDILNYVTRADLTQRKTNDVNKIEMINKFTTYSRKITYAYWSLVYTTVIFVVGYPIFKYVFFSSYRQNVLNGSEPFFEIVSSWVPFDKSTIWGYILASIYQAYSSIVGGGWITSFDSNAMVIMVFFRAELELLRIDCANIFGTEKAQVSDEVAMVRLKDCQRRHAEVMKYIHLFDECLSPVMLCYTIICSVMLCVTAYQITTEPSFVQRLVFTEYLVFCVTQLFIYCWHSNDVLYASRDLSLAPYESIWWSRGVEHRKNLFILTAQFSKVVEFSVGPFTKLTVATFIQILKGAYSYYTLLSKSDE

>CsupOR16

MKDIFILKTYCQYIYRVGSGNFWYEERIVGNDRSLSYKIYRGLHFFLYGCLTILEIMAAIFGVFPSDEKRDAVTFAVSHTIVMIKLFSVISNKALIKQLNKNMTELCEEHEEQQLMAEKYKIVKINVAVYFIIVYVTAVFFAFEGLRKLFNGVHFVTVVTYYPAYEDNSALANSFRIFTTIILQVMLMSMIVTVDTFTMTYLIIFKYKFITLRHYFDSLRENYLKMSKNGNQEIAAEQLTNGFVKGIIMHQKLLKTAKNIDTAFGLVIALQLCQSSGSAVSLMLQLALTDQLTFLASIKAILFVLALFFLLGMFLCNAGEITYQASLLADAIFYIGWHEFAPQPPPKRSLRRLVLLAIAQAQQPLIMKSFKMIELTYGTFLQVVRGTYSVFALFYAQ

>CsupOR17

MSEEAKREIAESLSLNTFCMERIGISFESPKSNIANVRQKLMFVLSVWGICYHVFSEIAYICLTLTKSPRVEDVVPLFHTFGYGALSITKLFVLWYKKNVFKQLIFELAGIWPLPPLDDDGQSTKNKSLAALRMTHRWYFAVNVLGVWFYNLTPIGIYFYRKWQGLDVEMGYVWVSWYPFDKHMPYAHFAVYIFEMFAGQVSVFIMVSTDLLFSSMASHISLLLRLLHRRLEALATTNKTEHEQFDEISANIKLHQRLIRYCNDLESAFSLSNLVNVVLSSINICCVVFVIVLLEPFLNVSNKLFLGSALIQIGMLCWYADDIFQANLKVSAAAYNSGWYHTSPRCRRAILFVIQRAQKPIAFTAMGFTNITLVTYSAILTRSYSYFALLYTMYNKG

>CsupOR18

MLKRFFNSLEDPDRPLLGPNYWILKKLGLLLHFGKLGNIFTILIHNMGLLFVSTQYVDLYLIRSDLDLVLQNLKISMLSVVCVLKVNTFLLWCSKWKEVINYVTEADKYERNTDNPDNVQIVKKYTKYCRRITYNYWILVFTTAFITVVTPLLQYAYSSTFRESVKNGTEAFPHIFSSWVPINKNDFPFNWVTVAWHTYICVQGALVVMAYDTNVMVTMVFIGGKLDLLRERCKLMVGVNGVTTSNVDLAEKLRELHKTHVLILRYSRLFNSLLSPVMFFYMVMCTLMLCASAYQLTSATDTTQKLVMAEYLTFGIAQLFIFCWLSDDVLTKSEKVMLGPYESQWWLANVKQRKIILLMAGQLRIVPVFTAGPFTKLTLSTFLNILKGSYSYYTLLR

>CsupOR19

MPPAERHFLGSIILQWMSNRGFLMNNNCYTYWLNGVTMICCHVTFVLQCVAVIDARDDPERLMQCFCDASFGGMCVLKEFSLRKHRHCWISLLSKISHLEEEELEFETKSINQDEDAHNIVFSGHIKEINKKVKKLNNILSRLFSVTAIGYMLTPFIEYGIRKLIGAETAGLPHITQYWSPLKTNLLGYILAITLEILFVINNYAVHTTFDFSVFGIMIFISGQFRLLHDYSEGDCGSTLCISETREDLAHAKIKKCHEIHVKLIRITNKLSKLIKNILGVYFTLSTISLCAIAIRLSTETNIIKVMVLALYTLSAFLQLFLFCYFGDNLQNMSSIGMGKGPYAAAYWALSPRVRKELILLAKGMSRPCHLYAGPFTRLNLPSLLQVKKSYISSR

>CsupOR20

MSLASSSVSPHLTLLRRVGYCRLSPGVTGSSSTRYLHEIYRKFALAAILTYTSEQAIYAYQNRRDIEKLAPVLFLFLCHITCIVKKLVFHLNAPRIDQLIAELEDAAYNSQTPPHRAMLRSTASSALRLLRAYVGCAIFTCILWVMFPLVDRLQQQDIEFHFWIPVDYNRPLTFPLVLIYSYYVTTLVAVGNTTMDAFIATMLSQCKTQLSILRMNFEDLPLRAMEMAYNSTSYEAALSKLFLDCIHHYQKISETMNELLRVFGLAVLVQFAVGGWILCMAAYKLVSVNILSFDFVSTTLFLVCILTELFLYCYYGNEVYVESDLMVQSLYSMEWVHTPLAFKRSLLLTMERAKRPLRPAAGHLIPLSLDTFVTILKSSYSFYAVLRQTK

>CsupOR21

MFIKNPNKSVGCSLSAMMMFGFWLPKNLTEIERILYQCYGCFWFILILGCYAATQIVQLYFDLGILHLMISASFLLLTISTEIIKLLNIVYRRRMIKSMIDDFDQVLRSTDTEEARAIVKRCDRETTILLAIYAIVTIITMVGFAAAADKGMLPIRAWFPYDVTKHPNYEITYTYQILALSVDAFLNVSTDTLVSSLMAQVRCRFQLLGLSLRNLCQGIRINEPLLASDQVVIVKDRLRLCVEQHCATLEAAQKLQDYFSFPTFMQLSVSLLIICVTAYQMNAVIGKPMAFIGVAAYLLDMMLQVFLYCYQGSMLSEESIAIADAAYECPWYVMPVPLRRSLLIVMTRTRRVAKFTAEGLTTLSLSCFMGVIKTSYSMFSILQGME

>CsupOR22

MLYLIKKFLVPAICLGLTFMATGMEMMFVLHGIQIKDYSFATECFCYCVMLGIIPVFYATNLHKKKYLLQILEDMAEDFVFICKLDTKLRNHFIKGQLLIWKLYLSWIVFICVMGILYVGMTLLPLTYQSLFATLDEHMVRPLIFPIWLPKDDPHRTPNYEIFLFLQLALVLLYMKAFGFYVYIQFHVLLHNKVLLELIIMDFDTLFDGLDEFVAMLPNNDMRRIAVQHTLNKRLERIVTWHNSVFKSIAALSSVHGAPLMYQVGFSPVAVCLMLYQIADKLDHGSFDIIFTALFFAACIQLWIPCYLGTLLRNKAFDVGDACWNCGWHETSLGRLVRMDIIIIILRSQKPLSIKFIGLPNLSLETFSSVGITNTRLHIIL

>CsupOR23

MLDENGKKCFENSLRRTKLFLSLSGIRISATKWPKALEKLFDTYFYYFQVFWLYADVLGEISWLIEGVLNGSSFLELSLAVPCITVSCLATSKSIFLYLNRDVVVKVIDKLREIYPESDETLKYHSNADLSHDKELDIFEDDSNKSDINTDIERDIKNESVDFLNLVVKVQYYICSAVVVAFPLMPVSTMVFIYYSTGVLEYKYVYMVKYFFDPFKMALWPFVYFHQVMSTVIVAMNVFGSDTLFYAACIYIQMHFRILCHHYENAVSASSIQTRLNLKVAIRRHHELIDLVNRVEILYTKSTLFNIVTSSFLICLSGFIITMVEDIIVMVTFATFLFMNLSQISLLCYFGDMLMSSSTQIVNAIYNSLWYDADERVKK

>CsupOR24

MVHIYFQHPRLALLLTGLWTPPKEKKFRLLYIAYRICVISMQYAFVTCNLVNMVMLWGDLEQISDACYLFFSVATCSLKNTNYLLSQKKFLSLLDFMENEVFVSQSLVQDKIISAYAKKMGRIYLIYIVCGFCNCVEWALVPVFEKEGHKIFPFKIWMPMDAASAEIPEYLLGYVLQLFGIIFSVSTYLTMDIVAISLLMFVPVQLDIITCKIKEVQHVSILFDPKQRRDLIEHNSALLKDCIRRHQALLRYIEGVADIFDIHIFFQLSATVIVVCIIALQMTIEPPNTFHYYSTVNYLMAMLVQLCFYSWSGNEITERNNVLRDGLYECLWYEQDLSFKRTLWIAMEFMSRPLIFKAGNYIPLSRPTFVSVGLQTWL

>CsupOR25

ETGGTWYTRADYLDFNLKCLFYAGLWPNEKWSRNKQKIYKIYEVTLFIMSFTFMFITSIGIYMAKSGDTIFFFADVDKNIVTYNYIFKIIAFFTKRNEVKTLINYIIYSGDRITDQRKKLMVTHVIVVTGMILALTGVFQILALMKGELIIVAWFPFDPMKNQWSLFLAEQLLVILFAVPCVFRAISIQGIVCSIIMYICDQLTELQSRLKNLNYSVETAAETKEELKLIIKKHIRLMGYAQSLSYAFKEYFLIQNLAVTAEFGLNALMVSIVGADQKKHLLSFIAFLMLALVNAYIFCFLGNKLMYESTCIALAAYESSWISWPVSMQKDVLLIITVAQRSFKLTAGGMAYMSMQTFAQALYNGYSMFAVVRDLVN

>CsupOR26

YLRVCGFYRLDQSSSKSVKILHRIYRRLVLSFFILYTIQQLLKIYDARSNVDKVMGTMFLFLTNTDCIYKAVILWKKADHIEGILEVMKGPIFNKGEPGHRLFLQDTIRKTLLVFRIYNYMSLFTCFLWVLHPTVLHMQGKLIDLPIRLPFDPNTKYYTAALYVWIQTSWLAYCNTAADVFISILLEQCRTQVTILRYDLENVVQKSKEEATETHGNYGDILERKFREMLLHHKEIVKTAGEILDIFSGAVFYQFLVSGWILCTSAYKMVNMNPASIEYASMISYIICVSIQLYVYCYYGNEINYESRRLTDSAYAVDWLEIPVRQRKTLIIFMERIKQPIEPMAGTIIPLSNSTYVSILRSSYSFYAFLKNSSN

>CsupOR27

RDEESNKIYQCYRVVLLTLFKFVFFISFTLDLFFTPINVGLIVSQSLLYFSELAGLFKIFMVLFKRNSILEVFEILDSKEFLAEDEASQQIIQKSHIFFRKYRTACTLFYCTGVIIFFVPVIKYWTTTGAQLKLPSFQYYFLNDNVREKYSMYLFLYQHCCLVLVVLSNSASDTLICGLIMMATTQFEVLNWKMSHLALRPSEKHYDPKEEEIIIKDRLNKCLRHYDVILRYCKEVQETTGLSLFAQYTTGAITICISLSSFLIPMTHEDFVILVCYIAGMTVEIFYPAYLGAELTEKSENLIFSVYCGDWISRPESYKRSLRLMLERANKPVVITCLKMIELSLITFTSVMKSAYSFFTLLKCLLERQQ

>CsupOR28

VWIPPTNKSLLHKLYRSLMITLQYLFLIFQIIFIIQVWGDLETVSQAFYLLFTQACLCLKVSVFHVNVDKLRELLKQMNGEIFQPQSDRQKQILSKQASRIKALLLAFMVSSQFTCSLWAMKPLFDDVGSRKFPFDMWMPVSPEASPHYEIGYAIQVLTIGMSAYMYFGVDSVALSMLIFACAQCEIIMDKIMSVTSINYAMKNKERQKIFAKNRKKLIDCVKHHEALYAFTKLSEDAYHSYLFFQISGNVGIFCMTALRLTVVEWKSVQFFSMATYLYVMMGELFVCSWSGHELTSTSEMLHTAMYDCPWYEQDVRFKRDLCFAMMRMSRPLVFRTGHYVSLSRQTFIAILR

>CsupOR29

RGSCIWSSGVPMAITFVLCLIYRFELRGFLEEMAFKDEMQAMPLIQHVNSLTEGNLLYELKELVRMSQMKLANFSRIFLKVYIMSVLVIATLYPWSSIYEMCVTEDDTLRLIGFDMWFPWSLDDISVYVMSFLFNVYLGCLCSIAYPGLQTTIVLFLGQLIRQLRILNFILSNLSDLADEIVGDQNHNDIWQEVCNSLLCQCVDHYVKLKSFSNRINLTFHFYYLALLLMATVLVCMCSVKIAISDKLALDTMKYYMHGFCFIMMVLLLCTLGQQVDNECEKLEESVTNKWYLYNKNLKVNIQIFKMALDQRMPISIFGSATLSFPTFTWFIKTGTSFFTLVMSVLDN

>CsupOR30

FIKGIQDRHSTDELVNTLFLFLTTVTAIVKQVAFTVRMKRIKQLFDTTDGELFSPKNSAHLELVEQNEKYMRRLHFLYICTVLSTCAYMSLYPLGNKAFGQDHQDINYRIYFPFDPRKSPIIEVFACSYFGIALTLQGYVNSIVDCTIVAFYGQCELQLKLLRYNLEHLTDLDDVDLQEGTINENTLSYIDDNLIKKRLVHCVKHHQKIIWFLSETQSISNEFVTLQLSVACWTICMSVYKLVTVDMFSGEFFLTIGYLNCMLMQFFMYCYHGSQVLVESEFIAESAYCSNWVDISPRSRRLLLIFMMCCTRPLVICAAKIVPINLESYLAVLKASYTLFTILHKK

>CsupOR31

LHKMDTSFEVDLHRHTSLHIAMMKFFQIWLYIPHPRTSYKYWLSVTLRFFVGLFIFVIPTTAQFMYLFSIIRSDDFEIQEVASIINLVITEMLTSLKLLALHLRREDFLDLMKQLDGKQFVCHTKNHKKIVERSIHFSRGIYIVLSICTFIDVLVHMVVVPAVHRFQELPLKMDLIFFDVNDPSYFPYVYAFQISYKPAMVTTFVTLNTLCWACMCCAISQLDILINKLENMKKLIKDTKVDWHYDENEAFDKIFGGIVQHHLAIIRFTKVLQSVLGGQLTLSLFMTAIIVCTTAIEILSIESPRKHITELLWMLVFVSIIVGNLFADCYFGNAITDKSV

>CsupOR32

MFVEKPGMIKNGIRYKPECSVSGALGLVLRLSQLVGIGPLSFRKRNRGWFVSLSPSLCLVSYVAATVLNTAALTGILLDLQARPSKSARVSSPTLKFVWVSDYIVVLVIASVAAYGAPRRLTTTILCLARIQKINTGVSSKSSNDWKTSLLLMAFLLYVACVLTADYCIFLRAVFLSDRAFTAACLYSFYYFAYFLLVLLEMQYVFSALEVSKTMGRLNKLIGEVEHMLTMHYASLKKIESNDPIKLPLKYDNLMIDSMDTFKFDSGMTVGLASKSISETIRRLALTYMEVCEVVRQLDSSHGVGVLLLLLSFLLHLVITPYHLIVKITCLY

>CsupOR33

VSCPHTTVELGTREQQQKDLTNTTFFMPMKQEMSLAGRSVAPHLLLLRWCGFCRLRPRHASAGHPSLLQRAGRRLHAAYCTFALAATSIYLMQECVYAYQEHNDMDKLARVMFLLLCHITSIIKQVVFYTDADRIDDMIAALDDRLFNPKESSAQALLQGTARSAKRLVRWYSSTAVATCVMWIIFPIMYYVSGHQVEFAFWITVDHSGPLMFTVLIIYSFYVTTLVGIANTTMDAFMATILYQCKTQLRILRLNFENLVETANKIVAANPQESYENVLMKLFLEYLEHYQQISETNNCLQDIFGTSILVQFGIGGWILCMAA

>CsupOR34

IYRKIINYISHVEQKQIADGDLEINQIVSEYISRSRRFCYLFWALPVFVDPIMFLQVFITSIRFGKSTGTYPKILDIYIPYSDYPPGYYFSLLIQTTIGFTMSAYIVSWDSLVCVTMIFFAAQLKITRLMCSRMIDPQNPQKSHDNIVECLKFYTTLIEKQRIFNKLISPVMFVNLFVTSINFGISIIEIARVEDDFATLASGCTYLGACLIQLLIFYWYSNEVTVESAKVSYGVFASDWPLISNKYQREVALLGVATAKTLVFEAGPFNEMTLSTFLGIIRASYSFYTLLNKTN

>CsupOR35

SRFVTYNSLPLIIVVDSFPRIIMYYEHEILGEELVYLYPFDGWYPFDKINWYYTIYIWESFMTCVVIFIYGFCNIIHAAYTALICMELEILGNHLENLITADDVKNISRGRNAQKTHENIKSKLKNIMDRHQFLARIAKELDNALGDIMLLYYIFGAIIICLAMFTAIAVDDLYKTVRYFFMCCYLLWEVFFQCVNGQILSDHSLELSTAIYTADWIYADKDTKTMLHMLMARAQKPFLYTAKGYTTMNLNSFSGICKISYNLFNLLRTAYS

>CsupOR36

GHRRVTPIVTVRFCSKMDIPAFEELVKQIKINFWLIGIPFNDVKLHFRFYLLLFSLMIMVAEECGFLFVEYSPENLLEITELTPCTCIGILSALKIISITPYRHKIFKLTESLNELYSETLENQAAKKLITKKIILMKNLVTYYFVLNVVLVSVYNFSSVVIMSYTYIKTGKTVFYLSYAILVPFSIDTWPTWSLAFIHAISSGYICVLLFTTIDALYYVLSLHICNNFSLLTEDIRCLNETNSQNIRDIVKKHQHLLKLSEDL

>CsupOR37

GVLYKSSGKLVMQIPFVAWYPFDETDIRYWPIAYFHQLWAGFFDASSVHGSDSFYSLSCVFLQIQFKTLQYDIEQIIPEETNINTPELYKSFRKRFMLIVMRHQELIRCVNVLEVIYSKSNLCIIAVSSIVICISAFNFTTSDDIIWRTIFLGFFIMWLLQVFSLCYYSNLISLSSTEVSNAIYNSYWYKANAEVMKDLLFVLRRGQKPCKLTAWGYSDLNLAVFSKIVSTSWSYFALLQTMYSE

>CsupOR38

HSRHVTYFFWALAFFSNFSIFSEPYQKNINSDNGDPVFKKIFDGYIPYSDYPPGYYISMFIQTVLGNIVSAYVVGWDTLICTIMIFFAGQLKVARLLCSRVINVQNPELCRKYIADCHRFHTTLVKNQKLFENLISPAMFVYLIVISVNLGVCIIEIAKIKNDTPTLISSCLFLLDCFIQLLLFYWHSNEVTEDSVLVSYGVFESDWYQAENKYQREVA

>CsupOR39

MSEETKMYEKLLKRVNTLLIMAGCHFDEADAKRTVIQRFSSRRYFCFNMVMLTIHLICDFTWLALEYKRASLIELTYFIPCVTLSLLACTKSYLLVKNGEHVIDLIKSTKILQAISGRFELRTKTERESKKSVTILTAFMNFNLVLYILGYILFAIGPLILT

>CsupOR40

MSEETELYETLLKKVDTFLIVTGCYFDEADEKRNVVQRFLSRRYYCFNILMLTSHLICDFAWLALEHKTASLIELTYFIPCVTLSMLACAKSYLLVKNGNHVTDLIKSTKKLKAISGRFE

>CsupOrco

MMAKVKAQGLVSDLMPNIKLMQAAGHFLFNYHSDNSGMSTLLRKIYSSVHAILIVINFLCMAVNMAQYSDEVNELTANTITVLFFTHTVIKLLFFAVNSKSFYRTLAVWNQSNSHPLFTESDARYHQLALTKMRRLLYFICTVTVLAVVSWVTITFFGESVRLIANKETNETLTEPAPRLPLKAWYPFDAMSGTMYIIAFAYQVYWLLFSMAIANLMDVMFCSWLIFACEQLQHLKAIMK

PLMELSASLDTYRPNTAELFRASSTEKSEKVPDPVDLDIRGIYSTQQDFGMTLRGGGGRLQTFGQQNTNNPNGLSQKQEMLARSAIKYWVERHKHVVRLVASIGDTYGTALLFHMLVSTITLTLLAYQATKIGGINVYAFSTVGYLSYTLGQVFHFCIFGNRLIEESSSVMEAAYSCQWYDGSEEAKTFVQIVCQQCQKAMSISGAKFFTVSLDLFASVLGAVVTYFMVLVQLK

>CsupPR1

MDFELKENRFRTIEFLHNRIVRNFLMPLGGWPCEVFQEKTPLFSRFFRRFIPIQGSCMIYGELNYIIQNYSRLNFFLLGHIYVTMFLTGVMIIRAILPNKKEYNDLVNFFYGEFDLEHFKHKGSYYQKASEIVYKFSYYYSLVMAGMMIYGMLLYNALPLYHNYNAGVLHRSNRVANVTIEFSVYYSFPGFMPEDHFWFVTFTNLYLTYSCTVEICIFDLFMALFVFQMIGHIMILINNIKNIEMPKTCHNIEGFKTQTNVTVELYDYEENEILRNKIVEIVNHHRFILRFVSDVSFLLGPALASTYLCHLISCCLLLIECSQLDPDALAQYGPITVIMFYQLFQISVLFELLGAKSEKLIDAVYELPWECMDVRNQRLLCFLLQRVQTPVQVTALGLTKVGVTPMVAILKTTYSLFAFLRSTV

>CsupPR2

MIYGELNYIIQNYSRLNFFLLGHIYITMFLTFVMLVRAVLPNQQLYKDMVEFFYGKFDLEHFKHKGPYYQKASEMVYKISYYYGLVMTGMMICGMFLYNALPLYHNYNAGVLHKRNRVENATLEFSVYFIFPGFMPENHFWSVTFVNLYFTYSCSVEICIIDLFMALFVFHMVGHIMILLNNIENVEMPKTHYNIEGLKAQTSVTVALYNDEENEIMRSKIIEFINHHRFIVSFADDVSSLFGPVLASTYMFHLISCCLLLLECSQLDPDALAQYGPLTVIIFNQLFQISILFEFLGAKSEKLIDAVYGLPWQCMDVRNQRSVCFLLHRVQSPVQVTALGMTNVGVTPMVGILKTTFSFFTFLRSIV

>CsupPR3

MLESTPLKNLETRKFYLLAVFLLSTFQMYETVDLFSRLYTILMINMMVGGMILFNLTPLYSNYKNGVFSKNPPENVTYAYSVTYSVPGFNFYEHFTLTTILNWIMSYDVSVNVCVKDLYLSFLVFQIIGHIQILKYNLEHFPKPKNQATNRFDAEENKQIRKTIAECVDHHRLIVSFADDVSDFYGPMLALNYMYHLISCCLLLLECSQKEPDALARYGPLTVIIFGQLISVSVVFEIVETKVKSTKYGANCLTLTLSMVPFSK
