# Supplementary material for: Identification of chemosensory genes from the antennal transcriptome of Semiothisa cinerearia
Source: PLoS One. 2020 Aug 7;15(8):e0237134. doi: 10.1371/journal.pone.0237134 (PMC7413487; doi:10.1371/journal.pone.0237134)
Supplement: S8 Table — (DOCX) [file pone.0237134.s016.docx]

>ScinGR1

MYCVLVPGVESARVEGPAEQVAPEAARAHDRPSRCLVGGAHAFILRVSSYFGLAPLKFESRSNGLSVSISSAMCIYSYILVTILVVCTILGLVAEVSAGVEVSVRMPSRMAQVVSACDVLVVVLTAGAGVCGAPRRMRTMLAFMGSVAMVDTSIGGKYSKSTERKLCALLLCILIFFSVLLADDFCFYAMQAKRTDRQWNVVMNYAGFYLLWYVVMILELQFAFTALSVRARFLAVNDALALTARHLYIPLEKAKSPSPLNVYAIRVSPPGPGGGVDPRANVSLLVDSLAAREQPVVLTKSVHGTAALGVSPSEAVRRLASLHGTLCEVVRCVDSSYGLPLIVVLISTLLHLIVTPYFLIMEIIVSTNRVHFLVLQFLWCLTHMLRMLVVVEPCHYTIAEGRRTQSLVCRLMTSVSERPALGSLPSRVELFSRQLLLQSVCYSPLGMCTLDRPLVASVIGAVTTYLVILIQFQRYDT*

>ScinGR2

LLSGCVLQQSKSGLYHEMYEHIRQRPTPVSNEEGVDWVANNETYAFLMESTTIEYEIERNCNVTKVGPPLDSKGYGIAMRKNSTYRQALNLALLKLQEAGELRRMKTSWWIEKNGGGACKVAEAPGSEPLGFDNFCGLFLVLAVGCAFGLVFSCVELAAGTARRAAARREPFSRRFLAELRFVFRFKDSVKPLHVSAARPARAPRAPRYSPPTCRA

>ScinGR3

VLAALFTASTIDLAIEKFANIEEILFMPVFMVQKLEVFLFCLMIATLRNRLDIINGYITKFLEEKDDIVSIFTLEYKDGIKTMNMIGRPSDSNMKIRDLALMYDSIGSLCSMINDNFNFQIFMTLVSTFCYIVITIWTSLYYYKTPEYNSDALISVIIWCVCTLVMVAAMCIVCERLLSMRNSTKILVNQLIMDYSLPKTMRVQAKAFMELIEAW

>ScinGR4

EEYGRATQLVRKVDDVISGIVFISFANNLFFICLQLYNTLEDGIKGTGECSSRTKKTTLLGGYEAAIYFLFSLVYLITRSVAVSLIASQVNTASDVPAPVLYDVPSPVYCVEVQRFLDQVNGDKIALSGLQFFTVTRGLLLTVAGTIVTYELVMFQFNPPSPGTTVAPNITTTLPT*

>ScinGR5

HFQLGTDTYIQVQVWRKLREAYVRQSALVDQVNDKLGALILLSNVNNLYFICLQLFFGLGTERGSVINRAYYFFSLGWLLFRACSVVLAASDVHVHSRRALPSLHSYPDSGYNIEIKRLIQQLANDNVSLTGLGLFKLHRNLLLQVAAAILKYELVLIQYDYN*

>ScinGR6

LFSFVRVSPGRDPELAKTPVKHALQVSWQLLWCLYHGGRLLMVVEPCHLTRMEMETTREVVSHLMRCPASVYDPLAIELDLFFKHLLLNQASYSAMQATVISRSLVATILGSITTYLVVILQLQ*

>ScinGR7

LRVSFLWSVGLFIVLLAIVLGSIWKLVRLLGGMARNHSGGRGFIGRLSGLIFYGNALVSLLLTVRFVSSWAELSLAWTKANTESGLNLPPDIKIRKRVIFVTCFVTTCACVEHMMSMMS

>ScinGR8

TKYLDFLVYTCWEIAIALGIVFLVIVFSETTVGHAKKTSFIIHEIINEDFGPAVNEEAMKFSVQLLHEIPEFTVCGLFTLEYAYLQQATRSVSTYLVILLQFVTENK*

>OfurGR1

MGIMPIMRVPKDAQTTKRTTFNWISKATLWAYLVWGLECIIVVRVGKERLANFQQNTNKRFDEVIYNIIFLSILIPHFLLPVASWRHGPQVAIFKNMWTHYQLKYLKITGTPIVFPNLYSLTWGLCVFSWGLSFAVILSQ

>OfurGR2

AVYGALSEIVDHGIGFSFKEIGLFVDTVYCSTLLFIFADCSHNSTQKVADGVQETLLSIDVLSVDRPTQKEIDHFIQAIEMNPAVVSLKGYANVNRELLTSAISMIAIYLIVLLQFKISLPKDPQMPAT

>OfurGR3

LVRMVDAQIGALILLSNINNFFFICLQLFLGLNKTGGSLMSYFYYFLSLGWLLFRACSVVLAAADVHIYSRRALEYIRLCPDSGYNVEIKRLNNQLSHDFVALRGMGFFWLSRQTLLEVAG

>OfurGR4

LFRGYEQAIYFVYSCVFLVARSLAVSLIAAQVHTASTKPAQALYRVSSSAYCVEIQRLLDQIHMDTVALSGIQFFNVTRGLVLTVAGTIVTYELVLMQFT

>OfurGR5

KTTREDYCRATQLVRRVDEVISGIVFISFANNLFFICLQLFNTLEDGIKGTGECSSRSKATSVKLLGGYEAATYFLFSLVYLISRSIAVSLIASQVNAASMVPAPVLYEVPSPVYCVEVQRFLDQVNGDNVALSGLQFFSVTRGLLLSVAGTIVTYELVMVQFTTPSPPAVSNSTSGVLANLTSTAMPTIATG

>BmorGr1N

DIYGPEITDKDDGALLDKHDSFYLNTKSLLVLFQIMGVMPIMRVPKSAQTTRRTTYNWISKATLWAYLVWGLECIIVVKVGQERLANFQIGSNKRFDEVIYNIIFLSILIPHFLLPIASWRHGPQVAIFKNMWTHYQLKYLKITGKPIVFPNLYILTWGLCIFSWVLSFAVVLSQHYLQDDFELWHSFAYYHIIAMLDGFCSLWYINCNAFGTASRGLAINLHKALEAEHPALKLAQYRHLWVDLSHMMQQLGRAYSNMYGIYCMVIFFTTTISLYGALSEILEHGLSYKEMGLFVIVAYCMTLLFIICNEAYHASRKVGHEFQDRLLNVNLGAIDRSTQREVEMFLVAIAKNPPIMNLDGFTNINRELFTANISFMSTYLIVLMQFKLTLLRQGARKTVTAIVRAIFNTTITDNGAGGSDEDQE

>BmorGr2NJ

KEQEQRDLLSSQDGDTCEIHDQFYRDHKLLLVLFRALAVMPITRSRPGTITFSWKSTATIYAVCFYIAATAVVLIVGYERIQILQSIKRFDDYIYAILFIVFLVPHFWIPFVGWGVAHQVAIYKTNWGKFQVRYYRVTGENLKFPNLKTLIVIISVGCLLLAVCFLLSLCALLDGFLLKHTSAYYHIITMINMNCALWYINCKAIKIASQSLSECFQRDVDIECSAQLIARYRYLWLNLSELLQSLGNAYARTYSTYCLFMFANITIAVYGALSEIVDHGFGFTFKEVGLFVDAAYCSTLLFVFADCSHKSTLKVAAGVQDTLLSIDVLAVDRPTQKEIDHFIQAIEMNPAFVSLKGYAHVNRELLTSAISMITIYLIVLLQFKISLPKEPHGTGQ

>BmorGr3F

MSFEIKNNFFRTSVPIPNGFPVQTEAKSKNKPIFLDVSPAPTPKVNSPNAIIPMKNNLIDPFINKDIIYENIKPVFMVLRIMGVLPLTRTTSGVNEFHFISPAMVYSLTVFIILVSYISYLSLHKVQIVRNSEGKFEEAVIEYLFTVYLFPLTVVPILWYETRKIANVLNGWVQFEVTYKQLSNRILPVKLYKKSLLIAIIIPILSTTSVIVTHVTMVHFKTSQIIPYVFLEILTYMLGGYWYLLCEILSLCANVLADDFQQALRHVGPAGKVAKYRALWLRLSKLARNTGVANCYTFTFVNLYLFLIITLSIYGLLSKISEGFGTKDIGLALTALCSVFLLFFICDEAHYASHNVRTNFQKKLLMVELSWMNTDAQTEVNMFLRATEMNPSQISLGGFFDVNRTLFKSLLATMVTYLVVLLQFQISIPDATQPEIPTNIDDHVQNITDTTTEASSPISTLMSAFAKRKND

>BmorGr4S

MDKDKFQEFLPTMSRIFSMTRYFGVSTCKPSIAFGWTVILLLMLLAIEVGAIWKIVRLLGGWAVHSTDSRGFTARLSGCIFYGNALLSLILSIKFVSSWEQLSERWSRTETDPGLRLPSDSRIKRRTVLVSAFVMTCACVEHMLSMMSATGFDCPPEEYTERYILSSHGFLVQNDEYNLWLAIPIFIMSKLATALWNFQDLIIILISMGFTSRYNRLNTYVHRVVMLERNLKEGAQVSSENYMRFQIWRRIRQAYVRQAALVRLVDDQLGALVLLSNVNNLYFICLQLFLGINSKDRGSFINRLYYFISLGWLMFRACGVVLAAADVYIHSKKALISLYLCPELAYNLEIKRLKYQLKNDEVALTGMGLFSLNRELLLEVAAAVLKYELVLVQYDK

>BmorGr5NJF

YFAFYLSTGCNTFIFLRVASKWPTLIKHVYETQLDSYIDVKVKNKCFAAYIIFFSMSMTEHMLSLLSKFVITMDCLPKGSDLFESYIIRNFPWLFEFDVPYYLPIGVILQFLTLVSTINWSYSDLFIVCMSIYLTSILKQINKKIEMAGNSNHLPIPFWRTLREDYTRATRLVRSFDDTISSVIFLSFASNLFFICLQLYNILSNGVTSKYNLLKEMCPNYPSGPLGGYEQIMYLLFSLSFLLGRSLVVSLVAAKVHSASMVPASALYNIPRNMYCSEIQRFLDQVHGDKVALSGLRFFYVTRSLVLSVAGTIVTYELVLLQFSNED

>BmorGr6P

MLLRNYKQNLSFWTSAKKSKIHKIQSQETVTFQGSLKLVLFIGQLFSLFPVCGLLSNDANKVXFVPISWKCGYSMLSMIGQLFIIVMCILYVAHFETTLNGTTPIIFYGVTFISMIAFIRASRRWPELIQHISKSEELDPSFDFRLKKKCNITLLLVLVLAILEHIFSIRSAYSASQICYPHTGFYEGFVRYLYPWVFDFLPYSEELGMVTQFLNIQSHFIWNFTDLFVICMSYYLTSRLDLVNKKLLPAQGKYLPEIFWRTTRETYCRATKLVRKVDEIINGILFISFANNLFFVCVQLFNTFDDSVDMVGLCYNYSERRTKPVGREPVIYLLFSLGFLISRSITVSLIASQVNLASTVPAPILYDVPSAVYCVEVQRFLEQVNGDNVALTGLQFFSVTRGLLLSVAGTIVTYELVMVQFNQAPASDSFTEKLVENNISTIETFYNYS

>BmorGr7

MVLEAHTQIQYCTAKANYCEFHAGLRHLMRLARWAGFFPVQGLSQTNPDDVRFEFRSLYALYHAITVIGQTVMTFLAFYSFVDSNVSLSVVSNFLFYFTNYVTLVLLWRLSKNWSALISKTLEFEQSVTEIRTTRNLVSRTNTLTYVVLIFAMVEHALSKVFNIRSVMCCLGETSLNHTVINNYFKFKWKFVFDYFSTSTTYSYFVGFIAEFLCMQATFLWSFTDVLIMCFSIYLSSFFEDFNSTVSSFMKKASKTVPWSTLRVQYSQIVLIVKQMDEQLDYFVLISYFTNLFFICFQLYNSLNRIYDANDVCNENMDIIATASVTYLTYYVFSFLFLVTRALLLSIMAANVHSCAQVPQLALYEVPTADYSLDVQRFQLQLRYTTVGLSGVCFNVTRGMILRVIGTIVTYELVLIQLTKKNLDNDTSIRDYYLPKHLI

>BmorGr8R

MAPRSVRSMVGTSKKDMLKGGFYETVRIPLYIYRLIGILPISGLWHRSSKYNRFSLKSFYTIIYAPTIVMQTFLLLVHIYDLFAFFFGHQRLGRLIYHMNFYTITILIFMGSRKWKNVIKEIETIELTLPRLRNSKKALALTKSFVFAFFVFSLAEVVLILQFTLRLTKQRHVLPGDSGLYLRSYFVYIFPYLYDHFPFSYVMGFIVQIIKVQGIITLNMVNCSVVILSIYLTNRLKHYNRIVFAKGSKTNNTRLKWVELNLLYTRISNLVKIIDKNLNPFVFISFTANLSYICAQLFYILNKLTSSRTVKITSFLEDKRCDWETVLYISISFALVVLKVLLVSIIAAEVHTTSREPLRLLYTLPTAEYTIETQRLMTQVYYSNLSLSGLNFFHITRGMLLGMVATLLTYEIVLLQI

>BmorGr9R

MPPSPDLRADEPKTPCLVGGAHAFILKISSFCGLAPLRFEPRSQEYAVTISKGKCFYSYILVTFLVICTIYGLVAEIGVGVEKSVRMSSRMSQVVSACDILVVAVTAGVGVYGAPARMRTMLSYMENIVAVDRELGRHHSAATERKLCALLLLILLSFTILLVDDFCFYAMQAGKTGRQWEIVTNYAGFYFLWYIVMVLELQFAFTALSLRARLKLFNEALNVTASQVCKPVKKPKNSQLSVYATSVRPVSCKRENVIVETIRVRDKDDAFVMMKTADGVPCLQVPPCEAVGRLSRMRCTLCEVTRHIADGYGLPLVIILMSTLLHLIVTPYFLIMEIIVSTHRLHFLVLQFLWCTTHLIRMLVVVEPCHYTIREGKRTEDILCRLMTLAPHGGVLSSRLEVLSRLLMLQNISYSPLGMCTLDRPLMVTVLGAVTTYLVILIQFQRYDS

>BmorGr10

MTMSIKPRLQCMVPPSLALALRVSRLAGIAPLKFVAKQSNIMIRLSTSLCVYSYLLVTALNVCTLIAVMIDFSVPVKLSIRMQTETKRFVWIADVVIVGMLSGVGVYTAPIQMRRLIAYLHRIHKINSDLQTYSSSLTDKMLHRLTIGMLLITSVIIVTDFTFVMYLADLNHRQLLIAIMYWCYYCSYFIAHLLEMQFVLIAALALSSLKLVNNGLRTMLHQSGIESLTEIPNSNEQHAANAVLPQPPKKSVNNSIDTLAFVVTKRSVRFPTAGWTDQRTIRRLALSYGSICEVVRQIDNNNGIIVLLLLASFLLHLVVTPYYLIISFVTESPHTGFEKVLNPILQTVWCLYHTFGLVMIIEPCHRTHEEMETTRELVSRVMCSADPRDPISVELEMFFRQLVLNKASYAPLKVCTLTRSLVATILGSITTYLIVIVQLEIKNMQ

>BmorGr11F

MKPFRFFLFVENVICVYRNYSFHKRYARAIILSRVMFEVSLIILTLHSCRNFGAVKYKTEIIFTYLATASSTILILLALYKTNRFTELFLNFKAFYRNRNLDVDHLEKWNRKQKMATVIIVLFCVIKFSTLIYTDLIGEYSTPCRGYFTEYLFYTNLFMCNARYLFEFSTACVVLHLVSEQLDYIAISMDCTMFLYIDISKKNIMSSAKKRKLKYFDIFKQFEKWTDAYMNVKRSANLCDTVFRAQLAIMITTITLYYIILLYGITSFNIERGKFSVVKSLSYLISLFGFLIALLLLSKAGQRIQKSAENLRRKLSKFLLHSLEDPEFHRAATNLLRLVCTHHIKMRCFGFIDIDMTLLPSCLMFVTSYTVIALQFNNVV

>BmorGr12P

MKNLKLCRTTFYFKIIMCSRFISGLYFTATSKKWISYLYKVICVLYIICITRLFYAKEDTFKPLVFXQFIGNSIESLRTGEGHVLKCYSTIFSLKLIRNYLPDSNNHIPISSITNFLVIIWKVFDQVYIVLMHYFYTTDIIIHLRILSILTTIGVNLSLMPIIVIFELMWRAVKALRKSLGEHLKGPVLIEGRERLKAQQILRCLNVYKDLNATLKFNSTPMKTMILISTLATFIRLTLFLYQAILGHNEGLHLPRKILAIIYYALPVCLLGVLMELVARECDKLKTLMTKELLVCKDDSYCTVIVDAVSYIELNPLKFSILRAFNVNSTLILGLTNLCTTYLIAVIQFTYSCEDINGLSHSHSH

>BmorGr13

MEDSFNRLLSIRNMIIFQNVCGFYHMCTEKLYISRIIKMYCVALAIVLSVFCFQNPDITYLSWDVVWVTFGYTLNVIICLRYNGNYFFQYWNGLHEIDIKMNLTSIDKEKVPISRAVFTVFLILRSTAFAMTIFVFGYLETGILSNTIISIYSINLTEFYRNMSNIPMILMFETFYVRIKILKEQLCSELSTVLGCNNDARQLKLILKYLRNYRSLVRHLMDTTLPFKILILVILVGSFLRSLLIGYAFVYNSDQIILLSLPVMFSTKILSEVVEIKLICTKELLKNKNEGLVLLDLDSKKPTFLTSKACGEQLQDALSFLNNRSYSYTLLQVIEFDCSLAFVFTSFCITHLIVVVQFTHVLD

>BmorGr14

MNLHKNIIPIRNNLFANKVTAIALPKTLSVLFKLIHIFFLLDLGVYEYKTFKIKCIVKFLTISGSLTISVVCFSFMVSNLSEHTFVGWYGFFISTYIFVVLFFNLSNRMTFVEFYKTLLRFDANYGIDSNEYKFNFKIIFVNILFIANRMVLSFVYCSYYPQNCIRPRYAQILFMLPWLTLDVLLTTNMFLFYATYCRIAKFPMLIKNSMNIVALRNSYKLIVDSLEKTQTSFDIVFIIALVFSVPEIMMSIYSTLLEVISKHFLEVASILSLNYVAIAQSLLLTLAPSLCAGVLPWKTNNIKIILHEKLFTEKDKASAREIELFIKYIESRPLKLRACNLVPLDFSLTIIVLNICVTYLIVIIQFTHLY

>BmorGr15

MISSSDINHKRNKVFAYNVPGIALSKTLTVLFKLLHYVLLLDVGIYEYKTFKNKCIVKFLTIATGVSVSIVYFCLIATVLRKNAFFYWFYVLFISQYMIIVFIFTLSNGMSFTDYYKMLLRFDAKYQINSNNYYFNIKIILVIIISILNRIGMAIIYCSYYTKNCYEMSFSQIIFVLPWLTRDVILIMNVFLFYVTYCRITKFPALLENTKNVGSLRNSYKLIVDSLEKTQKPFDFVFTISLVFNIPEIMLSIYFTLLQVIHSHFLEVAPTLSISYFSITHSVVLILAPSLCAGVLPWKTNTIKIVLHDKLFLEKDKNSARNIKLFIKYIEARPLKLRACNLVPLDFSLPVIVLNLCVTYLIVIVQFSHLS

>BmorGr16F

MIMNLTTDRISKRNKVFAYNVPEVTLPTTLKVLFKLIQFTLSLDFGVYKYKTFKMKCVAKVLTLAGCLAASAACVSLIISNIFENQLFFGWYTLFVCQYTIVIFMFTFSNGMTFIDYKMMLLRFDAKYQIDSNVYHFNIKIVLVVVISVTSRLFLCAVYCIYSTENCIKPWYNQLLFFPWLSLDIVLIMNMFLFYATYCRLAKFPSLFENPKNVVPLRNSYKLIVDSLEKTKKSFDAVLIAALIFNIPEIMMSIYYTLFQVMNKHFQEVAPVLSLSYFTIILSVLLILAPSLCAGVLPWKTRHMRLILLEKLFAEKDKNSAREIELFIKYIEARPLQLRACNLVPLDFNLPVIVLNLCITYLIVIIQFTHLF

>BmorGr17F

MGFSLGTTALSMFFFEKPVVFTIIQITMIIVKPAKYKLSDPFRPKDTSKLSESIIMYFKLFHIFLGIDLGGFRYQNRQVKYAVRLISLIQPLAIYGLCIYALLKIIANTEFLWYTISFTEYVAMSVAITLFSNEMTYCNFMINLKFIDTKLKIGDESFRIGVKLISSTILIGVTRCFTTTTYCLLGFCAKPTAAQILFQIPWLTIDLMLLQYMFIFYACYCRLVKILRILKKRNTDIEEMRRIYKTLVDVLDRARAPFDLAYLLGLLFSIPDVLYSIYESIIKVGEINTAKALSMSIIYITNIQSLALMFAPALTAGFLPSLTMKMRIILHDKLLEEQDKKTYRHIVLFIKYIETCPLKLKACQIIPLDFSFPIIILNIVVTYLIVAIQLTHFL

>BmorGr18

MRRSTKVISMVNQSDKGEIKTCSRFMKIYFFVIYILTGFNFGFYTGRGLNFLRVIQASVLLLRFIIASNCIYIAFHFRLLEAIWYSLTFSESLAIVVCFMLSRSALSCKNLFEYLYSVDQELKKSVGPSIEVKLALYTVVVSVLRLTVYVFCAIAYYETLHEGFCVELVYNTPCYCSDLYLVIHFTIFHSVYCRLKALRISMNEKFDVYKGTLIYKSLIDNLEEIKKSLDVPFFVILLNAVAIAMINILVTLEISYGQTMKFIRTAPRYLETVLLFSSAFAPVLAADMMASEAQKIKVTLNNILQRDDSLLEDDRRKVKQFAGYVSARPFRLRACRVLSLDCTLPVTVLSICVTYLIVVVQFTHLY

>BmorGr19J

MRRSTKVISLVNQSDKGEIKTCSRFMKIYFFVIYILTGFNFGFYTGCGLNFLRVIQASVLLLRLSVASYSMYIARYSPLLEVIWCCLTASENLAVVVCFMLSRSALSCKNLFEYLYSVDQELKKSVGPSIEVKLALYTVVVSVLRLIIYVFCATAYYRKLFDGLRLELLYHTPCYSLDLYLVVHFTIFHSVYCRLKALRISLNEKFDVYKGTLIYKSLIDNLEEIKKSLDVPLFVILLNAVAIAMINILVTLHISYGKTMKLITAAPRYLETVLLFSAAFAPVLAADMMASEAQKIKVTLNNILQRDDSLLEDDRRKVKQFAGYVSARPFRLRACRVLSLDCTLPVTVLSICVTYLIVVVQFMHLY

>BmorGr20J

MRRSTKVISLVKQSDKGEIKTCSRFMKIYFFVIYILTGFNFGFYTGCGLNFLRVIQASVLLLRSIIASYSIYVAIHFRVLEAIWYCLTFSESLMVVVCFMLSRSALSCKSLFEYLYSVDQELKKSVGPSIEVKLVLYTVVVSVLRLTVYVFCAIAYYESLHEGFSVELIYNTPCYCSDLYLVVHFTIFHSVYCRLKALRISMNEKFDVYKGTLIYKSLIDNLEEIKKSLDVPFFVILLNAVAIAMINILVTLEISYGQTMKFIRTASRYLETVLLFSSAFAPVLAADMMASEAQKIKVTLNNILQTDDSLLEDDRRKVKQFAGYVSARPFRLRACRVLSLDCTLPVTVLSICVTYLIVVVQFMHLY

>BmorGr21

MAHRTNSINLFRSRPPDIRAGVGEPRIFSKFICGTMFTQKSLVNFDLGKTPRGGDQEHSKFFKIYFLAVHSVTALDFGFDRNAKKLTKILISMFSISVRMGLAAVSFMSLWGRPNALALGWAPGTLLCENILVAVTYSASRSTFKCGDLFADLSTIDELFGSACDYRIESKMLLFTATMTVLRVVIYSTSRLVRADGFDFVDVLEVLNNLETMCMYLFLTVYFFVLFSIYCRFKKLRELMKNDFEIRRANLIYIALKDCTDKIKQSLDVPFLVVLVFTVLVVMVDVFITLEMIISNKYNMAVYVVRYLEITLDFLMLFAPVLLADMMAVQVDGLKITLHDRLCLNNGVDMKRYSSLAEFIGYVEARGCRLRACRVVPLDLTLPVTVFNVCVTYLIVMIQFADLY

>BmorGr22J

MTRFEFSQRTKTKSVSKTLNILFNCIYVLTCMDFGFSLGVYKRMKVLENISLVLRVMVAIMCAAMVMKQDILDSAWADITLTESLLVIVSFKLSKPKLSYRELLENLSIVDETQGAPPAGYKVERKLITYIAGVTALRLTVLCLYCVAHTEQYSIDNFIEFLYNVPCYCLDLYLIVHFIIFHSIYCRLRTLRKALSNNFDVYRAHLIYKTLIDCTEEIKKCLDIPLVVILIATILVVMVNVLVTLRMLFKGESIISAFLLRYIEVILSLALLFVPVLVADMMALEAHKIHIFLHGRMQTANDAAEERREVRQFAHYVGTRPFRLRACHVLALDSSLPITVVSVCVTYLIVIVQFTHLY

>BmorGr23J

MAQFQIPSMAGSGLNVAPFSRGRRHEGPKHSNFTKKYFLLVHLVTCLDFGFHRDNDTKTYKWFHAANIGVRLVLSAYVCSVSLSQDLSFASAAWTILNNSKHLLVVAIFTIFKPKSSCAEILKDLLMIDEALKIHRGCDVKGQITVCIVLVTAARLLIAAASSLSLHEAFSASVGAAEVLYSFQSYCLDFYILANFFIFYSVYCRLKNLRRVLQNNFNIYRGNMIYKVLVEHMDDIKKFLDIPFVTSLLVTVIMAMINVLKTLQLIHDGENDVLTIVLRYLEMFLSFSLIFAPVILSDLMSIEADNINVVLHNYIYETDAAEERERRHLSQFAWYVSARPFRLRACRVLSLDCTLPVTVLSICVTYLIVVVQFTHLY

>BmorGr24P

MCINKKIQSIIKSLVSIRTIMLVQSILGFYHKMSNNFFVSFLFLTYTTILISVLSFYSVNDVMAHKFAYTLSMILEYDINTILSLITAGRQYFNFFEEMKKIDFSIGFGELNIEDLPLSRTLFVTIFVTNILLSIMTAALILFFSTPFLIISSGSTYAMAIVFFGLSLNVLPRIIIFELIYKRIKYINLSLKRKLKALALECDHTIARFEIINENLIIYNKLLQSLGNVNVSLKSSILLTTFTCFFRCSLICYYVITMNEDKVYIMQIIELTKQTLFLGVLIILAEYIKNEIENLKMTVSLQLFTCTDTQLYHQVSDXFEIHRITPIQLCGFKNMSVDTNLFLGLINVCSTYLIIITQFLNAYVN

>BmorGr25J

MFVKCLKYVKKFKPMFSVMFIMNFRLICGLYYRIHSDAFVCFVFKVYCILCSMFLFFTSSDLAAPFSRSIPILATLFEYVANVLDCILTGQSYFFHLRMELMRIDPRLRGLDRPPASSIVFTAILSYKIFILAVYIHGKARTTYLQYEWFSSIGIHLLVLFSNLVHMNRMLIFEMVTFSLEAQKKTLGELLKSSLRRERVERKCEILNRFLKTYKRIIELFNNTMAATKLMTLISVVSCFIRILTYLYQVLTTQLSSSSAGSIFTTRHLTFIVTFVREHVLSVYFLKKVGICQRLQINFKIKCNVKTETLELISQDDEYTEKLEDALDFINSCSSKITILRAMTVDATLPLTFISLCTTYIIVVIQFSHIYD

>BmorGr26

MNKTKIYRKKLDKNERLVCSVQPAMFARLIVGLYYDIKVSNRVKWMIKSYCISLSSFICYLIIFRDDNFSLHPKLTSVMEYITYVTFSFLTCDKYLFRYLRFNPRTDGYPIFLYLCKKFEKFFKIIICLFVSFKILGVVLMMQSWPILSTPKYIWGTLALHFLWLASHMGRLVFILVYGILFCRMRTIRIIFENRGFQNTPQNRLTPKRYILMYEAVLNSIESVDFPVKFLIFTFICCFAPKLVVSLFEIMEEMKKGELSLTTFIWFLVELSPSYLFLLLSAIALDLVSEDVQELLSITIDRRLNCKNEKERSEIQEFFQYLRNNPFNYTLWQVVSLNLRTLLVATSFSIANVIAIMQIKNSKI

>BmorGr27

MVFKYKIMTKAPKSLPVLKILMLFRLVFGNYFRLSSNRYINFLVKSYCSTFTILLSVMCGKRLKNDSPYMLSLTEYILNKILNYATSEGYIFKYCNSIKTCDKIMGFKKLPIITIDVFIAIIITVITRTAITIYFGFLFPFDKYQVVLYVGCIVFSNDLNSLTIMNVFGLLNNRMNLLRKSLEAMTVPINIIGKNEVAPKVRLVRNAFRYYSNLLDNLDSVNHCVQYSLSVTLLLKFPKAVLLCYDSIKTYFVKIDNNFAMDIVDPTEIILSIVVMSFPAMLCEMITNEVEKIKAILTKHLIQCSDNSLRFELNITLLYICHRPFKYILWRAIPLDTSVPIGIVSLIITYVIVLIQLLHFST

>BmorGr28P

MAHKIATVGPTNATATVKNNKRKLKISKRVTIFKVVRCLRFILGHYTELTSSKLKAFLIKCCSLLLAVIIIYAPLNYIKMAYVMGLIEYLLFVLLSLFTGDEYFYKFHNSIKSIDVLMGYKRGKIIDSNAIIFLLSVITIMRIVIIYCRSTVLAFRFTIIGVYLAIFSLRISYMLITVIFFAMYHRMKFLRKKFEIITIPVTIIGKQKVASKIRLIRKYLINYHHLLDCLRDINGGLQYFLAIMIACNLPKYIFFAYSAIKIQVLEHITIHSAVQNVKCZRILFVVVPAIFAELTTAEVERIIDVINRQLLRCTDEHMELELKVALEFIRRRPFDYVIWRTVPLNASLPIAIISLCITYVVIVIQLTQFHDNF

>BmorGr29

MYLRSKKSRFKLFSFERMIKILLMICGHYVQTDSSNVVSSIHRIFSIVITICLCPYFQFNPFFFHVIESVWYSILSQFTQYGFFFRYCSTIKTFDLLSGFKQIPLYTKRVCFFLLITLLVRLIIVLIHFSAHQTKLKTFCAFLIILSANTGHILMTIMFSILNTRMTLIQKLFANNPIPVNIVGKNQNASHIKRVRKGLICYNNLLDTLKVAEKEIQFTLTVTYLCHVPTIICYVYFVITVIYKSKFSGYNLIPMLDMILACMAVTAPALFAELTKNTVDKIKKILGSQLLRCSDESLRYELEITLEYVIQRPFSFSIWRAVSLDASLPVAMTSLCITYVIVILQLTQLRP

>BmorGr30S

MYLRSKKSRFKLFSFERMIKILLMICGHYVQTDSSNVVSSIHRIFSIVITICLCPYFQFNPFFFHVIESVLYSILSQFTQYGFFFRYCSTIKTFDLLSGFKQIPLYTKRVCFFLLITLLVRLIIVLIHFSAHQTKLKTFCAFLIILSANTGHILMTIMFSILNTRMTLIQKLFANNPIPVNIVGKNQNASHIKRVRKGLICYNNLLDTLKVAEKEIQFTLTVTYLCHVPKIICYVYFVITVIYKSKFSGYNLVPLFDMILACMAVTAPAVFAELTKNTVDKIKKILGSQLLRCSDESLRYELEITLEYVIQRPFSFSIWRAVSLDASLPVAMTSLCITYVIVILQLTQLRP

>BmorGr31JI

MYLRSKKSRFKLFSFERMIKILLMICGHYVQTDSSNVVSSIHRLFSIAITICLCPNFEFNPFYFHVIESVLYSILSQFTQYGFFFRFCSTIKTFDLLSGFKQIPLYTKRVCFFLLITLFMRLFTVLIHFLAYQSKFVTFCAFIIMLSANTGHILMTIMFSTLHTRMKSIQKLFANNPIPVNIVGKNENASHIKRVRKGLICYNNLLDTLKDAEKEIQFTLTVTCLCHVPKIICYVYFVITVIYKSESLRYELEITLEYVIQRPFSFSIWRAVSLDASLPVAMTSLCITYVIVILQLTQFRP

>BmorGr32S

MCYTNFVSRQVSKCINFFSTIRYVIYLRMFCGLYYNCSSSFKIRCIARLYCFIIYCLNLHYNLYIFTSSVSLTNFLHTFITLAEVSIHILFSLYTGESNFMSFCIEMNKLTSGPIDFVATKCVATHFIAFFVIGLHILSSTLICGAEVSCFTFSVVLASMTFLTTLLSRFTTIIMFDLVWIRMRSLRKILVNALESDLSEDEKVKSIESFLKAYKQIIASIRITKLATRNLVTFNFVSLFGKIMTLIYFCINCPGYLNTYLISSWIFGILLAGFVTCAPPVLVEMNVNELDEIKYALADQLVDYTDDNYRTAIYNALDYVEVHSIRYTLWKNFPMDLTMFFGFAGFCATYIIGLLQFTY

>BmorGr33F

MCYTNFVSRQVSKCIHFFSTIRYIIYLRMFCGLYYNCSSSFKIRCIARLYCFIIYCLNLHYNSYIFTTNVSLTNFFHTFIILAEVSVHILFSLYTGESNFISFCIEMNKLTSDPNEFIATKCVTTHFIAYLVIVSHILSSTLICGARASCFTFSVILTSMTFLTTLLSRFTTIIMFDVVWIRMRSLRKILVNALESDLAENEKAKSIESFLNAYKQIIASTRITKLATRNLVIFNFVSMFGRIMTLIYFCINNPGYLDTYHMSLWIFGILLAGFVTCAPPVLVEMNVNELDEIKYALADQLVDYTDDNYRTAIYNALDYVEVHSIRYTLWKNFPMDLTMFFGFAGFCATYIIGLLQFTY

>BmorGr34J

MCYTNFVSRQVSKCIHFFSTIRYVIYLRMFCGLYYDCSSSFKIRCIARLYCFIIYCLNLHYNLYIFTSGVSLTNFFYSFITFAEVSIHILLPLYTGESSFMSFCIEMNKLTSGPNEFIATKCVATHFIALLVIVSHILSSTLMCGARASCFTFSVILASMMFLTTLLSRFTTIIMFDVVWIRMRSLRKILVNALESDLAEDEKAKSIENFLNAYKKVIASIRITKLATRNLVTFNFVFMFGKIMTLIYFCINNPGYLNTYLISSWIFGILLAGFVTCAPPVLVEMNVNELDEIKYALADQLVDYTDDNYRTAIYNALDYVEVHSIRYTLWKNFPMDLTMFFGFAGFCATYIIGLLQFTY

>BmorGr35S

MYSSLKLKDYIVNMESVMCSDQSITISFKNVFKVFFDYISSLDFMMVCRLCFGYYYEFNCSNLCKIMFKCFSISVCIFCVSMHLVQLISPPYLNHCVIMLESTVSIITSLVTEDKYFFEFCLDMKDINSMMNQPRNIKSFKIIYVIISGAICHVIRHISICREKALSFCFSTEYLTASFTIISGYWNYLNITMMFDLLYQRLVAVKQMLTNGLNICDTDEYKIKSVQKFIDVYKALTVSLSKTSNLIKHTVSLGIFCALWRIIFFVYYCISMDFQVESAQFITWVSSMCLSVFLVYIPALIVELCSNEVDAIKWILASELLEYRDKRLRTSLRDALDYIDVCPIDFEIWHCFPMNLSLCLGFIDISSSYIISILQFKY

>BmorGr36JP

MTVPYDKIKSALSKFVQLLFSINVVLFVRFLFGFYMKIGSRKYFHIATKIWIVTLTIFRVYFQCRNFMNYPSYALILHDFTCTVELVILCIISSLGGEQHFYTYCSEMAELIDNRKNKRASYFTTSTLLIGFIILIVPTSISCKKVSNALSMLFLNIFNYVACFMHHLTIIYVFELLWREIRKCRISLERLEIMSVDDKIMKIENFLDSYKRSLDSLNKANGVMIPTMALAYFAIIAKIVFFTYNILSLRGFNILVHGDSWLLSTSIAIIFICAPALLVELAANEVNKIQNLLAVELLKMKDDKFZYRATLYDALDYIEVRSVKSMIWRNFPMDLNLCVGFINLIITYVIGLLQLLY

>BmorGr37JI

MSAKINYQEKLCSIKSIMYLGLFCGLYFRSSTSRMMLLMTKVYCIVLLFLGISFHLNMLSSDLPTETTLHSSIIMLEFFIHIITSLSTGQAKFLHFCTEMMKINGHNSKGSLDFQLVITNIALIIIIITQTTSSLLYCIIRSKCLSHSYVFTVITSLCVLFSSFTMIIKYELIWNTVRSLKNTLVSNLDSFDLSEQEKVNSVYNFLSTYRDIKANVDLTIKGTRTTDCLHASARVSAIVLSVAVTCIAPILVEINVYEFGRIKFALADQLLEYTDSKLRRSVYDVVEYITVAPPTVTIWPEFTVDLGLYMDFIGLTVCYCIMILQFEY

>BmorGr38P

MKPKYVTNIKLVLFLRFLCGYYYEMEIPRRLKTVAKAYCIFFLFFYLVLHHLYCSFSNHTAKWSLYLEYSIYVFMSLYSKKMYLMDYYTSSRIIDFEPHSRIYKKLNIYLAILIPFLVVLKIVNMVTFCLSKSFNCWSWVSLLHNLLWNFTVLGRIPPVFVFALLFCRTRIIRRTLVSITVGPGSTVLKSLFKCTRYWLTVLKRPNTHLNSXLTVFLLCSTPKLILETFLMLNKIKESGPIVEKLAVYAIETFYTHLFFVVSSILFDLINVDLQRIKILIVEKRMKTKNTKHRIEVEKLFQFVKSQTIECTLWRVLSLNVRNILSFVSFAVTTIIAVLQIKNNNIY

>BmorGr39SI

MNVSPQYSVLKIFKPLFKVQTLFGSVRVKVGDNGITKTTKLQKFYSIFNILFATTGHFYTSFVYSVCVPCVGNSVAETSMALIQIYAGHLMNSFIVFSNTFLHNEKNVQMFKSLCSIDELMKIVRLEHRDLKLFIAIILLLSSTVIMNVYFLIYMVIILPISEKWVPFANIGIMNEDLEAITFVSVLYMLYDRVKYINQTALDPVNIAKLIKESDGQNDEETVSRILKAFKEISKAYKIVEKTFRVFNLSWGTFMSKNLIGVKFIARIMLISVCVSYLERELQKTKGLCNLAVRNCENDIVRCHLKNIYRIIDTEIEPMTVFGLFYINNVPLDLISLTATYTVVLLQFAFL

>BmorGr40SP

MLPICYIKLVYNISSIMFLRLIFGLYFELNVSNRMRWLQRMYCVFISIVIMYFCYFKTYRILMTVKVPFILEYGMHVILSLYLRENFEYTRYDPIIDSAADTKNFYKKFDIFIKIFLFVAILLKMZSMLVFCVLWSGLCYTGFIGICGMNFIWIATFMGRMVLPLIFGILLCRIRMFRLTLQKQGFDNLPYNRFSPRRYIMMYDSIVRGLEKTDFPAKNIMFVFTICIYSKILTGLFDLISVLKREGPKLMNVMLFTLEFLPSYVLLMIYSVTLDMVSTEMKEILKIVTEKRVFYEKQHANIQELCQYIKNNQLKYTIWRLVSLNMQSLLRATSFCIVSTIAILQIKDWNG

>BmorGr41SF

MSENSLEEYIHMSFSPIYKYQKFLGSNRISLKAKNKITVANNWEKLYAFLWMLAASYSIHHFISFFYSYYYERSNIIFLACSLGISMHYLTYILTITYDKFLTREADIDLFIDIQKIDRLLKLDRCTVLFKKFRLINIFLLILVTVPFISGFLIHVFDYIDKPYKTFFLGLGVTITYVDVLVTAFFITKLTLRLAYINDRIAMYNKINIPHKKYSGIRRSILWIFGWRIFKIMPKIKKNGTREKKSTFIKYPSIIFNILKCYRSITEIYSLPVFLITATVSIWTFLVIGSLVAGSRSEIKIFPVVAMITVGLWNFYYIIQLTSLAFVNDLFLMEVKNTKQLCISVLLYTCDDSINKAANTILKNIECVPPIFSVYGIFVFDKSIILFLFGIITSNVMTVIQFSY

>BmorGr42P

MNKTKKIERLSRDILDEDFIDVFKSIFIFQRIFGLLSVNITYKYITETSKLYKLFVMSLWTVNVLCLVDYILNYRTSFDVATDSMLKLVMSVNVTTNALIVWRNNFKLNTLKSQIYVKLQNLDRDLKTKDAVTMNKKLSALSIALMICGFIWCTIWLFVYNAIAMNTFCVPLTIILSANVGNWLEMVLLFIIFYFVNVRAEYVNKLLRRRLNQTECPDRVFLIQNAKPSDTVSREFICGMQSLLEIIGNIKDIYQFPIFLSTCQVMLCILVIVQNLIISVKEQTSTMVDSMLCMLPALLLMLTIFFSLCVIAEALTSKLDITKKLCAMGMHSFTDDISRRNSXQIVLLLKAKRPLSVFKIYTLGTRLPIHLLGVTASYTIVLLQFAVL

>BmorGr43JI

MLKKSAMITMKSPEYLSKDILDEDFVRVFRFPFLVQMVLGSCRVHLKARFITIPTLGQKLYTVMSIIICSLLYFNITKLYISLYYEQSIVYFLFLTVAGLDQLSFFANLIHVRFLNGETNTGFCIMMQRIDRKMKIDHNNIFNKTVIRANILTITLIILLYMSLVISTIILKKYSLVTLFGLVHGQLILLVEMAYCSNLIIFFFIRVRFVNAIIKNHVHPENQNQPPKLVRYFVTNRIMRYLAAQTHDFIVNDTDVYLKQIFEGFSMFIDIYRFQVCLFCIKLIVMSLLTFEFCLVGIQKNGPLRKKATRMLKIIEESTPQFSIYDMWQMDGYTFVKICSLVTNLIVTSLQFAYL

>BmorGr44P

IFLSTCQMMLCILVILQNLIISVKQZTSTIVDSMLCMLTALLIMLTIFFSLCVIAEALTSKLDMTKKLCAMSMHSFADDISRRSSKQIVLLLEAKRSMSVFNIYTLGTRLPIHLLGVTASYTIVLLQFAVL

>BmorGr45JR

MITMKSPEYLSKDILDEDFVRVFRFPFLVQMALGSCRVHLKARFITIPTLGQKLYTVMSIIICSLLYFNMTKLYLPLYYEHSIVYYIFVTVTGLDQLSFFANLIHLRFLNGETNTAFYIMMQRIDRNMKIDHNNIFNKTVTLANILTITLIILHYVGLVISTIILKEYSLLSLFGLLYGQLMLMVEMALCSNLIIFFFMRVRFVNAILKNHVHPENQNQPPKLVRYFVTNRITRYLAAQTHDFIVNDTDVYLKQIFEGFSMFIDIYRFQVCLFCIKLVVLSLLNFEFCLVGIQRNLLETKNLTNYYIMTYSVIGFFTALYVSGRCELFFREIRETKRLAVAVLLQYQEGPLREKATRMLKIIEESTPQFSIYDMWNMDGYIFIRICSLVTYLIVTLLQFAYL

>BmorGr46JR

MITMKSPEYLSKDILDEDFVRVFRFPFLVQMALGSCRVHLKARFITIPTLGQKLYTVMSIIICSLLYFNITKLYLPLYYQHSIVYYLFLAVTGLDQLSFFANLIHVRFLNGETNTAFCIMMQRIDRNMKIDHNNILNKTVIRANIFTITFIILIYVVLVISTIMLNEYSLVTLFGLLYGQLIFMVEMAHCSNLILFFFTRVRFVNAIIKNHVHPENQNQPPKLVRYFVTNRITRYLAAQTHDFIVNDTDVYLKQIFEGFSMFTDIYRFQVCLFCIKIVVLSLLTFELCFVAVQRNLLETKNLTNYYIMTYSVIGFFTALYVSGRCELFFREIRETKRLAVAVLLQYQEGPLREKATRMLKIIEESTPQFSVYDMWNMDGYIFIKICSLVTNLIVTLLQFAYL

>BmorGr47JR

MKIRFLFGFYCDFPFNKRFQNILKFYCISVLVVLILGSWACSTGFRSDKKIVIYCEYIAYFLISLSTKDRYIFDYYKQQPLIDGSTTSKVLYKKLERLLKYFVTITIVLKMLNIFVFCGWNLTKCINELDGVLFINLLWIGLLLARLSLPVIYGLLYFRLRVLRMTLESKGFSNSPQNRFTPKKYITIYEKIMKDLLKMDYPLKYVFIIFLIGSVPKLLQNSWQFLNSLKNYGPEISKILEFTLECLHSYIVIILPIVVALDLSEDEIKKMKIITLNKRLACLNERQKMEIQQLFLLLKNNSLRYNLWRVVPVNLKSVLIFLSFGVTNAIAIMQAKNLN

>BmorGr48F

MSGNYLEEYINMSFSPIYKYQKFLGSNRISLKAKNKITVANNWEKLYAFLWMLAASYSIHHFISFFYSYYYERSKILFLGCSLGTSSQYLTYILTITYDKFLTREADIDLFINLQKIDRLLKLDRCTVLFKKIRLIYIFLLILVTVPFISGFLIHVFDYIDQPYKTFFLGLGLTIIYVDVLVTAFFIANLTLRLAYINDRIAMYYKRSLPLRKDSGIRRSRSWICGSWIFQIMPKIKNNGTRMKNYTFIKYQSLIFNILKCYRLITEIYSLPVFLITATVSIWTFLVIGSIVAGSRSEIKLFSIVAMITVGLWNLFFIIQLTSLAFVNDLFLMEVKNTKQLCIRVLSYTRDDSINKAVNTILKDIEYAPPIFSVYGIFVFDKSIILFLIGIITGNIMTVIQFSY

>BmorGr49SJI

MAGIRTISFKVKPLELPDVSENNFADDGLKIVQRFKFFIYIQVITGINRLYLLKCNKFVMLFSYLYAIFLISFVASVYWTKEPMKNSHLVIRLFSFIEYILLICISVFLKKKKMMKFFENLSMFDQILKIDKNVNSTFCMKRVFFWVTGSIVYNLIEFYALEFYDNTSKGLMTIICTYTIAPAAIGVCVYLIVHCIKYTLLVVIPCYYSSITATQVSLIRITLHDAINTIPLGKLQRRKVKAFYLMTKEYSFVYTLAGVIKLNMSLPLSYISLCTTYLVIIIQFSKFLD

>BmorGr50S

MAGIRTISSKVKPLELPDVSENNFADDGLKIVQPFKFFIYIQAITGINRLYLLKCNKFVLMFSYLYAIFLISFVALVYWTTEPKKNSHLVIRLFTFFEYTLLACISVFLKKKKMIKFFENLSLLDKMLKINKNVNSTCCMKQVFFWVTGSIVYNLIEFYAMEFYDNTNKGLKTIICTYAIALAHDCEQIFFFTLQRVVYLRLLVVKRHIQEYFKVDEDSSRKKPNKYEMLSNNVQLNLTALHEVYALLHNCAEKLNTVMSIPVLLMLFTSGLSTTILLKFFVRVIQLTDPSNPGSAIGVCMYLIVRCIKYTLLVVISCYYSSITATQVSLIRITIHDAINTVPLGKLQRRKVKAFYLMTKEYSFVYALAGVIKLNMSLPLSYISLCTTYLVIIIQFSKFLD

>BmorGr51F

MAMGIRTILSKVKPLELPDVSENNFADDGLKIVQRFKFFIYIQVLTGINRLYLLKCNKFVMLFSYLYAIFLISFVASVYWTKEPMKNSHLVIRLFSFIEYILLICISVFLKKKKMMKFFENLSMFDQILKIDKNVNSTFCMKRVFFWVTGSIVYNLIEFYALEFYDNTSKGLMTIICTYTIALTHDCEQIFFFTLQRVVYLRLLVVKRHIQEHFKVDEDSNRKKPNKYEMLSKNVQLNLTALHEVYGLLHNCAEKLNKIMSIPVLLMLFTSGLTTTILLRILVRVIQLADPSNPGSAIGLCVYLIVRCIKYTLLVVISCYYSSITATQVSLIRITINDAINTIAFGKLQRRKVKAFYLMTKEYSFVYTLAGVIKLNMSLPLSYISLCTTYLVIIIQFSKFFD

>BmorGr52J

MAGIRTISSKVKPLELPDVSENNFADDGLKIVQRFKFFIYIQVITGINRLYLLKCNKFVMLFSYLYAMFLISFVVLVYWTTEAMKNSNLVIRNFTCLEYILLICIAMFLKKKKMIKFFENLSCLDKMLKIDKNVNSTCCMKRVSFWVAGSIVYNLIEFYAIEFYDNTNKGLVTIICTYTFALAHDCEQIFFFTLQRVVYLRLLVVKRHIQEYFKVDEDSSRKKPNKYEMLSNNVQLNLTALHEVYALLHNCAEKLNTVMSIPVLLILFTSGLSTTILLKILVRVIQFTDPSNPGSTIGVCVYLIVRCIKYTLLVVIPCYYSSITATQVSLIRITLHDAINTIPLGKLQRRKVKAFYLMTKEYSFVYDLGGVIKLNMSLPLSYISLCISYLVIIIQFSKFLD

>BmorGr53

MAHIKDENQSKQQQKEHETLNKNKLKKVVYTLKPALMLENWFGLSDFLLVNEDELVLLMQTEKFGVILSIFFIVMFAVFVDFPDTETESIMELMDEVPSMVVLSQYFIASITTSSCLSAIAIRIFETFADLDSMLLITTTQDFYNKSRYQTNKYLIILGVSHIISSTLDLLTDDEIVWCKFFVLPIYFLQKLEVLTFCKLIVMIQCRLQIINKYLTNFIEEQEKNKALVFTLAESNPKKTDKFNWIGCPSPNNMKIRDLATMYDVIGTICSLINDLFNIQIFMTLVSTFTYIVIAIWSTLYFYRAPNFTFGTLTTIIIWCITIILSVVVMSFVCERLVSVRNNTKILVNKVIMNYDLPKTMRVQAKAFMELIESWPLKIMVYDMFSVDISLMLKFISVATTYLIVIIQLSHFV

>BmorGr54S

MITIKNNFGFRQNNLSFYRPILIILQLCGYDFDYYNINLVLNVLTKAYCASLTCVVVYATIACCSSIQLSHIWSLIEYGTSVVIIACFRSQTKLFLKQLTTLDVYLRISNRRFVLEKCKIFTITSVIFLLRIVYTSIYCSTHHCFNVLIYFLLSQFALVCLDVNRIWRCIVFDAIRYRLKTLRLRMEENPDCNYYLYVKNNKSIRKNKISFCLFLYRTIADLVDLVSPELNVSLFLSVACSLPKIVSNAYHLLLIIEDREPLETGGYVLMHTLQVSLLLFTPFIIVECYTMEVEKIKLYLVHRLIDENGEDTTMRDNIRLFLEYMSVRTFRYRIFRIVPVNATLPLELVNLCVNYVIVLINFTHLYG

>BmorGr55

MERINLLKSFAFLENVMCIYRNFMFYNQRARFIIIGRIVAELVFYIFSAYNGFLLVYTDWFSQNFSVFFIEIISKSSFYVITFFTMVNGILKSREYKTFIFSINKIHDYILNDTDYLKRLKCTNIFCTATIIILFVVTLIRTAIDGSNYGQLSGINARSVIWMLTTILLECQYQTECVVYFGFILFIHAIMKYLNIRVTNTIIKIARSDMAVKRIPKYIIGRTELKDETDTGVDVNNVVDLEEVRYWVFIYRQLGLTTELLQKCFGMQTAFIFVTAVLNQIITVFRVIAVFIYGSLANRGAEHSIIANFLFTLLYRLPGLLMIIVGGQMVQNQTDMLRRSMARLNNIISNPHRETFSALSDFHRMIVKNPVKIYVLSVLPVGAYMLPLFMTLLINHIIILLQFNHVA

>BmorGr56

MKKIRLLRSIVFLENLLCIYRNFLFFNKKARAIILIHITIELVLYVLSIVNNSFIIYSYFHSDNRSMLIVFTTICCFYVVTFVSIVMGILRSEEFKDLVTSLELINKFFTNNKTYLKSLGRSNTMIIAITTILYCVTCIGIAVDKITLNDFYEFTSSDVIWTVSSTLLELRYQTECVVYFGIEYLFLIFTKHLNLLVKEAIKKVSLDNNGTVKDVPISSDAVTKNEVKRWATIYRQLMMSSKLLQACFSLQIICVFVSAVINFITTAFRMVKVSVLGSIATDMNEIIIVNLIFTLLYQNIGLVLIIVTGQRVWNQILLLNVLLARLYNGILIQPCRDTLRTLKNLQRMVVKNPVQIKMLSVLPVGSYMLPMFMTLSVSYIIVMLQFGHVV

>BmorGr57

MEEIKAIKLVTFIENCICVYRNYAMCTKRNKKIISLRIIVEIIIVFFVNINNILLLHKYYNGSGLLYIIYLFLVVYYINYMFCIFYGALQGKAYRQLIFCFNKINAIAKRDKSYKKSLARLKNMCIVISIALLIISALSVFVDRSNSWNIYEVSLRDSLLILSKIHMDFFYHFEYVVYFTHIKIFHLTLRYLNSRVKMAQFEMKMTRRDVHDEGERNIRILLTKELTTEWAVLYKCLVFGTKTMKSLFGLQMLIAMVMSFVNFTLSLYGIILICSIEQSQTASQHNLLLILTYYTATMLLIFIVAQSVYNEVEMLKRNLARMYNILAVDSDETQQKLVKDFLRMVYKNKVEIKMLSIFPVGMPMLTFFLSLSASYVVVMVQFSNVF

>BmorGr58

MSSRRVLYRAEVLLSNNVDAHVQDMLKPLNFFQFILFFPKYTIRDGYITPNSLIRNIWSATGAFVFISICVFRILTMNKIAVYDTFTTMLLISKYFDVALYCIGFIVNTYVNIAYSNVNVLLYLKLQTIKTFIPRNNEIMKNVKWYSVILIIVLFCGTLAMFSFFHLSFSYFNIFDLTTDLAVFSFDLNLVYACSVLNFLAQSLDELNKEIWRLGNAKVTVCKDGSKPDWNGINLTYINVLDAYNYFKEAFRLLIFFHTFKTLTHMFIYIQSIIELCKKFYPGDDYDAITVGAVVGVWFFRNITLQCLVGVSCQNFYSATSNTESICAVQVGSIVSDEHKLFLKAVRRLNNVVFYKWSMYGMFIVDATLPRRLIELIATYTVVFLQFAFK

>BmorGr59F

MPYKKDSNRCEVLLYNNVDTDLQDMLRPLNFIQTIYLSPKYTIKDGYITPNSLFCNILSAAGAIVFFSICVYRILTASKIGTFEGFSTTLLITKYFDAILFSLGFVANAYVSIRLSHLNVLLYLKLQAIKTFVPCKKIMQKVKYYSIVLIIGLIIVRLIMYIHFHWSLGYLSYLDLITDLGVISFDLNLVYASSIVKFLGYNLEELNKEILRLDEIKATMDEEGSKPDWNGIRRTYLKFSEAYNYFKDAFRILILFHTLNTFAHVFIYVQSVIELCKAPADNYSAAFSVLIAVIVWLLRNIILQSLIGISCQSFYSATSNTQSICSILVRSVLSDDQKLFLKTVQRLNKANISKLDVYGLFIVDATLPLRLIQVIATYTIVLLQFAFQ

>BmorGr60JR

MLTPRSDLCNEKLSPSFPSGKTTAADKDDTEARCQVDSSLERLLLPFNLVQHVSFIPMYSIRRGLVSPDGPLAYLYSLLGFCLFTSVSVYRNAIMHGTRLSSLHLFTLYSDLVSFVINYSLSLICNVVNSKSNVEFVCRLQRLQTVLRRNQREQEQFARSNWAHLAVVTALYLAVVGLLNVVVLKQSLPDTLYLLLLFCIDVNVLYATRMLALLRCYLQLWTRKINEKAFNPVHHNMFTAYLDILQEYEVYTTLFKKIITYYVLETFLHGLLYVQVAIQICKSIRRSGRFSEQLMMIVSIFTWTIKNMIIMTLHNVECEKFYLAVEQAVAACQTQRASTTRCREEKRLYKNVCRVSRAAFSRERGWGLLAAGAALTLRFMDLATTYVTVLLQFAFVSRT

>BmorGr61

MSIRFEKDLLHNYVEIELQYFLRPFNVMQSLFFQSKYRIVDNFILPNTLFKNIMSFVVSVLCALSFIYTIISVWQNTHATSFHALVTSVYLSYNIYGILIGSVLIIWLSDRNIEFVLKIQDLIKILEFNKCFLIEYAFINSIIMAAIFILNFLLYGYFVVHLQKFALGLTFSAIVCILNQDLDIIYVIIFANILKKCASRWTVEARQKNNFNDQGKWVKLFNAFLNLTESYQLYQKIFEFYELLRRVGIVFLGLQLTVCRVCSNDIKSIQCTVMLHAFQLICVWIVKKFITLSILSFEMEIFYEKLREIETVCIILVSSDNPSERELKIWKNIIRVSSCSVRKTTACGLCEVGAALPQWLLQATTAYTIVLLQFHITTFSRARNDIYDLD

>BmorGr62

MNDLFLSKIVKWTKTTKYKLDDDFQSLFRVFNIAQAMNLCPKFLIYDKYITNNAWFIHILAISSFIVLVCLDSFFANFRLVLSEAMGPPFYGFSFYFISILYENIGVIIQITMNGYLTKNNVLIITKLQDTFKDFRTTDYITKSNRWTNWFIFFIYMNFIANYSYFNFYVNTFSFHKFCFAFIKMCFDLNIVYTIFIFKMIGDSLTMFKDTAFCSKNMKLYEVSNRVYWNKMLRLYSNILDVFELSKRTLNFFIFYFVSNILLRILSHVQLAILMNSINWLQHVAYSNIVMVLLTLAKEGIILIVLIAKCEKIYCVIGDVQTACQLALGNAACPEKRRFCKNVRRSSSAAFSKIYICNILAVDAKLAVSLMSVTTTYTIVMLQAILIK

>BmorGr63F

MQIGNAVIHLKSTKLTTMNTISPTTKLLKIFALNSNIEEIDLKCSTKLRITMTAFVLCSLIFYSLYYKFIYVFDYVNISIKITDCVQMVYDFCQYIVDLYFVTNYGRNISSEYFQQYKIIDKILEVVCYEIIKHRIVKLLWVFMCIWFSSSCFDFIAWFLNYGWITPLVYSVAYIFLLIKILTTLDLSAHIMNVEIRLKMIADLIHHYYMSCEDNFQAEETLCHKNWLNSKERAKYYELQFRIHALKQLSCNNNEIKLLSRCYLMLTEQVEIINRMYGFRILLNSLSLLIDMVRFTNISVRIMIGSQNLAYNCGYFPAVSSIFRLLTCGAVIINLVSHCERVYYQRTRICNVIDHMIVNKNLSRESTEALQEFRNLVQNHPIEFNMANFFQLNYSLLVSIASVVVTYTIILLQSVN

>BmorGr64F

MKISLRKIVSIRNMTLIQNMFGFYHKFTDNRAIGVLLKIFCGFYSLFLSFLCINCTPRFTNDFLTYDIFFFVIEYLTSVLVCLLYDGQYFLNYLYDLKLIDREAGIEESLEKLPISQPLFSLIFITRVIYLLSCLLMFDGIKDSLFLPAQSSVFGANFTEFARTIGYFPRVIMFEMFYKRVNYLKSQLRNDLAHANLYPIGFVCSKVIMKYINFYKLLLRNLQQNSLQFKILMSMSSLYIIIKALASAYAFIYREDGVHVFIFIEFATGVFLFFVMSSIIISIFNEIEDIRQIVLAQLRYCKQGANTKRVQDALTILNIRCFKYALCRIYTVDFTFILRILDVSVTYVIVLVQFTHILD

>BmorGr65JI

MKISLRKIVSIRNMTLIQNMFGFYHKFTDNRAIGVLLKIFCGFYSLFLSFLCINCTPRFTNDFLTYDIVVFVIEYLTSVLVCLLYDGQYFFNYLYDLKLMDREAGFEESCSQKLPISQPLFCFVFITRVIYLFLCLLMYDGINDTLFLHGQISVFGVHVFIFIEFAIGVFLFFVMSSIIISIFNEIEDIRQIVLAQLRYCKQGANTKRVQDALTILNIRSFKYALCRIYTVDFTFILRILDVSVTYVIVLVQFTHVLD

>EoblGR3

MGQKFVKKNLALWIPTKQNKVHAKKVKTKSVATFKKSLRATLLIGQLFSLMPVVGLLSNSANNVKFVRTSWKCVYTFLSLIGQIFMTIMCIHRASRQQTTLNGTTPIIFYCTTCITMLMFFRVARQWPALVHAISRTEDLDPIYDSNLTLKCNVTCGIVLFLALMEHILSLMSAFAGAEICYPTKSTYEGFVMHFYPWVFGTLPYSPLLGGLTQFLHFQSTFIWNFSDLFVICMSYYLTSRLDQVNKKLLVAQGKYFPEVFWSSVREEYGRATQIVRKVD

SVISGIVFISFANNLFFICLQLFNTLEDGIKGTGECSSRSKKTTLLHGYEAAAYFLFSLVYLITRSVAVSLIASQVNTASEVPAPVLYDVPSPAYCIEVQRFLDQVNGEKVALSGLQFFSVTRSLLLTVAGTIVTYELVMFQFNSS

>EoblGR2

MGVEPGKVEVSSEPVRAPAHALDGAHCVVGGAHAFILRVSSLFGLAPLKFESKTNGYTVSISNAMCIYSFVLVTVLVLCTIFGLVAEINVGVELSVRMTSRMSQVVSACDVMVVVVTAGAGVYGAPHRMRNMLDFMGRVAAVDNSLGGRYSKVTERRLCALLLCILIFFSVLLADDFCFYAMQAKRTDRQWNVVMNYIGFYLLWYVVMILELQFAFTALSVRGRFLAVNDALALTARQVSVPLEKPKISSPLNIYAIRVAPAADPQRPANVSLLIDSGTREQPVVVRKSAHGSATLAVAPCEAVRRLASLHGTLCEVVRCVDASYGLPLIVVLISTLLHLIVTPYFLIMEIIVSTNRVHFLVLQFLWCLTHMLRMLVVVEPCHYTIAEGRRTQNLVCRLMNNVTDRPALGTLPSRVELFSRQLLLQSVSYTPLGMCTLDRPLVASVIGAVTTYLVILIQFQRYDN

>EoblGR1

MFDNVSDMDGLYQANGTIFYPQEATILAAVCACIFSVVGVVGNSVTTVALLMHPKLRGHVTTMFVLSLCVSDLLFCSINLPLTAYRYIEQHWSLGDRLCQMFAFVFYGNEAVSLLSMVAITINRYTLIAYYDMYSQIYTTTKIWIQLLLIWLVSFGLMVPSLLGVWGQLGLDERTFSCTILKKDGRSPKKYLFVFGFVLPCVVIIVSYSCIYWKVRESKKKLEGRSKLSGQTAKEKEEDSRLTTLMATIFLCFLACFLPLMIMNVADDGITFPWLHVIASILAWASSVINPLIYAATNRQYRAAYGNLLRICKNNPVTRRTTWGSRTMGQSSNSPHYPEKRSPKDKPTNL

>HarmGR1

MGQTSFRRNMSFWIPVKKNKVDVAKPKVKNITSFQDALRVTVIIGQVFSLLPFVGVFTNVASNVKFVKTSWKCVYSLLSLIGQMFMAVLCINKLAKTTVSLNGTSPVIFYVTTCVTMMLFFQVARRWPALVQHISKAEDMDPNFDCSLTRKCNITCAVVLILALCEHILSLLSAFAGASACYSGMDTYEGFVTHFYPWVFSYLPYSIVLGVITQFLHFQSTFIWNFSDLFVICMSYYLTSRLEQVNRKLLAAQGKYLPEIFWRATREDYCRATQIVRKVDEVISGVVFISFANNLFFICLQLFNTLEDGLKGTGECTPKLKKIVVSKSGPLGGHEAAAYFLFSLVYLLSRSVAVSLIASQVNSASSVPAPVLYDVPSPVYCVEVQRFLDQVNGDKVALSGLQFFSVTRGLLLTVAGTIVTYELVMFQFNSSTPTLNITSPTVVTHTITTLAT

>HarmGR4

MLWIETHHYLGVESAKVEEVTAAPVPSESGSRPSRPTHCVVGGTHAFILRISSFFGLAPLRFESRSNGFTVSISGAMCVYSYILVTVLVICTIFGLVAEINVGVELSVRMSSRTSQVVSTCDVLVVVATAGAGVYGAPRRMRNMLKFMENIASVDTSIGGQYSLVTERKLCGIILAILIFFSILIADDFTFYALQAKKLDREWDVVTNYLGFYLLWFVVLILELQFAFTALSVRARFSAVNDALALTARQVSIPVEKPKSSSPLNIYAIRVAPVDSQHSANVSLLVDTMTGREHVVIIKRTASGEPRLVVSPCDAVRRLAALHGTLCDVVNSIDDSYGLPLVVILISTLLHLIVTPYFLIMEIIVSTNRIHFLVLQFLWCVTHMLRMIVVVEPGHYTIAEGKRTEGLVCRLMTSAPSTGVLPSRLEIFSRQLMLQSVSYAPMGMCTLHRPLIASVIGAVTTYLVILIQFQRYDN

>HarmGR5

MQNGWNNVISNISVGSVNTVNYLFRTWERLAPNRNMDLYSLEKFKKYKNDWNYPVHVRYQDQVMAEKEKPRTTFQTAMKVTLTIGQCFGLNPVQGIREKDASKLRFKLLSGRCLFTFFSLIGQFIMAFVLFLSLFKETSSTVDTATALIFYSFGFTTTILFFRIATNWPKLCMHIAKVESVDPNTDNKLGKKFNIACISILFLALMEHLFSELHGISIALDCFPDTPVYESFMKLSFQWLFGFIPYSDFAGGMAHFSNLQCTFNWNFADVFVICMSMYLTARLEQVNQRIIAAKDKNSPSSFWRTMREDYNRSVHLVRQVDKIIGGVVFMSFASNLFFVCSQLLHTLAGGIKASQRCKPEIGADRRFFYGYEHSIYFVFSFSFLVIRSLAVSLTASKVHAASLEPAYSLYDVSSANYCVEVERFLDQIHGDTVALSGLQFFHVKRGLVLTIAGTIVTYELVLMQFTGITPTTSPESVSGVIK

>HarmGR3

MSFHTSNSLFPMTVPIPNGFPVQINSKPKNKIIFLDVTPVSTPIKPHSPNVVVPMRNNLVAPHISNDIIYENIKPVFTLLRIMGVLPITRPSACVNQFQIASSSMLYAILVFLSLVSYVLYLSLHKVQILRTAEGKFEEAVIEYLFTVYLFPMIAVPLLWYETRKIANVLNGWVDFEMVYKQLSGRTLPVKLYKKALAMAVIIPILSTTTVIVTHVTMVHFKPMQLVPYVFLEILTYMLGGYWYLLCETLSICANILAEDFQNALRHIGPAGKVAEYRALWLRLSKLSRDTGIANCYTFTFVNLYLFLIITLSIYGLLSQISDGFGIKDIGLALTAFCSISLLFFICDEAHYASHNVRTNFQKKLLMVELSWMNTDAQTEVNMFLRATEMNPSQISLGGFFNVNRTLFKSLLATMVTYLVVLLQFQISIPDESQNRDEEEEVPYNITSATTEAMTTSTTTIMTTVLTTLAKKKKKN

>HarmGR2

MTIPDHLFDEGINNTLLQHDMRHVQQNRIVYEKTQREYEQEQRDMLSSQDGDTCEIHDQFYRDHKLLLVLFRALAVMPITRSRPGTITFSWRSTATMYAVCFYIAATAVVMIVGYERIMILRSIRRFDEYIYAILFVIFLVPHFWIPFVGWGVAHQVAIYKTNWGKFQVRYYRVTGENLKFPNLKTTIVMISVGCLLLAVCFLLSLCILMDGFLLRHTTAYYHIITMINMNCALWYINCKGIKIASQSLSECFRRDVEAECSAKLISRYRYLWLNLSELLQSLGNAYARTYSTYCLFMFANITIAVYGALSEIVDHGFGFSFKEMGLFVDAAYCSTLLFIFVDCSHNSTLTVAAGVQETLLSIDVLSVDRPTQKEIDHFIQAIEMNPAVVSLKGYAHVNRELLTSAISMIAIYLIVLLQFKISLPRDPQIVAT

>HarmGR9

MGVESAKVEEVTAAPVPSESGARPSRPTHCVVGGAHAFILRISSFFGLAPLRFESRSNGFTVSISGAMCVYSYILVTVLVICTIFGLVAEINVGVELSVRMSSRMSQVVSTCDVLVVVATAGAGVYGAPRRMRNMLKFMENIASVDTSIGGQYSLVTERKLCGIILAILIFFSILIADDFTFYALQAKKLDREWDVVTNYLGFYLLWFVVLILELQFAFTALSVRARFSAVNDALALTARQVSIPVEKPKSSSPLNIYAIRVAPVDSQRSANVSLLVDTMTGREHVVIIKRTASGEPRLVVSPCDAVRRLAALHGTLCDVVNSIDDSYGLPLVVILISTLLHLIVTPYFLIMEIIVSTNRIHFLVLQFLWCVTHMLRMIVVVEPGHYTIAEGKRTEGLVCRLMTSAPSTGVLPSRLEIFSRQLMLQSVSYAPMGMCTLHRPLIASVIGAVTTYLVILIQFQRYDN

>DmelGr2a

MEFGMDTLRALEPLHRACQVCNLWPWRLAPPPDSEGILLRRSRWLELYGWTVLIAATSFTVYGLFQESSVEEKQDSESTISSIGHTVDFIQLVGMRVAHLAALLEALWQRQAQRGFFAELGEIDRLLSKALRVDVEAMRINMRRQTSRRAVWILWGYAVSQLLILGAKLLSRGDRFPIYWISYLLPLLVCGLRYFQIFNATQLVRQRLDVLLVALQQLQLHQKGPAVDTVLEEQEDLEEAAMDRLIAVRLVYQRVWALVALLNRCYGLSMLMQVGNDFLAITSNCYWMFLNFRQSAASPFDILQIVASGVWSAPHLGNVLVLSLLCDRTAQCASRLALCLHQVSVDLRNESHNALITQFSLQLLHQRLHFSAAGFFNVDCTLLYTIVGATTTYLIILIQFHMSESTIGSDSNGQ

>DmelGr5a

MRQLKGRNRCNRAVRHLKVQGKMWLKNLKSGLEQIRESQVRGTRKNFLHDGSFHEAVAPVLAVAQCFCLMPVCGISAPTYRGLSFNRRSWRFWYSSLYLCSTSVDLAFSIRRVAHSVLDVRSVEPIVFHVSILIASWQFLNLAQLWPGLMRHWAAVERRLPGYTCCLQRARPARRLKLVAFVLLVVSLMEHLLSIISVVYYDFCPRRSDPVESYLLGASAQLFEVFPYSNWLAWLGKIQNVLLTFGWSYMDIFLMMLGMGLSEMLARLNRSLEQQVRQPMPEAYWTWSRTLYRSIVELIREVDDAVSGIMLISFGSNLYFICLQLLKSINTMPSSAHAVYFYFSLLFLLSRSTAVLLFVSAINDQAREPLRLLRLVPLKGYHPEVFRFAAELASDQVALTGLKFFNVTRKLFLAMAGTVATYELVLIQFHEDKKTWDCSPFNLD

>DmelGr8a

MSGHLGRVLQFHLRLYQVLGFHGLPLPGDGNPARTRRRLMAWSLFLLISLSALVLACLFSGEEFLYRGDMFGCANDALKYVFAELGVLAIYLETLSSQRHLANFWWLHFKLGGQKTGLVSLRSEFQQFCRYLIFLYAMMAAEVAIHLGLWQFQALTQHMLLFWSTYEPLVWLTYLRNLQFVLHLELLREQLTGLEREMGLLAEYSRFASETGRSFPGFESFLRRRLVQKQRIYSHVYDMLKCFQGAFNFSILAVLLTINIRIAVDCYFMYYSIYNNVINNDYYLIVPALLEIPAFIYASQSCMVVVPRIAHQLHNIVTDSGCCSCPDLSLQIQNFSLQLLHQPIRIDCLGLTILDCSLLTRMACSVGTYMIYSIQFIPKFSNTYM

>DmelGr9a

MSLWLEHFLTGYFQLCGLVCGWSGSRLGRLLSSTFLVLILIELVGEIETYFTEENPDNESVPAYFAKVIMGVNMAYKMIHAWIALSALFECRRFRYLLEELPPVKATSFIYRHLILEIILFACNAFLVLSEYTIRGIYLENLRYAYSLQAVRARYLQMMVLVDRLDGKLEQLHHRVISGSSDYKTLRLDYAHLAKVTRSLSHLFGLSLLLLNVLCLGDWIIVCNVYFMVAYLQVLPATLFLFGQVMFVVCPTLIKIWSICAASHRCVSKSKHLQQQLKDLPGQTPVERSQIEGFALQIMQDPIQIDVCGIYHLNLQTLAGMFFFILEALVIFLQFVSLVRT

>DmelGr10a

MTSPDERKSFWERHEFKFYRYGHVYALIYGQVVIDYVPQRALKRGVKVLLIAYGHLFSMLLIVVLPGYFCYHFRTLTDTLDRRLQLLFYVSFTNTAIKYATVIVTYVANTVHFEAINQRCTMQRTHLEFEFKNAPQEPKRPFEFFMYFKFCLINLMMMIQVCGIFAQYGEVGKGSVSQVRVHFAIYAFVLWNYTENMADYCYFINGSVLKYYRQFNLQLGSLRDEMDGLRPGGMLLHHCCELSDRLEELRRRCREIHDLQRESFRMHQFQLIGLMLSTLINNLTNFYTLFHMLAKQSLEEVSYPVVVGSVYATGFYIDTYIVALINEHIKLELEAVALTMRRFAEPREMDERLTREIEHLSLELLNYQPPMLCGLLHLDRRLVYLIAVTAFSYFITLVQFDLYLRKKS

>DmelGr10b

MRVGKLCRLALRFWMGLILVLGFSSHYYNPTRRRLVYSRILQTYDWLLMVINLGAFYLYYRYAMTYFLEGMFRRQGFVNQVSTCNVFQQLLMAVTGTWLHFLFERHVCQTYNELSRILKHDLKLKEHSRFYCLAFLAKVYNFFHNFNFALSAIMHWGLRPFNVWDLLANLYFVYNSLARDAILVAYVLLLLNLSEALRLNGQQEHDTYSDLMKQLRRRERLLRIGRRVHRMFAWLVAIALIYLVFFNTATIYLGYTMFIQKHDALGLRGRGLKMLLTVVSFLVILWDVVLLQVICEKLLAEENKICDCPEDVASSRTTYRQWEMSALRRAITRSSPENNVLGMFRMDMRCAFALISCSLSYGIIIIQIGYIPG

>DmelGr21aNEW

MSFWAVSRGLTPPSKVVPMLNPNQRQFLEDEVRYREKLKLMARGDAMEEVYVRKQETVDDPLELDKHDSFYQTTKSLLVLFQIMGVMPIHRNPPEKNLPRTGYSWGSKQVMWAIFIYSCQTTIVVLVLRERVKKFVTSPDKRFDEAIYNVIFISLLFTNFLLPVASWRHGPQVAIFKNMWTNYQYKFFKTTGSPIVFPNLYPLTWSLCVFSWLLSIAINLSQYFLQPDFRLWYTFAYYPIIAMLNCFCSLWYINCNAFGTASRALSDALQTTIRGEKPAQKLTEYRHLWVDLSHMMQQLGRAYSNMYGMYCLVIFFTTIIATYGSISEIIDHGATYKEVGLFVIVFYCMGLLYIICNEAHYASRKVGLDFQTKLLNINLTAVDAATQKEVEMLLVAINKNPPIMNLDGYANINRELITTNISFMATYLVVLLQFKITEQRRIGQQQA

>DmelGr22a

MSQPKRIHRICKGLARFTIRATLYGSWVLGLFPFTFDSRKRRLNRSKWLLAYGLVLNLTLLVLSMLPSTDDHNSVKVEVFQRNPLVKQVEELVEVISLITTLVTHLRTFSRSSELVEILNELLVLDKNHFSKLMLSECHTFNRYVIEKGLVIILEIGSSLVLYFGIPNSKIVVYEAVCIYIVQLEVLMVVMHFHLAVIYIYRYLWIINGQLLDMASRLRRGDSVDPDRIQLLLWLYSRLLDLNHRLTAIYDIQVTLFMATLFSVNIIVGHVLVICWINITRFSLLVIFLLFPQALIINFWDLWQGIAFCDLAESTGKKTSMILKLFNDMENMDQETERRVAEFTLFCSHRRLKVCHLGLLDINYEMGFRMIITNILYVVFLVQFDYMNLKFKTD

>DmelGr22b

MFGSSREIRPYLARQMLKTTLYGSWLLGIFPFTLDSGKRIRQLRRSRCLTLYGLVLNYFLIFTLIRLAFEYRKHKLEAFKRNPVLEMINVVIGIINVLSALIVHFMNFWGSRKVGEICNELLILEYQDFEGLNGRNCPNFNCFVIQKCLTILGQLLSFFTLNFALPGLEFHICLVLLSCLMEFSLNLNIMHYHVGVLLIYRYVWLINEQLKDLVSQLKLNPETDFSRIHQFLSLYKRLLELNRKLVIAYEYQMTLFIIAQLSGNIVVIYFLIVYGLSMRTYSIFLVAFPNSLLINIWDFWLCIAACDLTEKAGDETAIILKIFSDLEHRDDKLEMSVNEFAWLCSHRKFRFQLCGLFSMNCRMGFKMIITTFLYLVYLVQFDYMNL

>DmelGr22cNEW

MFASRSDLQSRLCWIILKATLYSSWFLGVFPYRFDSRNGQLKRSRFLLFYGLILNFFLLLKMVCSGGQKLGIPEAFARNSVLENTHYTTGMLAVFSCVVIHFLNFWGSTRVQDLANELLVLEYQQFASLNETKCPKFNSFVIQKWLSVIGLLLSYLSIAYGLPGNNFSVEMVLINSLVQFSFNCNIMHYYIGVLLIYRYLWLINGQLLEMVTNLKLDCSVDSSRIRKYLSLYRRLLELKGYMVATYEYHMTLVLTTGLASNFLAIYSWIVLDISMNINFIYLLIFPLFLLVNVWNLWLSIAASDLAENAGKSTQTVLKLFADLEVKDIELERSVNEFALLCGHCQFNFHVCGLFTINYKMGFQMIITSFLYLIYMIQFDFMNL

>DmelGr22d

MFRPRCGLRQKFVYVILKSILYSSWLLGIFPFKYEPKKRRLRRSMWLIPFGVVISSSLLILMVKQSAEDREHGIMLDVFQRNALLYQISSLMGVVGVVSICTVHLRTLWRSKHLEEIYNGLMLLEAKYFCSNAVECPAFDGYVIQKGVVIVVGLLAPWMVHFGMPDSKLPVLNVLVVSMVKLGTLLLALHYHLGVVIIYRFVWLINRELLSLVCSLRGNHKGSSSRVRFLLKLYNKLVNLYSKLADCYDCQTVLMMAIFLAANIIVCFYMIVYRISLSKMSFFVMLIMFPLAIANNFMDFWLSMKVCDLLQKTGRQTSMILKLFNDIENMDKDLEISISDFALYCSHRRFKFLHCGLFHVNREMGFKMFVASVLYLLYLVQFDYMNL

>DmelGr22e

MFRPSGSGYRQKWTGLTLKGALYGSWILGVFPFAYDSWTRTLRRSKWLIAYGFVLNAAFILLVVTNDTESETPLRMEVFHRNALAEQINGIHDIQSLSMVSIMLLRSFWKSGDIERTLNELEDLQHRYFRNYSLEECISFDRFVLYKGFSVVLELVSMLVLELGMSPNYSAQFFIGLGSLCLMLLAVLLGASHFHLAVVFVYRYVWIVNRELLKLVNKMAIGETVESERMDLLLYLYHRLLDLGQRLASIYDYQMVMVMVSFLIANVLGIYFFIIYSISLNKSLDFKILVFVQALVINMLDFWLNVEICELAERTGRQTSTILKLFNDIENIDEKLERSITDFALFCSHRRLRFHHCGLFYVNYEMGFRMAITSFLYLLFLIQFDYWNL

>DmelGr22f

MKMFQPRRGFSCHLAWFMLQTTLYASWLLGLFPFTFDSRRKQLKRSRWLLLYGFVLHSLAMCLAMSSHLASKQRRKYNAFERNPLLEKIYMQFQVTTFFTISVLLLMNVWKSNTVRKIANELLTLEGQVKDLLTLKNCPNFNCFVIKKHVAAIGQFVISIYFCLCQENSYPKILKILCCLPSVGLQLIIMHFHTEIILVYRYVWLVNETLEDSHHLSSSRIHALASLYDRLLKLSELVVACNDLQLILMLIIYLIGNTVQIFFLIVLGVSMNKRYIYLVASPQLIINFWDFWLNIVVCDLAGKCGDQTSKVLKLFTDLEHDDEELERSLNEFAWLCTHRKFRFQLCGLFSINHNMGFQMIITSFLYLVYLLQFDFMNL

>DmelGr23aA

MKTLECLTRRFLEVIFSVLALVPLPPISQLGWLFLSLAIRCCWIVYFIYLLDVAISFSWVAIENVGNAVGTMLFVGNSVLGFALLLESVLKQKTHSQLEDLRVQTELQLQRLGMFGRSRHAAYLLPLIGVQFTCDLVRLATNFGETVSPVFCISLPLMWLLRYRYVQLVQHVMDLNQRSIHLRRSLLSMASGNDLWQPYGVQECLQLQTLRTTYERIFECYETFSDCYGWGMLGLHLLTSFQFVTNAYWMIMGIYDGGNVRSLIFNGATGIDFGTPIATLFWHGDSGAENGRQIGCLISKLVKPQGSKLYNDLVSEFSLQTLHQRFVVTAKDFFSLNLHLLSSMFAAVVTYLVILIQFMFAERSSTRGSG

>DmelGr23aB

MFPPTRVQASSRVVLKIFHFILVAFSLRSRRLSRLVLWLQFLGWLTWFISMWTQSVIYAQTIDCTLDCSLRHILTFFQTVSHAFIVVTSFLDGFRIKQDQLDEPIAFEDSDPWLAFTVLAMLVPTLGVEYLVCSNAPEYAFRIRIYHLKTLPSFLALQVQIISFILEVMKVNIRVRQTKLQLLILARELSCRWPQRKQKPQFSDQQAHRVKDLKRRYNDLHYLFVRINGYFGGSLLTIIIVHFAIFVSNSYWLFVDIRTRPWRIYAILLNLGFIFNVALQMAAACWHCQQSYNLGRQIGCLISKLVKPQGSKLYNDLVSEFSLQTLHQRFVVTAKDFFSLNLHLLSSMFAAVVTYLVILIQFMFAERSSTRGSG

>DmelGr28a

MAFKLWERFSQADNVFQALRPLTFISLLGLAPFRLNLNPRKEVQTSKFSFFAGIVHFLFFVLCFGISVKEGDSIIGYFFQTNITRFSDGTLRLTGILAMSTIFGFAMFKRQRLVSIIQNNIVVDEIFVRLGMKLDYRRILLSSFLISLGMLLFNVIYLCVSYSLLVSATISPSFVTFTTFALPHINISLMVFKFLCTTDLARSRFSMLNEILQDILDAHIEQLSALELSPMHSVVNHRRYSHRLRNLISTPMKRYSVTSVIRLNPEYAIKQVSNIHNLLCDICQTIEEYFTYPLLGIIAISFLFILFDDFYILEAILNPKRLDVFEADEFFAFFLMQLIWYIVIIVLIVEGSSRTILHSSYTAAIVHKILNITDDPELRDRLFRLSLQLSHRKVLFTAAGLFRLDRTLIFTITGAATCYLIILIQFRFTHHMDDTSSNSTNNLHSIHLGD

>DmelGr28bA

MIRCGLDIFRGCRGRFRYWLSARDCYDSISLMVAIAFALGITPFLVRRNALGENSLEQSWYGFLNAIFRWLLLAYCYSYINLRNESLIGYFMRNHVSQISTRVHDVGGIIAAVFTFILPLLLRKYFLKSVKNMVQVDTQLERLRSPVNFNTVVGQVVLVILAVVLLDTVLLTTGLVCLAKMEVYASWQLTFIFVYELLAISITICMFCLMTRTVQRRITCLHKVLKNLAHQWDTRSLKAVNQKQRSLQCLDSFSMYTIVTKDPAEIIQESMEIHHLICEAAATANKYFTYQLLTIISIAFLIIVFDAYYVLETLLGKSKRESKFKTVEFVTFFSCQMILYLIAIISIVEGSNRAIKKSEKTGGIVHSLLNKTKSAEVKEKLQQFSMQLMHLKINFTAAGLFNIDRTLYFTISGALTTYLIILLQFTSNSPNNGYGNGSSCCETFNNMTNHTL

>DmelGr28bB

MSALRRVRKYFISSQVYEALRPLFFLTFLYGLTPFHVVRRKMGESYLKMSCFGVFNIFIYICLCGFCYISSLRQGESIVGYFFRTEISTIGDRLQIFNGLIAGAVIYTSAILKRCKLLGTLTILHSLDTNFSNIGVRVKYSRIFRYSLLVLIFKLLILGVYFVGVFRLLVSLDVTPSFCVCMTFFLQHSVVSIAICLFCVIAFSFERRLSIINQVLKNLAHQWDTRSLKAVNQKQRSLQCLDSFSMYTIVTKDPAEIIQESMEIHHLICEAAATANKYFTYQLLTIISIAFLIIVFDAYYVLETLLGKSKRESKFKTVEFVTFFSCQMILYLIAIISIVEGSNRAIKKSEKTGGIVHSLLNKTKSAEVKEKLQQFSMQLMHLKINFTAAGLFNIDRTLYFTISGALTTYLIILLQFTSNSPNNGYGNGSSCCETFNNMTNHTL

>DmelGr28bC

MDIEMAKEPVNPTDTPDIEVTPGLCQPLRRRFRRFVTAKQLYECLRPVFHVTYIHGLTSFYISCDTKTGKKAIKKTIFGYINGIMHIAMFVFAYSLTIYNNCESVASYFFRSRITYFGDLMQIVSGFIGVTVIYLTAFVPNHRLERCLQKFHTMDVQLQTVGVKIMYSKVLRFSYMVLISMFLVNVLFTGGTFSVLYSSEVAPTMALHFTFLIQHTVIAIAIALFSCFTYLVEMRLVMVNKVLKNLAHQWDTRSLKAVNQKQRSLQCLDSFSMYTIVTKDPAEIIQESMEIHHLICEAAATANKYFTYQLLTIISIAFLIIVFDAYYVLETLLGKSKRESKFKTVEFVTFFSCQMILYLIAIISIVEGSNRAIKKSEKTGGIVHSLLNKTKSAEVKEKLQQFSMQLMHLKINFTAAGLFNIDRTLYFTISGALTTYLIILLQFTSNSPNNGYGNGSSCCETFNNMTNHTL

>DmelGr28bD

MSFYFCEIFKPRDAFGAEQTLLLYTYLLGLTPFRLRGQAGERQFHLSKIGYLNAFLQLSFFSYCFLAALIEQQSIVGYFFKSEISQMGDSLQKFIGMTGMSILFLCSSIRVRLLIHIWDRISYIDDRFLNLGVCFNYPAIMRLRLLQIFLINGVQLGYLISSNWMLLGNDVRPIYTAIVAFYVPQIFLLSIVMLFNATLHRLWQHFTVLNQVLKNLAHQWDTRSLKAVNQKQRSLQCLDSFSMYTIVTKDPAEIIQESMEIHHLICEAAATANKYFTYQLLTIISIAFLIIVFDAYYVLETLLGKSKRESKFKTVEFVTFFSCQMILYLIAIISIVEGSNRAIKKSEKTGGIVHSLLNKTKSAEVKEKLQQFSMQLMHLKINFTAAGLFNIDRTLYFTISGALTTYLIILLQFTSNSPNNGYGNGSSCCETFNNMTNHTL

>DmelGr28bE

MWLLRRSVGKSGNRPHDVYTCYRLTIFMALCLGIVPYYVSISSEGRGKLTSSYIGYINIIIRMAIYMVNSFYGAVNRDTLMSNFFLTDISNVIDALQKINGMLGIFAILLISLLNRKELLKLLATFDRLETEAFPRVGVAMHQVAANKKMNRLVIILVGSMVAYITCSFLMISLRDTTTFSISAVISFFSPHFIVCAVSFLAGNVMIKLRIYLSALNEVLKNLAHQWDTRSLKAVNQKQRSLQCLDSFSMYTIVTKDPAEIIQESMEIHHLICEAAATANKYFTYQLLTIISIAFLIIVFDAYYVLETLLGKSKRESKFKTVEFVTFFSCQMILYLIAIISIVEGSNRAIKKSEKTGGIVHSLLNKTKSAEVKEKLQQFSMQLMHLKINFTAAGLFNIDRTLYFTISGALTTYLIILLQFTSNSPNNGYGNGSSCCETFNNMTNHTL

>DmelGr32aNEW

MSPNTWVIEMPTQKTRSHPYPRRISPYRPPVLNRDAFSRDAPPMPARNHDHPVFEDIRTILSVLKASGLMPIYEQVSDYEVGPPTKTNEFYSFFVRGVVHALTIFNVYSLFTPISAQLFFSYRETDNVNQWIELLLCILTYTLTVFVCAHNTTSMLRIMNEILQLDEEVRRQFGANLSQNFGFLVKFLVGITACQAYIIVLKIYAVQGEITPTSYILLAFYGIQNGLTATYIVFASALLRIVYIRFHFINQLLNGYTYGQQHRRKEGGARARRQRGDVNPNVNPALMEHFPEDSLFIYRMHNKLLRIYKGINDCCNLILVSFLGYSFYTVTTNCYNLFVQITGKGMVSPNILQWCFAWLCLHVSLLALLSRSCGLTTTEANATSQILARVYAKSKEYQNIIDKFLTKSIKQEVQFTAYGFFAIDNSTLFKIFSAVTTYLVILIQFKQLEDSKVEDPVPEQT

>DmelGr33aNEW

MIQIMNWFSMVIGLIPLNRQQSETNFILDYAMMCIVPIFYVACYLLINLSHIIGLCLLDSCNSVCKLSSHLFMHLGAFLYLTITLLSLYRRKEFFQQFDARLNDIDAVIQKCQRVAEMDKVKVTAVKHSVAYHFTWLFLFCVFTFALYYDVRSLYLTFGNLAFIPFMVSSFPYLAGSIIQGEFIYHVSVISQRFEQINMLLEKINQEARHRHAPLTVFDIESEGKKERKTVTPITVMDGRTTTGFGNENKFAGEMKRQEGQQKNDDDDLDTSNDEDEDDFDYDNATIAENTGNTSEANLPDLFKLHDKILALSVITNGEFGPQCVPYMAACFVVSIFGIFLETKVNFIVGGKSRLLDYMTYLYVIWSFTTMMVAYIVLRLCCNANNHSKQSAMIVHEIMQKKPAFMLSNDLFYNKMKSFTLQFLHWEGFFQFNGVGLFALDYTFIFSTVSAATSYLIVLLQFDMTAILRNEGLMS

>DmelGr36a

MFDWVGLLLKVLYYYGQIIGLINFEIDWQRGRVVAAQRGILFAIAINVLICMVLLLQISKKFNLDVYFGRANQLHQYVIIVMVSLRMASGISAILNRWRQRAQLMRLVECVLRLFLKKPHVKQMSRWAILVKFSVGVVSNFLQMAISMESLDRLGFNEFVGMASDFWMSAIINMAISQHYLVILFVRAYYHLLKTEVRQAIHESQMLSEIYPRRAAFMTKCCYLADRIDNIAKLQNQLQSIVTQLNQVFGIQGIMVYGGYYIFSVATTYITYSLAINGIEELHLSVRAAALVFSWFLFYYTSAILNLFVMLKLFDDHKEMERILEERTLFTSALDVRLEQSFESIQLQLIRNPLKIEVLDIFTITRSSSAAMIGSIITNSIFLIQYDMEYF

>DmelGr36b

MVDWVVLLLKAVHIYCYLIGLSNFEFDCRTGRVFKSRRCTIYAFMANIFILITIIYNFTAHGDTNLLFQSANKLHEYVIIIMSGLKIVAGLITVLNRWLQRGQMMQLVKDVIRLYMINPQLKSMIRWGILLKAFISFAIELLQVTLSVDALDRQGTAEMMGLLVKLCVSFIMNLAISQHFLVILLIRAQYRIMNAKLRMVIEESRRLSFLQLRNGAFMTRCCYLSDQLEDIGEVQSQLQSMVGQLDEVFGMQGLMAYSEYYLSIVGTSYMSYSIYKYGPHNLKLSAKTSIIVCILITLFYLDALVNCNNMLRVLDHHKDFLGLLEERTVFASSLDIRLEESFESLQLQLARNPLKINVMGMFPITRGSTAAMCASVIVNSIFLIQFDMEFF

>DmelGr36c

MDLESFLLGAVYYYGLFIGLSNFEFDWNTGRVFTKKWSTLYAIALDSCIFALYIYHWTGNTNIVNAIFGRANMLHEYVVAILTGLRIVTGLFTLILRWYQRCKMMDLASKVVRMYVARPQVRRMSRWGILTKFIFGSITDGLQMAMVLSAMGSVDSQFYLGLGLQYWMFVILNMAMMQQHMIMLFVRTQFQLINTELRQVIDEAKDLLLSPRHQGVFMTKCCSLADQIENIARIQSQLQTIMNQMEEVFGIQGAMTYGGYYLSSVGTCYLAYSILKHGYENLSMTLSTVILAYSWCFFYYLDGMLNLSVMLHVQDDYWEMLQILGKRTIFVGLDVRLEEAFENLNLQLIRNPLKITVVKLYDVTRSNTMAMFGNLITHSIFLIQYDIEHF

>DmelGr39aA

MGTRNRKLLFFLHYQRYLGLTNLDFSKSLHIYWLHGTWSSTAIQIVVVGVFMAALLGALAESLYYMETKSQTGNTFDNAVILTTSVTQLLANLWLRSQQKSQVNLLQRLSQVVELLQFEPYAVPQFRWLYRIWLLVCLIYGAMVTHFGINWLTTMQISRVLTLIGFVYRCVLANFQFTCYTGMVVILKKLLQVQVKQLEHLVSTTTISMAGVAGCLRTHDEILLLGQRELIAVYGGVILFLFIYQVMQCILIFYISNLEGFHSSNDLVLIFCWLAPMLFYLILPLVVNDIHNQANKTAKMLTKVPRTGTGLDRMIEKFLLKNLRQKPILTAYGFFALDKSTLFKLFTAIFTYMVILVQFKEMENSTKSINKF

>DmelGr39aB

MDFQPGELCAYYRLCRYLGIFCIDYNPTKKKFRLRRSVLCYIVHFALQAYLVGCISVMVTYWRRCFKSELTTTGNHFDRLVMVIALGILVVQNAWLIWLQAPHLRIVRQIEFYRRNHLANVRLLLPKRLLWLIIATNVVYMANFIKTCIFEWLTDASRLFVITSLGFPLRYLVTSFTMGTYFCMVHIVRLVLDWNQSQINAIIDESADLKMTSPNRLRLRVCLEMHDRLMLLCNDEISLVYGFIAWLSWMFASLDVTGVIYLTMVIQTKKSIVLKLITNVVWLSPTFMTCAASFMSNRVTIQANKTAKMLTKVPRTGTGLDRMIEKFLLKNLRQKPILTAYGFFALDKSTLFKLFTAIFTYMVILVQFKEMENSTKSINKF

>DmelGr39aC

MKRNAFEELRVQLRTLKWLGVLRFTIDFNKCLVRENASEERSAWLYLIGVVGITCSLIVYSTYFPSHFIMGKHNTTGNCYALINIRSCSIVTMLIYTQLYIQRFRFVALLQSILRFNQISGSHREEGRFAFYYYTHLSLLIICMLNYAYGYWTAGVRLTTIPIYLLQYGFSYLFLGQVVVLFACIQQILLSILKYYNQVVLKNIKSSKESREFYYNFCKYNQVIWLSYTEINHCFGLLLLLVTGLILLITPSGPFYLVSTIFEGRFRQNWQFSLMSFTAILWSLPWIVLLVLAMGRNDVQKEANKTAKMLTKVPRTGTGLDRMIEKFLLKNLRQKPILTAYGFFALDKSTLFKLFTAIFTYMVILVQFKEMENSTKSINKF

>DmelGr39aD

MSKVCRDLRIYLRLLHIMGMMCWHFDSDHCQLVATSGSERYAVVYAGCILVSTTAGFIFALLHPSRFHIAIYNQTGNFYEAVIFRSTCVVLFLVYVILYAWRHRYRDLVQHILRLNRRCASSCTNQQFLHNIILYGMLTILCFGNYLHGYTRAGLATLPLALCMLVYIFAFLVLCLLLMFFVSLKQVMTAGLIHYNQQLCQGDLISGLRGRQQILKLCGGELNECFGLLMLPIVALVLLMAPSGPFFLISTVLEGKFRPDECLIMLLTSSTWDTPWMIMLVLMLRTNGISEEANKTAKMLTKVPRTGTGLDRMIEKFLLKNLRQKPILTAYGFFALDKSTLFKLFTAIFTYMVILVQFKEMENSTKSINKF

>DmelGr39b

MLYSFHPYLKYFALLGLVPWSESCAQSKFVQKVYSAILIILNAVHFGISIYFPQSAELFLSLMVNVIVFVARIVCVTVIILQVMVHYDDYFRFCREMKYLGLRLQCELKIHVGRLKWQSYAKILALGIGFLVTVLPSIYVALSGSLLYFWSSLLSILIIRMQFVLVLLNVELLGHHVSLLGIRLQNVLECHLMGANCTLDGNANRLCSLEFLLALKQSHMQLHYLFTHFNDLFGWSILGTYVVLFSDSTVNIYWTQQVLVEVYEYKYLYATFSVFVPSFFNILVFCRCGEFCQRQSVLIGSYLRNLSCHPSIGRETSYKDLLMEFILQVEQNVLAINAEGFMSTDNSLLMSILAAKVTYLIVLMQFSSV

>DmelGr43aNEW

MEISQPSIGIFYISKVLALAPYATVRNSKGRVEIGRSWLFTVYSATLTVVMVFLTYRGLLFDANSEIPVRMKSATSKVVTALDVSVVVMAIVSGVYCGLFSLNDTLELNDRLNKIDNTLNAYNNFRRDRWRALGMAAVSLLAISILVGLDVGTWMRIAQDMNIAQSDTELNVHWYIPFYSLYFILTGLQVNIANTAYGLGRRFGRLNRMLSSSFLAENNATSAIKPQKVSTVKNVSVNRPAMPSALHASLTKLNGETLPSEAAAKNKGLLLKSLADSHESLGKCVHLLSNSFGIAVLFILVSCLLHLVATAYFLFLELLSKRDNGYLWVQMLWICFHFLRLLMVVEPCHLAARESRKTIQIVCEIERKVHEPILAEAVKKFWQQLLVVDADFSACGLCRVNRTILTSFASAIATYLVILIQFQRTNG

>DmelGr47a

MAFTSSQLCSLLTKFTALNGLNTYYFDTKTNAFRVSSKLKIYCAIHHALCVLALAHMSYSTASNLRVSVTVLTIGGTMACCVKSCWEKAQGIRNLARGLVTMEQKYFAGRPSGLLLKCRYYIKITFGSITLLRIHLIQPIYMRRLLPSQFYLNVGAYWLLYNMLLAAVLGFYFLLWEMCRIQKLINDQMTLILARSGQRNRLKKMQHCLRLYSKLLLLCDQFNSQLGHVAIWVLACKSWCQITFGYEIFQMVAAPKSIDLTMSMRVFVIFTYIFDAMNLFLGTDISELFSTFRADSQRILRETSRLDRLLSMFALKLALHPKRVVLLNVFTFDRKLTLTLLAKSTLYTICCLQNDYNKLKA

>DmelGr47bNEW

MQRDDGFVYCYGNLYSLLLYWGLVTIRVRSPDRGGAFSNRWTVCYALFTRSFMVICFMATVMTKLRDPEMSAAMFGHLSPLVKAIFTWECLSCSVTYIEYCLSLDLQKDRHLKLVARMQEFDRSVLMVFPHVQWNYRRARLKYWYGTVIVGFCFFSFSISLIFDTTRCTCGIPSTLLMAFTYTLLTSSVGLLGFVHIGIMDFIRVRLRLVQQLLHQLYQADDSSEVHERIAYLFEMSKRCSFLLAELNGVFGFAAAAGIFYDFTIMTCFVYVICQKLLEREPWDPEYVYMLLHVAIHTYKVVITSTYGYLLLREKRNCMHLLSQYSRYFSGQDVARRKTEDFQHWRMHNRQAAMVGSTTLLSVSTIYLVYNGMANYVIILVQLLFQQQQIKDHQLTSGKDVDIVGPMGPITHMD

>DmelGr57a

MAVLYFFREPETVFDCAAFICILQFLMGCNGFGIRRSTFRISWASRIYSMSVAIAAFCCLFGSLSVLLAEEDIRERLAKADNLVLSISALELLMSTLVFGVTVISLQVFARRHLGIYQRLAALDARLMSDFGANLNYRKMLRKNIAVLGIVTTIYLMAINSAAVQVASGHRALFLLFALCYTIVTGGPHFTGYVHMTLAEMLGIRFRLLQQLLQPEFLNWRFPQLHVQELRIRQVVSMIQELHYLIQEINRVYALSLWAAMAHDLAMSTSELYILFGQSVGIGQQNEEENGSCYRMLGYLALVMIPPLYKLLIAPFYCDRTIYEARRCLRLVEKLDDWFPQKSSLRPLVESLMSWRIQAKIQFTSGLDVVLSRKVIGLFTSILVNYLLILIQFAMTQKMGEQIEQQKIALQEWIGF

>DmelGr58a

MLLKFMYIYGIGCGLMPAPLKKGQFLLGYKQRWYLIYTACLHGGLLTVLPFTFPHYMYDDSYMSSNPVLKWTFNLTNITRIMAMFSGVLLMWFRRKRILNLGENLILHCLKCKTLDNRSKKYSKLRKRVRNVLFQMLLVANLSILLGALILFRIHSVQRISKTAMIVAHITQFIYVVFMMTGICVILLVLHWQSERLQIALKDLCSFLNHEERNSLTLSENKANRSLGKLAKLFKLFAENQRLVREVFRTFDLPIALLLLKMFVTNVNLVYHGVQFGNDTIETSSYTRIVGQWVVISHYWSAVLLMNVVDDVTRRSDLKMGDLLREFSHLELVKRDFHLQLELFSDHLRCHPSTYKVCGLFIFNKQTSLAYFFYVLVQVLVLVQFDLKNKVEKRN

>DmelGr58b

MLHPKLGRVMNVVYYHSVVFALMSTTLRIRSCRKCLRLEKVSRTYTIYSFFVGIFLFLNLYFMVPRIMEDGYMKYNIVLQWNFFVMLFLRAIAVVSCYGTLWLKRHKIIQLYKYSLIYWKRFGHITRAIVDKKELLDLQESLARIMIRKIILLYSAFLCSTVLQYQLLSVINPQIFLAFCARLTHFLHFLCVKMGFFGVLVLLNHQFLVIHLAINALHGRKARKKWKALRSVAAMHLKTLRLARRIFDMFDIANATVFINMFMTAINILYHAVQYSNSSIKSNGWGILFGNGLIVFNFWGTMALMEMLDSVVTSCNNTGQQLRQLSDLPKVGPKMQRELDVFTMQLRQNRLVYKICGIVELDKPACLSYIGSILSNVIILMQFDLRRQRQPINDRQYLIHLMKNKTKV

>DmelGr58c

MNQYFLLHTYFQVSRLIGLCNLHYDSSNHRFILNHVPTVVYCVILNVVYLLVLPFALFVLTGNIYHCPDAGMFGVVYNVVALTKLLTMLFLMSSVWIQRRRLYKLGNDLMKMLHKFRFNLGNDCRNRCLCKGLLTSSRFVLLTQQLLTRDSVVNCESNSSLRQAMVPYQSAAIVYALIMILLMSYVDMTVYMVEVAGNWLLVNMTQGVREMVQDLEVLPERNGIPREMGLMQILAAWRKLWRRCRRLDALLKQFVDIFQWQVLFNLLTTYIFSIAVLFRLWIYLEFDKNFHLWKGILYAIIFLTHHVEIVMQFSIFEINRCKWLGLLEDVGNLWDINYSGRQCIKSSGTILSRKLEFSLLYMNRKLQLNPKRVRRLHIVGLFDLSNLTVHNMTRSIITNVLVLCQIAYKKYG

>DmelGr59a

MKRIGQAYNVYAVFIGMTSYETMGGKFRQSRITRIYCLLINAIFLTLLPSAFWKSAKLLSTADWMPSYMRVTPYIMCTINYAAIAYTLISRCYRDAMLMDLQRIVLEVNREMLRTGKKMNSLLRRMFFLKTFTLTYSCLSYILAVFIYQWKAQNWSNLCNGLLVNISLTILFVNTFFYFTSLWHIARGYDFVNQQLNEIVACQSMDLERKSKELRGLWALHRNLSYTARRINKHYGPQMLAMRFDYFIFSIINACIGTIYSTTDQEPSLEKIFGSLIYWVRSFDFFLNDYICDLVSEYQMQPKFFAPESSMSNELSSYLIYESSTRLDLLVCGLYRVNKRKWLQMVGSIVVHSSMLFQFHLVMRGGL

>DmelGr59b

MVYWMIKLYFRYSLAIGITSQQFSNRKFFSTLFSRTYALIANIVTLIMLPIVMWQVQLVFQQKKTFPKLILITNNVREAVSFLVILYTVLSRGFRDTAFKEMQPLLLTLFREEKRCGFKGIGGVRRSLRILLFVKFFTLSWLCVTDVLFLLYSTDALIWVNVLRFFFKCNTNNILEMVPMGYFLALWHIARGFDCVNRRLDQIVKSKSTRKHRELQHLWLLHACLTKTALNINKIYAPQMLASRFDNFVNGVIQAYWGAVFTFDLSTPFFWVVYGSVQYHVRCLDYYLIDNMCDVAVEYHDSAKHSWSEVRWTKEISSYVIYANSTKLQLWSCGLFQANRSMWFAMISSVLYYILVLLQFHLVMRK

>DmelGr59c

MVDLVKTILLIAYWYGLAVGVSNFEVDWLTGEAIATRRTTIYAAVHNASLITLLILFNLGNNSLKSEFISARYLHEYFFMLMTAVRISAVLLSLITRWYQRSRFIRIWNQILALVRDRPQVVRGRWYRRSIILKFVFCVLSDSLHTISDVSAQRKRITADLIVKLSLLATLTTIFNMIVCQYYLAMVQVIGLYKILLQDLRCLVRQAECICSIRNRRGGVYSIQCCSLADQLDLIAERHYFLKDRLDEMSDLFQIQSLSMSLVYFFSTMGSIYFSVCSILYSSTGFGSTYWGLLLIVLSTASFYMDNWLSVNIGFHIRDQQDELFRVLADRTLFYRELDNRLEAAFENFQLQLASNRHEFYVMGLFKMERGRLIAMLSSVITHTMVLVQWEIQNDES

>DmelGr59d

MADLLKLCLRIAYAYGRLTGVINFKIDLKTGQALVTRGATLISVSTHLLIFALLLYQTMRKSVVNVMWKYANSLHEYVFLVIAGFRVVCVFLELVSRWSQRRTFVRLFNSFRRLYQRNPDIIQYCRRSIVSKFFCVTMTETLHIIVTLAMMRNRLSIALALRIWAVLSLTAIINVIITQYYVATACVRGRYALLNKDLQAIVTESQSLVPNGGGVFVTKCCYLADRLERIAKSQSDLQELVENLSTAYEGEVVCLVITYYLNMLGTSYLLFSISKYGNFGNNLLVIITLCGIVYFVFYVVDCWINAFNVFYLLDAHDKMVKLLNKRTLFQPGLDHRLEMVFENFALNLVRNPLKLHMYGLFEFGRGTSFAVFNSLLTHSLLLIQYDVQNF

>DmelGr59e

MDSSYWENLLLTINRFLGVYPSGRVGVLRWLHTLWSLFLLMYIWTGSIVKCLEFTVEIPTIEKLLYLMEFPGNMATIAILVYYAVLNRPLAHGAELQIERIITGLKGKAKRLVYKRHGQRTLHLMATTLVFHGLCVLVDVVNYDFEFWTTWSSNSVYNLPGLMMSLGVLQYAQPVHFLWLVMDQMRMCLKELKLLQRPPQGSTKLDACYESAFAVLVDAGGGSALMIEEMRYTCNLIEQVHSQFLLRFGLYLVLNLLNSLVSICVELYLIFNFFETPLWEESVLLVYRLLWLAMHGGRIWFILSVNEQILEQKCNLCQLLNELEVCSSRLQRTINRFLLQLQRSIDQPLEACGIVTLDTRSLGGFIGVLMAIVIFLIQIGLGNKSLMGVALNRSNWVYV

>DmelGr59f

MRSSATKGAKLKNSPRERLSSFNPQYAERYKELYRTLFWLLLISVLANTAPITILPGCPNRFYRLVHLSWMILWYGLFVLGSYWEFVLVTTQRVSLDRYLNAIESAIYVVHIFSIMLLTWQCRNWAPKLMTNIVTSDLNRAYTIDCNRTKRFIRLQLFLVGIFACLAIFFNIWTHKFVVYRSILSINSYVMPNIISSISFAQYYLLLQGIAWRQRRLTEGLERELTHLHSPRISEVQKIRMHHANLIDFTKAVNRTFQYSILLLFVGCFLNFNLVLFLVYQGIENPSMADFTKWVCMLLWLAMHVGKVCSILHFNQSIQNEHSTCLTLLSRVSYARKDIQDTITHFIIQMRTNVRQHVVCGVINLDLKFLTTLLVASADFFIFLLQYDVTYEALSKSVQGNVTRYK

>DmelGr61a

MSRTSDDIRKHLKVRRQKQRAILAMRWRCAQGGLEFEQLDTFYGAIRPYLCVAQFFGIMPLSNIRSRDPQDVKFKVRSIGLAVTGLFLLLGGMKTLVGANILFTEGLNAKNIVGLVFLIVGMVNWLNFVGFARSWSHIMLPWSSVDILMLFPPYKRGKRSLRSKVNVLALSVVVLAVGDHMLYYASGYCSYSMHILQCHTNHSRITFGLYLEKEFSDIMFIMPFNIFSMCYGFWLNGAFTFLWNFMDIFIVMTSIGLAQRFQQFAARVGALEGRHVPEALWYDIRRDHIRLCELASLVEASMSNIVFVSCANNVYVICNQALAIFTKLRHPINYVYFWYSLIFLLARTSLVFMTASKIHDASLLPLRSLYLVPSDGWTQEVQRFADQLTSEFVGLSGYRLFCLTRKSLFGMLATLVTYELMLLQIDAKSHKGLRCA

>DmelGr63a

MRPSGEKVVKGHGQGNSGHSLSGMANYYRRKKGDAVFLNAKPLNSANAQAYLYGVRKYSIGLAERLDADYEAPPLDRKKSSDSTASNNPEFKPSVFYRNIDPINWFLRIIGVLPIVRHGPARAKFEMNSASFIYSVVFFVLLACYVGYVANNRIHIVRSLSGPFEEAVIAYLFLVNILPIMIIPILWYEARKIAKLFNDWDDFEVLYYQISGHSLPLKLRQKAVYIAIVLPILSVLSVVITHVTMSDLNINQVVPYCILDNLTAMLGAWWFLICEAMSITAHLLAERFQKALKHIGPAAMVADYRVLWLRLSKLTRDTGNALCYTFVFMSLYLFFIITLSIYGLMSQLSEGFGIKDIGLTITALWNIGLLFYICDEAHYASVNVRTNFQKKLLMVELNWMNSDAQTEINMFLRATEMNPSTINCGGFFDVNRTLFKGLLTTMVTYLVVLLQFQISIPTDKGDSEGANNITVVDFVMDSLDNDMSLMGASTLSTTTVGTTLPPPIMKLKGRKG

>DmelGr64a

MKGPNLNFRKTPSKDNGVKQVESLARPETPPPKFVEDSNLEFNVLASEKLPNYTNLDLFHRAVFPFMFLAQCVAIMPLVGIRESNPRRVRFAYKSIPMFVTLIFMIATSILFLSMFTHLLKIGITAKNFVGLVFFGCVLSAYVVFIRLAKKWPAVVRIWTRTEIPFTKPPYEIPKRNLSRRVQLAALAIIGLSLGEHALYQVSAILSYTRRIQMCANITTVPSFNNYMQTNYDYVFQLLPYSPIIAVLILLINGACTFVWNYMDLFIMMISKGLSYRFEQITTRIRKLEHEEVCESVFIQIREHYVKMCELLEFVDSAMSSLILLSCVNNLYFVCYQLLNVFNKLRWPINYIYFWYSLLYLIGRTAFVFLTAADINEESKRGLGVLRRVSSRSWCVEVERLIFQMTTQTVALSGKKFYFLTRRLLFGMAGTIVTYELVLLQFDEPNRRKGLQPLCA

>DmelGr64b

MPQGETFHRAVSNVLFISQIYGLLPVSNVRALDVADIRFRWCSPRILYSLLIGILNLSEFGAVINYVIKVTINFHTSSTLSLYIVCLLEHLFFWRLAIQWPRIMRTWHGVEQLFLRVPYRFYGEYRIKRRIYIVFTIVMSSALVEHCLLLGNSFHLSNMERTQCKINVTYFESIYKWERPHLYMILPYHFWMLPILEWVNQTIAYPRSFTDCFIMCIGIGLAARFHQLYRRIAAVHRKVMPAVFWTEVREHYLALKRLVHLLDAAIAPLVLLAFGNNMSFICFQLFNSFKNIGVDFLVMLAFWYSLGFAVVRTLLTIFVASSINDYERKIVTALRDVPSRAWSIEVQRFSEQLGNDTTALSGSGFFYLTRSLVLAMGTTIITYELMISDVINQGSIRQKTQYCREY

>DmelGr64c

MQQSGQKGTRNTLQHAIGPVLVIAQFFGVLPVAGVWPSCRPERVRFRWISLSLLAALILFVFSIVDCALSSKVVFDHGLKIYTIGSLSFSVICIFCFGVFLLLSRRWPYIIRRTAECEQIFLEPEYDCSYGRGYSSRLRLWGVCMLVAALCEHSTYVGSALYNNHLAIVECKLDANFWQNYFQRERQQLFLIMHFTAWWIPFIEWTTLSMTFVWNFVDIFLILICRGMQMRFQQMHWRIRQHVRQQMPNEFWQRIRCDLLDLSDLLGIYDKELSGLIVLSCAHNMYFVCVQIYHSFQSKGNYADELYFWFCLSYVIIRVLNMMFAASSIPQEAKEISYTLYEIPTEFWCVELRRLNEIFLSDHFALSGKGYFLLTRRLIFAMAATLMVYELVLINQMAGSEVQKSFCEGGVGSSKSIFS

>DmelGr64d

MLRSHLSVHGLQMERSVQENTLHYTIGHVLIIARIFGVLPLAGINPNGKPENVRFRWFSPYILFFVVAFTFVIADFMLSTKIVLNDGLQLYTMGSLSFSVICIFCFGSFIKLSRRWPHIIRETALCERIFLKPCYANQEGLNFTRFLRRWALILLVAALCEHLTYVGSAAWSNYVQIRDCNLKVGFVENYFLRERQELFSVFEYRAWMVFFIEWNTMAMTFVWNFGDIFLFLMCRGLKIRFQQLHWRIRQNLGKPMAKEFWQEIRSDFLDLDSLLKLYDKELSGLILVCCAHNMYFICVQVYHSFQVKGAFMDELYFWFCLLYVISRLMNMMLAASSIPQEIKDISNTLYEVRSSPWCDELGRLSEMLRNETFALSGMGYFYVTRRLIFAMAGALMGYELVLFRQMQGAVVQKSICSRGPGSSMSIFFS

>DmelGr64eNEW

MARTTGDPAKRRRCMSRIKFWRRSRVGSEATLGIIKYRVVEKDTKRFKLSLIKAWLLRIRQEDYKYSGSFQEAIKPVLIIAQIFALMPVRKVSSKFAEDLTFTWFSVRSYYALVTILFFGVSSGYMVAFVTSVSFNFDSVETLVFYLSIFLISLSFFQLARKWPEIAQSWQLVEAKLPPLKLPKERRSLAQHINMITIVATTCSLVEHIMSMLSMGYYVNSCPRWPDRPIDSFLYLSFSSVFYFVDYTRFLGIVGKVVNVLSTFAWNFNDIFVMAVSVALAARFRQLNDYMMREARLPTTVDYWMQCRINFRNLCKLCEEVDDAISTITLLCFSNNLYFICGKILKSMQAKPSIWHALYFWFSLVYLLGRTLILSLYSSSINDESKRPLVIFRLVPREYWCDELKRFSEEVQMDNVALTGMKFFRLTRGVVISVAGTIVTYELILLQFNGEEKVPGCFEN

>DmelGr64fNEW

MKILPKLERKLRRLKKRVTRTSLFRKLDLVHERARKKAFQESCETYKNQIENEYEIRNSLPKLSRSDKEAFLSDGSFHQAVGRVLLVAEFFAMMPVKGVTGKHPSDLSFSWRNIRTCFSLLFIASSLANFGLSLFKVLNNPISFNSIKPIIFRGSVLLVLIVALNLARQWPQLMMYWHTVEKDLPQYKTQLTKWKMGHTISMVMLLGMMLSFAEHILSMVSAINYASFCNRTADPIQNYFLRTNDEIFFVTSYSTTLALWGKFQNVFSTFIWNYMDLFVMIVSIGLASKFRQLNDDLRNFKGMNMAPSYWSERRIQYRNICILCDKMDDAISLITMVSFSNNLYFICVQLLRSLNTMPSVAHAVYFYFSLIFLIGRTLAVSLYSSSVHDESRLTLRYLRCVPKESWCPEVKRFTEEVISDEVALTGMKFFHLTRKLVLSVAGTIVTYELVLIQFHEDNDLWDCDQSYYS

>DmelGr66aNEW

MDNMAQAEDAVQPLLQQFQQLFFISKIAGILPQDLEKFRSRNLLEKSRNGMIYMLSTLILYVVLYNILIYSFGEEDRSLKASQSTLTFVIGLFLTYIGLIMMVSDQLTALRNQGRIGELYERIRLVDERLYKEGCVMDNSTIGRRIRIMLIMTVIFELSILVSTYVKLVDYSQWMSLLWIVSAIPTFINTLDKIWFAVSLYALKERFEAINATLEELVDTHEKHKLWLRGNQEVPPPLDSSQPPQYDSNLEYLYKELGGMDIGSIGKSSVSGSGKNKVAPVAHSMNSFGEAIDAASRKPPPPPLATNMVHESELGNAAKVEEKLNNLCQVHDEICEIGKALNELWSYPILSLMAYGFLIFTAQLYFLYCATQYQSIPSLFRSAKNPFITVIVLSYTSGKCVYLIYLSWKTSQASKRTGISLHKCGVVADDNLLYEIVNHLSLKLLNHSVDFSACGFFTLDMETLYGVSGGITSYLIILIQFNLAAQQAKEAIQTFNSLNDTAGLVGAATDMDNISSTLRDFVTTTMTPAV

>DmelGr68a

MKIYQDIYPISKPSQIFAILPFYSGDVDDGFRFGGLGRWYGRLVALIILIGSLTLGEDVLFASKEYRLVASAQGDTEEINRTIETLLCIISYTMVVLSSVQNASRHFRTLHDIAKIDEYLLANGFRETYSCRNLTILVTSAAGGVLAVAFYYIHYRSGIGAKRQIILLLIYFLQLLYSTLLALYLRTLMMNLAQRIGFLNQKLDTFNLQDCGHMENWRELSNLIEVLCKFRYITENINCVAGVSLLFYFGFSFYTVTNQSYLAFATLTAGSLSSKTEVADTIGLSCIWVLAETITMIVICSACDGLASEVNGTAQILARIYGKSKQFQNLIDKFLTKSIKQDLQFTAYGFFSIDNSTLFKIFSAVTTYLVILIQFKQLEDSKVEDISQA

>DmelGr77a

MPLPLGDPLALAVSPQLGYIRITAMPRWLQLPGMSALGILYSLTRVFGLMATANWSPRGIKRVRQSLYLRIHGCVMLIFVGCFSPFAFWCIFQRMAFLRQNRILLMIGFNRYVLLLVCAFMTLWIHCFKQAEIIGCLNRLLKCRRRLRRLMHTRKLKDSMDCLATKGHLLEVVVLLSSYLLSMAQPIQILKDDPEVRRNFMYACSLVFVSVCQAILQLSLGMYTMAILFLGHLVRHSNLLLAKILADAEHIFESSQKAGFWPNRQELYKGQQKWLALELWRLLHVHHQLLKLHRSICSLCAVQAVCFLGFVPLECTIHLFFTYFMKYSKFILRKYGRSFPLNYFAIAFLVGLFTNLLLVILPTYYSERRFNCTREIIKGGGLAFPSRITVKQLRHTMHFYGLYLKNVEHVFAVSACGLFKLNNAILFCIVGAILEYLMILIQFDKVLNK

>DmelGr85a

MYSLIEAQLLGGKLVNRVMASLRRIIQRSLGYFCALNGILDFNTDIGTGNLRRYRVLFMYRLLHNFAVISLTLKFLFDFTDHFKYIESSTLITVNFFTYFTLVFFALLSSMGSCYQWQNRILAVLKELKHQRDLSRHMGYRVPRSKQNSIDYLLFALTVLLILRLSIHLATFTLSARMGFNHPCNCFLPECMIFSMNYLLFAILAEITRCWWSLQSGLKMVLLNRQLSTVAFNLWEIERLHTRFQCLIDLTSEVCSIFRYVTLAYMARNLWSGIVAGYLLVRFVIGNGLQDVELVYLVFSFITCIQPLMLSLLVNSMTSTTGSLVEVTRDILKISHKKSVNLERSIEWLSLQLTWQHTHVTIFGVFRINRSLAFRSASLILVHVLYMVQSDYISITN

>DmelGr89a

MLRFPHVCGLCLLLKYWQILALAPFRTSEPMVARCQRWMTLIAVFRWLLLTSMAPFVLWKSAAMYEATNVRHSMVFKTIALATMTGDVCISLALLGNHLWNRRELANLVNDLARLHRRRRLSWWSTLFLWLKLLLSLYDLLCSVPFLKGAGGRLPWSQLVAYGVQLYFQHVASVYGNGIFGGILLMLECYNQLEREEPTNLARLLQKEYSWLRLIQRFVKLFQLGIFLLVLGSFVNIMVNIYAFMSYYVSLHGVPLTISNNCLVLAIQLYAVILAAHLCQVRSAKLRKKCLQLEYVPEGLTQEQAMASTPFPVLTPTGNVKFRILGVFILDNSFWLFLVSYAMNFIVVILQTSFEHINHGEI

>DmelGr92a

MFEFLHQMSAPKLSTSILRYIFRYAQFIGVIFFCLHTRKDDKTVFIRNWLKWLNVTHRIITFTRFFWVYIASISIKTNRVLQVLHGMRLVLSIPNVAVILCYHIFRGPEIIDLINQFLRLFRQVSDLFKTKTPGFGGRRELILILLNLISFAHEQTYLWFTIRKGFSWRFLIDWWCDFYLVSATNIFIHINSIGYLSLGVLYSELNKYVYTNLRIQLQKLNTSGSKQKIRRVQNRLEKCISLYREIYHTSIMFHKLFVPLLFLALIYKVLLIALIGFNVAVEFYLNSFIFWILLGKHVLDLFLVTVSVEGAVNQFLNIGMQFGNVGDLSKFQTTLDTLFLHLRLGHFRVSILGLFDVTQMQYLQFLSALLSGLAFIAQYRMQVGNG

>DmelGr93a

MFSSSSAMTGKRAESWSRLLLLWLYRCARGLLVLSSSLDRDKLQLKATKQGSRNRFLHILWRCIVVMIYAGLWPMLTSAVIGKRLESYADVLALAQSMSVSILAVISFVIQARGENQFREVLNRYLALYQRICLTTRLRHLFPTKFVVFFLLKLFFTLCGCFHEIIPLFENSHFDDISQMVGTGFGIYMWLGTLCVLDACFLGFLVSGILYEHMANNIIAMLKRMEPIESQDERYRMTKYRRMQLLCDFADELDECAAIYSELYHVTNSFRRILQWQILFYIYLNFINICLMLYQYILHFLNDDEVVFVSIVMAFVKLANLVLLMMCADYTVRQSEVPKKLPLDIVCSDMDERWDKSVETFLGQLQTQRLEIKVLGFFHLNNEFILLILSAIISYLFILIQFGITGGFEASEDIKNRFD

>DmelGr93b

MVYGFTMSGLLVMPRILRCLNVSRISAILLRSCFLYGTFFGVITFRIERKDSQLVAINRRGYLWICLVIRLLASCFYGYSYDAWSGQYEDMYLRAFFGFRLIGCLICSVIILVMQFWFGEELINLVNRFLQLFRRMQSLTNSPKNRFGDRAEFLLMFSKVFSLLFVFMAFRLMLSPWFLLTLVCDLYTSVGTGMITHLCFVGYLSIGVLYRDLNNYVDCQLRAQLRSLNGENNSFRNNPQPTRQAISNLDKCLYLYDEIHQVSRSFQQLFDLPLFLSLAQSLLAMSMVSYHAILRRQYSFNLWGLVIKLLIDVVLLTMSVHSAVNGSRLIRRLSFENFYVTDSQSYHQKLELFLGRLQHQELRVFPLGLFEVSNELTLFFLSAMVTYLVFLVQYG

MQSQQI

>DmelGr93c

MIERLKKVSLPALSAFILFCSCHYGRILGVICFDIGQRTSDDSLVVRNRHQFKWFCLSCRLISVTAVCCFCAPYVADIEDPYERLLQCFRLSASLICGICIIVVQVCYEKELLRMIISFLRLFRRVRRLSSLKRIGFGGKREFFLLLFKFICLVYELYSEICQLWHLPDSLSLFATLCEIFLEIGSLMIIHIGFVGYLSVAALYSEVNSFARIELRRQLRSLERPVGGPVGRKQLRIVEYRVDECISVYDEIERVGRTFHRLLELPVLIILLGKIFATTILSYEVIIRPELYARKIGMWGLVVKSFADVILLTLAVHEAVSSSRMMRRLSLENFPITDHKAWHMKWEMFLSRLNFFEFRVRPLGLFEVSNEVILLFLSSMITYFTYVVQYGIQTNRL

>DmelGr93d

MKATKYSVGILRFMSFYARFLSLVCFRLRKQKDNNVWLEEIWSNRSRWKWISVTLRIVPLCIYAFTYAEWISNRMLITEKFLHSCSLVVSIPCYLSIIHLKICHGPEVTKLVNQYLHIFRLGTLDIRRRSQFGGGRELFLLILSVCCQIHEYVFILVIASRLCGFQHIIWWVSYTYVFIICNSIMCFGFIWHLSLGVLYAELNDNLRFESGFQTAFLRKQQRIRVQKSMALFKEISSVVTSLQDIFNVHLFLSALLTLLQVLVVWYKMIIDLGFSDFRIWSFSLKNLIQTLLPVLAIQEAANQFKQTRERALDIFLVGKSKHWMKSVEIFVTHLNLSEFRVNLLGLFNVSNELFLIIVSAMFCYLVFVTQCVIVYRRRYVI

>DmelGr94a

MDFTSDYAHRRMVKFLTIILIGFMTVFGLLANRYRAGRRERFRFSKANLAFASLWAIAFSLVYGRQIYKEYQEGQINLKDATTLYSYMNITVAVINYVSQMIISDHVAKVLSKVPFFDTLKEFRLDSRSLYISIVLALVKTVAFPLTIEVAFILQQRRQHPEMSLIWTLYRLFPLIISNFLNNCYFGAMVVVKEILYALNRRLEAQLQEVNLLQRKDQLKLYTKYYRMQRFCALADELDQLAYRYRLIYVHSGKYLTPMSLSMILSLICHLLGITVGFYSLYYAIADTLIMGKPYDGLGSLINLVFLSISLAEITLLTHLCNHLLVATRRSAVILQEMNLQHADSRYRQAVHGFTLLVTVTKYQIKPLGLYELDMRLISNVFSAVASFLLILVQADLSQRFKMQ

>DmelGr97a

MRFLRRQTRRLRSIWQRSLPVRFRRGKLHTQLVTICLYATVFLNILYGVYLGRFSFRRKKFVFSKGLTIYSLFVATFFALFYIWNIYNEISTGQINLRDTIGIYCYMNVCVCLFNYVTQWEKTLQIIRFQNSVPLFKVLDSLDISAMIVWRAFIYGLLKIVFCPLITYITLILYHRRSISESQWTSVTTTKTMLPLIVSNQINNCFFGGLVLANLIFAAVNRKLHGIVKEANMLQSPVQMNLHKPYYRMRRFCELADLLDELARKYGFTASRSKNYLRFTDWSMVLSMLMNLLGITMGCYNQYLAIADHYINEEPFDLFLAIVLVVFLAVPFLELVMVARISNQTLVETRRTGELLQRFDLQHADARFKQVVNAFWLQVVTINYKLMPLGLLELNTSLVNKVFSSAIGSLLILIQSDLTLRFSLK

>DmelGr98a

MEQMSGELHAASLLYMRRLMKCLGMLPFGQNLFSKGFCYVLLFVSLGFSSYWRFSFDYEFDYDFLNDRFSSTIDLSNFVALVLGHAIIVLELLWGNCSKDVDRQLQAIHSQIKLQLGTSNSTDRVRRYCNWIYGSLIIRWLIFIVVTIYSNRALTINATYSELVFLARFSEFTLYCAVILFIYQELIVGGSNVLDELYRTRYEMWSIRRLSLQKLAKLQAIHNSLWQAIRCLECYFQLSLITLLMKFFIDTSALPYWLYLSRVEHTRVAVQHYVATVECIKLLEIVVPCYLCTRCDAMQRKFLSMFYTVTTDRRSSQLNAALRSLNLQLSQEKYKFSAGGMVDINTEMLGKFFFGMISYIVICIQFSINFRAKKMSNEQMSQNITSTSAPI

>DmelGr98b

MVAQKSRLLARAFPYLDIFSVFALTPPPQSFGHTPHRRLRWYLMTGYVFYATAILATVFIVSYFNIIAIDEEVLEYNVSDFTRVMGNIQKSLYSIMAIANHLNMLINYRRLGGIYKDIADLEMDMDEASQCFGGQRQRFSFRFRMALCVGVWMILMVGSMPRLTMTAMGPFVSTLLKILTEFVMIMQQLKSLEYCVFVLIIYELVLRLRRTLSQLQEEFQDCEQQDMLQALCVALKRNQLLLGRIWRLEGDVGSYFTPTMLLLFLYNGLTILHMVNWAYINKFLYDSCCQYERFLVCSTLLVNLLLPCLLSQRCINAYNCFPRILHKIRCTSADPNFAMLTRGLREYSLQMEHLKLRFTCGGLFDINLKYFGGLLVTIFGYIIILIQFKVQAIAANRYKKVVN

>DmelGr98c

MEMEAKRSRLLTTARPYLQVLSLFGLTPPAEFFTRTLRKRRRFCWMAGYSLYLIAILLMVFYEFHANIVSLHLEIYKFHVEDFSKVMGRTQKFLIVAIATCNQLNILLNYGRLGLIYDEIANLDLGIDKSSKNFCGKSHWWSFRLRLTLSIGLWMVIIIGVIPRLTLGRAGPFFHWVNQVLTQIILIMLQLKGPEYCLFVLLVYELILRTRHVLEQLKDDLEDFDCGARIQELCVTLKQNQLLIGRIWRLVDEIGAYFRWSMTLLFLYNGLTILHVVNWAIIRSIDPNDCCQLNRLGSITFLSFNLLLTCFFSECCVKTYNSISYILHQIGCLPTAEEFQMLKMGLKEYILQMQHLKLLFTCGGLFDINIKLFGGMLVTLCGYVIIIVQFKIQDFALIGYRQNTSDTS

>DmelGr98d

MEANRSRLLAAARPYIQIYSIFGLTPPIQFFTRTLHKRRRGIVILGYACYLISISLMVIYECYANIVALQKDIHKFHAEDSSKVMGNTQKVLVVAMFVWNQLNILLNFRRLARIYDDIADLEIDLNNASSGFVGQRHWWRFRFRLALSVGLWIVLLVGLTPRFTLVALGPYLHWTNKVLTEIILIMLQLKCTEYCVFVLLIYELILRGRHILQQISVELEGNQSRDSVQELCVALKRNQLLAGRIWGLVNEVSLYFTLSLTLLFLYNELTILQIVNWALIKSVNPNECCQYRRVGTCLLLSINIFLSCLYSEFCIQTYNSISRVLHQMYCLSAAEDYLILKMGLREYSLQMEHLKLIFTCGGLFDINLKFFGGMVVTLFGYIIILVQFKIQFFAQSNFMQNINSTELKAYTA
