# Supplementary material for: Identification of chemosensory genes from the antennal transcriptome of Semiothisa cinerearia
Source: PLoS One. 2020 Aug 7;15(8):e0237134. doi: 10.1371/journal.pone.0237134 (PMC7413487; doi:10.1371/journal.pone.0237134)
Supplement: S9 Table — (DOCX) [file pone.0237134.s017.docx]

>ScinIR1

ADTGAGGDGLRRPRTSPAPPPPRAPSVITAALVVPHKAFGTRDYTKAEKAALAKLPRKQLKLFSLVRLNITLSTLTLTPSPMSILDSLCKEFLAVNVSAILYLMNHEQYGRSTASAQYFLQLAGYLGIPVIAWNADNSGLEKRASHASLRLQLAPSIEHQTAAMLSILERYKWHQFSVVTSAIAGHDDFIQAVRERVIALQDRFKFTILNAVVVKRGSEDLTELVTSEARVMLLYATREEAAEILSAAGDLHLTGENFVWIVTQSVLGSLQQPNKFPVGMLGVHFDTSSSSLIAEIATAVKVFAYGVESYVSEPDNVRYPLGTRLSCGAGAGEARWSTGERFYRHLRNVSVEGEAGRPSIEFTPDGELRAAELKIMNLRPSIGEQLVWEEIGTWNSYPKERLVIKDIVWPGGLHTPPQGVPEKFHMRITFLEEPPYINLALPDPVTSRCSLDRGVICRVAPDSEVAGLDPGVAHGNSSIYQCCSGFCIDLLQQLAEQLGFTYELVRVEDGRWGTLQNGKWNGLIADLVNKKTDMVLTSLIINSDREAVVDFSVPFMETGVAIVVAKRTGIISPTAFLEPFDTASWMLVGAVAIQAATFTIFFFEWLSPSGFDCSTGQESKRVPQNRFSLCRTYWLVWAVLFQASVHVDSPRGFTSRFMTNMWAMFAVVFLAIYTANLAAFMITREEFHELSGIDDPRISRPNAFRPPLKFGTVPWSHTDATLAKYFPLPHSYMTQFNRSTVSAGVTGVLTAELDAFIYDGTVLDYLVSQDEDCRLLTVGSWYAMSGYGLAFTRNSKYLSMFNKRLLDLRSNGDLERLRRYWMTGTCKPNKQEHKSSDPLALEQFLSAFLLLMAGILLAAILLGLEHVYFRYMRAHLAASSAGPCCALVSLSMGQSLTFHGAVVEAATRGYGAGSRGHCRSAVCAAQVWRARHERDVAVARARQLAAALAAHGLAAPPRRLASAAALLAASTDATRPRTLRPAPGADLLPDLDRPLSCGDLRQRERTRVEMETVL*

>ScinIR2

MELLFLSAFFIGMASAASEISLRFVFIIEAQDRDLAGAISRALKDVEASSPGVKLDDEVVLLDREDEDLSYRMLCSALSKGVTAIVDVSWSPWEAAAGAVRGAAHAQPLLSALDQYLRYRNATDAAIIVPTERDVDRALYRLLGASGTRVLTHAGLSRESARALRNMRPEPEFFAVVGPTNFVMDTYARAVKEKLVRRAHRWNLVFTDYPGSNLDLSSLILPTTILYVDESECCKLSGGEDCACPSDAQRPQAILTAIIRQLTSTASKLDPPLSPRESCEESTRNTSSLPQQLAEDVADNRTVLYWDPDRSSLELRSRFVLSTFSPDEGLTQLAGWTAEEQFRLLPGVTLQPLRMFFRIGTSTAVPWTMPKLDPESGVQLVTEEGQPQYEGYCIDLIARLSETMGFDYEIVTPKSGGFGRKLDNGTWDGVVGDLMLGETDMAVGALTMTAEREEVIDFVPPYFEQTGILIVIRKPTRKTSLFKFMTVLRTEVWVSIVAALVCTALMIWLLDRHSPYSARNNPDAYPYPCREFTLKESFWFALTSFTPQGGGEAPKALSGRTLVAAYWLFVVLMLATFTANLAAFLTVERMQTPVSSLEQLARQSRINYTVVEGSTVHQYFINMKFAEDTLYRVWKEITLNATSDQAQYRVWDYPIREQYGHILLAINASGPVADAKTGFEQVNEHTDADFAFIHDSAEIRFEMTRNCNLTEVGEVFAEQPYAIGVQQGSRLQEELSRALLELQKERFLEQLAAKYWNETARQSCPDADESEGITLESLGGVFIATLFGLGLAMITLAWEVFYYKRKEKNKVQALDTSIEKPEPFGEKKGIGMITESVARLRKREKRGKTKQVTIGKSFKAAAEKPAVSYISVYPKNEFRP*

>ScinIR3

VHGYINDKGHLQEPELLKPVKECLHYIIFLTDVKVSAKILGKQSESKVVVVARSSQWAVQEFLAGSLSRMFVNLLVIGHSFKDGDDDALEAPYILYTHKLYTDGLGASLPVVLTSWSHGKYSRNVNLFPLKMTKGYAGHRFEVAAAHQPPFVFRTVKTDLDGGNPRVQWDGIEIRLLKLMAERNNFSIEIKEPQELSLGPGPAVVKEIVNGRADIGIAGMYQTIERFKEMDLAFSHSQDCAVFITLMSTALPRYRAILGPFHWHVWVALTFTYIFGMFPLAFSDKHTLKHLINNSGEIENMFWYVFGTFTNCFTFVGKNSWSKTNKLTTRLLIGWYWIFTIIITSCYTGSIIAFVTLPIFPETVDTIDQLLDGFYRIGTLDRGGWEKWFINSSDPKTNKLMKKLELVTSVEDGIRNTTKAFFWPHAFLGSKAELEYLVQANFSATKSKRAVLHISNECFVPFGVSLGFPKNSLYTAKLSNDVRRMFQSGLTDKIVDEVRWEMQRSSTGKLLAAGAGTLKIPSAEEKGLTLEDTQGMFLLLAVGFLVAATALISEWMGGFTRRCRFLCRQKREREQAASLSREELISPTGSGEIRALSDDTESRIHFDTRTSSAGSRDTLEGQIIHVTQESIMVHEKLEVDGWDSRRSSSVDLDREVNEIFERDRVRRGMVADDATDVVGEERNTTVSKGAFGDHLSNSKEN*

>ScinIR4

YQSYFFLYQSYSFELEILPSLPCTLINFIKLHLLQTVLLPCLILSAQQIFFFHFIDTLHPCPFESASLPFTSPTNSYIVIIILNSFSLISDKKLQLYSVLGGTDSNPPITLQFIDHNQITPANFFNREQNMVFLADLECPGVIKHLDDSSVKTLFRTPFRWILVNNKINQSKIQRHEIKNLIIPEAISHLDILPDAEVILAHRIGDDCFDLYLIYKISSGSPWITERYGHWGIQNRFEKSMISNETMVLRRLDLDGFEITICYVLTDNDSINHLTDEVNDHIDTITKVNFPTTNHLLDFLNAGRKFYFANTWGYKVNGSWNGMTGYLVREEVEIGGSPMFFTSERISIVDYIASPTPTRSKFVFRQPKLSYENNLFLLSFRWTVWYSSLALVFLMFLAIFVVAIWEWKKPGSDKKLKEADPGILRPSVSDIVILIFGATCQQGSPVELKGSLGRIVMLVLFLALMFLYTSYSANIVALLQSSSSQIRTLDDLLHSRLSFGVHDTVFNKYYFSTATEPVRKAIYEKKVAPPGVSPRFMTMEEGVKKMRKGLFAFHMETGVGYKFVGKYFEESEKCGLKEIQYLQVIDPWLAVRKNTPYKEMFKIGMKRIQEHGLQSRENRLLYEKSPKCSGRESNFVSVSMVDCYPALLVLSYGSIIALCVCVIENIIHRRHSLKERVKFSRNRTSFHS*

>ScinIR5

AARADLQFVLADTLAPLTRRRAVSCAAVVCDTVYLNVFDGPLFRRTAKMPFVMTVVEDREDLLSPNYYTLETLRAARKDGCNLYIILLANGYQAARLLRFGDRHRIMDTRARFIILHDFRLFHSELHYLWKRIVNVIFFKYHTQAAGTENKAWFDLTTVPFPNPIRGVLVPRRVDIWRNGKFHYNRPLFTDKTRDLRGETLSVVYLDHVPSVVVTQNNETKKIGGIESEILNTLAQKMNFIPKLYQPPNADLHKWGQKQPNGSFSGLLGEMVNGHADMALGNLQYNPYHLELTDLSIPYTSQCWTFVTPEALTDNSWKTLILPFKLYMWIAVLLVLLITGSIFYGLATYYTTLQTYKKDYKHPGRAKLGEKQEYYEDEKPVGLYLFGEIINSILYTYGMLLVVSLPKLPTGWSIRLLTGWYWLYCILLVVSYRASMTAILANPAPRVTIDTLTELVDSKVTCGGWGAETKQFFVNSLDEIGQKIGEKFVTIDDPMEAADKVAQGVYAYYDNRDFLKYISVKRKNALVMQSIDDKMENSTNVTVAVAAKTESERNLHIMSDCVVHIPISIGFHKNSPLKPLADIYTKRIVEVGLVEKWLNDAMRPIRALEVNEEEVKALMNLQKLYGAFIALGIGYGLSAIALIGEILHWNFVVKRDPKFDKYAVDLYYMNKNKKQ*

>ScinIR6

MNVLKVLSVLILSLKPALCFRNDIRVQILTMVDVIRSYERPSAILANVCYDISDIKILAKELMQVNGAKTFQLVENFSSIEKFKGDGMVILLDLDCLNSGKILEDASQNGKFQNPYRWLLFSSELEWEAIMKSFENLSILIDSDVTVAYNMKSNGTILQKVYRIDSNSKIIPEKFATWSVEDGLSIETEAKRPILMRRTNLQGKKIGISLVTIDNTTTKTDIFILKDLVKDNLAKSSFHNVLPLYAFLNASSEIMFSNTWGYYINNTWNGMIGQISRGEADLCGTVTFMSERRSTVISYLTQPIKIQAKFIFREPPLSLQNNLFYLPFRTTVWISCGALMVLIFVVIYVNAKWEAMKLKVDNNDPSILKPDVSDVAILVVGAVVQQSSYSVLQGSRGRLVIFLLFLICLFLYTAYSANIVALLQSSSDQIRTLSDLLESKLELGVEDQPYTRHYFTTATEPVRKAIYESKVARTGYKPNFMPVEEGVKKLQNLDAPFAFHMNTGLGYRLIEENFLDHEKCGLREIQYVQSGVPWLCCRKQSPFKELYKIGLARILEHGISDRVNRLIYTRKPSCNSRSGSFGSVNMTDFHPVLLCLLYGIITSLLLLVIEILVYKRNNENKMQQKNDVDDRASEASLH*

>ScinIR7

MSIQILNLILFSLVRVIDSQTTQNINVLLINEENNALAEKSFDVAKEYVRRNPSLGLAVDPVIVVGNRSDAKAFLENVCRKYNDMLSAKKTPHVVLDFTMTGVGSETIKSFTAALGLPTISGSFGQAGDLRQWRNLNTNQTKFLLQVMPPADILPEAIRAIVTKQDITNAAIIFDEFFVMDHKYKSLLQNIPTRHVITPVKSFNKDEIKTQLRSLRELDIVNFFVVGSLKTIKNVLDAADENQYFGRKTAWFALSLDKGDISCGCKDATIVYQRPTPDATSRDRLGKIKTTYSMNGEPEITAAFYFDLSLRTFLAVKSLLDSGKWPNDMKYITCDDYDGKNTPNRTLDLKTAFHEVKETPTYAPVFIPEDDPMNGRSYMEFSTDLSAVTVKDGASIGSKALGTWKAGLSNPLSLTDPENMSDYSAQLVYRIVTVEQQPFIIRDDAAPKGFKGYCIDLIEEIRQIVKFDYEITLSPDGNFGTMDENGNWNGIIKELMEKRADIGLTSLSVMAERENVVDFTVPYYDLVGITIMMKLPRNPTSLFKFLTVLENDVWLSILGAYFFTSLLMWVFDKWSPYSYQNNREKYKDDEEKREFTLKECLWFCMTSLTPQGGGEAPKNLSGRLLAATWWLFGFI

>ScinIR8

MKLVIILTLIGAVLGFHRDKFNLVKDYFSYKAVKYVCYLSCEQPFHNTKVVKELILINIRTSVFQIDKDIGKLAEAVFQSRTNVGILMDGACTNTPAVLNEASESILFDATHSWLIFAPDEHNSTDIISMQFQNLNLSIDADIAVVSKNGIEYHIFDVFNFGKIQGNSLEIMLLGSWRPDNGLIIYLQGFKYYNRWDFHNLTLRAISVMLGRGAESDPRLLADAGHTPGVVAMTKVTSQLLNLLKEHHHFRFRYTIVDRWIGDPKPNSTLAVTNSLYWKEQDISSTSARIFPQWLDWVDICFPPTTTLETKFYYLMLEEGVGRYENRFLTPMSAGVWWSTLAASAGCAAVLAAAARLEARAQPAVYALFSVTAALFQQDFEDGGQERKLTSQGRKLTLLVVGVTSMLLYNYYTSSVVSWLLSAAPPSLTSLDQLIQSDLELVFEDIGYTRGWLQNPGFYYYSGYVNPKEDRMREVKVTKAKRTVEPLQSVGTGIELIRTGLYAYHTEPYTASREVSPRLGDEEVCALAALPLMLPAHVYIMVQKHSPYREFFIWSLMRMWERGHVQASRRRFPASMPACSGRRPRALALGQAAPAFLALLQLSALAALILLAECACHRFQPHHLEFRH*

>ScinIR9

GNSDVKIPSQTHEITVEVALASDAARFVADAVESAPEEFKIEAQPIECGSEDKWEAGEAFTSHLLTNPIFAITDKIIVDNITGERTNFNVEIMELSNVGFNRIAKWNADSGFEYTRGDDISDRLAEKWQNKTFRVVSRIGEPYLMEKKPKDGEILTGNDRYEGYSKDLIHEILAETLHVNYEIILEPKNAYGSFDKKTQEWNGLIRFLIDRKADLAICDLTITYERRSAVDFTTPFMTLGISILYSQAPEAETNLFSFLSPFSVDVWIYMAGAYLLVSLLLHILARLAPNDWENPHPCDKSPEELENIWHIKNSCWLTMGSIMTQGSDILPKGYSTRWVCGMWWFFALIMCSSYTANLAAFLTNAAMDDSIKSAEDLSMQNKIKYGTVKDGSTYSFFQHSNESLYQKMWSTMESARPSVFVKDNKVGVDRVKRKTRDYAFFMESSAIEYQLERECTLLQVGGQLDSKGYGIAMPFFSSYRTPVDNAVLKLAESGKLVELKNRWWKLPPDDPHACEKKEDEEEGGSAAELGVSNVGGVFVVLGIGCGMAAFMGMLEFLWNVREVAVEQRMTQSEAFWAELTFALSFWETEKPVKHSRPSSTASGSNPSRASSMLRSAADLFHIELFK*

>ScinIR10

MPTINVLLYTILKKFLFNSYCITIVSDAPIDLHLNSLFTYIIPNRTVDELRDQLLEVSERGCSDYIVNMVDQVLFMTAFDKVSKIGNIRRSDRKVILIPAPGNQSVDAVFSMKEINNIANLLMIIPNNASEQECQEIYDLVTHKFEGPDELVYELIKQPILLDQWDTCKGEFKENANLFPHNMNNLFGKTVKVATFTYKPYVLLNVDTAITPTGRDGLEMRIVEEFCRWVNCSIQILDEWQGQWGEVYENGTGVGIMGSVVEDKADFGMGALYSWYEEFLHLDFSAAEIRSAVTCVAPSPRVLASWAMPLLPFTWNMWYAVLMTLLFASLGLSMAKGSFDGTLITVFGVMISQSQKSAERNWRVRRVTGWLLAAGLILVSAYGAGLASTFTVTNYEPSVDTIQDLVDRKMEWGATHDAWIFSITMSNEPLIKQLVSQFKIHSAEELKRKSFKRSMAFSIEKLPAGYFAIGDYLTQEALEDYTLMLEDFYYEQCVVMMRKSSPYTEKMSALIGRLHQSGLLLTWETQVALKYLNFKVQQEVKLSRLRSDVGFKPLDLHNVVGIFILYGIGVVLSILIFMVENASTKKSGLRIEE*

>ScinIR11

MLMGAAACLHQDNIWLAKDYFSYKNIPYVCYFSCEKQYYNKEVAKEMFLINTRTSVTQIDKSTEFSIEQVLYQWKFSVGVLIDGTCDNASYVLEKASKATLFDATHAWLILAPDGYNTTLYIEETFHILNLSIDADIAVVGANETDKTITDVFNFGKIQANGLEIASLGSWRPGDGLNIYLKGFKYYHRWNFHNLTLRAVSVMLHRPEVFRPELLASIGYTPGVLAITKIASQLLNLIKEQHNFRFNYTIADRWVGSPKPNSTLAVTNSLFWREQDIASTSTRVFAPWLEWVDVCFPPTATIQTKFYYLMLERGAGALENRFLRPLSAGAWWCVACACAACALVLAAAASLEARPRPRAYAFFSVAAVLFQQDFDDGCQVKNISSQGRKLTLLVVGVTSMLLYNYYTSSVVSWLLSAAPPSLTSLDQLIQSDLELVFEDIGYTRGWLQNPGFYYYSGYVNPKEDRMREVKVTKAKRTVEPLQSVGTGIELIRTGLYAYHTEPYTASREVSPRLGDEEVCALAALPLMLPAHVYIMVQKHSPYREFFIWR*

>ScinIR12

MAGAELIISAICNATFCEAVYDNPISDIRLTKKQSDILNLTKEINGKHLRIATYNNYPLSWVEKGDNGTLVGFGVAFAIVDILQERFNFTYDVVVPEQNFEMGGRGPGNSLIGMLNSSQADMAAAFLPKLGKFINMISYSSDLDEGVWKMMLKRPTASAAGSGLMAPFNGLVWYLTLAAVLCYGPCITFLTRLRSRLVKDERHLPLSPSFWFVYSAFIKQGTNLAPEANTTRILFATWWMFIILLSAFYTANLTAFLTLSKFTLDIETPEDLYKKNYRWVSTDGGSVQFIVKNPMEKLYFLNKMVTSGRAEFRNFPNDMDYLPLVDKGAVLVKEVTAIDNLMYGDYLKKAREGVNEVDRCTYVVAPKSFMTKPRAFAFPHDSPLKTLFDTVFVYIVQAGIINYLQLKDLPSTKICPLDLQSKDRQLRNSDLLMTYMIMVTGLCAAAAVFIGEVVFKRYYIKTKLHKEALKPKGRRSKKTSKRLRFEDDGGLPPPYDSLFGNTRYNLNENSKRKIVNGREYYEYTSSTGDTRLIPVRAPSAFLYH*

>ScinIR13

LFIEVYKILDSTELIFTPRASWRDFDNSRSNSTESDSNSLAVYDDSKANRVVQKNGWVEDYRKSKVLSSRRNNVRRHPLTMVNVITDSNDTRNHLNDRLKLHQDSIAKMSYSIVHICFEMLNATEQLIFTHTWGYKDKYGKWQGVVDYLMKKEGDLGTLTIFTQERMQVVDYIAMVGSTAVRFVFREPPLAYLANIFALPFTGMVWLAIFICVLGCALFLYITSKWEASMSMHPLQLDGSWADVLILIIGAVLQQGCTLEPRYAAGRVVMLILFIAMTILYAAYSANIVVLLRAPSSSVRSLPDLLNSPLKLGASDFEYNRYFFKKLNEPVRKAIYDKKIAPKGKKPNFYSMEEGVEKIRQGLFAFHMELNPGYRLIQETYQEHEKCDLVEIDYINEINPWVPGQKRAANKELYRINFIKIRETGVQSCMHRRLHVPRPRCSGAVSTFSSVGVADMYPAMLATLYGFLLSPAVLVLEILYHRLIVARANRAKTIQPFPEDSSLRSYYE*

>ScinIR14

MKLWRICLFACVFGTGRGEDFPSLITANASIAVVLDHQFLGERYQAVLDELKDYIKELARVQLKHGGVVVHYYSWTSISLKKGFLAVFSIASCVDTWDLFSRTQAEELLLFALTEVDCPRLPTREAITATYAEAGEELPQLLLDLRTSNAFKWKAAVILHDDTLSRDMVSRVVQSLTSQIDDDSANPVSVTVFKMKHEINEYLRRKEMYRVLSKLPVKFIGENFIAIVTTDVMTTMADTARELLMSHTSAQWLYVISDTNVHNGNLSGLINTLHEGENVAYIYNVTDDHQDCVNGLMCYSREMMSAFISALEAATQEELRVAAQVSDEEWEAIRPDKLQRKNALLKHMQQYIQVNSRCGNCSTWHVLAAETWGATYKDINEATIVEPSHNHTNNTASPGVIEQIELLNVGFWRPIDAVSFSDALFPHVEHGFRGKELPIVTYHNPPWTILKRNESGAVISHTGLVFDIIHQLAKSKNFT*

>ScinIR15

MPTAVSRRKNLGNLYFKAATVITQPQHFKGWTDLTTRQIDTFPKLTYPLMMLLAEDLHFRYNLKQVDLYGEERNASFDGVSGLLQRREVEIGVTSMFLRADRMRVLHFSSETVELKGAFIFRQPSQSAVSNVFLLPFSRGVWTATGAVLLLSALLLALASWKLRTAHPDLGQVTLAECFSFAIGTICQQGFHLSPGAVPVRVAMVSALISSLFLFTAYSAKIVALLQSPSSAIQTIDQLTRSPLALGVQETTYKRTYFAESKEPATQRLYRRKLLPLGDKAYLSVVDGVARLRTGLFAFQVEESSGYEIISKTFSESEKCGLQQIQAFKLPMVAVPLVKHSGVRDLLAARLRWQREVGLMGRWRRRWLAGRPRCDAGAGGSVSVGLRDVLPALQIWMGGSALAVAILFTEIAARAVEIRKMKHDSNSIDNIDKIPSVATGVVWWETGMPASQDRAFKELGWLG*

>ScinIR16

YYNYSRESYNGTLTDVRPNRSLFRRRMDLMGHTLTMSNVIQDSNKSTYHLPKEDGLDFQNDPIAKISWINAKVAFEMLNATPRYIFSYRWGYRVNGQWSGMIDDLNTGRADIGTNCIVSNTDRLSVVAYTDTLAPFKVRFIFRQPPLSYVANIYSLPFSGNLWVGTAACAVISALAIWGAASWERRFYPIPDATQLDGGISDALFWTMGAVSQQGCDMVPRRTPGRVAGWVVLAALTALYAAYSANIVVLLQAPSHSILTLSQLAASKVTLAANDVDYNHFIFKQYKDPVRVAIYKKIEPPKEKAHFYDLNEGVERIRKGLFAFHSLESQIYRRIEETFTASEKCDLTDIDFMNALDPYAPMKKDSPYLELMRVAFKRMRETGIHTAISRRYHVPKPICANKGSAFSSVGLLDLKPVLALMAIGIGASFAVLLVEIAVFKM*

>ScinIR17

FRIKIISTSKMKHNFNDSTLEMLQSKSAMATMAAVAKGQAALAAACFTVLSDPMPGINYTIPISTQPYSFMIARPQELSRALLFLLPFTTDTWLCLGFAVLLMGPTLYAIHRMSPYYEAMEITRQGGLATIHNCLWYIYGALLQQGGMYLPHADSGRLVVGTWWLVVLVVVTTYSGNLVAFLTFPKQEMPVTTVLELLDNSAFYTWSISKGSYLEMELKKSEEPKYTALLKGAQLSTIPGGMEGSSESSGKLLRQVRSQRHVVFDWRLRLTNLISIDAMKSDSCDFSLSTEEFLNEKLAMIVPAGSPYLAVINKEIKRMHKAGLIAKWLSSYSGRRSRCSAGGGAHEVDNHTVNLSDMQGSFFVLFLGFFSASSVLLFEWLYNRRKRQHEEIVIKPYIE*

>ScinIR18

IVEELCRWINCTIEIVDEYKHQWGEVFENGTGNGVIGSVVDDRADIGISGIYSWYEEFIYADFSTPSIRSSVTCLAPSPRMLASWAMPLRPFNWSMWCAVLVTFLFASLALIMTEGCSSDRVFLTVVGLMISQSQKNAEKSWRVRSVTGWLLVAGLVLVSAYGGGLASTFTVPKYESSIDTMQDLVDRNMEWAATHDAWVFSLTLSDEPMIKQVINLYHTYSEDDLKRKSFGRKTAFGIEKLPGGHYAIGDYLTLEALGDMTVMVEDFYYGEVIAVLRKSSVYTEKLNVLIGRLQQSGLMLAWETQVALKYLNLKVQQEVKMSRLRKNVGFVKPLDTQTVLGIFILYAIGVVLSTSIFIIERLMKNKIPNVE*

>ScinIR19

RTIRKWFDPTVSYTQDEMTWCVPRAGQASTWDNLVIIFQWSTWVATIASFIFIGLLFHYLHYRELGGKVTKWPTNSLLMTFSMLLGWSASFQPRTATFRILIFGWLCFSINMGISYESFLRSFLMHPRFEKQISSERDLIQSGLAFGGREIYRSYFETNNASSFYLYRKYNSTTFAEGIRRAALERNFGVVSSRRQAVYQDQELGRGAPLIYCFPESDNLYKYGVVLLARKWFPMLERFNNIIRSVSENGLIEKWSEELFIRRSSVESTGIIVPLSIQHLLGAFMFIGFMYGASIVVFLAEIAYGWFERRKNT*

>ScinIR20

QNPGFYYYSGYVNPIEDRMREVKVTQAKRTVPPLQSVKVGVKLMRTGMYAFHTEPYTARQEVSAAFSDEELCSLAALQVMPPARLYVLLQKRSPYKEFFVWSMARLWERGHVSASQRRFPDELAACSGRKPRALALGQAAPAFLLLLAGLGLAGGVLLAERACHRFHPPRRLLHRRRGSAESFHFN*

>ScinIR21

GGMCTDDDAGGGDAQPLVLANVGGVFIVLVAGSGLAIVCAFLEMVVDVWFISHRMKVSFKEELIAELKFILSFSGDTKPVRHRESTGSGSGGSKKTEEEVEKRGDDEISVKEEDNRTPTPSARSTHSRHTLHSRRPSNATHMARMRKYSARSARL*

>ScinIR22

YAYHTEPYTASREVSPRLGDEEVCALAALPLMLPAHVYIMVQKHSPYREFFIWSLMRMWERGHVQASRRRFPASMPACSGRRPRALALGQAAPAFLALLQLSALAALILLAECACHRFQPHHLEFRH*

>ScinIR23

MTQGCDILPKAIGTRWVCGMWWFFAVIFCQTYIAQLSASMTSARDIEPINSVEDLANQNKIRYGAYRVGSTLEFFRTSQDKIYSKMYDTMMANEAVLVGSNDEGESRVKKRRRQIRLLYGIFIYRL*

>OfuriGluR1

MSILDSLCKEFLAVNVSAILYLMNHEQYGRSTASAQYFLQLAGY

LGIPVIAWNADNSGLEKRASHASLRLQLAPSIEHQTAAMLSILERYKWHQFSVVTSAI

AGHDDFIQAVRERVTALQDRFKFTILNAVVVKRPSDLNELVTSEARVMLLYATREEAA

DILSTAGDLHLTGENFVWIVTQSVLGSMQQPNKFPVGMLGVHFDTSSSSLIAEIATAV

KVFAYGVESYISDVENIRHPLGTRLSCGGAGAGEARWSTGERFYRHLRNVSVDGEAGR

PNIEFTPDGELRAAELKIMNLRPAIGEQLVWEEIGTWNSYPKERLVIKDIVWPGGLHT

PPQGVPEKFHMRITFLEEPPYINLAPPDPVSGRCSLDRGVICRVAPEVDVAGLEAGAA

HGNSSLYQCCSGFCIDLLQQLAEQLGFTYELVRVEDGRWGTLHQGKWNGLIADLVNKK

TDMVLTSLIINSDREAVVDFSVPFMETGVAIVVAKRTGIISPTAFLEPFDTASWMLVG

AVAIQAATFSIFFFEWLSPSGFDCSTGHNSKRVPQNRFSLCRTYWIVWAVLFQASVHV

DSPRGFTARFMTNMWAMFAVVFLAIYTANLAAFMITREEFHELSGIDDPRISRPLTHR

PALKFGTVPWSHTDATLAKYFSEPHAYMTQYNRSTVSAGVTSVLTGELDAFIYDGTVL

DYLVSQDEDCRLLTVGSWYAMSGYGLAFTRNSKYLSMFNKRLLDLRSNGDLERLRRYW

MTGTCKPNKQEHKSSDPLALEQFLSAFLLLMAGILLAALLLLLEHVYFRYMRTHLAAS

SAGSCCALVSLSMGQSLSFHGAVVEAASRGFGAGGRGHCRSAVCAAQVWRARHERDAA

VARARQLAAALAAHGLAP

>OfuriGluR2

MRGAKAVFLILFFGHLSALPDTIRIGGLFHPEDDKQEVAFRYAV

ERVNADRAVLPRAKLLAQVETISPQDSFHASKRVCHLLRSGVAAIFGPQSAPAAAHVQ

SICDTMELPHLETRWDYRTRRESCLVNLYPHPAALSRAYVDLVRAWGWKSFTIVYENS

DGLVRLQELLKAHGPSELPVAVRQLPDSHDYRPLLKQIKNSAESHIVLDCATERIRDV

LQQAQQIGMMSDYHSYLITSLDLHSVDLEEFKYGGTNITALRLLDPERADVQRVIRDW

VYDEARKGRKLQLGHTSAKENMTFIKTETALMYDAVHLFAKALHDLDTSQQIDVRPLS

CEAEDTWPHGYSLINYMKIVEMKGLTGVIKFDHQGFRSDFTLDIIELTRDGLQKAGTW

NSSEGVNYTRSYGENQKQIVEILQNKTLIVTTILSAPYCMRKEASEKLTGNAQFEGYA

IDLIHEISKILGFNYTFKLAPDGRYGSYNRETKEWDGMIRELLEQRADVAIADLTITY

DREQVVDFTMPFMNLGISVLYRKPIKQPPNLFSFLSPLSLDVWIYMATAYLGVSVLLF

ILARFSPYEWDTPRNCLDEPPVLENQFTLLNSLWFTIGSLMQQGSDIAPKAVSTRMVA

GMWWFFTLIMISSYTANLAAFLTVERMDSPIESAEDLAKQTKIKYGALKGGSTAAFFR

DSNFSTYQRMWSFMESARPSVFTSSNKEGEERVMRGKGSYAYLMESTTIEYVVERNCD

LTQVGGMLDSKGYGIAMPPNSPYRTAISGAVLKLQEEGKLHILKTKWWKEKRGGGSCR

DDTSKSSSTANELGLGNVGGVFVVLMGGMGVACVIAVCEFVWKSRKVAVDERASLCSE

MASELRSALKCPSGAGSGGTPGNQRDGTGSPYLHYGFSTKSQLH

>OfurIR8a

MMEIPLLLLFLINLGCVLSEISLRFVFITEVHDSDLAHQIGRALRNAEEQRSGVKISDYMVQLDRENEDESYRRLCSGVSKGASLVIDLSWAPWDMAEQLCAESGLPLVRTLLGSQQLVAALDEYLESRNATDAAILLESESDVDKTLYELLGRSNVRLWVHAGLTRDSAKALKSMRPEPSFFVIVGESGFLMDTYRRAVKEKLVRRDYRWNLVLTDYSGDSIDVTQLPLPTMILHVDQVECCRLLGLREECSCPSDLKRKQLIISALVLYLSETYSKLERELPVLSTKVDCDNVLASEMNVTRDRLVRQFGEDVEINNDTLFYWDDDRSGLFLRSSFVLSVYRPDSGLETVASWSANDEYKLLPGVTLDPLKLFFRIGTSPAVPWTLPKLDPETGEPEVNEDGQPVYEGYCIDLISKLAETMEFDYEIITPKSGSFGKKLPNGSWDGVVGDLMRGETDLAVAALTMTAEREEVIDFVAPYFEQTGILIAIRKPIRKTSLFKFMTVLRTEVWLSIVAALVLTGLMIWLLDKYSPYSARNNPQAHPYPCREFTLKESFWFALTSFTPQGGGEAPKALSGRTLVAAYWLFVVLMLATFTANLAAFLTVERMQTPVSSLEQLARQSRINYTVVEGSTIHQYFINMKFAEDTLYRVWKEITLNATSDQAQYRVWDYPIREQYGHILLAINASGPVPDAKTGFEQVNEHTDADFAFIHDSAEIKYEVTRNCNLTEVGEVFAEQPYAIAVQQGSRLQEHLSRALLDLQKERFLEQLASKYWNESARQACPDADESEGITLESLGGVFIATLFGLGLAMITLAWEVFYYKRKERNKIQGIDAKVEKAAFVDPKKKDKLGVRLRKGKSKVAKLDVVGKGKGVTIGDTFKPAAEKMGVSYISVYPKGEYRP

>OfurIR21a

MTRLKCLIANIFIFVSFVLCEDVEYYPSQAALNSYSNIAKRSVNEPELEKGKVAIKWRHFNENKNETQEVKTKRAVDPIFHGHPKTREELWNERFLNKSSAFDQTPSLIKLIHNITLRYLNDCIPVILYDSQIKTRESYLFQNLLKDFPVSYVHGYIDDNNKLKEPELLIPVKQCLHFIVFLTEVKSSAKVLGKQSESKVVVVARSSQWAVQEFLASSYSRVFINLLVIGQSFKDDDDNSLEAPYILYTHKLYTDGLGASQPKVLSSWTHGKYSRDVNLFPPKMTEGYAGHRFIVAASNQPPFVFRKIKTDLDGGNPRVIWDGIEMRLLHLLAERNNFSIEILEPQEPHLGSGDAVTKEIAMGRADIGVAGMYLTVDRTKSMDMSFSHSQDCAVFITLMSTALPRYRAILGPFHWHVWVALTFTYLIGILPLAFSDKHTLRHLLHNSGEIENMFWYVFGTFTNCFTFVGKNSWSKTTKITTRLLIGWYWIFTIIITSCYTGSIIAFVTLPVFPETVDTIEQLIAGFYRVGTLDRGGWERWFFNSSDAKTNKLFKKLELVPNVESGIRNTTKAFFWPYAFLGSQAELEYIVQSNFTATKSKRAMLHISNECFVPFGVSMGFPTNSLYSAKLSGDLRRMFQSGIVDKIVDEVRWEMQRSATGKLLSAGSGSLKITSAEEKGLTLDDTQGMFLLLAAGFLMGASALVSEWMGGITRRCRIGRKKPSSANSKEELIATPELESEIKVISDCTESRLNFDTRCSSACSRDTLEGQVINVTEENIVVHETLDAATWDSRRSSSVDLDREVQEIFEKDLRRRRIVTGDIEEAAEVKRELTASNGAFGDHLN

>OfurIR25a

MKPKESRFSLKLLLLFSFVRVAIFQTTQNINVLLINEENNALAEKAFEVAKEYVRRNPSLGLAVDPVIVVGNRSDAKVFLENVCRKYNDMLSAKKTPHVVLDFTMTGVGSETIKSFTAALALPTMSSSFGQAGDLRQWRSLDANQTRFLLQVMPPADILPESIRAIVTKQDITNAAIIFDELFVMDHKYKSLLQNIPTRHVITPVKSFNKDEIKTQLRSLRELDIVNFFVVGSLRTIKNVLDAADENQYFGRKTAWFALSLDKGDITCGCKDATIVYMRPTPDAKSRDRLGKIKTTYSMNGEPEITSAFYFDLSLRTFLAVKSLLDSGKWPNDMKYITCDDYDGKNTPNRTLDLKAAFQEIKETPTYAPFYIPEDDPMNGRSYMEFNTDITAVTVKDGASIGSRVLGSWKAGLSNPLSLTDPDNMSDYSAQLVYRVVTVEQEPFIIRDDEAPKGFKGYCIDLIEEIRQIVKFDYEIVLSPDGNFGTMDENGNWNGIIKELIDKRADIGLTSLSVMAERENVVDFTVPYYDLVGITIMMKLPRTATSLFKFLTVLENDVWLSILAAYFFTSFLMWVFDKWSPYSYQNNREKYKDDEEKREFTLKECLWFCMTSLTPQGGGEAPKNLSGRLLAATWWLFGFIIIASYTANLAAFLTVSRLDTPIESLDDLSKQYKIQYAPLNGSAAMTYFERMAHIEVKFYEIWKEMSLNDSLSDVERAKLAVWDYPVSDKYSKMWQAMKEAGLPNSIEEALQRVRDSKSSSEGFAWLGDATDVRYHVLTSCDLQMVGDEFSRKPYAIAVQQGSPLKDQFNNAILQLLNKRKLEKLKENWWTNNPNAMKCEKQDDQSDGISIQNIGGVFIVIFMGIGLACITLGVEYWWYKIRKRSTIGDITQVEPAKSSRINTDFKGEGFTFRSRNLGLSNLKPKF

>OfurIR40a

MKFVPFFLFLNTAHCFFDIQDIISQTMTKLPKDFAVAIKDIAEGLPAKTITVVRGESTKIRSQDIFQLLCLLSEHNIQVINLDITTKQNKDKYYSFVKQALDISEDRTSLILCEPFECEKILTELTDNNLIHRTILYIFYWPYGTVSDKFLNTMKEAMRVAVLTNPRESVFRVYYNQATPDRLHHLSLVNWWSGRLYKSPVLPPAGKIYQDFKGRMFDVPVLHAPPWHFVRYNNDSSVNVTGGRDDKLLSLISKKLNFRYQYYDPPDRSQGSSISGNGTFKGTLGLLWKRKADFFIGDVTMTWERLQAVEFSFLTLADSGAFLTHAPAKLSETLAIIRPFRWEVWPLVCATVLVTGPALWVVIAAPSLWQRRQRDQLRLLNNCCWFTTTLFLRQSSSKEPSKTHKARLVSVLVSLGATYVIGDMYSANLTSLLARPARERPIGTLQALEEAMRDRGYELVVERHSSSLTILENGTGVYGRLARLMRRQRVQRVRSVEVGVRLVLTRRHVAILGGRETLYYDTERFGSHNFHLSEKLYTRYSAIALQIGCPYLETFNNVVMTLFEAGILAKMTTDEYKNLPEQSRRSEPVTESDKPNNDITGDSPAASQGGTTPGESTKALEPVSLRMLRGAFCLLGIGHLLAAIALGVEIQIHRRSKKFIKIVEPNGGKNVPGKRALRKANKFIRQGIGRMVRAFCRSVDRALGPGNQ

>OfurIR41a

MLQDTVLFFPVEILLTSIVNLYLNSSYCLTIVSEKSLDLSISNSFTSMVPEDGDLLVNQLLQVSEMGCSDYIVKMRDPARFMAAFERVNHLGNVRRSDRKVVFLPYEDNNITRTDLLQLLTLKETSFLANILLILPSLESGLCSIYDLATHKYTGPDDQVDQPYYMDRWNSCSLKFEKDANLFPHDMTNLHGKTVKVACFTYKPYALLDLDPLEEPLGRDGTEVRIVDEFCRWINCTIEVVRDDEHEWGELYDNQTGVGVLGNVVKDRADLGITALYSWYEEYLELDFSGSGIRTAITCVAPSPRLLASWEMPLLPFSWYMWMALGFTFVYASFALAIAKGCSTDKVFLATFGMMVTQSQADVGATWRVRSITGWMLLTGLVLDNAYGGGLASVFTVPKYEKSIDTVQDIVDRGMEWGATHDAWVFSLTLSPEPLVKQLVSLFRVSSAEDMKIKSMQRSMAFSVERLPAGYFAVGDYITKDAMLGLTLMQEDFYYEQCVVMMRKSSPYTQKVSKLIGRLHESGLMLAWETQVALKYLNYEVQLEVRLSRFHKDVDNVEPLKLRHVVGVFIIYIIGVIISTMLFILEIIHKHKKRVY

>OfurIR64a

MDINYFLNFISIAEISLVIDLLKLKEIQNVVNINCDGQKSIFHHKILNDNNIHASYWSLNSTDQNMMQMSYHKTGVILDASCSNWEQALNNFDNSMFRNEFIWLIITEDLLSTARSLTNCPIEIDSDVTVALKTNGIFMLYEVFHTNYSSGVLSIRNVGYWDTTLHIATSSRRDLQGLKMRCPVVVTDKVVHQTFEEYLSKHQVFQVDSLHKLKFVALLNYIRDMYNMSYELQRTNSWGYMRNGSFDGVVGSLQRQHADFGGSPLFFRADRAELIDYIAETWQSRQCFILRHPKHPGGYYTIYTRPLTAKVWYCILAMLIFSGVILCLMLKTKVTQSHEKSTDSSFSLALLFAWSAICQQGMTVNRSSTSVKIVVIVTFVYAVTLYQYYNATVVSTLLREPPKNIRTLEDLLQSNLKAGAENVLYTKDYFKRTTDPVALRMYHKKITPKHQYNFYSPEYGMSLVKQGGFAFHVDSVVAYRIMRKTFTEREICEAHEVLLYPPQKMGMVVRKASPYKEHFTYGIRKIYEAGLMDRLQSVWDEPKPSCVHTPDSSVFSVSIVEFSTALLALVAGNVAAILVLFAEIVLHRCEMKKRIAFTH

>OfurIR68a

MWIAVLVVLLISGSIFYGLARHYMNLQEYIKTHENRNTNEKQLDTAEKPVGLYLFGEIINSILYTYAMLLVVSLPKLPTGWSIRLLTGWYWLYCVLLVVSYRASMTAILANPAPRVTIDTLKELVESKIACGGWGMETKKFFQESSDDIQTIGQRFETINDPFVAANKVAKGVYAYYDNENFLKYIRVKRKNIDMNIQSDMVNATSNTTDVFAGEMERNLHIMSDCVVNTPISIGFHKNSPLKPLADIYLRRIVEVGLVEKWLNDAMHPIKSLETNEDEVKALMNLKKLYGAFIALAIGYSISTIGLIGELIHWHLIVKRDPKFDKYAIDLYYLSKNKKQ

>OfurIR75

MNLRDLLLVFILVYFVCTFSKSSDEISIITDLIQSSDKLTSVVAHACWKPSKQIQLASRLGNKNRPMTVRFVNKNWAGIVEPQHRERLLIVADLNCPSTKVFFKLANTTNKFSFPYRWIVIGKAVNESVAVTSNFQNIPLLPDSDVIIAQKNDKNSYILTTIYKIQIKGKWIVEQFGIWTSANGLKKFEAVKHPISTRRKNFQRAPIKMAMVILDNRTISNPYDLSDILTDTVSKSSFRQTDPIFGYLNASRTLIYSPTWGYYRNGSYGGMIADMTIGDAELAGTVLIATQDRMEVVEYLSCPTPISIKFVFRQPPLSYQNNLFLLPFKSTVWYCIGAFVLVLAFILYINALWENKKLESSEQNFEDPTVLRPNVGDIAILVISAISQQGSSTELKGTLGRIVMFILFLAFLLLYSSYSASIVALLQSSSNQIRTLSDLLNSKLELGVEDTPYNRYFFPIATEPVRRAIYQTKIAPKGTKPKFMSLEDGVKKLQKEPFAFNMNKGIGYRLVERYFHEHEKCGLQEIPYLYATKTYITCRKNSPYKEIFKIGLFRIQEHGLSDRENRLIYARKPPCQARGGSFGSVNMVDFHPILLMYLYGILLAFFFFFVEILAHKKLHPRQSQRR

>OfurIR75d

PRRLLRADPALVQITPAEAFTFAIGTICQQGFHVSPRVASVRLV

MFCTLLASLFAFTSYSAKIVAILQTPSDAIRTIDDLARSPMTLGVQETTYKRVYFAES

EDPATQRLYRRKLLPLGERAYLSVVDGVARLRSGLFAFQVEESSGYDIISKTFTESEK

CGLKLIQAFKLPMVAVPIRKHSGYRELFAVRLRWQREVGLIERSRRIWLAARARCDAA

GAGFISVRLSDVLPAVQVLLYGTLFAVVLLFIEIAVKRSTDRIKHKSKVKDLKDL

>OfurIR75p1

MMKYLELVVFALFINNGVTARDSFTTNFIKHYILNEETPTQTIF

WDLCWDTSTVVKLVNELSKAGFRSSTLMGNNAPDYLLHNVLFLVDYNCPNIADIIFTG

TRKKLFSSPFRWLVLSGTNINQTSLDLLMDCPLLPNSDVVLAERDDHGYKMSELHRSS

SSQPMIKTPRGYFNGTFIDTRPHRELFRRRRDVMGHPLTMANVIQDSNTTQYHLPREN

RLELQFDSIAKICWMDVKLAFQMLNATPRYIFSHRWGYKQNGQWSGMINDLHTGKADL

GTNCMINDKERLDVVTYTDMMAPFRVRFIFRQPPLSYVSNIFSLPFSTSVWVAIIVCA

VVSTVTLYLASKWEANLGAGPSQLDGSIGDALLLTMSAVSQQGCFLEPRRISGRVTAW

VFFAALMALYAAYSANIVVLLQAPSNSIKTLSQLAASKVTLAANDVDYNHFVFSLYKD

PVHVTIYKKVDPEKGKGQFYDINEGVERIRQGLFAFHSIVEPVYRRIEDTFLETEKCD

LAEVDFMNGFDPFVPVRKDSPYLELLRVAFKQIREAGLQSALVRRMQVPKPRCAHKVS

AFSSVGILDLKPVLLFMLYGTAASVAILVLEILTFKLSIRNAGSISRKTHK

>OfurIR75p2

MRSVLVFMIIIKVVIGKDDFTINFIKSFIENERKPTHLIYNGLC

WEKKMELNLVNELFKYGVRCSSSNLPRSTNQDHDLMFLVDLDCPDSEEVLRNASSNNL

FRFPFRWLVLSKAGLMDHRNATLWKCPVLISSDLVLAEENEKEFVLTELYRSSPDGPM

LSSPRGKYNGAFLDSRPHRELFRRRRDLMGHALVMANVIQDSNSTKEHLLKEDRLELQ

NDAAVKICWIAARHAFEMLNATRRHIFSYRWGYKVNGEWSGMIQDIKSNRADLATNCV

LYSERLDVVTFTDMVAPLRMRFVFRQPPLAYVSNIFSLPFSSNVWVAIAVCTAASTLT

LCLTSHWETKVEKNPTQLDGSISDALLLTLSAVAQQGCFVEPKRAPGRILEWFLFTAL

MALYAAYSANIVVLLQAPSNSIKSLSQLAASKITLAANDVDYSRFVLSPYKDPTHVHI

YRRIMPEGEKPNFYHIHEGVEKIRQGLFAYHSIVEPVYRRIEETFLEQEKCDLTEVDF

VNGFDAFTPVKKDSPYLEMLRVVYKQIREAGVQGALIKRFLVPKPRCVGEMSAFTSVG

LLDIQPVLVFMVYGLLLSVLIAIGEIVVHKVTKRCSIKKMRKQ

>OfurIR75p3

MTSIAKRLGEAGLNVARSLQKNRTEFYLQHALVMYDLDCPGSAG

FLKKASDEGYFKSPYRWLLLDLENSHSVNEFEELEMVVDSDVVVVRRIGGEYQFIETY

KVSTHSEVIQTHRATWRPLKYDSRNSTSKQNTSNSIEEDEVINIDTPSSMQEVKINTN

TPSTITVDKYGVIEDYRRTKVLSSRRRDLRRHTLTMVNVITDSNDTRKHLDDRLQLHQ

DSITKMSYSVVRICFEMLNASEELTFTHTCGYRDKNGNWQGIVDDLIKKKADLGTLTI

FTQERMEVVDYIAMVGSTAVRFVFREPPLSYISNIFTLPFTAAVWWAIVVCVLGCSVF

LYITSKWEASMSMHQFQLDGSWADVIILIIGAVLQQGCTLEPRYAAGRGVTLLLFLAL

TILYAAYSANIVVLLRAPSSSVRSLPDLLNSPLKLGASDFEYNRYFFKKLKDPIRKAI

YDKKIAPKGKKPNYYSMEEGVEKIRRGLFAFHMELNPGYRLIQETYREDEKCDLVEID

YINEIDPWVPGQKRSPFKDLFKINFIKIRESGIQSCIHHRLHVPRPRCSGTVATFSSV

GVADMYPALLATLYGMLLAPAVLLLEIVYHRLTELRAKSKKRKWISKLQRRN

>OfurIR75q2

MMFCIFVASFYFFNIIVRNAKTDSEVLVLSDVIRAMEKPSTVVA

TLCWQTSKKVDFYNAVTSIDRSRAATARFDDMKHAKKDYGQDQHMLFVADLKCPNISA

YFDKKKEEQYFRSPFRWILISSSENDEIVPNEIAYIDLLPDSEVIVLRQIEADTYDLH

FIYKISPNSDWRTQFYGSWNLEQRFVKTNQQLVESTALQRLDLLGFEMSICYVLTDND

SINHLTDEVNDHIDTITKVNFPTTNHLLDFLNASRKYIFAETWGYRVNGSWNGMTGYL

LREEVEIGGSPMFFTSERISVVDYIASPTPTRSKFVFQQPKLSYENNLFLLSFRTSVW

YSSTGLIFLLLLALFAVAIWEWKKNSHHIYRKQDSGILRANFVDVIILIFGAICQQGS

PVELKGSLGRVVMLILFLALMFLYTSYSANIVALLQSSSSQIRTLDDLLHSRLKFGVH

DTVFNRYYFSTATEPVRKAIYEKKVAPPGTTPRFMSMEEGVKKMRKGLFAFHMETGVG

YKFVGKYFNEGEKCGLREIQYLQVIDPWLAVRKNTPYREMFKIGMKRIQEHGLQAREN

RLLYEKRPKCSGRESNFVSVSMVDCYPALLILSYGSLVSLTLLIFEFIYHKRETIIHK

LKHSNTHSHSM

>OfurIR76b

MATGIELIISTICNATFCEPVYDNPVLESHTSSNLHQYRDLIKE

LNGKHLKIGTYNNRPFSWVERGENGTLIGNGVSFVLVDILQKRFNFTYEVVVPQKNFE

MGGAKPEDSLIGLVNNGQVDMAAAFLPKLTRFHEKVSFSYDLDEGIWVMMLKRPKESA

AGSGLLAPFNSAVWYLILVAVLSYGPCITLLTKLRSRLVPDGEKYIPMSPSFWFVYGA

FIKQGTNLAPDANTTRVLFTTWWIFIILLSAFYTANLTAFLTLSKFTLDIENPQDLYK

KNYRWVSAEGGAVQYVVSSPNEVLYYLSRMISTGRAEFRSMTSLYDYLPLVNGGAVLV

EERVGIDELMYGDYLRKAREGVAETDRCTYVIAPNFFMKKLRGFAYPKNSKLTGVFDS

VLSYVLQAGIVDFLEHRDLPSTKICPLDLQSKDRQLLNSDLLMTYMIMFTGLAAAIAV

FIGEVIVKRYIIKDSKSNKLKRKKTKFQKNPRFYNYDDSRPPPYDSIFGRSPKIKVNA

ATKTKIINGREYLVIDAANGDTRLIPVRTPSAFLYRLDR

>OfurIR87a

MCTIIFLPLFFALHVSATINENSLLTTTGNSEQTAKTAECVLKL

SAKYFVEKKALSGSIVIININSYASTTQVLLLQTIHGGIKYSVMVKDSFYPHANASHF

PEKAKNYMLILEEKSELTRNILQLNKLPTWNPLAKAIVFYQLNKTEDAEQTAIEFINE

LRHYKLFKSIIFIYSPEEKEVISYTWTPYSDTNCGGKCDSVYILDTCKDNVIHELATQ

KEMFPLDMKGCPLVTYAIVSEPYVLPPAMKLSNTSYNDAYVFQKGGEINLVKIITQFT

NMSLVMRTSDVPENWGNVYWNGTATGAYGVLRNDEVDMVIGNIEVTRTIRRWFHPTVS

YTQDEMTWCVPKAGQASTWNNLVIIFQWSTWVATFGSLFVMGLLFHYMYYRENNQKVT

KWPTNSLLMTFSMLLGWGASFEPKSATFRILIFGWLCFSVNMGISYESFLRSFLMHPR

FEKQISTETDLIQSRIPLGGREIYRSYFETNNASSFYLYRKYNSTTFAEGIKRAAKDR

NFAVVSSRRQAAYADQRLGKGKPLIYCFPESDNLYKYGVVLLARKWFPMLERFNTIIR

SVSENGLIDKWNQELLIHTANAEGASEIEPLSIQHLLGAFMFIGFMYAASIAVFIAEI

CIGVFQKWKARKNWQSLYKHIRFRNAK

>OfurIR93a

MRICLFVSLYLLRVSGEEFPSLITANASIAVVLDRQFLGEQYQATLDELKDYIKELARVELKHGGVVVHYFSWTTISLKKGFLAVFSVASCEDTWSLFSRTEEEELLLFALTEVDCPRLPTDSAITVTNVMPGEELPQILLDMRTEMAFKWKSAVILHDDTLSRDMVSRVVQSLTVQIDEGASTSPVSVSVYKMKHEINEYLRRKEITRVLSKLPVKYIGENFMAIVTTEVMTTMAEIARDLVMSHTLAQWLYVISDTDAQNGNLSSLINALYEGENVAFMYNITESNPECKNGLMCYCQEMMNAFISALDAAVQDEFDVAAQVSDEEWEAIRPNKIQRRGMLLKHMQQHISTKSSCGNCSTWRALAADTWGATYRSYGDSDQLFKEPDNATTKGVIEHVDLLQVGYWRPIDALRFEDVLFPHVEHGFRGKALPIITYHNPPWTILQVNESGSVVSCSGLIFDIVNQLAKNKNFTVKVILPSHVKNLLSNDTTADMMHSQDAALTLIAVAKGQAAIAAVAFTVLSDPPSGINYTLAVSTQPYSFMIARPRELSRALLFLLPFTTDTWLCLGLGVILMGPTLYIIHRLSPYYEAKEITRQGGLSTIHNCLWYVYGALLQQGGMYLPRADSGRLVVGTWWLVVLVVVTTYSGNLVAFLTFPKQEIPVTTIGELLENQLTYTWSIQKGSYLEMELKNSDEPKYAALLKGAELSGAGASGNLSSWKKQLIRIREQRHVIFDWKLRLSYLMRAEHMLTDTCDFALSAEEFMDEQLAMVLPAGSPYLPVINKEINRMQKAGLISKWLFAYLPKRDRCWKTSSIAQEVNNHTVNLRDMQGSFFVLFLGFFSASVVLLLEWFCNRRRRRSEDVIIKPYVE

>OfurIR1

AAATSLYHKHQDITPILRWENVIDKVDLVHPPVTSIETRYFYRIPTYGAGKFENQFLRPLSYGAWISVVIVITLCACVLLISAKLERRRSAGQYAIFSVLASMCQQFFEDNTSFTPRVSAARQLTIFVTGISCVLIYNYYTSSVVSWLLNGPPPSINSLQELLESPLELIFEDIGYTRSWLQKKSYYYNIRNIEIEDELRKKKVFNKKPNAPLLVPVEEGIKLVKAGGYAYHTDTNNANRLISQTFTQSELCELGSLQSMAKAELYPALQRNSPYKEFFVWGSIRLYERGVVKFVQRRISSPAVECEGSSPRALALGGAAPAFLLLAAGYLLATVIMLVERAIWRRKYKSKVVLKLKK

>OfurIR2

RVLVPAKLWYSTQDVLRSESADLNAGVLRIMETRMEYLDYIMPIWLFSVGFTYLAERESSSNMYVEPFTAACWWTCLGIGVTLALAQRVAARGKQEKEGAFMAVLATWLQQDAAAVPDGVAGRWTFTVMSICAMLVHAYYTSAIVSALMSTGRGGPNTLRELADSRYAIASEDHDFIRNGMFNVETDWEELEYLKKKKMTSKLFQDMEYGVQLIQQGYTAYHAEYHQLYHFLEKFSDDEICKMQHVDTIPEIMTWVSASARGQWTEIFRSTGAWLYETGLARQLLSSIQTHPPPCRAAMLAERVTFWDVAPLLGLTTIGAIASIGLLGLEIVLHRWTEKNRREKKWGSSEASLVALE

>OfurIR3

KNIGFKYYRRWDFHNLTLRAVSVILEKPDVFKPEMLLDVKYTPG

VAAMTKISAQLLKALMEKHNFRFNYTIVSRWIGEPVVNSTLTVTNSLYWRQQDISCTT

ARIFPKWLEWVDIFHPPASMLETKFYYLIPDRGVGEYENRFLTPMSPGVWWCSCGAAL

ACALVLAVSAALEGRPKP

>Eobl75q2

MKVLFLFVTGFALSYGEVYLDTKMVADVVRAMNRPSTVVSWLCWPFNKKVQLYSALAGEKIDEPVMMQFV

RNQTTNFYSREQNVVFLVDLACPHVIQFLNDSSAMTLFRTPFRWILLSNGNNTLHGDIIPQPITHLDIFP

DAEVIVTRQVNDSFILRFIYKISPESPWKTELYGHWNPEQGFQKPLKNNEAIALRRLDLDGFVIKICYVL

TDNDSIHHLTDEVNDHIDTITKVNFPTTNHLLDFLNAGRQFYFADTWGYKVNGSWNGMTGYLVREEVEIG

GSPMFFTSERFSIVDYIASPTPTRSKFVFQQPKLSYENNLFLLSFRWTVWYSTVALVFLMFLALLGVAFW

EWKKQGPDRELKESDASILRASVGDVFVLIFGAACQQGSPVELKGSLGRVVMLVLFLALMFLYTSYSANI

VALLQSSSSQIRTLEDLLHSRITFGVHDTVFNRYYFSTATEPVRKAIYEKKVAPPGVTPRFISMEEGVKR

MRKGLFAFHMETGVGYKFVGKYFKEGEKCGLKEIQYLQVIDPWLAVRKNTPYKEMFKIG

>BmorIR87a

MTTGNSDQIAKTAECVLKLSAKYFVERKALSGSIVIINVNSYSSTTQGLLLKTIHSSIKYSVMAKDSFYPHANASHFPEKAKNYMLILEERTELKRNIFQLNKLPSWNPLAKAVVFYQIKGNESAQRIAIEFINELREHKFFRSIIFINNGTESGVTSYTWRPYSENNCGGKCDSVYVLDRCKNNIVEQIEPQPEWFPSNMNGCPLTTYAIVSEPYVMPPIRKIPNAKFDDVYEFQKGGETNLVKTIAEFSNMTLIVRLSAIEENWGIIYANGTATGAYGVLRNDSVDIVFGNIEVTKQIRKWFHPTISYTQDEITWCLPKAGQASAWDNLVIIFQWTIWVATFTSLILMGLLFHYMYYREKNKKITKWPTNSLLMTFSMLLGWGSHFEPKTATFRILIFGWLCFSINMGISYESFLRSFLMHPRFEKQIATESDLIQSGIRFGGREIYRTYFESNDASSSYLHTEYSSTTFSEGIRRAALNRDFAVVSSRRQAEYQDQKLGKGASLIYCFPESDNLYKYSVVLLARKWFPMLERFNGIIRSVSENGLINKWNDEMFIHRVSLEGASTIVPLSIQHLLGAFMFIGFMYGTSAFIFLVEVFVGFVQRRAFLSAFFCGKKKRFSAVFKVKV

>BmorIR7d.3

MRTEPEDITLFLQHFHGSAVIVPLDYQNMKAVSELNKATGFKQTVLFAVSVEEFILFITTLNLDLIVPIRMVLVLTTQLTDLAMITKEAWKHDLAEIIIISKDENEEIRLTTYFPYKNGICGDYTPHSISNEKELFPEKFKNLHGCPIKVTLLNFLPYVGLQKVNGTITFIFGIDGSVFILLIKELNAIMDIVSSTDHGGMGVFVNGSWKGSFGDIVRREADIFAPAGIITQKRFSVAQMSHTYETLNIHWCAPPRREIYAWAKVLLPFLTNITPFLVLAFTVFVITIVLVKRSKLHGIKSNKNVFLQSFMIFLGQGVKFETKSSVINSFFVAWLWFCLIVRIAYQGDLVNGLQKKIYEPPFESVEQALQELDGYGGTELFREYYAGSPIADNYQVIKIGDLPRYIRDVIAGKRFLIATDILMHQYAKKFQILQEPLTHSPTCLFMRPGWPVSRRVDVIIIRAIEAGLVQKIIYDFHYTVRLRRHEKEEETGTRPLGMSTMFACYYGLILLWIFSFVIFLFEVLYYNWKHKIAYIKRKRNKLFKFHH

>BmorIR7d.1

FNLPLKLTILSLFLGIILLNMLRKTIFFNNIRRVCNITPPKRNSLFYAWLLFLGLPLEKFSSRKHFKIIILAWIWFSFVIRCAYQVTLVTSLKSITYNYNLRYDSDILKYPFGGMSSIRDYFIEDKDFYENWTSVDMQKAYKLLDEIMEEKTDFVLALNKDTILHHAAEHIGSKRIQVIDNCIVNSPIVLYFRKHSPMTDPIAKIMNAALECGFIQYSYQTNWKRQKHLLNSHYAYNLQPLTLDNFSGCFFLLIIGYGISILYFVLEVVCHKIDKTNQRIDLRVDQE

>BmorIR7d.2

MSPRNLSDASHFCEENSNEITTAALNIALHNFKWRILTYVFFNATFLCNLNIFLKTYNKGVVVGNGLVEPRIDGKIQQLVLFCDDIVGITLALNSLPNQFDETGKVIVICQSPISWKCSAEEAMRSFWSVKITNVVFLKKDVFVMAYTYMPVYNEQCEISDPIPLFGLKPCIINATKCGVFDKKLDNLNKCKIVVSTLIRRPFMIINNGIPEGADGDLLLLIMERLNATLEVIIPGDHNYWGKLDSNGTWSGSLGDVYYGAADISMTSAALTASIISYFKISIPYRSTNVVWISHPPKALSPALKLLHPFKPSTQIALGIIFFIVIACVLFVSSKKMWLLCCRRVRPTKKKPSLLFNTWMICIGVPIAHLPSTSTFLSLIVLWIWYCFLIRTFYQVWLINSLQGKFYLDGFEKIDEAIEAGYDIGGGIFLKEYFVDYPYIYNNWKETVSLNVTLHEISEGSNFIAATIYDLAKSLTNFEKINVHFLAEKVVVSPSVLFFNKNSPLVAPINELLQQLTESGFVEKISRNYFTHNVTNWKRQKHLLNSHYAY

>BmorIR41a

WINCTVQGVIGSVVEGRSDFGIAALYSWYEEWKAMDFSVSVVRSAVICLVPAPRVWELPFLPFKSIWIAVVITFVYASIGLTIAQ--GCKLLIVFGTIISQSQYIVSDSWRIRSVIGWLLVSSLILVSAYGAGLASTFTVPPSIDTVQDLLNSRMEWGFEELQRRSAFSLEQDMQMFYFDCVAMLHKNSPYTEKLSELIGRLHQSGLLWESQVSLNFNHVEGIFLIFITGTILSTLFFALE

>BmorIR68a

KMNFRPKGLLGEMVNGRADLALGNLQYTPYHLELIDLSIPYTSQCWTFLTPEALTWKTLLLPFLYMWIAVLLVLIT-GTIFYGLARYFGNILYTYGMLLVVSLPKLPTGWSIRFLTGWYWLYCILLVVSYRASMTAILANPVTIDTLVELAASKLTCGPNIAADKVAYYDNRNLHMVVNIISIGFHKNSPLKPLTDIYITRIVEVGLVWLNDAMMNLKKLYGAFIALAIGYFLSVMCLIGE

>BmorIR21a

KNNFSIEAVAKEIAKGRADIGVAGMYLTIDRTREMDVTFAHSQDCAVFITLMSTAYQAILGPFWHVWVALTLTYLFGMFPLAFSDKHSGNFWYVFGTFTGRNSWSKTDKITTRLLIEMVLDFTIIITSCYTGSIIAFVTLPETVDTIHQLLAGFYRVGVEAGIMNTAFLGSKVLHSFVPFVTIGFPNNSLYTAKLNNDLRRMVQSGIVIVDEVRLTLEDTQGMFLLLAAGFLIAATALISE

>BmorIR64a

MYNITYDGMVGSLQRHEADVGGSPIFFKTDRAYVVDYVAETWPSKQSFIFRHPKHHTVYSRPLNSVWYCVIAFLFVTASTVFFMLKFETLFLFAWSAICQQGMSLRRNSLALKVVVFVTFVCSITLYQYYNATVVSTLLKEITIRTLKDLLQSDLKVGPEYGMSLVAFHVDCEIHVYPPQMGAVLKKNSPYRNYFAIGIRRLWETGLMMKHIWDVSILEFSTPLFIVVFGVIASVVVLLCE

>BmorIR75d

DLRFRFNGTVGLLQRGRAELGVASMFMRSDRWRVLHFSSATVALLNAFMLRAPAQSNIFLLPLRGVWCCAAALLCGSAVLLAVLSCLLLEFVFSIGTVCQQGFYIMPKLSSIRMIMFLTLLTSLFTFTAYSAKIVAILQTPAAVRTVADLADSHMDVGVVEGVERMAFQVECGLMIFKLPVAVPLRKHSGYRELFGTRLRWQREVGLMVRAIWLVRLLDMLPALQMLAAGGLVAVVLLILE

>BmorIR75p

MLNATPRGMINDLHTSKADLGTNCVVSDVERLSVVTYTDMLAPFRVRFVFRQPPLANIFYLPFGRVWAAVAVCAMVYTAAIYWASKWDGDMLLTMSALSQQGCFIEPKRAPGRIMLFVLFTALMALYAAYSANIVVLLQAPNSITSLAQLAASKVTLALEDGVDMIAFHSICDLTVLSSFPFVPVKKDSPYLELLRVSFKQIRESGIQLNRRYQVGIVDLRPVLIMMIYGIISSCLILIME

>BmorIR75q1

FMNASHKGMMGDLAKGTVDFGGTIAFLTSQRLQVVDYLSSPVPINAKFVFREPPLNNLFLLPYANVWYCTAAFVVLLVIILYINAKWQPDTILVISAISQQGSSNELKGTLGRAVLFLLFLTFLFLYISYSANIVALLQSNKQIRTLQDLLNSNLNIGIEEGVKKLAFNMNCGLQIIESSPWMSCRKNSPFREIYKLGLFKLQEHGITENRLLFVNMVDVYPVILMFLYGLFLAFLILLVE

>BmorIR75q2

FLNAERKGLTGFLVNGDVEIGGSPMFFTAERTAVVDFISSPTPTRSKFVFQQPKLNNLFLLSFTAVWYSTLALISLIFTMLLSVTAWRPDTMLVFGATCQQGSTVELKGSLGRVVMLILFLTLMFLYTSYSANIVALLQSSSQIKTLEDLLHSRLKFGMEEGVKKMAFHMECGLKILQVIPWLAVRKNTPYKEMFKIGMKRIQEHGLQENRLLYVSMVDCYPALLVLSYGIIIAIALVIME

>BmorIR40a

KLNFRYRGTLGLIWKRQADFFLGDVTMTWERLQAVEFSFLTLADSGAFLTHAPAKTLAIIRPFWEVWPLVCATLFITGPALWIVIAAMGNCWFTVTLFLRQSSTKPSSTHKARLVTVLISLATYVIGDMYSANLTSLLARPPPIGTLPALEEAMREHGVEAGVRLVAVLGGHNFHLLYTRSAIAFQIGSPYLETINNVVMTLFEAGILMTTDEYVSLTMLRGAFCLLGIGHLLAGVTLLIE

>BmorIR76b

KFNFTYESLIGLTNTSKVDMIAAFIPRLVRFRKLVTFSRDLDEGVWMMMLRRPKEGSGLLAPFNFVWYVTLASVLCYGPCICFLTHVPLPFWFVYSAFIKQSTNLAPEANTTRVLFATWWLFIILLSAFYTANLTAFLTLSLDIETPEDLYKKNYRWVPDQEYLPIAVLVKCTYVAFMKKRAFVYPVGSKLKSLFDPTLAYILQSGIILEHKDLLTNSHLMMTYYIMCVGLASGLAVFVVE

>BmorIR93a

NKNFTIRYNNIPLYFRAVFIHQAGVNLKNNYYRCINYTIPVSTQPHTFIVARPREALLFLLPFTDTWLCLGFAVILMGPMLYIVHRLLANLWYIYGALLQQGGMYLPRADSGRLVIGTWWLVVLVIVTTYSGNLVAFLTFPAPVTTISELLKNSYTWSARGTLDRVLIFDWCDFAAFMEEVAMIVPAGSPYLPVINKEINRMHKAGLIWLSAYLVNLSDMQGSFFVLFLGNDKIVYMYIAE

>BmorIR8a

AMNFDYEGVVGDLTTGETDIAVAALTMTAEREEVIDFVAPYFEQGILIAIRKPIRLFKFMTVLTEVWLSIVAALVLTGFMIWLLEKYDFEFWFALTSFTPQGGGEAPKALSGRTLVAAYWLFVVLMLATFTANLAAFLTVETPVSSLEQLARQSINYTAETGFKQVAFIHDCNLTVFAEQYAIAVQQGSRLQEDISRALLELQKERFLLTSKYWITLESLGGVFIATLFGLGLAMITLAWE

>BmorIR25a

IVKFDYEGIIKELIEKRADIALTSLSVMAERENVVDFTVPYYDVGITIMMKLPRTLFKFLTVLNDVWLSILAAYFFTSFLMWVFDKWEFELWFCMTSLTPQGGGEAPKNLSGRLLAATWWLFGFIIIASYTANLAAFLTVSTPIESLDDLSKQYIQYAIEEAVQRVAWLGDCDLQVFSRKYAIAVQQGSPLKDQFNNAILQLLNRRRLLKENWWISIQNIGGVFIVIFMGIGLACITLGVE

>SlitIR21a

MAVPWFYVVFLAYHVVFGAEVIIEYYPSQSVLDMNNKVVRKREVNNTDDPNLNINGSDSYWRHFNNDTDDGDIHKRALDPVFYGHPKTREELWNERFLNETTSFDQTPSLVKLLHNITLTYLKDCTPVILYDNQVMSKESYLVQNLLKGFPTTFIHGYINDDGELVEPELIHATIECQNYILFLSDIKISAKILGKQPENKIIIIARSSQWAVQEFLASVTSRNFVNLLVVGQSFKEGDDAKLESPYILYTHKLYTDGLGASQPLVLNSWTHGKFSREVNLFPLKMTEGYAGHRFVVAAANQPPFVFRRIKSDLDGGNPRVVWDGIELRLIKLLAERNNFSIEIIEPREPNLGPGDAVAKEIVTGRADIAIAGMYLTNDRIREMDMSLAHSHDCAVFVTLMSTALPRYRAILGPFHWHVWVALTFTYLFGMFPLAFSDKHTLRHLIHNSGEIENMFWYVFGTFTNCFTFLGKNSWSKTNKITTRLLIGWYWIFTIITTSCYTGSIIAFVTLPVFPETVDTIKQLLAGFYRVGTLDRGGWEKWFLNSSDPQTNKLLRKLELVPSVEAGIRNTTKAFFWPHAFLGSKAELEYIVQANFTATKSKRAVLHISNKCFVPFGITIGFPNNSVYSAKMNLDISKMIQSGLIDKITNEVRFEMQRSPTGSLLAAGSGTINIPSAEEKGLTLEDTQGMFLLLAAGFTIAATALVSEWMGGFTRRCRFQKKSETPTSANSRDNLIITPKTDVDSEIRIIEDTERRLHFEERPSSSVSVDTLEGQVIHVTESSIDVHNTFNVDRFDSRRSSSLDLDREVREIFEKDQKRRRIFSRDMESLDENGSTVSRAAFGDSVKNDI

>SlitIR25a

MNGEPEITSAFYFDLSLRTFLTIKSLLDSGKWPNDMKYITCDDYDGKNTPNRTLDLKTAFQEIKETPTYAPFYIPQDDPMNGRSYMEFSTDLLAITVKDGASISSHSLGSWKAGLSSNLTLTDPNNMSNYSAQLVYRIVTVEQKPFIIRDDQAPKGFKGYCIDLIEEIRAIVKFDYEISLAPDGNFGTMDENGNWDGIIKELVDKKADIGLSSLSVMAERENVVDFTVPYYDLVGITIMMKLPRTPTSLFKFLTVLENDVWLSILAAYFFTSFLMWVFDKWSPYSYQNNREKYKEDEEKREFTLKECLWFCMTSLTPQGGGEAPKNLSGRLLAATWWLFGFIIIASYTANLAAFLTVSRLDTPIESLDDLSKQYNIPSATVSMDLRLMTCFQRGGYLGGSFLKFGGSWAKRPFKGGGTAQNLRYGIIPVRDKYIKFWRAMEEAVLPLPFLGKLYRGVRDSKSFSEGFAWLGDATDVKYHVMTSCDLQSVGDEFSRKPYAIAVQQVSPLKDHFNNAILQLLNKRKLEKLKEIWWNNNPESMKCEKQDDQSDGISIQNIGGVFIVIFMGIGLACVTLGVEYWWYKWRKRPAVGDVTQVEPAKLTRNNVDKQGEGFNFRGRNLGLNFKPKF

>SlitIR40a

MRRQRVQRVRNVEVGVRLVLSHKRVAVLGGRETLYYDTERFGSHNFHLSEKLYTRYSAIALQIGCPYLETFNNVVMTLFEAGIVAKMTTDEYKNLPEHARRSDPVTESDKQGGEVMGESAATSSQTPQGESTKGLQPVSLRMLRGAFCLLGIGHLLAAISLAVEIQLHRRSKRRRKPEHNEHRKAQKLLVLGKSVMLFKRGCKKVCTSVFTSIDKALGSDNKDYFDKMAPDLGNLFLIVVCLLTSRVFLVHTGVLCRIFSSLQPVLYLIEQNSYFAVSIKIFTRVSENARLPVCTRGAREGNSNFVFKCFFYLSRLKVVATVIVK

>SlitIR41a

MLLPTISLPLEILLNTIITQYLDSSYCVTVFSDKPLSPIISTSFIYLIPDEENLVEQIYNVSERGCSDYIVRMRDPQNFMTAFERVVHIGNVRRSDRKIIILPYNEEYNDNNDENLPSLIFSMKGSEYLANMLMVVNHNSSNSDCKEFDLITHQYVGPDDVSNLPKYLDRWDSCSQQFENNANLFPHDMTNLFGKTLRVACFTYKPYALLDIDTAIEPLGRDGVEIRIVDEFCRWVNCTVEVVREDVDQWGEIYKNESGGIGVIGSVVKDRADLGITALYSWYEEYRVMDFSVAGVRTAITCIAPAPRLLSSWEMPLMPFTWYMWLAVVFTYFICLNWDFNSTGIWFIIVSILNAFGMMIGQSQYEGKPSWKIRSVTGWLLIAGLILSSAYGAGLASTFTVPRYEPSIDTVQDIVDRKMEWGATHDAWIFSLTLSTEPLVKELVSQFRIYSFDELKRKSFTRSMAYSIEKLPAGNFAIGEYVTQEAILDMMVMLEDFYYEQCVVMMRKSSPYTEKVSQLIGRLHQSGLLLAWETQVALKHLNYKVQVEVRLSRSKNDVGTTKALNLGNVMGIFIVYAIGLMLSIATFLGELYVHHHKQKKERIHVD

>SlitIR68a

MLRILIIFVITSTYNSRLQVESFPIIKDLHERRDLEFVLIDLLNVLTRDYEVTCIAIICDEVYLNVFGGPLFKRTASVPYVMTVVEDYEDLLSPNFVTLESLRAARKEGCNVYVILLANGLQASRLLRFGDRHRILDTRAKYIMLHDFRLFRSELHYIWRRIVNIIFVKYHKKILGVSKSRPWFELSTVPFPNPIKGVFVPRRVDIWKNENFYYKRPLFADKTSNLNGEVLNVVYLDHVPSVVVVKNNGSNKIGGVEVEILHTLAEKMNFKPKPYQAINAELHKWGQKQPNGSFSGLLGEMVNGRADVALGNLQYTPYHLELTDLSIPYTSQCWTFLTPEALTDNSWKTLILPFKLYMWIAVLLVLLITGTIFYGLAKNHMNLQEYKKLRPIQTKDDEGIDAKPGLYLFGEIINSILYTYGMLLVVSLPRLPTGWSIRLLTGWYWLHCILLVVSYRASMTAILANPAPRVTIDTLRELVDSKVTCGGWGTQSKKFFQESLDENTQKIGDKFETIDDPMKAASKVAQGVYAYYANSDFLKYISVTRKDALKGSKGNSTNTTDIAPKIDSQRNLHIMSDCVVNIPISIGFHKNSPLKPLADVYMWRVVEVGLVEKWLNDVMHPIHSLETNEDELKALMNLKKLYGAFIALAIGYTLSALCLAGELTHWHFIVKRDPNFDKYALHLYYRNKNKKDY

>SlitIR75d

MELISFILSYFITKDLSMMTAFICWPSEQALELQRSARVAGVRLTVVSELRHSAPMTTSGYFREAMLLDLNCPDTHFVLEKASRSRVLNKRHSWLLLHNSSAEPALVEETLYAYEILPDADVVWSSPNSLVDVYKTKPNQPLLQVQLGLSRNSSHQELLSLWGALPTAVTRRRDLRNVSLKGISVVTEPDNFKGWADLRNRQIDTFPKFTYPLMMLLAQDLHFRFDLRQVDFYGVSHNGSFDGLVGHLQRREAEVGLASLFMRHDRMQVADFFSETCVLACAFIFRQPSRSAVSNVFLAPFSAGVWGASACVAASAALLLVALRRLRQHTRASTDLQLFTLLEAVTFALGSMCQQGFHRTPPVTSVRLVMFSTLLTSLFVFTAYSAKIVAILQTPSTALQTIDDLVRSPMTIGVQDTTYKTVYFLESPEKSTQQLYRHKILPQGERAYHSVVDGIARVRTGFFAFQVEKSSGYDIIKQTFTEREKCSLSEIEAFKPPLVAVPMKKHSGYRELFASRLRWQREVGLMDRARHVWLVS

>SlitIR75p

MTGISIVLFFLVTQCSVLIQSKDMENINFIKLFILNDQKPTHLIYGGLCWKKELINKLVVEMSNIGVRTSASFKPRSKYQDHAIMYLTDLDCAQSRTVLSYASSKELFQFTYRWLILVTSPQLQQSKISLLENGPVLVDSDVVLAERVGNMFKMTELHRPGPNGSMISTPRGYYNGSVVDVRAHRELYRRRRNMRGHAITMSNVIQDSNTTRLHLPREDRLKLQYDSITKACWSAAKIGFEMINATPRYIFSYRYGYKVNGQWSGMIADLYANKADMGTNCVIFRDRFDVVTYTDLVAPMRMLFIFRQPPLAYVANVFYLPFSTRVWVTIAVCTAIATVTLFLASKVEIVITKTTTQQQLDGGICDVLLLTMSAVTQQGCYIEPRRAPGRMMVFVLFTALMALYAAYSANIVVLLQAPSDSIRSLPQLANAKITLAANDVDYNHFVFNQSREPLYISIRDRVFPENGKAKLYSLADGVERIRQGLFALHSVAEPVYRQIEATFLESEKCDIATVDYLVTFDSFTPVRKGSPYLELIRVVHKQIRESGIQSAIRRRYLVSKPHCTTKMSSFSSVGLMDMRPVLILMLYGVAVSVIIVFGEIIVHKLINRYYKQKSKVQMVKTIHY

>SlitIR75q.2

MKKITFIFIAFFFSSINGKTEQANMIVDIIQAANRPSSVIGKLCWTPTKIIHLSSALVKENIQFSANADLTNDYMQFYDEEQQIVFLADLNCPDIEDYFQMNSTRTFFRAPFRWILFGDTGSVNEDNIVPEAIANVDVLIDSEVLVVRSIDDAYEMHFIYRISPNNTWNTEYYGTWDSENRFQKSNRFVEPTSLRRLDINEYEISICYVLTNNNSINHLSDGLDDHVDYEFTKVNFPTTNHLLDFLNAGRKYIFAETWGYKVNGTWNGMTGYLVRGEVEIGGSPMFFTFERVSIVDYISSPTPTRSKFVFQQPKLSYENNLFLLPFNTTVWYCTVGLVFVIYLVLLLVAKWEWKKTKHTIETREKDAGVLRANVVDVIILIFGAACQQGSPSELNGRWLGRIVMLVLFLALMFLYTSYSANIVALLQSSSSHIKTLDDLLHSRIKFGVHDTVFNRYYFSTATEPVRKAIYEKKVAPPGTTPRFMTMDEGVLQMRKGLFAFHMETGVGYKFVGKYFNEGEKCGLREIQYLQVIDPWLAVRKDTPFREMFKIGTKRIQEHGLQYRENRLMYEKRPKCSGGGSNFVSVSMVDCYPAILILSYGTIVALFFLGLEILVHKREKVLLKLKCLKRKLRLMTEVILRRNALDVEPLVVEL

>SlitIR75q.1

MKYLTFVLNIICLDYCVTFNTNTELQIIVDVAKSYDKPTSVIAKMCWETSKRSKYAPLEAKLAKMLANLDRPMNIRYLRQNETIDNDNYPNNHLLFILNRTCDDANAFLRWASANHKFRKSHRWLILGKSLIIKDETFNVSPEFDDIRISVDSEVIIIGKINNSEEVSLHTFYKLKPHTKWIIEDYGTWSFDTGFTKSTTRIESNVIRRKDFMGETLITSVAISDNRTKTDLLGLGNIFIDTPAKSSFRTIVPLFDFLNATKVVKSLILGVLINGSGMEGLVILSGRSRLVWNCNVHNERTYDNFRIFDPSYTHHTEVCLQTTALVVPEQFIPSAIFHWCLAVHWCIHCYINCHIVRQHDMGFKEIQRLNKQKIDQTCLPPTWSDITIFVLSAISQQGSSNELKGTLGRLVMFLVFLAFVFLYTSYSANIVVLLQSTSNQIRTLSDLLHSRLELGLEHAPFNKFYFSSAYTADDPIKKALVDTKIAPKGVLTNVMNIEQGVRIMQKKPFAFNMNTGXRVQNCFSNLKNHGKVSGLQEIEYIPNSNPWLCSRRLSPYGELFKVGYIRIQEHGLSDRENRLIYAKKPACTVMGGSFGSVNMVDLHPVCLVLLYGMILAFLLLGVEILVHRKQMKMRNQARVECNVKLCSKVASCEIRFWFCIAYKMCSNDAFIAKILLIFFTVNRKLLMASPPLTSVTGDPMAFNVLIDDFH

>SlitIR76b

MAGIELIISSICNATFCEVPYNETYQAPDSLAEKDTNFMSLMKEVNGKNIKVTTYNNTPLSSTELENGTVVGKGVAFTILNILRKKFNFTYEVVLPTKNFELGAKISDDSIIGLLNSSKVDMAVAFIPTLLPYREWVSFSIDLDEGVWVMMLKRPKESAAGSGLLAPFNDLVWYLVLAAVLTFGPCITFFTRVRSKLITDDEGVLPLKPSFWFVYSAFLKQGTNLSPEAHTTRVLFVTWWLFMILLSAFYTANLTAFLTLSKFTLAIETPKDLYQKNNRWVASAGSSVEHVVKTEGEDLYFLNAMISSGKARFLSVAGDKDFLDFVKKGAVLVKEQTVVDHLMYNDYISKKDVEESEKCTYVVAPSAFMKKQRAFAYPVGSKLKGLFDPVLTQIFQAGILDFLKRSDLPSTKICPLDLQSKDRKLRNSDLIMRYMVMVAGSATAVAVFGAEVFIKRYVSGKLNKNKKSKRKKSKTGKSLKSHDDSRPPPYDSLFGKNPKFNVETTRMKMINGREYYVFETSNGDKKLIPARAPSSFLYRSDK

>SlitIR1

MLAQKLTKENVRVSVRRLNGDNVDVVRVAHQTTVPVGVLVDGHCDQTQTLMNQASFNKLFDAVHSWLILTDFEDDNCTEYVMQTFQWLNLSVNADVAVVANRGDSFAIIDVYNFGKIQGNHLETALLGTWQPDQGLEIILKGYKYYNRWDFHNLTLRAISVIVDQPKVFYPEMLSEMTYTSGVAAMTKITTQMLNTIKERHNFRFNYSIASRWIGSPERNSTMAVTNTLFWEEQDLSSTCARIFPKWLNWVDIYHPPTTNLQTKFYYSLIPETGCRGQYEGTGFLTLGCSHGGLGGCVLLSPGIPRTRGSWQPRLRMEKQTQARDCMPSFRVFAAGLSTRLGRRRSGCWSQTLSSQGRRTNPASDRTDEHAAVQLTTPAVWCPGCWTLRAPFPSANLEGLINSDFELVLEDIGYTRGWLDNPGFFYYSGFKNVKEDELRDKKVTKAKRTVSVLQNVNKGVELLRTGKYAFHTEPYTASQVISKTYEDKELCNLGALQMMLPAHVYIMAQKKSPYKEFFDWSLLRLLERGHVKAIRARFAGTMSACSGAQPRALALGQAAPAFLMLASFAVLSCFILVLEVLWKRVQLKNRGQ

>SlitIR87a

MTLITRTSEILENSGVVYRNGTATGAFEVLRNETADLVIGNVEVTRVLRKWFHPTVNYLQDEMTFCLPKAQQAPTWDNLVIIFQWSTWVATFLSLVIMGLVFHFFYYREHSNATKWPTNSLLMTFSMLLGWGATFEPKSPTFRILIFAWLCFSINMGISYESFLRSFLMHPRFEKQISSEADLIQSGIPLGGREIYRSYFETNNASSFYLYRKYNSTTFSEGVRRAALQRNFAVVSSRRQAVYQDQKLGKGAPLIYCFPESNNMYKYGVAILTRRWFPMLERFNNIIRSVTENGLIDKWMNELLIHTVSSEEASTIVPLSIQNLLGAFMFIGFMYACSIVIFLGEVIMGVIGKRRRVKKFKCKCSW

>DmelIR8a

MELPLLVLLLALRFAGSEVLKITFWIEPVQRAEFDTDIAMVLKELDALRLDVKVDDTTLTLTRSEDGLDMQRFCEILSTVGASAVIDLTYSHWEEGYNLVRSLGIGYVRLERIMRPFLDMFGDFMRQKRANNVAMVFMNARDAVEAMQQMLVGYPFRTLIMDASQTDPGQHFLERIRSLRPAPTYIALFARAAAMNGIFEKVQKADLFQRPLEWHFVFLDTRDRVFKYRRQAELCTRFTLNPRAICRSMPMPDLYCGSGFTMQRAMLLNVLRSLINAAQVSPGYPLAIYQDCNATASSSEVSDPLEKDDYNWLDMVHWSNFLAYAPPLPHIQDQFQSPVPGLTFAVNISAGYYSSEHEAKTDLAAWSSVGEMRLLNETISPARRFFRIGTAESIPWSYLRREEGTGELIRDRSGLPIWEGYCIDFIIRLSQKLNFEFEIVAPEVGHMGELNELGEWDGVVGDLVRGETDFAIAALKMYSEREEVIDFLPPYYEQTGISIAIRKPVRRTSLFKFMTVLRLEVWLSIVAALVGTAIMIWFMDKYSPYSSRNNRQAYPYACREFTLRESFWFALTSFTPQGGGEAPKAISGRMLVAAYWLFVVLMLATFTANLAAFLTVERMQTPVQSLEQLARQSRINYTVVKDSDTHQYFVNMKFAEDTLYRMWKELALNASKDFKKFRIWDYPIKEQYGHILLAINSSQPVADAKEGFANVDAHENADYAFIHDSAEIKYEITRNCNLTEVGEVFAEQPYAVAVQQGSHLGDELSYAILELQKDRFFEELKAKYWNQSNLPNCPLSEDQEGITLESLGGVFIATLFGLVLAMMTLGMEVLYYKKKQNALEITQVRPVNDSSGSGGNSSTAPPTATSTTKQAWHIPVLEAEEKPAKVSPPPSFETATFRGKKLPARITLGDGKFKPRHGLYARRNLGASDSHSGYME

>DmelIR25a

MILMNPKTSKILWLLGFLSLLSSFSLEIAAQTTQNINVLFINEVDNEPAAKAVEVVLTYLKKNIRYGLSVQLDSIEANKSDAKVLLEAICNKYATSIEKKQTPHLILDTTKSGIASETVKSFTQALGLPTISASYGQQGDLRQWRDLDEAKQKYLLQVMPPADIIPEAIRSIVIHMNITNAAILYDDSFVMDHKYKSLLQNIQTRHVITAIAKDGKREREEQIEKLRNLDINNFFILGTLQSIRMVLESVKPAYFERNFAWHAITQNEGEISSQRDNATIMFMKPMAYTQYRDRLGLLRTTYNLNEEPQLSSAFYFDLALRSFLTIKEMLQSGAWPKDMEYLNCDDFQGGNTPQRNLDLRDYFTKITEPTSYGTFDLVTQSTQPFNGHSFMKFEMDINVLQIRGGSSVNSKSIGKWISGLNSELIVKDEEQMKNLTADTVYRIFTVVQAPFIMRDETAPKGYKGYCIDLINEIAAIVHFDYTIQEVEDGKFGNMDENGQWNGIVKKLMDKQADIGLGSMSVMAEREIVIDFTVPYYDLVGITIMMQRPSSPSSLFKFLTVLETNVWLCILAAYFFTSFLMWIFDRWSPYSYQNNREKYKDDEEKREFNLKECLWFCMTSLTPQGGGEAPKNLSGRLVAATWWLFGFIIIASYTANLAAFLTVSRLDTPVESLDDLAKQYKILYAPLNGSSAMTYFERMSNIEQMFYEIWKDLSLNDSLTAVERSKLAVWDYPVSDKYTKMWQAMQEAKLPATLDEAVARVRNSTAATGFAFLGDATDIRYLQLTNCDLQVVGEEFSRKPYAIAVQQGSHLKDQFNNAILTLLNKRQLEKLKEKWWKNDEALAKCDKPEDQSDGISIQNIGGVFIVIFVGIGMACITLVFEYWWYRYRKNPRIIDVAEANAERSNAADHPGKLVDGVILGHSGEKFEKSKAALRPRFNQYPATFKPRF

>DmelIR21a

MSYYWVALVLFTAQAFSIEGDRSASYQEKCISRRLINHYQLNKEIFGVGMCDGNNENEFRQKRRIVPTFQGNPRPRGELLASKFHVNSYNFEQTNSLVGLVNKIAQEYLNKCPPVIYYDSFVEKSDGLILENLFKTIPITFYHGEINADYEAKNKRFTSHIDCNCKSYILFLSDPLMTRKILGPQTESRVVLVSRSTQWRLRDFLSSELSSNIVNLLVIGESLMADPMRERPYVLYTHKLYADGLGSNTPVVLTSWIKGALSRPHINLFPSKFQFGFAGHRFQISAANQPPFIFRIRTLDSSGMGQLRWDGVEFRLLTMISKRLNFSIDITETPTRSNTRGVVDTIQEQIIERTVDIGMSGIYITQERLMDSAMSVGHSPDCAAFITLASKALPKYRAIMGPFQWPVWVALICVYLGGIFPIVFTDRLTLSHLMGNWGEVENMFWYVFGMFTNAFSFTGKYSWSNTRKNSTRLLIGAYWLFTIIITSCYTGSIIAFVTLPAFPDTVDSVLDLLGLFFRVGTLNNGGWETWFQNSTHIPTSRLYKKMEFVGSVDEGIGNVTQSFFWNYAFLGSKAQLEYLVQSNFSDENISRRSALHLSEECFALFQIGFLFPRESVYKIKIDSMILLAQQSGLIAKINNEVSWVMQRSSSGRLLQASSSNSLREIIQEERQLTTADTEGMFLLMALGYFLGATALVSEIVGGITNKCRQIIKRSRKSAASSWSSASSGSMLRTNAEQLSHDKRKANRREAAEVAQKMSFGMRELNLTRATLREIYGSYGAPETDHGQLDIVHTEFPNSSAKLNNIEDEESREALESLQRLDEFMDQMDNDGNPSSHTFRIDN

>DmelIR31a

MNLLISMFILILAAGEGEIIPSMEESVVTNFVKSLVKTKQAIVFSCLFKDFKEISLALMRINQFVSVVNLNQSYSLTSILTRENYARTSVMVNARCSGSSELLFEASENRYFNKTYQWFLWGVDLEVQSLFPLNLNYVGPNAQITYVNETADGYAYWDIHSKGRHLKSNLEINLIATLINDTLNIARDIFHLQSIDFRGQFNGLTLRGASVIDKEDIISNEQIESILSRPTKDAGVAAFIKYHYELLGLLRERFNFTVNFRNSRGWAGRLGNTTFRLGLLGIVMRNEADIAASGAFNRINRFAEFDTIHQSWKFETAFLYRYTSDLDTHGKSGNFLSPFSDRVWLFCLLTLGAFSIIWVLFEIIDYKILRIRVNSQKLEHLNQKSSVICIKTTCIERILQTFGACCQQGLDPNPVDRSVRFLVMTLFLFSLVMYNYYTSSVVGGLLSSSDQGPSTVDEITASPLKISFEDIGYYKVLFRESQNRSITRLIEKKLSSSRSLNELPIFSHIEDAVPYLKAGGFAFHCEVVDAYPVISEYFDANEICDLREVSGLMEVEILNWILHKNSQYTEIFKTAMCNAQEKGFVERILRRRQIKKPACQSLYTVYPVSLSGVLPGFVILICKSINKFS

>DmelIR40a

MHKFLALGLLPYLLGLLNSTRLTFIGNDESDTAIALTQIVRGLQQSSLAILALPSLALSDGVCQKERNVYLDDFLQRLHRSNYKSVVFSQTELFFQHIEENLQGANECISLILDEPNQLLNSLHDRHLGHRLSLFIFYWGARWPPSSRVIRFREPLRVVVVTRPRKKAFRIYYNQARPCSDSQLQLVNWYDGDNLGLQRIPLLPTALSVYANFKGRTFRVPVFHSPPWFWVTYCNNSFEEDEEFNSLDSIEKRKVRVTGGRDHRLLMLLSKHMNFRFKYIEAPGRTQGSMRSEDGKDSNDSFTGGIGLLQSGQQADFFLGDVGLSWERRKAIEFSFFTLADSGAFATHAPRRLNEALAIMRPFKQDIWPHLILTIIFSGPIFYGIIALPYIWRRRWANSDVEHLGELYIHMTYLKEITPRLLKLKPRTVLSAHQMPHQLFQKCIWFTLRLFLKQSCNELHNGYRAKFLTIVYWIAATYVLADVYSAQLTSQFARPAREPPINTLQRLQAAMIHDGYRLYVEKESSSLEMLENGTELFRQLYALMRQQVINDPQGFFIDSVEAGIKLIAEGGEDKAVLGGRETLFFNVQQYGSNNFQLSQKLYTRYSAVAVQIGCPFLGSLNNVLMQLFESGILDKMTAAEYAKQYQEVEATRIYKGSVQAKNSEAYSRTESYDSTVISPLNLRMLQGAFIALGVGSLAAAALNNTINVRSLNSRDKFICGGPVKIWYYLVLLLWYYFNRGLVGIYQLWHKTSIRNTGKGMPFLGE

>DmelIR64a

MHWWLLVFLPLSCQGLPEHELLELELDYGLAEPQRTSLLQSSLILQFSQDYKHIPRITYFTCQKPHLQTPNQIPNAAEHRDAFAAKNFQLIKSLYESELFVRIVLLDVLAQSPTSGRPNRPGNGPTGGFSQTPSQAQSNSEWLEGVLRMEALRQIAVVDLACGAVSRRFLELASAKMLYSEKFHWLLIEDFAWHGRTQTAEGSGKRDDGEMEEEEPPGQQIQATDDEDLPSIESFLGGMNLYMNTELTLAKRMSEAAHYTLFDVWNPGLNYGGHVNLTEIGSFTPTEGIQLHTWFRTTSTVRRRMDMQHARVRCMVVVTNKNMTGTLMYYLTHTMSGHIDTMNRFNFNLLMAVRDMFNWTFVLSRTTSWGYVKNGRFDGMIGALIRNETDIGGAPIFYWLERHKWIDVAGRSWSSRPCFIFRHPRSTQKDRIVFLQPFTNDVWILIVGCGVLTVFILWFLTTIEWKLVPHDGSALIKPKGGAPPRHHYQQQQQQEQVEAPVRPITAVSVVVSKEKVEEKQEEYEDSTPIDAGTLWQRCYQKLNKYIKDRKAKQKKAPERVGLFLESVLFFVGIICQQGLGFSTSFVSGRCIVITSLLFSFCIYQFYSASIVGTLLMEKPKTIKTLSDLVHSSLKVGMEDILYNRDYFLHTKDPVSMELYAKKITSVPTTKENEADEDEPVDPNPVSTDPAKSYRDIVHSHETGAHAKDNAASNWLDPETGLLRVKHERFAFHVDVAAAYKIIAETFSEQDICDLTEVSMFPPQKTVSIMQKNSPMRKVISYGLRRVTETGILTYHFNVWHSRKPPCVKKIETSDLHVDMDTVSSALLILLFSYAITLMILGTEILYSKWHNRIQLKWVGAT

>DmelIR75a

MQLVQLANFVLDNLVQSRIGFIVLFHCWQSDESLKFAQQFMKPIHPILVYHQFVQMRGVLNWSHLELSYMGHTQPTLAIYVDIKCDQTQDLLEEASREQIYNQHYHWLLVGNQSKLEFYDLFGLFNISIDADVSYVKEQIQDNNDSVAYAVHDVYNNGKIIGGQLNVTGSHEMSCDPFVCRRTRHLSSLQKRSKYGNREQLTDVVLRVATVVTQRPLTLSDDELIRFLSQENDTHIDSLARFGFHLTLILRDLLHCKMKFIFSDSWSKSDVVGGSVGAVVDQTADLTATPSLATEGRLKYLSAIIETGFFRSVCIFRTPHNAGLRGDVFLQPFSPLVWYLFGGVLSLIGVLLWITFYMECKRMQKRWRLDYLPSLLSTFLISFGAACIQSSSLIPRSAGGRLIYFALFLISFIMYNYYTSVVVSSLLSSPVKSKIKTMRQLAESSLTVGLEPLPFTKSYLNYSRLPEIHLFIKRKIESQTQNPELWLPAEQGVLRVRDNPGYVYVFETSSGYAYVERYFTAQEICDLNEVLFRPEQLFYTHLHRNSTYKELFRLRFLRILETGVYRKQRSYWVHMKLHCVAQNFVITVGMEYVAPLLLMLICADILVVVILLVELAWKRFFTRHLTFHP

>DmelIR75b

MNFSVLESHFKEAQIFVDADVTYVTHDPFSKNFLLYDVYNKGRQLGGELNITADREIFCNKTNCRVERYLSELYTRSALQHRKSFTGLTMRATAVVTALPLNVSIKEIFDFMNSKYRIQLDTYARLGYQARQPLRDMLDCKFKYIFRDRWSDGNATGGMIGDLILDKADLAIAPFIYSFDRALFLQPITKFSVFREICMFRNPRSVSAGLSATEFLQPFSGGVWLTFALLLLLAGCLLWVTFILERRKQWKPSLLTSCLLSFGAGCIQGAWLTPRSMGGRMAFFALMVTSYLMYNYYTSIVVSKLLGQPIKSNIRTLQQLADSNLDVGIEPTVYTRIYVETSEEPDVRDLYRKKVLGSKRSPDKIWIPTEAGVLSVRDQEGFVYITGVATGYEFVRKHFLAHQICELNEIPLRDASHTHTVLAKRSPYAELIKLSELRMLETGVHFKHERSWMETKLHCYQHNHTVAVGLEYAAPLFIILLGAIILCMGILGLEVIWHRHCTLH

>DmelIR75c

MTSWPLYRLIVFNLLEINLSNLMVFHCWSIKEAFPLVEMLNQNGIFSQYIDVQNPDNLANVHKEYLDSDLVSLNADVTYVSREDEERFILHDVYNKGSHLGGKLNITVDQTLQCNRSHCQVKEYLSELHLRPRLQHRMDLSSVTFRLAALVSVLPINSSEEELLEFLNSDRDSHMDSISRIGNRLIMHTQEILGFNVQDAFGGAIGMLTNESAELCTTPFVPSWNRLHYLHPMTEQAQFRAVCMFRTPHNAGIKAAVFLEPFMPSVWFAFAGLLIFAGVLLWMIFHLERHWMQRCLDFIPSLLSSCLISFGAACIQGSYLMPKSAGGRLAFIAVMLTSFLMYNYYTSIVVSTLLGSPVRSNIRTIQQLADSSLDVGFDTVPFTKTYLVSSPRPDIRSLYKQKVESKRDPNSVWLSPEEGVIRVRDQPGFVYTSEASFMYHFVEKHYLPREISDLNEIILRPESAVYGMVHLNSTYRQLLTQLQVRMLETGITSKQSRFFSKTKLHTFSNSFVIQVGMEYAAPLFISLLVAYFLALLILILEICWARYAKKKFSTIIPQNQ

>DmelIR75d

MKVQVAHWLPLIFFLLVSGTPRVAGSWRSEYSRQDPDPKTRWGNQLPDMLVAYYRHHGVHSLMLVVCHTDIADFRLWKLWQHFNLNNFYVQVSTESSLRDLQHVDALDEHKDAPPPKSFHANNSTHWETSFLLPALPYKMGILLLEFSSECALNLLRWSAASEHNYFTTNRFWLLLTEDPGDIDLLEDPEIFIPPDSELRVLHYENVGNFSCSLIDLYKVAAWKPLKRTLVGHNIRNSRHVIHALQHFGSAITYRQDLEGIVFNSAIVIAFPDLFTNIEDLSLRHIDTISKVNHRLMLELANRLNMSYNTYQTVNYGWRQPNGSFDGLMGRFQRYELDLAQLAIFMRLDRIALVDFVAETYRVRAGIMFRQPPLSAVANIFAMPFENDVWVSILMLLIITTVVLVLELFFSPHNHDMSYMDTLNFVWGAMCQQGFYVEVRNRSARIIVFTTFVAALFLFTSFSANIVALLQSPSDAIQSLSDLGQSPLEIGVQDTQYNKIYFTESTDPVTKNLYHKKIASKGENIYMRPLLGMEKMRTGLFAYQVELQAGYQIVSDTFSEPEKCGLMELEPFQLPMLAIPTRKNFPYKELIRRQLRWQREVSLVNREERKWIPQKPKCEGGVGGFVSIGITECRYALGIFGCGAAVSFVLFLFEFIFRHFKQVYRIIKGYREVQR

>DmelIR76a

MENLLVESYYFSTVLSFFAQQFFADSHATCIFWHPAFDFRLETVHPMPLIIMDWHRWANRSDQDVYDYKIKEDEFEGKGIPYNDWTLRLTVAIERSHCETFIAFQEQIPEFARYFYHASIYSIWRSLRNRFMFVYTKEFEDKKDSYLSGYIFQDQPNILVITSQYLNSSTFEIKTNRFVGPRNFNKNPEPVEFYILQRFDAKGTKATWETQSAMSSKMRNLKGREVVIGIFDYKPFMLLDYEKPPLYYDRFMNTTDVTIDGTDIQLMLIFCELYNCTIQVDTSEPYDWGDIYLNASGYGLVGMILDRRNDYGVGGMYLWYEAYEYMDMTHFLGRSGVTCLVPAPNRLISWTLLLRPFQFVLWMCVMLCLLLESLALGITRRWEHSSVAAGNSWISSLRFGCISTLKLFVNQSTNYVTSSYALRTVLVASYMIDIILTTVYSGGLAAILTLPTLEEAADSRQRLFDHKLIWTGTSQAWITTIDERSADPVLLGLMEHYRVYDANLISAFSHTEQMGFVVERLQFGHLGNTELIENDALKRLKLMVDDIYFAFTVAFVPRLWPHLNAYNDFILAWHSSGFDKFWEWKIAAEYMNAHRQNRIVASEKTNLDIGPVKLGIDNFIGLILLWCFGMICSLLTFLGELWRGQG

>DmelIR76b

MATGIELLVAAALCVACPPLNDSPPTNLIQMGENGTLSPVTELPMDVDASEAGFDADAPVETLETINRKKPKLREMLDWIGGKHLRIATLEDFPLSYTEVLENGTRVGHGVSFQIIDFLKKKFNFTYEVVVPQDNIIGSPSDFDRSLIEMVNSSTVDLAAAFIPSLSDQRSFVYYSTTTLDEGEWIMVMQRPRESASGSGLLAPFEFWVWILILVSLLAVGPIIYALIILRNRLTGDGQQTPYSLGHCAWFVYGALMKQGSTLSPIADSTRLLFATWWIFITILTSFYTANLTAFLTLSKFTLPYNTVNDILTKNKHFVSMRGGGVEYAIRTTNESLSMLNRMIQNNYAVFSDETNDTYNLQNYVEKNGYVFVRDRPAINIMLYRDYLYRKTVSFSDEKVHCPFAMAKEPFLKKKRTFAYPIGSNLSQLFDPELLHLVESGIVKHLSKRNLPSAEICPQDLGGTERQLRNGDLMMTYYIMLAGFATALAVFSTELMFRYVNSRQEANKWARHGIGRTPNGQSVAPSRWLRGWRRLNSGHGQLLGASTHGQNVTPPPPYQSIFNGGSHGDPLNRWRRPLANGNALGNGVLLGGDSEGGVRRLINGRDYMVFRNPNGQSQLVPVRSPSAALFQYSYTE

>DmelIR84a

MIKLQVKVISWPLIILTAFLRVLQIESINTNFLELAAFEDFLRSEHLSHVLVVRGDDADGDWKIECHQKLLANYRVQFYRPEMSANFEDLMFYGSPRTAVLVLNSEHVLVRRQVFGVASEAGYFNNSLAWFILGSGRESLPVEQLIDQLLSGYRMGIDADITVALRGPDNASMLFYDVYRISRQANTPLIIEKKGLWTHSGGYQKFGNFKNTWVIRRRNFLNVTLIGSTVLTEKPPGFGDMEYLADDKQLQQLDPMQRKTYQLFQLVERMFNLSLAISLTDKWGELLDNGSWSGVMGQVTSREADFAVCPIRFVLDRQPYVQYSAVLHTQNIHFLFRHPRRSHIKNIFFEPLSNQVWWCVLALVTGSTILLLFHVRLERMLSNMENRFSFVWFTMLETYLQQGPANEIFRLFSTRLLISLSCIFSFMLMQFYGAFIVGSLLSESARSIVNLQALYDSNLAIGMENISYNFPIFTNTSNQLVRDVYVKKICKSGEHNIMSLQQGAERIIQGRFAFHTAIDRMYRLLLELQMDEAEFCDLQEVMFNLPYDSGSVMPKGSPWREHLAHALLHFRATGLLQYNDKKWMVRRPDCSLFKTSQAEVDLEHFAPALFALALAMVASALVFLLELFLHWLPDFRRRLGTMST

>DmelIR92a

MLLQPLVMHLSQLLRIIVGQYFAEFPSILIVYNNSASTTPLQLEYLSALELVLRELSKPIRLQWINVAFLKDLNDLEDQVMGALNSSVTEGFITILSQTHHFIHARYYATRNANVRLKDKRYLFLCEDESPAELLCMDILQFYPHHLMVRPGTETAPTGPTGPHPDPRRGGGASVSTKNKDDGEGGAGNKTTSPYRDINFELWTQKFVGAVGNLDALLLDAFLPNETFANRVELYPNKLLNLQRRSLLVGSITYVPYTITNYVPAGQGDVDPIHPQWPNRSLTFDGAEANVMKTFCQVHNCHLRVEAYGADNWGGIYDNESSDGMLGDIYEQRVEMAIGCIYNWYDGITETSHTIARSSVTILGPAPAPLPSWRTNIMPFNNRAWLVLISTLVICGTFLYFMKYVSYRLRYSGTQVKFHHSRKLEKSMLDIFALFIQQPSAPLSFDRFAPRFFLATILCATITLENIYSGQLKSMLTFPFYSAPVDTIEKWAQSGWKWSAPSIIWVHTVQSSDLETEQILARNFEVHDYSYLSNVSFMPNYGFGIERLSSGSLSVGDYVSTEALENRIVLHDDLYFDYTRAVSIRGWILMPELNKHIRTCQETGLYFHWELEFIDKYMDKKKQEVLMDLANGHKVKGAPQALDVRNIAGALFVLAFGVAFAGCALVAELLIHRMDLSK

>DmelIR93a

MNPGEMRPSACLLLLAGLQLSILVPTEANDFSSFLSANASLAVVVDHEYMTVHGENILAHFEKILSDVIRENLRNGGINVKYFSWNAVRLKKDFLAAITVTDCENTWNFYKNTQETSILLIAITDSDCPRLPLNRALMTVECRINAVVFVDQTILEENALLVKSIVHESITNHITPISLILYEINDSLRGQQKRVALRQALSQFAPKKHEEMRQQFLVISAFHEDIIEIAETLNMFHVGNQWMIFVLDMVARDFDAGTVTINLDEGANIAFALNETDPNCQDSLNCTISEISLALVNAISKITVEEESIYGEISDEEWEAIRFTKQEKQAEILEYMKEFLKTNAKCSSCARWRVETAITWGKSQENRKFRSTPQRDAKNRNFEFINIGYWTPVLGFVCQELAFPHIEHHFRNITMDILTVHNPPWQILTKNSNGVIVEHKGIVMEIVKELSRALNFSYYLHEASAWKEEDSLSTSAGGNESDELVGSMTFRIPYRVVEMVQGNQFFIAAVAATVEDPDQKPFNYTQPISVQKYSFITRKPDEVSRIYLFTAPFTVETWFCLMGIILLTAPTLYAINRLAPLKEMRIVGLSTVKSCFWYIFGALLQQGGMYLPTADSGRLVVGFWWIVVIVLVTTYCGNLVAFLTFPKFQPGVDYLNQLEDHKDIVQYGLRNGTFFERYVQSTTREDFKHYLERAKIYGSAQEEDIEAVKRGERINIDWRINLQLIVQRHFEREKECHFALGRESFVDEQIAMIVPAQSAYLHLVNRHIKSMFRMGFIERWHQMNLPSAGKCNGKSAQRQVTNHKVNMDDMQGCFLVLLLGFTLALLIVCGEFWYRRFRASRKRRQFTN

>DmelIR7a

MFHHLWLLMGLRSLAMGALHPPQPEAMTPLVAAALEILAEQVSPSQSTLAVMDLTQDAEHRDERQEQLMTIILRSVGSEMALRTFQKPPAEVPASFVVFLVNSAQAFNTLGFHFTDIHSTREFNFLILLTHRMSSRAERLQVLRDISRTCVRFHTSNVILLTEKRDGVVLVYAYRLLNMDCDLSVNLELIDIYKNGLFRHGHEARSFNRVLSLSGCPLQVSWYPLPPFVSFIGNSSDPEERAQIWRLTGIDGELIKLLASIFDFRILLEEPCNKCLSPDIKDDCSGCFDQVIISNSSILIGAMSGSHQHRSHFSFTSSYHQSSLVFIMHMSSQFGAVAQLAVPFTVIVWLALVVSSLLLVLVLWMRNRLVCGRSDLASHALQVLTTLMGNPLEARSLPRSSRLRILYAGWLLLVLVLRVVYQGKLFDSFRLPYHKPLPTEISELIRSNYTLINQEYLDYYPRELTVLTRNGSKDRFDYIQGLGKEGKFTTTSLIATMEYYNMMHWSTSRLTHIKEHIFLYQMVIYLRRHSLLKFAFDRKIKQLLSAGIIGYFVREFDACQYRKPFEEDYEVTPIPLDSFCGLYYISLIWLSAAVVAFILELLSQRIVWLRRIFE

>DmelIR7b

MKYWLYILSCCSLVASTMESSSDWDLAEALAQVVANSEMGRFKTLYIYTHTNSQSTGGHLEELLDQVLMIVPNNLQARRLLLQQSMEYKPYVHAVLALVDGLPSLSAIYARIRATQDLSHTLIYMSMPTDAYGEEMQATLRFLWRLSVLNVGVVLRPPGDHILMVSYFPFSALHGCQVISANVVNRYQVGTKRWASQDYFPSKLGNFYGCLLTCATWEDMPYLVWRPDGSGSFVGIEGALLQFMAENLNFTVGLYWMNKEEVLATFDESGRIFDEIFGHHADFSLGGFHFKPSAGSEIPYSQSTYYFMSHIMLVTNLQSAYSAYEKLSFPFTPLLWRAIGLVLILACLLLMLLVRWRHHHELPRNPYYELLVLTMGGNLEDRWVPQRFPSRLVLLTWLFATLVLRSGYQSGMYQLLRQDTQRNPPQTISEVLAQHFTIQLAEVNEARILASLPELRPEQLVYLEGSELQSFPALAQQSGSSARVAILTPYEYFGYFRKVHPMSRRLHLVRERIYTQQLAFYVRRHSHLVGVLNKQIQHAHTHGFLEHWTRQYVSAVDEKDESVARIASTSYSTLDGIDGDPSLSESEEDQQVAPVRQNVLSMRELAALFWLILWANLGAVVVFVLELLLPRIKLRKILRKMKKSTRASATTTSTLSSPSTTKDIPFSCKDGFQDSWPKCSLLVS

>DmelIR7c

MLHSAVHNVSLVYALVWAIDNYYGMATSTPLAVVQFPTSRESRRLHNDLIDAALGRSSGTGRIQFLLEDDRVEMTETDTDPPPPSGLTGRPIAIWFLDSLRSYFRLEMYLNQLGSPYKRNGFFLVIYTGLEDQPMESLKIMFRRLLNMYVLNVNVFLQRDGTVHLYTYYPYGPHHCQSSLPVYYTAFQDLAAPANGFGLTKPLFPRKLTNMHGCEMVVATFEHRPYVIIEDDPKTPGGRSIHGIEGLIFRSLAERMNFTIKLVEQKDKNRGEILPDGNFTGILKMMVDGEVNLTFVCFMYSKARSDLMLPSTSYTSFPIVLVVPSGGSISPMGRLTRPFRYIIWSCILVSLIFGFVLICLLKITALPGLRNLVLGRRNRLPFMGMWASLLGGLALYNPQRNFARYILVMWLLQTLILRAAYTGQLYLLLQDVEMRSPIKSLSEVLAKDYEFRILPALRTIFKDSMPTTNFHAVLSLEESLYRLRDEDDPGITVALLQPTVNQFDFRSGPNKRHLTVLPDPLMTAPLTFYMRPHSYFKRRIDRLIMAMMSSGIVARYRKMYMDRIKRVSKRRNLEPKPLSIWRLSGIFVCCAGLYLVALIVFILEILTTNHRRLRRAFNVINRYAA

>DmelIR7d

MDIRCVVALLLGLCKVQAVVWPHQHLLEEQLASQISATLQKIFINGLAVYNFGVFISTSYEEMDRDRVILVHQVLNRNLYPPNFPVAVVLASKMNRKITAQVFTQLLFVQNAEQAIAIAEGVNRNGLCVIVLLTSQPERPIMTKIFTYFMQERYNINVVILVPRLHGVQAFNVRPYTPTSCSSLEPVEIDIKDGDLWDVFPRRLKNLHGCPLSVIVWDIPPYMRINWKSSDPMDGLDGLDGLLLRIVARKMNFTLKLIPNEPNGLIGGSSFMNGTFTGAYKMLRERRANITIGCAACTPERSTFLEATSPYSQMSYIIVLQARGGYSIYEVMLFPFEKYTWLLLSTILGLHWIVGSRWRMPSPILAGWMLWIFVIRASYEASVFNFIQNSPVKPSPRTLDQALSGGFRFITDHASYRMTLKIPSFQGKTLISAGQPVDVFDALLKAPWKTGAFTSRAFLADHLVRHRKHRNQLVILAEKIVDNMLCMYFPHGSYFAWEINKLLFNMRSFGIFQHHSQILAWDNLPTTTDTDTPGKRIHSSTESVATGFAESMSFVVAALNCLMGALCISIVVFGLELLSRRRHWTGLEWLFERV

>DmelIR7e

MNHINEFVARAVLHVVHHYILSVTPSLVLTLCCRSNHTCNFYNKMMSTLFREWGLAPLQIVNVLRGVPWHPVPGRRHFNVIFTDSFAAFEEIRMEYYSREYNYNEHYFIFLQARDRLLQGEMRLIFDYCWRYRLIHCSIQVQKSNGDILFYSYYPFGEHGCSDMEPQLINRYNGSMLVEPDLFPRKLRNFFGCPLRCALWDVPPFLTLDEDQEEVLRVNGGYEGRLLLALAEKMNFTIAVRKVHVNMRDEALEMLRRDEVDLTLGGIRQTVARGMVATSSHNYHQTREVFGVLASSYELSSFDILFYPYRLQIWMGILGVVALSALIQLIVGRMLRERMGSRFWLNLELVFVGMPLLECPRSHTARLYCVMLMMYTLIIRTIYQGLLYHLIRTHQLNRWPQTIESLVQKNFTVVLTPIVQEVLDEIPSVQHMRFRLLEANSELDPLYFLEANHQLRQHVTASALDIFIHFNRLSADKVHQRGEQGSGAHFEIVPEDIISMQLTMYLAKHSFLIDQLNEEIMWMRSVGLLSVWSRWELSESYLRNEQSFQVLGTMELYAIFLMVLVGLIVGLLVFILELVSMRSIYLRKLFT

>DmelIR7f

MQGEDANLYVARALRLVIENVLAQLSTTLVVTISTRHLGTAHWFEYMMNILMDSWRMVAVQLLRIRPDLVVNPVPGRKRVSLLMVDSYQGLLDTNITASNANFDDPDYYFIFLQARDHLIPKELQLILDHCLAHFWLHCNVMIQTAQVEVLVYTYYPYTADACQKAYPIPVNTFDGRKWKASQMFPDKLSQMHGCPLTVLTWHQPPFVELVWDPKHNRSRGSGFEIQLVEHLARRMNFSLELVNIALLRPNAYRLAEGSSEGPIEKLLQRNVNISMGYFRKTARRNQLLTTPMSYYSANLVAVLQLERYRIGSLALLVFPFELSVWMLLLLALLIHLGIHLPSARRGNEEDGGGGLQVVALLLGAALARLPRSWRHRFIAAHWLWASIPLRISYQSLLFHLIRLQLYNTPSFSLDQLLAEGFQGICTANTQRLLLEMPQLARDPDSIQSVDTPFDWDVLNVLTRNRNRKIFAVANQDVTLSFLHSSAHPNAFHVVKQPVNVEYAGMYMPKHSFLYEKMDDDIRRLDASGFIHAWRRASFASVHRKEQVHMTSRRYINHAKLSGIYMVMAGLYLLAGLLFAGEVLLRQRN

>DmelIR7g

MNVTSLLNFESMKYIGAQTQAASINHHVAQALRVFIEDFYQRIAPAFIVVLSCRRPSPMNFYRNIMQLLYESVDTMIVQLVLVELGRPRRIAGPRTHNLLLVDSLDALLDIEIHTYTAQSDTSEYYFIFLQQRDALIPHDMQGVFAYCWRHQLINCNVMTQSSGGQVLLHTYFPYAPGQCNDSQPTRINMFLGESWKHRDYFPSKLHNLNGCPLIVLARKVSPFLDLDEGQRELRGLEGRLLQELSRRMNFSIQFSGLQDQLKNRTTWTEKQLLQKLVQERIAHLAIGYVRKRIQYATNLTPVFPHYSNRVVGCLLLNAHNLTSLEIWSFPFQALTWICLVAGDRLALVLAVYAASLGLPIDPPERPSLQLLFASWLIFGLIVRSMYSALLFFILRYHLHQRLPGNLQDLTHGDYAAVMGRTTLQDLREVPSLQDLLGLKSVIVTSEREEEVLRTLDRCTLREGAGSHPLFFGLISQDALLHLTQRGHRAGAYHIIPQDVLEQQLAIYLQKHSHLASHLDHLVMSIRSVGLVHHWAGQMASERYFRSRFLYREKRIRQPDLWAVYILTAGLYLLSLVVFICELLASRRAGL

>DmelIR10a internal deletionMAVLGTVFLLFMLDLKTLNLTRLNGLLVEPTRDLPQLELWLRAGSDHQDAENPYVQWFLLRTEIPLSIVTYQENRYWMDDPFGRRNLVLVMSLDQLLTNRGAAAPIQKASTFFYILADQDKDLSADEQLRLEGSCRQLWTQHKVYNRFFLTRDGVWIYDPFKRRDSAFGRLVRYYGSETLDKLLFRDMAGYPLRIQMFRSVYTRPEFDKETGLLTRVTGVDFLVAQMLRERLNFTMLLQQPEKKYFGERSANGSYNGAIGSIIKDGLDICLTGFFVKDYLVQQYMDFTVAVYDDELCIYVPKASRIPQSILPIFAVGYDIWLGFVLTAFACALIWLTLRVINLKLRIVSLGNQHIVGQALGIMVDTWVVWVRLNLSHLPASYAERMFIGTLCLVSVIFGAIFESSLATVYIHPLYYKDINTMQELDESGLKVVYKYSSMADDLFFSETSPXWNRDLRADVIDEVARFRNKAGVSRYTSLILESSHFTLLRKIWVVPECPKYYTISYVMPRDSPWEDAVNALLLRFLNAGLIVKWIQDEKSWVDIKMRSNILEADAESELVRVLTIGDLQLAFYVVIGGNLLAFLGFLAEHFRWKLQKKGV

>DmelIR11a

MRFAILWLFSGCLLPGIQVGIWVVVRAQPTGRDVLLSRLGNQQNELNTRRLANASSYLTRNYIANRINTLVVREICVECPYELSERQRQLVDQILASLAPELSVLLHKGTAEETTWEYTLFVVNDHTAFTGQVFIFPDELLEREFFCIVVVSEIQSRQFVRQTVGSIVKSNLQMHFVNVVVVAQLEDGTVGTYSYKLFKANCTPGITVRQINHFDRITGKPQQSMPDLYPVRNGHLGDCPFNVGAAHMPPHLIYKRHKDPPPASNVSIPAEDLAGIDWDLLQLLAKALKFRIQLYMPQEPSQIFGEGNVSGCFRQLADGTVSIAIGGLSGSDKRRSLFSKSTVYHQSNFVMVVRRDRYLGRLGPLILPFRGKLWGVIIVILLLAVLSTCWLRSRLGLSHPIEDLLTVIVGNPIPDHRLPGKGFLRYLLASWMLLTLVLRCAYQARLFDVLRLSRHRPLPKDLSGLIKDNYTMVANGYHDFYPLELTCRQPLDFSARFERVQRAAPDERLTTIALISNLAYWNHKHPNISRLTFVRQPIYMYHLVIYFPRRFFLRPAIDRKIKQLLSAGVMAHIERRYMQYENKRKVASNDPVLLRRITKSIMNGAYRIHGLVIVLATGMFILELLAGRSNGRLRRWMEWVHQ

>DmelIR20a

MLASLNRSTGLSAELLDLYGLVVHFLLSGEHTTLVYFNPAGLDCSWGVLWQRNLTAHPQIVWQRNYSYPDLYYQFNAKLLVLACLPMDSRAAIQLEILANSLSHLRTVVRLLIEVAGPDQVTLARQYLSFCLRRSMLHVELYFRDYHHSLILYSFRAFPSFELVMRWISVGQGVKLFLHKLDDLRGHRLRVIPDLSPPNTFFYRDARGDNQVTGYLWDFLATFAGRLNAGLEVVRPSWRAGSASDSSYMLEYSAKGLIDVGLTTTLITKWNLWAIHQYTYPLLVSSWCTMLPVEKPLATPDLFGRIVCPTLAMTLLLIILVTWLVFRQLRCLTRLKNSRPARIVPHLLTLLLLTTCSAQLLSLLIFPPYHVRIASFEDLLRGDQKILGMRNEFYNFDGAFRARYAGVFYLIDDPNELYDLRNHFNTTWAYTMPYIKWLVIKTQQRHFSKPLFRWSKDLCFFDFMPTSVIVAPDSIYWESIKDFTFRIHQAGLMKHWIRKSFYDMIKAGKMSIKDYSDLETLKPLNIGDLEIVWRVCGAAIAVASAIFIMELLYFYINVFFNSL

>DmelIR41a

MFIDLSWSLVLSAIVGKYLNESTICIFWNDKFEFQLLHKSDYISFVGINIKSFDDNGGHYIIDTGLKKKELQNKHLFLDELVIKIIISIEVTHCETFVVFDKDIDRFVNAFNKASVYSIWRSLHNKFVFAHIANESPESRNHFFEDQPNILFVVRDHSSASSFDIKTNKFVGRKAENPSQMILVDRYLASEQRFQFGKSLFADKLNNLQGREVIIAGFDYPPYTVIKHNMSTNAQDMGVSGESDFKNVYIDGTETRIVLNFCEQFNCTIQIDSSAANDWGKVYPNMSGDGALGMLINRKADICIGAMYSWYEDYTYLDLSMYLVRSGITCLVPAPLRLTSWYLPLEPFKETLWAAILLCLCAEATGLVLAYKSEQALYVLPGYREGWWTCTSFGVCTTFKLFISQSGNSKAYSLTVRVLLFACFLNDLIITSIYGGGLASILTIPSMDEAADTVTRLRFHRLQWAANSEAWVSAIRASDEALVKDILYNFHIYSDDELLRLAQDQHMRIGFTVERLPFGHFAIGNYLGPQAIDQLVIMKDDIYFQYTVAFVPRLWPLLDKLNTLIYSWHSSGFDKYWEYRVVADNLNLKIQQQVQETMTGTKDIGPVPLGMSNFAGFIIVWILGSAIATLTFLLELSLTYILKQSNLK

>DmelIR47a

MRQIKLLVWLLVVGVVSSTEQLQFLKNFLEAVHKERSISTILLIQRKVHKNDFLHGLYPIFWPIICLDETKRVELVNNFNKDFLALVYMESEADTLLLSALAADLNHIRDARIMIWLQMSPSENFLDRIVFQASKQKFLNLVVIENTLKTRRFYPFPQPKVQVIDKPFEEKEIYPALWRNFMGKNAIAVPDLVPPRSFNSFDPKTGHRRESGSIYNVFKAFTQRYNITMLLKWPLIRNTTQEEIIGKSVRGEIDLPITGQLISFRHPNGSRSQPLLGMTALSIAVPCGPELPMFDRFFLFYGLATPITITGYYVLLNTIEIILGTLSDRIKRHPRRKKILNLVLNLRVFSCILSLPTPQGNRLRSVKGQLTMVMSITGLILSCIVAAQTSTILTMKPQYRHIKNFQELSDSNITVVCNHLNYLTIKQQMDPKFMAKFMQNIWIVNSIEQMKMIFDLNTSYAYQTFSYKKDPFTLLQMHTTRKAFCRTPGLDLVSGLAYTAVLEKNSIYALALQDYTLKAFSAGLVYYWAEESIRDLISTVGRTQFEKLPIVIGYQSLKLQDYNVCWKILLIGGALAFCVFIVEVVVGLINRRI

>DmelIR47b fragment

MREAQIIIFLLTSAAAVTLKQYEFLXSFLKAGEQEQTITTLLMMQKHVHTKNLLQGLYPXPWPIIHFVETQRIKFIALLYMSSEKDIFLSSLAANLKFERLDKPFGKSNIFPVLWRNYMGXIALTLDHLVEPRSFYWTDPRTNIKRRTGYIYMLITNFAEQHNITLQLXSPPNEDMSQMVIIERTHKGPRSTHNWADDQLETFERXQDSLLPWHGSMAIVVPCGQEMSAYERFHAAHAFRAPIIFFGFHIFLSLIDFLLRTISDRIRCNPRRIQLLQTVLSLCVLRCILSTSLPNSNXLRSRLRDNSPXXXVLQAXSYSALWXLTGTAXQXHNRDFQSHKLHDYXTTDGSXHSIEVPGLLKARNXXIXLFHIFSSLGTKFDLRIGSAGSHTSGVEFRYYELLDRXSSLENNIVSQVFTILKLPYSRFRVLKLEDCRGCWQTLFVGFSIATFVFIVNVLMGFFRNINQKK

>DmelIR48a internal deletion

MHLLITETYMIIGKTLHDILNELNERLIISTNIIFCKQFDNLIHFEAQTSRFVYSSLEAFNITSLWNHVGNDNKLFVIVGNVPPYELFAKLELSSPENCTQFILNNTVDMCADALVKNSKAFSVSRELRIAPANVIVPHGKPLLSYRYLAAPFNTKVWIALGTYVFLISGFLCLIHWLRSGKWDFSQNLLEVYSSLLFTVFHLKATNGIERYILFGVLFISGFVYSTSYLRLLKSMLIAETFEKQIQTFEELAESNIPLLINPYDRMIFQHHHIPKSLWTAVRTVSSETLLNHRSHGYVRLCPAILTASKIPSHTHRHLFSVCRFSHEQEVVPKGSSXXSLVPCIRKRNREXNHLGCLSGVSWPGISXFFHYGALGGEAFGSILLHDANYFPSPRLFRRLAELHYGSY

>DmelIR48b

MILQQSSNLLKLLLLLAISSVRTQGLNDIIIELNQRLLISNNFLYCNQSDKLNEYEIKYL

QHMPPISLMIFTSIESMNFTQVEYNLGADNKLFLIMGNEEPPYDFLHALNLHFQFAEYIIVIDEPVDLKKSTKWLDFVNHLWQQGYVQLLIYTSYDEKLYHKIIFPETVIEETLVEQYISIRGSFNNLYGYPVRVAAYNNAPRSMLYVNRWGKHIFAGFYMRFLRAFIDARNGSFVPVLTPSNSPGNCTLNLVNETVDVCADALAANPAAFSLTHGFRIASANVLVTHAKPLHSYRYLTAPFQWSVWACLVIYVLLVVNFLSFIGWLRSGKWEFSKYLLEVFSSLLFSGFYLKEIRGRERYILFGVLFIAGFVYSTEYLGLLKSMLISEVFEKQIDTFEALVESNITLMVDPYDKILFAKYNMPEILSPIMELVSFETLLKHRNRFDQDYAYILFSDRMALYDYAQQFLKHPKLLRIPIDFSFLYTGIPMRKRWFLKHHLGRAWYWAFESGLTRKLALDADFEAVRVGYLSFLITEHVEAQPLNVDYFVMPAIALAIGYILALLSFVIEMTAWRIREFLGCRKATMTSTGCSEGGHVDVD

>DmelIR48c

MSLLRIILIIIFLRIVSSIPDTIISHLSAELQIKIQIYFGLGNDLYDFSRLDGNYQKIIISHNISEEFKTYHDEPVLIIIRLERDLNLNLATLDVLRSYLTDRQYNDILLIDNDEENLNSYVDIRKAYWNAGFSQVLIYNSQQRTWSIKPYPYLQIRPTSLKEYIENRNTRNLMGYPLRVLVTNDPPHCFVDKDELPGSPNRYKGSIVTMLKIFADQLNATFQANPFREFRRYSTADCVQMVSDDEIDACGSIFIRTYTYATSQPVRLNRVVIMAPFGNPIEKFYYFFRPFDLYVWIGTGIIVVYIAVMGSLLHRWHFKEWNVGQYLLLAVQTLLNRELSLPQSSSGSKFMLLLLLFAIGFILSNLYVALLSMMLTTKLYQRPIENLADLKAANVNILLQTHNIRPNSVYGSSEELRERFLLVEESQHLEKRNGLDPSYAYVDSEDRMDFYLYQQKFLRRRRMKKLSNPVGYTWAVQVIKQNWVLEKHYNDHVQRFFETGLQNKLVDDVHELAVKAGFLHFFPTQTQTIEPLRLEDIVMAAMVLGGGHALAVICFLVELFA

>DmelIR51a internal deletion

MYNVLVLFLLLFTRAQMEPHRRGHNMTLLRSVLTVIRGRENWKNTPIFLGGHCNSDDLNNLMSWLQNTMEVTCHTVDTSTSAKNENALGHFNINADNSLGLLFCQSSHELIWFNMDKRLRRLRGIRLIVILSDKRSSSSKAIMSTFKRLWHFQFQXNFQGYVVSTPVENDIPRVFFVKDKKTGRKQIRGFGYRTFVEYLHRYNASLHVSNSQQEHAINSSVNMGRIINQIVDGQLEISLHPYVDVPENMGDNSYPLLIASNCLIVPVRNEISRYMYLLLPLNQSSWILLLGSVIYISGVLYYIQPGLLHRTWDQRIGLNILDSISRIINICSPSRIYNPSLRYFIVSVHLSILGFVVTNLYSIMLGSFFTTLVVGEQVDSMQQLIQXQQKVLVKYYEVSTFLRHVEPDLVDGVAQLLVGVNASEQVSALLGFNRSYAYPFTLERWEFFSLQQQYAFKPIFRFSSACLGSPIIGYPMKSDCHLQSSLNMFIMRIQAAGLLRHWVVSDFNDAMRAGYVRLLENFLGFHSLDVDSLRLRWAVLLCGWLLSTLIFLCER

>DmelIR51b

MCKVLTLLVVILLLALTNAAYNVTLLKSVLSLISTREPWINTPIFVGHNTQGGDLNDLII

WLHQTMGVTSLTMNLFLQPEHIRPLGHFKITRYNGIALFFCHDKHDIMWLTLDRNLRKLRRIRLIIILRNQRSGSQGAIKSIFNALWQYQFLNVLVLQRDQLYSYTPYPAMRFFKLDIHTEPLFPHAARNFHGYVVSTPAENDIPRVFHVHDPLTKSRKVLGYAYRTFVEYLDHYNASLRLTNPDENLDPTTSVNMNHIVQLIIDGQLEISLHPYVFTPPTATKSYPLLIYPNCLIVPMRNEIPRHMYLLRPFQLYSWYILLFAVFYITGILYCISPKLNKSSWPQRLGLNFLDAISKILFISPPITIYRPTWRHLIIFLQLSVLGFMSTSWYNIELDSFFTTIVVGEQVNSMDQLVHQQQRVLVKEYEINTFLRHVEPRLVEKVSRLLVPVNASEQVSALLSFNRSFAYPFTEERWQFFAMQQQYAFKPIFRFSSACLGSPHIGYPMRVDSHLETSLNHFILKIQDTGLLNHWVVSDFNDAMRAGYVRFVDNVLGYQSIDVDTLRLGWCVLGIGWILSALVFSCEYWHLYPWRFIA

>DmelIR52a

MALGWSVIILGFIGQLSAQILNYTQSRDLELLEGSLFRVLSRLNLEEEYNTLLIYGKECVFHSLLRKLEISAVTVPSGSTDYDWSFSTAILILSCGYDAENEENSYTLMKLQRTRRLIYLEDNSEPESVCMRYSLKEQHNIAMVKSDFDQSDTFYSCRLFQTPNYVEGHFFKDQPIYIENFQNMRGATIRTVADSLVPRTILYRDEKSGETKMMGYLGHMINTYAQKLNAKLHFIDTSKLGAKKPSVLDIMNWVNEDIVDIGTALASSLQFKNMDSVWYPYLLTGYCLMVPVPAKMPYNLVYSMIVDPLVLSIIFVMLCLFSVLIIYTQHLSWKNLTLANILLNDKSLRGLLGQSFPFPPNPSKHLKLIIFVLCFASVMITTMYEAYLQSYFTQPPSEPYIRSFRDIGNSSLKMAISRLEVNVLTSLNNSHFREISEDHLLIFDDLSEYLVLRDSFNTSFIFPVSVDRWNGYEEQQKLFAEPAFYLATNLCFNQFMLFSPPLRRYLPHRHLFEDHMMRQHEFGLVTFWKSQSFIEMVRLGLASMEDLSRKRNEEVSLLLDDISWILKLYLGAMFISSFCFILEILRCGERCKRLWRCRW

>DmelIR52b

MTWLVILLCFLGYMAAHIADISVQNQSLMDNELINLLLKLRNEEFYDTLLVYGKDCEFHSVIKNVDVAVVLVSDSMNFEWNFSSLTLILSCGPDIDNGGPNSTSIKLQRNRRLVLLKEDFQPSNICNIYTQKEQYNIALVRENFTKSKSIYTCRYFQDPNVDEVNLSGTKPIFIEQFQNMKGKAIRIVPDLLPPRVMLYQDANDGELKMIGYVANLITNFAQKVNATLQLDFLKPSTSITEISRMAKDDELDMGITLEASLNTSNLETSSYPYLLTSYCLMVQVPAKFPYNLVYALIVDPLVLGIIFVLFLLLSVLLIYSQKMSWQDLSVANILLNDKSLRGLLGQSFPFPLNASKKLRLIFTILCFASIMLTTMYEAYLQSFFTNPPSEPEICSFQDVGSYNRRIAMSALEVNGLIKTNNSHFREIRMDDLEIFDNMPECYELRDAFNLSYNYVVTGDRWRSYAEQQTLFKEPVFYFARDLCFSRLIFLSVPLRRHLPYRHLFDEHMMQQHEFGFVNYWMSHSFFDMVRLGLTSLKDLSRPLAYTPSLLMDDISWIMKIYLAAIVLCVFCFLLEIGVDKWKRWMKFRNLQILNTC

>DmelIR52c

MVWLIIILFCLGNSSSQILDVTNNSHLDFDYRLFGLLQRLQVEKSYDTLLVYGEDCAIPSLFERLQVPAVLVSSGSTNFDWNFSSLTLILSCNFQDEREENYRTLMKLQTSRRLILLKGHIKPESVCDFYSKKEQHNVAMVKENFYQLEVVYSCRLFQDQNYEKLNLFDGKSIYKDQFRNMHGAPIRTLSDKEPPRTIPYIDSKTGEEKFKGYVGMLISQFVKKVNATMQIREDLIKDDEEVSFVDITNFTSNDILDIGICEARTLEMSNYDAISYPYLMSSYCFMAPLPDSLPFSDVYMAIVAPSILIMFLIIFCICSVLIIYIQERSYRSLTIRSVLMNDICLRGFLAQPFPFPRQYNRKLKLIFMLVCFSSLISTTMYTAYLQAFLWGPPIEPRLTSFDDVKKSRYTMAINIYEREFLEALNVSLEDVEIYDYGKFSKLRSTFNTNYLFPVTALQWFTINEEQKLFKYKIFYYCDAFCLNQFDILSIPLRRHLPYRDIFEEHMLLQKEFGLTKYWIDQSYRDMIRANLTTFKDFSPLLENDYIEVHNLYWVFTMYFVGMGMGLCFFILEILRPLRYWRNCKIKCEYCYAFLKNFAK

>DmelIR52d

MVRIIIILLCLGYTKARILDATNTNHTDLEERLLSLLLRLQQEQFFNTLLIYGEDCAFSSLSRRLQVPTILVSSGSTSFEWNYSSLALILTCEFKAEREENYQTLKKLQMNRRLILLNGNIKPDSVCDFYSKKDQYNIAMVNNNFHQVGIIYACRLFQERNYEKVYLSEGNPIYVDQFRNMQGALLKSITFNLIPGSMAYRDPKTGQEKHIGYVANLLNNFVEKVNATLDMQVKLHKAGKKTSFYNITKWASEDLVDIGMSYAAYFEMTNFDTISYPYLMTSTCFMVPLPDMMPNSEIYMGIVDPPVLVVLIAIFCIFSVMLNYIKQRSWRSLSLVNVLLNDICLRGFLAQPFPFPRQSNRKLKLISMLVCFFSVITTTMYTSYLQSFMWGPPIDPKMCSFADLENSRYKLAIRRYDIEMLRPFNVSMDHVVVFDESSQLEYLRDSFDDNYMYPMSALSWSAFKEQQKLFAFPLFYYSEKLCLKPISFFSFPIRRHLPYRDLFEEHMLQQNEFGLSTYWIDRSFSDMVRLKLATMNDFSPPRLEDYIEVSDLSWVFGMYFTGLGISCCCFGLELLGLPSWTRRLRLTNWLRVRN

>DmelIR54a

MWTVITGIVLWAPVLVAGSAVDFIFRAAAEHSLSVIMIRIDYCPYNWAKDIFENQTIPVVVLSDSETFINIRMFSRPLHVACLPGHELQKDLALLENFTSSLMDFPSQKKIVYISNNFSDPTRMDYIFETCYHRRIWNIVGLLASDEHRYFYRYHLYPSFRTEYRSLESSTIFDKDFPNMHGHPLTVMPDQWLPRSVLYVDRRTGKQILAGSVGRFFHVLSWKLNATLQLSKKVTTGRFLNATALKELSESFSVDVPASLTIMERVEQLASTSYPMEVTHVCLMVPVARRIPIKDIYFILSSASNMFLAIVIVSSYGLALNLLRNMTHRDVRLVDFVLNDKALRGILGQSFNLPLSRSFSTRLIFLMLGIVGLNVSSIFGAGLDTLMAHPPRQFQARSFAGLRRTKIPLVTTEEDFPTWMKLRVPMLVVNVSEYNHLRNGRNTSNAYFASRLYWNLFSEQQKRFTRELFIYSTDDCLWSLALLSFQWPQNSLFTEPVSQLILEVNANGLYDFWVGMHYYDMTAAGLSGLEDPSLQLKEREHPTSLRIVDFQWMWQAYGTFMVIAILVFLLEVSWHRITSLFVSLVY

>DmelIR56a

MGSRFFIRNLILFGLLASSNMQIPFGELEKKFELDVDFLLGVTELVGHIQGLYSITVYADCIDIHPSIQQRIMDKFMVPVNTIGSNLSRPNYHKLDNSRIRIVLFTGLNDTILVNLNKTDVPYSDNFYMLAYASAIKNKCIELDFIEEVFTLLWKMSIQNAILLIRGEFMMEMWSYLYMGKIHKIKLTKPNSYLESLRKYNYRFSLEVINDPPAIFWYNSSEQADVTGGGNLSVSGPLGLIIINFLRHLNVTIDIVPIPGKQTSQYELFQQPDNLRAENGVNMVGSALLKYSPMVTQSRMCLLVSNRRMIPFSRFLDRLVSPGVHKLTFVSSIGIFVIKYFSHRPRSFVDAIFCTIRFFFAIPLPSIILNRLPVVDRFIEVFIIIFVQILLSSNISITTSALTTGFWEPPIINVETMRASGLHILTEDPTILQAFKENILPSSLADLVILVDEDTYFHHVTTLNNSYVYVVQAHNWQIFRLYQQQMTNEPFEIASEELCSKWRILGIPLNPKSPLRFMFKDYFYRILESGLREQWVHSGFKKFCEFNNLKKLPVDSVDSWQPLSIEFYSNVIRAYIIGLVIATLAFVAELLHNGYRRKNVKKT

>DmelIR56b

MLLDTDLASGVIRSPYSFDIPHAFIFNETQFVVPKFCGPYMEIVKHFAEVYHYQLFLDSLESLPKKSVVEQDIISGKYNLSLHGVIIRPEETSDFFNATQHSYPLELMTNCVMVPLAPELPKWMYMVWPLGKYIWTCLFLGTFYVALLLRYVHWREPGNATRSYTRNVLHAMALLMFSANMNMSVKLKHASIRVIIFYTLLYIFGFILTNYHLSHMTAFDMKPVFLRPIDTWSDLIHSRLRIVIHDSLLEELRWLPVEYQALLASPSRSYAYVVTQDAWLFFNRQQKVLIQPYFHLSKVCFGGLFNALPMASNASFADSLNKFILNVWQAGLWNYWEELAFRYAEQAGYAKVFLDTYPVEPLNLEFFTTAWIVLSAGIPISSLAFCLELFIHRRKQRRPQYERFECYDY

>DmelIR56c

MRSSFRICLFLLTTYHPSHGWNMQHLLNLLAPFGRMNVFQEIVWFVSPHQRLDQLDEFIMRIDEAFGKSATQTVVNNNTEMRMIYSSARRNHMSFVFTTGAEDPIMKVFSKVLLGRHFYVSMVIYVDKVGDMHPIYDLLTFAYNQQFFNSMVHFESMEGVNQLFGVSKFPVMSFENRTDFLKYMGKIWKQVQNARSDVGGFGFTTPLRQDLPHLFQSQGHYDGSTYRIIETFVRFINGSFKELIMPPDSLGGQVINMKDALQLIRERKMEFCAHAYALFMSDEELEKSYPLLVVQWCLMVPLYNSVSTYFYPLQPFDWNVWFFALGALLALVLLELMWLRMFGGWSGYRGAVLNSFCYIINVPIEGQLQQPCLLRFLLLATVFFHGFFLSAYYTSNLGSILTVNLFHAQINTMNDIVSAQLPVMIIDYEMEFLLNLNKELPQEFLELLRPVDSAVFSEHQTSFNSSFAYFVTEDHWEFLDEQQKHLKQRLFKLSSICFGSYHLAFPLQMDSSLWRDIEYFTFRIHSSGLLNFYARSSFGSALHAGLVQRMPDTQEYTSAGLQHLAIAFILLLVMSFLAGIVFVLETLSR

>DmelIR56d

MDNRAAELILRERNIFPTNGSDNITLLNNMFVLEMFYRITQLYHFKNFIFYISERLDLNNKDSQEFFHNFWTYFPMAPNLIITREHHLGIPMMQFISTPSLVMVFTTGKDDPIMELASHNQQGIHWLKTIFVLFPSLQSRDFETNPESLAQFTAEIKDVYDWVWRKQFINTFLITIKDNVFILDPYPTPSIVNKTGVWQAEEFFHKYAKNMKGYLVRTPILYDMPRVFKSDRPTNRYEKNFIHGTSGNLFLGFLEFVNATLMDTSANVTADYLNMTNLLDLVSQGVYETLIHSFTEITTKFVVSYSYPIGINDCCIMVPYRNQSPADQYMHEALQENVWVLISLFTLYITVAIYLCSPLRPRDLSAAFLQSICTLTYSVPTFIIRTPTLRMRYLYILLAIWGIVTSNLYISRMTSYFTTAPPVRQINTVQDVVEANLRIKMLAIEYERMAKSPLQYPESYLNQVDLVDKHMLDLHRDPFNTSFGYTVSSDRWRFLNLQQLHLRKPIFRLTEICEGPFYHVFPLHKDSHMRSVMTEYIMIAQQAGLMNHWERETFWEAVHLHRIHVHLFDDEPMALSLDFFSSLLRTWTLGLILAGLAFAAEMKWHEHVTFKRRPVIRITRKPRSFLRRFMKL

>DmelIR56e fragmentERXAFRNQWAFCFPRTXAIEVVLSAWSPXCPGQRSKPQPISXPHHXGSCWRKRKWKXKPRLLVVDKRTLVEHLNSLNDGYAYCIIAGHWQVGMM

>DmelIR60a

MWCNNPGLIIIIFLGQILNLCQGIVNLSNETANTVIFMLPEKDLGPDVWKAGVGCLDSFAQIFFFRNPKERFTRAYNLMLVHAFHLSSPADQIQEGFSKLINEAVTNPGPPDREELFQMRVASDYNITNGTEDKGELILADNYVIVVDSVDRLKELMKKKIVEMRSWNPGARFLVLFHNATCRNRPLGVASNIFKDLMEMFYVHRVALLYANSTMNYNLLVNDYYSNVNCRILNVQSVGQCHDGKLYPNNAVVKASMQDYVSGFSPRNCTFFACSSISAPFVEADCILGLEMRILGFMKNRLKFDVNQTCSLESRGEMDGPANWTGLLGKVQNNECDFVFGGYYPDNEVADHFWGSDTYLQDAHTWYIKMADRRPAWQALVGIFEAYTWIGFILILIISWLFWFTLVMILPEPKYYQQLSLTAINALAVTISIAVQERPICETTRLFFMALTLYGLNVVATYTSKMIATFQDPGYLHQLDELTEVVAAGIPFGGHEESRDWFENDDDMWIFNGYNISPEFIPQSKNLEAVKWGQRCILSNRMYTMQSPLADVIYAFPNNVFSSPVQMIMKAGFPFLFEMNSIIRLMRDVGIFQKIDADFRYNNTYLNRINKMRPQFPETAIVLTTEHLKGPFFILVVGSCWAALTFIGELIIHRWRTQLVSTSEQQDRRSDKRRRRRRRRKPEKDNRWQRQVQVAPVVRFTPVKRRKVFQGQTSQK

>DmelIR60b

MRRSLYLIIAIGLVDVHCVSLRYILNALENELQYRAILLVESASEIESCWEQKYIQGAVPILNFNANQSLYLKDALNTNILALVCLNENVESTMQALYENLEDMRDTPTILFVLSDSKVQDVFLECLRRKMLNVLAFKGLDRGFVYSFRAFPTFRVIERNVMDILQYFEQQLEDLGGHTLTTLPDNIIPRTVVYKSPDGSRQLAGYLYPFLRNYVSTINATLKVCWHLVPEDGMIQLGEVVRLSEIHDVDFPLGMHGIEHGSTSQNVPLEVSSWFLMLPMEPSLSRAQFFIMLGFEKVTPVLLLLTILLSTAHRIEMGLRPSWRCYVLGDRVLQGTLGQAFFLPRRLSVKLMLVYSLILLNGFTFSNYSITSLETWLVHPPSGHPIHSWEQMRTLNLKVLIVPSELDSMTKALGKQFTESNSDLFELSKSGNFQDKRLAMDQSYAYPVTCTLWPLLEHAQIRLPKPEFRRSREMVLIPLLIMAMPLPKNSMFHKSLNRYRALTHQSGLYEFWFKRSFNELVALRKIHYKVNGDHQIYRDFEWQDFSYVWLGFVGGTIASILVLLAEIGYHRWQLNQN

>DmelIR60c internal deletion

MEMRLALFFTFACLAGAHDGSLRNMLKSLEDELGYRTILLLEGFVYSFKAFPTLRVVKRRVKDVRRYFEPQLEDLGGCVLKVVPDGIMPRTMVYQGEDGELQMGGYLSHFIRNYVSTINASLQIRWDLFPEDGDFDMDSLTGSNHVDFPLGLGSLSFQTLHKDVAMEISSWFLMLPMEPSLPRARFFIRFGISLYLIPLIILLAIVLSNAHRFEAGLTPSWRCCSMGNTVLRGVLAQAFVLPKGLSPKLMFVYWLLLVSGFFVSNYVIVYLTAWLIQPPTSDPVTDFDQMRRAKLKILMVPTDMDYLKSIRGAEYVDAHSDVFQTADSTDFQTQRMSMELHFAFSVTGTLWPLLRQAQVKLHRPIFRRSKEMVFLPFVIMGMTMPNNSIFLSSLKQYRLRTSEAGLYLLWFKKSFSELVAIHKISYKEDWVHDSYSDLKWEDFLFAWLGFLGGTTVSCLALLAEIGYHRWLWKRTHQ

>DmelIR60d

MRLAIYVAFLSSIGNRSGFLSSLLMSLGKELHYKTILLVGGSSTCWSLEPFETGVPILNLRGENNAYPQDTFNSQMLALACLQTESEDAVKLLYRSLKDMRDTPTLLFASSEEHIHDTLFLGCFRENMLNVLALTASSKEFIYSYQAFPTFRVIKRKLVEIHRYFEPQLKDLGGHIVSALPGNIMPRTMCYRNAEGERQLAGYLNTFIRNYVESINGTLRISWGLVPEDDMRHLTISRLSKIQHVDFPLGIIPLYNKTDKQHVYMEISSWFLMLPMETSVPRAHLFVKLGLERLLPIIVVVGAVLGNAHRIEVGLGPSWRCYYLADKVLRGALAQPIVLPRRLSPKLMLIYSLLLLSGFFLSNYYMASLTTWLVHPPASDRILEWDQLRYLHLKVLTIPEEFKYMSLILGTDFMTAYGSIFQLTNSTDFQRRRISMDPSYAYPVTTSLWPFLELSQVRLRRPLFRRSYDMVLQPFQVMSLPLPRNSIFHKSLLRYAALTRETGLYYYWFRRSYYELVALGKISYKEEEGNPYCDLKWNDFRIVWLAFLGGTIISCLALLLEVAHYRWHLGNSSL

>DmelIR60e internal stop codon

MVIKMISFLLVSVLLCLVGASDSESMQVQVLQDLNLALQTELNVFIDFECCATSEILHKLDSPRILLSSNSREARDLRIRGNFTESTLIIVSVMDSDLNPLVASLLPRLLDELHELHIVFLSNEEPGFPKQDLYTYCFKEGFVNVILMSGKGLYSYLPYPSIQPISLSNVSEYFDRARIIRNFQGFPVRILRSTLAPRDFEYSNEQGGLVRAGYLFTAVKELTYRYNATIESVPIPDLPEYDVYLAVAEMLHTKKIDIVCYFKDFSLEVAYTAPLSIIREYFMAPHARPISSYLYYSKPFGWTLWAVVISTVLYGTVMLHLAARGARVEIGKCLLYSLSHILYNCHQKIRVAGWRDVAIHGILTIGGFILTNVYLATLSSILTSGLYDEEYNTLEDLARAPYPSLHDEYYRSQMKAKTFLPERLRRNSLSLNATLLKAYRDGLNQSYIYILYEDRLELILMQQYLLKTPRFNMIRQAVGFTLESYCVSNSLPYLAMTSEFMRRLQEHGISIKMKADTFRELIHQGIYTLMRDDEPPAKAFDLDYYFFAFVLXTVGLISSLLVFFAELVSGHL

>DmelIR60f internal deletion

MRFHLNIANSGLLGLHLCPTRSALPEQNPCFSKAGAVIXNLTLPWRRWRERCLLGALRPXTLPTPELQCXSKYLPXRKSQQENASSGLPGFCXGDXQTELHRGSRAIALPRSPYHDLYYVWIAYLGGTMIGIGMLAVEIACFKWDLLRRPPIXMY

>DmelIR62a

MYLQFLFALFLSRYQIVATENFDRAFELALFLDRIGRVHRLHAITIVNSLGSVDPSYLDDLHRGLMCNSSNHFYMLPQMTATDKDSSHVHFSSLQDEETIYLVFARDSKDAVIYLQAERARGRRYTRTMFLLRKQESQKDIKYFFELLWKLQFRSALVVVAARNFYQMDPYPTVRVIRMRRLSSYDPHHVFPPANRKNFRGYRMRLPVQQDVPNTFWYKNRRTKAWELAGLGGILINQLMMHLNVTMDLFRFEVNGSSLLNMAALTDLIVKGKVELSPHLYDTLQSNTSVDYSYPTQVAPRCFMIPLDNEISRSLYVFLPFSLTMWLCLLFVLLVVHFVYVRRLIPDGHFWAILGVPGAGQVRYGNRKPVRRFSTFLILFGIFILGQTYSTKLTSSLTVTLIRRPDNSLEELFLLPYRILVLPTDVYAIVDSLGHAEQFSTKFSCTDAENFSQKRISMHPEYIYPISTIRWRFFDMQQRFLRKKRFYFSKICHGSFPYQYQLRVDSHLKDALHRFLLHVQQAGLHDLWLDTCYRKAHRMGYLKDFSTLAELEEKLRLRPLALNLLVPAFSLFLCGMLGSGIAFLVEIRHSFGCRQKPPSINRNPGD

>DmelIR67a internal stop codon

MLPILVPVLLLFNETSWINPILTSIYKDRHHETVLLLQHSQHGNASGLERFPWPVFSFNEQMDFYVRGKYNSEMLVLIWQTGNSDWDLDLWQALDRSLLNMRKVRVLLLRKWEKIPTADVAATAEHLLFLHVAVIGQGNRIYRLQPYAPQSWLQVDPIESPIFIKIRNYFGRYIVTLPDQFPPRSIVYRNPKTDEIQMTGYVYKFLLEFIRIYNFTFRWQRPIVQGERMNLILLRNMTLNGTINLAISLCGFETPSXLGVFSDVYDMEEWYIMVPRAQEISIADVYVVMVSGNFLIVLIIFYFIFTILDTCFGPLLLKERVDWSNLMLNERMISGIMGQSFNMSARNTISSKVTNATLFLLGLVLSTLYAAHLKTLLTKRPTSQQISNFKQLRDSPVTVFFEEAERFYLKHAWDRPIRYIKDQLNFRETIEYNALRMGLNRSNAFSALTSEWMIVAKRQELFKQPIFTVQPELRVIQTSVLLSLVMQSNSIYEDHINDLIHRVQSAGIVEYWKHQTLREMITMGMISQKDPFPYVAFREFKVGDLFWIWLLWVSFLFMSFVIFLCELLVDCFISKTLIRNKRPH

>DmelIR67b

MELLYLNTLQSLSLLEGNRLVQTVQELNNIYQTELNVFLEFGNGADILESAQGTFVPTLWIKNPQNQKVMKGNFTSCTLTILYLEDEHLDRGLYYLANWLWEYHHLEVLIFFNGGSYDKLIQIFSRCFNEGFVNVLVMLPGSDELYTFMPYQDLKILNLKSIKEFYSLSRKKMDLNGYNITSGLVIAGAPRWFSFRDRQNRLILTGYMLRMIVDFTNHFNGSVRLMNVLTVNDGLELLANRTIDFFPFLIRPLKSFSMSNILYLENCGLIVPTSRPLPNWVYLLRPYAFDTWIAWLIMLIYCSLALRILSKGQISISAAFLKVLRLVMYLSGSRDMGTRPTTRRLFLFVILTTSGFILTNLYVAQLSSNSAAGLYEKQINTWEDLDKSDSIWPLIDVDIKTMEKLIPDRTKLLKKIVPTLEADVDTYRRNLNTSCIHSGFFDRIDFALYQQKFLRFPIFRKFPHLLYQQPLQISAAFGRPYLQLFNWFVRKIFESGIYLKMKDDAYRHGIQSGLLNLAFRDRHLEVKSNDVEYYYLIAGLWFGGLTLATVCFLLELLIGYAKIKVTISCKMNIM

>DmelIR67c

MFCWLIFLNIILLSDRSESWSAREVIHQFNHDQQLQLNIYLDCNDVELQIGQEVSNLFVNSTADKMKILGRFSSHSLIIACFKDSTRNRTLNGVKELLWGLQYLPILFVVDSNMDFYFQQALRHGFIHVLALNFMNGSLYTYKPYPKVEVHQIKDMQKFYKLTKLRNLQGQAVRTTVETMTPRCFRYRNRHGQLVYAGYMYRMVKEFISTYNGTEEHVFGNVDTVPYKEGLAALKNGEIDMMPRIIHALEWYYFYRSHILYNIKTYIMVPWAEPLPKSLYFIQPFRGTVWITIMVSFVYASIVIWWIRYRQQGNSSLTQSFMDVLQLLFQLPLSKIWHFNMGTHQVVSFIVLFVFGFMLTNLYTAQLSSYLTTGLFKSQINTFDDLFREKRTLLVESFDAEVLHNMTKEKIIQKEFESIILITSIEEVFKHRKSLNTSYAYEAYEDRIAFELSQQRYLRVPIFKILKEVYDQRPVFVALRHGLPYVELFNNYLRRIFESGIWIKLQEDSFLEGIASGEISFRKSKSREIKIFDKDFYFFAYILLGMGWCVSTIALFLELWSFKYSVTNVLHEG

>DmelIR68a

MRCLWILIVAFISLAMATSIPIPIANPAPLSGYEMQLKILLQKILWVANVKRCFAVITDDLHYPIYDRIFFESVGRRVIPFFVMRTNESDDLQRPSRQVELFVKAIKSSDCELNVITILNGWQVQRFLGYIYDNRSLNMQKKFVLLHDLRLFESDMIHLWSVFIDAIFLKRQLDNKYTISTIAFPGILSGVLVMKNIANWELGKGLNGRILFADKTSNLFGTSLPVAISEHVPMVLWANATKSFQGVEVEIMNALGKALNFKPVYYKPNQTENMDWTELDGGASVAYGSGNPDGYAQNGTHIDSMLVDEVAAHSARFAIGDLHLFQVYLKLVELSAPHNFECLTFLTPESSTDNSWQTFILPFSAGMWVGVLLSLFVVGTVFYAISFLNAIINGNVSSEFFRCLRPNRNVPMDPKIYRRISFRIAISRYRSSKGDRMPRDLFDGYTNCILLTYSMLLYVALPRMPRNWPLRVLTGWYWIYCILLVATYRASFTAILANPAARVTIDTLEDLLRSHIPPSTGATENRQFFLEANDEVARKVGEKMEVFGYSDDLTSRIAKGQCAYYDNEFYLRYLRVADESGSALHIMKECVLYMPVVLAMEKNSALKPRVDASIQHLAEGGLIAKWLKDAIEHLPAEALAQQEALMNIQKFWSSFVALLIGYVISMLTLLAERWHFKHIVMKHPMYDVYNPSLYYNFKRIYPQH

>DmelIR68b

MKFLVGLLLQWYLPGIYALAEIACRIAVEQNVQVTYLYRCASCPASFDADYSALELDLYRCVGSRLPVITRNMEAHELEPFRRTDSLSIFQIPAAEKGDSLVRRILDMLNPHQRRKHMHKYLFVWPNAGRHQLLRLFRGSWAKKLLYGLAITGRENGTFDFDPFAWGGLQVIQRLDGEVPYARKVKDLRGYPLRFSMFTDPLMAMPRSPVETAGYQAVDGVAARVVGEMLNASVTYVFPEDNESYGRCLPNGNYTGVVSDIVGGHTHFAPNSRFVLDCIWPAVEVLYPYTRRNLHLVVPASAIQPEYLIFVRVFRRTVWYLLLVTLLVVVLVFWVMQRLQRRIPRRGVIQFQATWYEILEMFGKTHVGEPAGRLSSFSSMRTFLMGWILFSYVLSTIYFAKLESGFVRPSYEEQVDRVDDLVHLDVHIYAVTTMYDAVRSALTEHQYGLLENRSRQLPLGIATSYYQPVVRRRDRRAAFIMRDFHARDFLAITYDSQAERPAYHIAREYLRSMICTYILPRGSPFLHRLESLYSGFLEHGFFEHWRQMDLITRVGASPDAEEFLEDLGDQTDTDSGSNELAIRNKKVVLTLDILQGAFYLWSVGIGISCLGFAVEHAHWFWRRQTLRNAVEARTS

>DmelIR85a

MSIQWLKHILLLAILVNLAGTRENHIPLDLKKSSIVMVKMSQILCKARIKVLFVYFENQTSHEHTGQILKEVTKCDISNQNTPLEAVKDDGILMYMVMITTNISQPLELSLIRKKSAAKHRSHVFLLVRDADTVSDAWMRASFRQFWKIWLLNIVILYWRDGRLNAYRYNPFMDNYLIPVDNKPNEVPTLEQLFPKTIPNMQRKPLRMCIYKDDVRAIFWRQGTILGTDGLLAAYVAERLNATMMITRPHSYNNHNLSSDICFLEVAKEYVDVAMNIRFLVPDTFRKQAESTVSHTRDDLCVIVPKAKTAPTFWNIFRSFGSLVWALILVSVLVANVFCYILKSEVGRVPMQLFAGALTMPMTQIPPNHSIRLFLIFWLYFGLLICSAFKGNLTSMMVFQPYLPDINQLGALARSHYHIIIRPRHVKHIQHFLTLGHKHESRIREQMLEVSDTQMYEMMRNNDIRFAYLEKYHIARFQVNSRVHMHLGRPLFHLMNSCLVPFHAVYIVPYGSPYLGFLDSLIRSSHEFGFERYWDRIMNSAFIKSGVKVVNRRRGSGNDEPVVLKLQHFHAVFALWLVGIGMACIVLAWEHLTHNYNLAVTKRRD

>DmelIR87a

MSTPEQRFWLAALLFLLSQHSEVRGFGINLMKVQTEDKGQEACILALLRKYFDSGDGLSGSVLCINRNYQLPNIEEQLLRGVNNYENYPWSLLITNSREGPSPAKFLMNEKPQCYFLIVDNLEDEDLDEVFEHWKGMVNWNPLAQFVVYLASLEETDEEMNDLMVELLLTFINKKIFNVNVIGQSEENQFYYGKTVFPYHPDNNCGNRVISVELLDACDYPSEETDSEDENDEDEGDGAQEEDDGPQEEGDGEQEEEDGPQEQEDGDQAKGDEGQENDDGGLENKVENEFRIGASDDDELENDLSSNSSEPEAIIEEFFRAKFEDKFPRDLSGCPLTASFRPWEPYIFRNSEEQPVDDYYYGLQGDEDDYNDTSPNYGESDDESYADPGEDGDGAIPDTETQSGGKLKLSGIEYEMVQTIAERLHVSIEMQGENSNLYHLFQQLIDGEIEMIVGGIDEDPSISQFVSSSIPYHQDELTWCVARAKRRHGFFNFVATFNADAGFLIGIFVVTCSLVVWLAQRVSGFQLRNLNGYFPTCLRVLGILLNQAIPAQDFPITLRQLFALSFLMGFFFSNTYQSFLISTLTTPRSSYQIHTLQEIYSNKMTVMGTSEHVRHLNKDGEIFKYIREKFQMCYNLVDCLNDAAQNEHIAVAVSRQHSFYNPRIQRDRLYCFDRRESLYVYLVTMLLPKKYHLLHQINPVIQHIIESGHMQKWARDLDMRRMIHEEITRVREDPFKALTFDQFRGAIAFSGGLLLVASCVFAFELCYVKYVYRTEKRERKTKKITKKVHNIKIQHD

>DmelIR94a

MALPKQLKFINIFLVLLIIYGSSDGTENQHEIFLNRLLQAVHNERSVETLFLLHHSNLANCSLQDWNPPRIPTIRSNELTVFNVEKTFNHNALALVCLMKNSYREILNTLAKSFDCMRQERIILMIHRKSDSKFIEDITHEVKNLQFLHLIVLIVQEKYNGQVFASTLRLQSFPEPHFKRIRNVFAIQRIFYRPINFHGKVLNAIPNDIPILFVALNEMFTEYARRYNSTLRIQNRTIKEDIEITEDNYDIDMKIQLHNSQNFLHHMNIAMDIGSNSLIILVPCATELRGLDIFKELGVRTLTWLALLFYIIFVLVEMLFVFISNRFNGRNFTMRYTNPLINLRAVRAILGQTSPISNRYSLSIQHFFVFMSLFGTLFGGFFDCKLRSFLTKRPYYSQIENFSELRKSGVTVVVDHTTRQFIEQEINANFFRDEVPNVRTTTIQELINHVYSYDRKFAFVANSIPWRTFREEMKSINQKILCDSKNLTILENVPLTFSIRRNAIFSHHLRNFIINAADSGMITCWFKMAGKVIRKHIKTTLRESEQQPSHLPLSFDHFKWLWAVLCIAYVMSFMVFVMEILWSKYQRRTRSVSIV

>DmelIR94b

MSLIFNLLFILILSQAVSQETEFLQLKYLNNIVRSMIKLHKMETLVIVKHHLDNNCSLQNWNAHGMGIIRTNDQGKLIMKDTFNSRTLAIICIGQNSHITLLRNVFETFGKVQQKKIILWTQMELKEKFFQEISKKSRDLKLLNLLVLKAVTKDKLLIYRLNPFPSPHFKRIENIWTPNDTLFMDTKFNFHGMTAVVKHDYNWTIQMGNIRKFPISRIEDKEVIEFALKYNLTLQFFNDVERFDIELRKRIILKSNSTQPIDSGIPMVFSSLLIVVPCGNYLSIQDVIKVSGIEKWIFYIILVYVIFVLIEITFLGVTILISRQSRHQMIPNTLVNLCAFRAILGLPFPETRRTSLSLRQLFLAIALFGMIFSIFINCKLSSMLTNPCPRPQVNNFEELKTSGLTVVMDHDAENFIEKEIGVDFFNQYMPRKVTLTFTERAKLLFSLKGNHAFTLFSESFAIIESYQRSKGLRAHCTSEDLIVAERVPRIYILENNSILDRPLRRFIRQMQESGITNHWLKNIPSSLEKNLMQITIPYDRERVHPLSIEHLTWLWCILILGYSISMIVFFVEMSLKRRKKNLENRAPNICIC

>DmelIR94c

MSKVFKLLVLPLIYLSLTKGSKNPQLKFLRELINVIEEGREIRTIMVIKHSRDEYCHLDQWNPRGSPILRTNEMGSIRISGYFNDQAVILACMGENSDYGLLKSLANAMDNMRQERIILWSEREPTKMLMDYISQQADRYNFAQIIIVTMNEDVDAVPSLHQLNPYPTPRFRQITNISNIRRTSFFGCGLSFQGKTAILKESVVSNIRFKVWSPSGPIPLSELKDYEIVQFAVKYNLSLKLYDQNESKSDHFDIQLGPLFITKDFPTQMAFVSPNTACSLIVIVPCSPKWRFMDVLHKLGVLKLIGCLLIAYAVFVLIETLILWLTHRISGREVRLTSLNQLLNPRAFRGILGLPFPEFRRSSISLRQLFLVISVFGLVYSNFVSCTLSALLTKPAQNPQVRNFKELRDSGLITIMDKYTHSFIEKHIDPEFFDHVLPHYLILQKKEALRMIWNFNDSYSYVMYTTTWKSLNTVQKSFDERVFCESESLTIAWNLPRMYVLGNNSVLKWMLSRYITYMPQTGIPDSWTEQLPKVLKLLYNVTSPRRIKEGAVPLSIQHLSWIWHLLFIGESIATLVFIVEILLQKSNQHTSNMRERSSEDDDFV

>DmelIR94d

MGQLHLLLVALVLLSPGGDSFYHSLIHHLNRELKIEYVLLLGNFDTTWLDILWQLPVSVLQIKEHSRETYSLLENPSHNVLTIAFVNDSPEDILEILYRNLRMLNTQPVLLVIRKSTIRVNSLLEWCWHHQLLKVVAIAQDFMESLIVYSYNPFPVLQFIERRLDNSTVIFEKRLENLHGYEVPIALGGSSPRLIVYRDLEGKLIFSGPVGNFMKSFEQRYNCRLVQPYPFDESAISPARDLIASVQNGSVQIALGAIYPQVPYTGYSYPIELMSWCLMMPVPEEVPHSQLYSMVFSPMAFGITIVAMVLISLTLSMALRLHGYRVSFSEYFLHDSCLRGVLSQSFYEVLRAPALIKAMYLVICLLGLLITSWYNSYFSTFVTSAPRFPQLTSYESIRHSNIKIVIWKPEYEMLLFFSENMEKYSSIFQLQEDYKEFLHLRDSFDTRYGYMMPMEKWSLMKEQQRVFSSPLFSLQDDLCVFHTVPIVFPMVKNSIFKEPFDRLILDVTATGLLSRWRDMSFTEMIKAGQLGLEDRGHPKEFRAMKVGDLIQIWRFVGWMLGLATIVFLLELICFWRHKMWQNMKYMFCRNKNI

>DmelIR94e

MDCPKWILSGLCLISLVSGATVIELLGTLKLELDFEYVLLMKNRNFSLSDQVWNGTSLTKDVMDEVQVPVLQFNENVSYFLHNSISRRLVTLGFMSDANLDEHRGLLTALVANLRHMTTSRVIFLVQSKASTDFLYELFRNCWRKKLLNVIVIFQDFETTSTFYSYSNFPILQIEERIYETSLQTLPIFPDRLRNLHGYEMPVILGGTAPRMIAYRNKKGNVVYDGTVGHFMTAFQQKYNVKFVQPLQAKNPLDFAPSMQTVGAVRNETVEISISLTFPTIPPFGFSYPYEQMNWCVMLPVEADVPPFEYYTRVFELAAFLLTLGTLVLISCLLASALSLHGYATNISEFLLHDSCLRGVLGQSFVEVFRAPTLVRGIYLEICVLGILITAWYNSYFSSYVTSAPKQPPFRTYDDILASKLKVVAWKPEYAELVGRLLEFRKYETMFLVEPDFNRYLALRDTLDTRYGYMITTNRWVLINEQQKVFSRPLFQKRDDFCFFNNIPFGFPLHENSVFMEPVQKLIMELAETGLYYHWITTGFSELIDAGEMHFVDLSPHREFRAMQIQDLQYVWYGYAFMVVLSSLVWLLENLAYTVKSKTIFPTHFMQRNKK

>DmelIR94f

MSGMWQQVLLAETSNWFRSDVLQRFWTHLRVEIRFRTMLNYRLESCDCWFDNVLGSDNSTALLWNDQTYPHYLRRRQDTDILVVSCLRFHQYQEVLLALSLMLDQMRSMPVVLQLCGDEDSMQELNSARLLLKHSQDLKMPNVVLLSSTFFTSATLYSYEMFPEFNVQKLVYQAYLTLFPYKLGNLKGHPIRTVPDNSEPLTIVRKTLNGSIAIDGLVWQFMIEFAKHINATLQLPIEPHPEKSIKLVQILDLVRNQTVDIAASLRPYSLNVQRSSTHIYGSPMMVGNWCMMLPTERVIGSHEALTRLMKSPWTWLILLLFYSVHRFLAQKTRLRSSLIHLIKLLINLSLICFLQAQLSAYFIGPQKVNHISNMQQVEESGLKIRGMRGEFMEYPIDMRSRYASSFLLHDLFFDLAQYRNSLNTSYGYTVTSVKWELYKEAQRHFRRPLFRYSEEICVQKLSLFSLIQQSNCIYCYRSRIFILRMHEAGLIRLWYRRSYYVMVTAGRFPIGDLSTVHRAQPIRWTEWQNVVLLHGVGLLFSVVVFVIELTVHYANVCLNNL

>DmelIR94g

MSTAVNSVHSKLVSLISRGQELTSIFFYAPAKEKCHLEDTISSATWGLPLVIWRTDRTVILNGFIGEGLLVLACLPGFHWRALLGSLARSLKYLRQARILIELMQDRDEFLVSEVLQFCLSQDMINVNAIFDDFPETENLSSFEAYPSFEVVNQTFTPDTQVSDLYPNKMLNLRGGVIRTMPDYSEPNTILYQDKEGNKEILGYLWDLLEAYAHKHNAQLQVVNKYADDRPLNFIELLDAAQSGIIDVGASIQPMSMGSLSRMHEMSYPVNQASWCTMLPVERQLHVSELLTRVIPYPTLALLLLLWIFYEVLRGRWRRHSRLQSIGWLVLATLVSSNYVGKLLNLFTDPPSLPPVNSLAALMESPVRIISIRSEYSAIEFTQRTKYSAAFHLALHASILIGLRNAFNTSYGYTITSEKWKIYEEQQKRSSKPVFRYSKDLCFYEMIPFGLVIPENSPHRAPLHSYTLLLRQAGLHDFWVNRGFSYMVKAGKINFTAVGERYEAKTLTITDLRNVFIIYVSVLLISLILFTCELFVSWVNYWLGF

>DmelIR94h

MLSNISFSSAPELVDLYGLVLKFLVSSETTLFYFNPTGQKCSWETLPRTILSNHPQIIWFREETYPGLYKRHSSNLFVMACLSSTSYDGQLQLLAESLTRYRSVRVLIEVQDKEGSFLASQILLLCQQHSMLNVVLYFSRWTRTLNVFSYLAFPYFKLLKQRLSGSLRPKIFINQLKDLQGYKIRVQPDLSPPNSFSYRDRHGECQVGGFLWRIVENFSKSLKGDTQVLYPTWAKAKVSAAEYMIQFTRNGSSDIGVTTTMITFKHEERYRDYSYPMYDISWCTMLPVEKPLSVEILFSHVLSPGSALLLILAFILFFLIVPQLIKCLGITFRGRLIGMASRIFALVMLCSSSAQLLSLLMSPPLHTRIKSFDDLLTSGLKIFGIRSELYFLDGGFRAKYASAFHLTENPNELYDNRNYFNTSWAYTITSVKWNVIEAQQRHFAHPVFRYSTDLCFSSETPWGLLIAPESFYREPLQHFTLKINQAGLITQWMTQSFHEMVRAGRMTIKDYSRTNLMKPLRIQDLRKCWVIFAVGLGTSTVVFTIELLLIYTNVFLNSL

>DmelIR100a

MATTLQLIMLALVGGTLGQANNTDHKQVLTSIVKQLEGGLELHLRTSEDGGNDLVQFLMQEKSSIIISAKQEEVPSRAKIMRHHFFIFDGVHQMQEIRTSLFNTDGFYILALENNTIEDDVLLMEFAADVWLQHGHSRIYYVQLSKKSVLLFNPFLQRLVVVQDSKTYSRIYKDLEGYHLRIYIFDSVYSSVIGDGENKVLSVTGADAKLAKTVARQLNFTADFVWPDDEFFGGRLANGEYSGGVGRAHRGEVDIIFAGFFIKDYLTTHIQFSAAVYMDELCLYVKKAQRIPQSILPLFAVHMDVWLCFLLVGLLGALVWLILRAVNLILGIEGVPDGSRATRISYFGAARRIFVDTWVIWVRVNVGRFPPFHSERIFVASLCLVSVIFGALLESSLATVYIRPLYYRDVNTLRELDESGQPIYIKHPAFKDDLFYGHNSEVYRRLDAKMMLVAEGEERLIEMVSKRGGFAGVTRSASLQLSDIRYVMTKKVHKIPECPKNYHIAYVLPRPSPYLEEVNRIVLRLVAGGIVGLWTGEAKERAKWSIQRFPEYLAELDVGRWKVLTLSDVQLAFYALTIGCLLSAIVCMAEILLGRQRRLHSPK
